# Ecological lags govern the pace and outcome of plant community responses to 21st‐century climate change

**DOI:** 10.1111/ele.14087

**Published:** 2022-08-26

**Authors:** Sebastián Block, Marc‐Jacques Maechler, Jacob I. Levine, Jake M. Alexander, Loïc Pellissier, Jonathan M. Levine

**Affiliations:** ^1^ Institute of Integrative Biology ETH Zurich Zurich Switzerland; ^2^ Department of Ecology and Evolutionary Biology Princeton University Princeton New Jersey USA; ^3^ Department of Environmental System Science Institute of Terrestrial Ecosystems ETH Zurich Zurich Switzerland; ^4^ Swiss Federal Research Institute WSL Birmensdorf Switzerland

**Keywords:** alpine plants, competition, demography, disequilibrium dynamics, thermophilisation, time lags, transient dynamics, transplant experiment

## Abstract

Forecasting the trajectories of species assemblages in response to ongoing climate change requires quantifying the time lags in the demographic and ecological processes through which climate impacts species' abundances. Since experimental climate manipulations are typically abrupt, the observed species responses may not match their responses to gradual climate change. We addressed this problem by transplanting alpine grassland turfs to lower elevations, recording species' demographic responses to climate and competition, and using these data to parameterise community dynamics models forced by scenarios of gradual climate change. We found that shifts in community structure following an abrupt climate manipulation were not simply accelerated versions of shifts expected under gradual warming, as the former missed the transient rise of species benefiting from moderate warming. Time lags in demography and species interactions controlled the pace and trajectory of changing species' abundances under simulated 21st‐century climate change, and thereby prevented immediate diversity loss.

## Graphical abstract

We forecasted trajectories of alpine plant community dynamics and quantified how they are shaped by ecological lags using field‐parameterized models of climate‐dependent plant demography. Ecological lags controlled the pace, trajectory, and long‐term outcome of changing species’ abundances under simulated 21st century climate change.

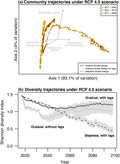

## PEER REVIEW

The peer review history for this article is available at https://publons.com/publon/10.1111/ele.14087.

## INTRODUCTION

Climate change is altering the distribution of species, the composition of communities, and the functioning of ecosystems around the world (Walther 2010). These changes are likely to accelerate, posing a growing threat to global biodiversity and ecosystem services (Urban, 2015). While we have increasingly reliable forecasts of the trajectory of climate change under alternative greenhouse gas emission scenarios (Kirtman et al., 2013), we generally lack forecasts of the trajectory of ecosystem change under these climate scenarios (Urban et al., 2016). This is because when climate change is rapid, we do not expect ecosystems to be in equilibrium with their current climate. While species' physiology can respond quickly to changing climatic conditions, it takes time for demographic and ecological processes to translate physiological responses into changes in species' equilibrium abundances and distributions (Alexander et al., 2018). As a result, ecosystems are undergoing transient dynamics, approaching climate‐driven equilibria which are themselves in continuous flux.

The pace and trajectory of ecosystem dynamics under climate change are shaped by time lags in demographic, ecological and evolutionary processes. For example long‐lived individuals may persist at locations long after climate becomes unsuitable for recruitment (i.e. extinction debt; Dullinger et al., 2012), whereas, due to slow dispersal and demography, some species' ranges are still expanding into locations which became suitable after the end of the last glaciation (Svenning & Skov, 2004). Ecological lags arise when, for example species that initially benefit from a change in climate, later suffer when their competitors rise in abundance (Suttle et al., 2007). Finally, evolutionary lags may cause populations that initially decline to later recover once they adapt to their novel environment (Carlson et al., 2014). Understanding and quantifying these lags will be necessary to accurately forecast the impacts of climate change on the diversity and composition of ecosystems.

Current approaches to studying the biological impacts of climate change fail to quantify the processes underlying lags, and are thus insufficient to forecast ecosystem changes over the coming decades. Space‐for‐time substitutions (e.g. climate niche models) rely on extrapolating current correlations between climate and species' distributions, assuming the two variables are (and will remain) in equilibrium (Elith & Leathwick, 2009). By neglecting the role of lags, space‐for‐time substitutions may reveal the long‐term fate of a community under a given climate (Elmendorf et al., 2015), but not the rate or trajectory of change. Manipulative experiments can be used to expose species to the novel climatic and biotic conditions they might experience in the future (Alexander et al., 2015), but necessarily impose abrupt changes in environmental conditions which do not reflect the more gradual trajectories of real climate change (De Boeck et al., 2015). Whether abrupt climatic changes simply lead to accelerated responses relative to what we would expect under gradual climate change, or instead lead to different transient trajectories or even different equilibrium outcomes, remains poorly understood. Moreover, since most experiments only last a few years, the results rarely reflect the equilibrium states we can expect under novel climates (Epstein et al., 2004). Indeed, the initial, short‐term responses of plant communities to climate manipulations can be modified (Hollister et al., 2005) or even reversed (Zhang et al., 2017) due to indirect effects of climate change mediated by changing species interactions (Suttle et al., 2007). These indirect effects can arise either from changes in the abundance (Suttle et al., 2007) or identity (Nomoto & Alexander, 2021) of interacting species, or from changes in the nature of their interactions (Olsen et al., 2016). Despite their potential importance, lags in changing species interactions remain poorly understood and their impacts on community trajectories under climate change are rarely quantified.

Combining the complementary strengths of experiments and models can generate better informed forecasts of community dynamics under climate change (Kotta et al., 2019). Experiments can quantify how climate affects species' demography and their interactions with other species. In turn, models of climate‐dependent community dynamics, parameterised with the experimental data, can predict community responses to the trajectories of climatic change forecasted by general circulation models. The effect of time lags can then be quantified by comparing the trajectory of these responses to those of a ‘no lag’ simulated community that stays in equilibrium with its climatic conditions. Similar to mechanistic niche models (Briscoe et al., 2019), this approach is empirically challenging, since it requires exposing replicated communities to different climates and quantifying effects on species' demography and interactions. While not feasible in many communities, the compact turf structure and high species density of many alpine grasslands enables the transplantation of replicated, multi‐species communities to lower elevations, where climatic and biotic conditions already resemble the conditions expected in alpine regions with future climate change (Tito et al., 2020).

Here, we use high‐resolution maps of alpine plant community change when transplanted along an elevational gradient in the Swiss Alps to quantify climate's impact on species' demography and interactions with neighbours. The key insight is that, while climate‐induced changes in competitive population dynamics may involve multi‐year time lags, these lags arise from climate effects on the demography and sensitivity to competition of individual plants which are measurable in a short‐term experiment. With such experimental data, we parameterised models to project the dynamics of the community under alternative scenarios of 21st‐century climate change, and aimed to answer the following questions: (1) How will the diversity and composition of alpine plant communities respond to contrasting climate change scenarios? (2) How do responses to gradual climate change differ from responses to abrupt climate change typical of experimental manipulations? and (3) How do ecological lags affect the pace, trajectory and outcome of community responses to climate change?

## MATERIALS AND METHODS

### Transplant experiment

We conducted a whole‐community transplant experiment along the south‐facing slope of the Calanda mountain (eastern Swiss Alps). The focal community was an alpine grassland at 2050 m elevation, dominated by perennial forbs and grasses. At this elevation, the snow‐free period typically lasts from early May to early November. Plant growth peaks during summer (June–August), when the mean daily air temperature is 12.3°C (mean from 2019 to 2020, see below).

We selected transplantation sites in pastures at five different elevations (1000, 1400, 1600, 1800 and 2000 m), which are between ca. 0 to over 5.5 K warmer than the focal site at 2050‐m, based on the adiabatic lapse rate of temperature. This encompasses the range of climatic conditions that the focal plant community may experience over different scenarios of 21st‐century climate change (CH2018, 2018). We selected the sites to resemble the focal site as much as possible except for climate. The sites are only a few kilometres apart and have similar topography, soil depth (>20 cm) and calcareous bedrock.

In mid‐October of 2016, we identified three transplant source areas within the focal meadow community at 2050 m, all within 220 m of each other, with similar community composition, and with low grass abundance. Across these three areas, we haphazardly selected 50, 1‐m^2^ turfs, avoiding rocky areas. We excavated turfs to a depth of ca. 15 cm and transplanted 10 to each elevation following a stratified random design. Since we were interested in understanding the dynamics of alpine communities under climate change in the absence of species migrating from lower elevations, we lined the sides of the transplanted turfs with a root barrier to prevent encroachment from the surrounding vegetation. We also weeded recruits of lowland species from the turfs during subsequent plant community surveys.

After the summer of 2018, lush growth of the plants transplanted to the 1800‐m site revealed that this site was incomparable to those at other elevations. We, therefore, selected a different site at 1800 m into which we transplanted 10 more 1‐m^2^ turfs from the same source sites in September 2018.

### Climate along the elevational gradient

To measure climatic conditions along the elevational gradient, we deployed air temperature (DECAGON ECH_2_O Temperature/RH) and soil moisture sensors (DECAGON ECH_2_O TE Moisture/Temp/EC) at the five experimental sites from late May to early October in 2019 and 2020. With these weather data, we fit models of each site's air temperature and soil moisture as a function of weather conditions at the nearby meteorological station in the city of Chur (weather data available from Swiss Federal Office of Meteorology and Climatology portal IDAWEB, https://gate.meteoswiss.ch/idaweb/). We then used those models to predict weather conditions at the different sites during the entire experimental period.

### Plant community surveys

To monitor community dynamics and quantify plant demography under climate change in a way that allowed us to parameterise models, we made detailed surveys of the spatial distribution of canopy cover of plant species in the transplanted turfs. To this end, in the spring after transplantation, we fixed a metallic grid subdivided into 0.1 × 0.1 m cells to each transplanted turf. Every summer for the next 4 years (2017–2020), we subdivided each grid cell into four 5 × 5 cm quadrants and surveyed the inner 256 quadrants of each transplanted turf (leaving a 10‐cm unsurveyed buffer from the turf edge). In each quadrant, we visually estimated the canopy cover of each species present using an ordinal scale (Table S1.2). We started surveys at the lowest site (1000 m) in mid‐June and moved up the mountain over the summer in an attempt to map turfs at a similar stage of phenological development, close to the peak of the growing season at each site (Figure S1.3). While we tried to identify plants as close to the species level as possible, time constraints during the field season forced us to lump some species into groups varying in taxonomic hierarchy and functional meaning (Table S1.1). Hereafter, we use ‘taxa’ to refer to both individual species and the various groups used in the surveys.

### Defining and tracking individuals to quantify demographic rates

The spatially detailed plant community surveys were the source of our demographic data. All the shoots of a given taxon rooted in a single 25‐cm^2^ quadrant were considered a demographic unit. Shoots rooted in adjacent quadrants were counted as different units (creeping species in the genera *Thymus* and *Helianthemum*, which have adventitious roots and are thus difficult to assign to a single location, were excluded from the analyses). We referred to demographic units as ‘individuals’, although most species in the community are clonal and thus individuals are likely not genetically distinct nor physiologically independent. We used an algorithm to track individuals from 1 year to the next, accounting for potential small changes in location due to re‐sprouting and observation error. If an individual could not be tracked into the following year, it was noted as a death. Individuals that could not be linked with previous‐year individuals were classified as new recruits. The change in a surviving individual's cover from 1 year to the next quantified its growth.

### Climate‐ and density‐dependent demographic models

We fit statistical models that quantified how a taxon's three demographic rates from the plant community surveys—survival, growth and recruitment—related to climate and crowding from conspecific and heterospecific neighbours. Crowding was a distance‐weighted sum of neighbours' cover, according to a Gaussian interaction kernel (Adler et al., 2010) with most crowding exerted within a 10‐cm radius. Heterospecific neighbours could occur within the same quadrant as the focal individual (i.e. distance = 0), in contrast to conspecifics (potentially biasing heterospecific crowding to be stronger than conspecific crowding, hampering species' coexistence).

We used a logistic regression to model the probability of individual survival to time *t* (*s*_*t*_) as a function of mean temperature during the previous summer (*T*_*t*−*1*_), individual size (*u*_*i,t*−*1*_) and crowding from conspecifics and heterospecifics (*w*_*i*_ and *w*_*j*_ respectively), as follows

(1) $$\text{logit}\;\left(s_{t}\right)=\lambda^{S}\left(T_{t-1}\right)+b^{S}∙u_{i,t-1}-\alpha_{\mathrm{ii}}^{S}\left(T_{t-1}\right)∙w_{i,t-1}-\alpha_{\mathrm{ij}}^{S}\left(T_{t-1}\right)∙w_{j,t-1}$$

where $\lambda^{S}$(*T*_*t*−*1*_) represents the direct effect of temperature on survival, *b* is the effect of individual size, and $\alpha_{\mathrm{ii}}^{S}$(*T*_*t*−*1*_) and $\alpha_{\mathrm{ij}}^{S}$(*T*_*t*−*1*_) are the temperature‐dependent effects of interactions with conspecifics and heterospecifics respectively.

We modelled individual growth with a linear hierarchical function similar in structure to the survival model in Equation 1, including temperature‐dependent intrinsic rates and neighbour effects (details in section 2 of Supplementary Methods). However, the survival and growth models differed in how intrinsic rates and interactions with neighbours depended on temperature. Specifically, the intrinsic survival rate ($\lambda^{S}$) was a sigmoidal function of temperature with an inflection point dependent on soil moisture, while the intrinsic growth rate was a Gaussian function of temperature. Moreover, while the strength of positive or negative neighbour effects (*α*_*ii*_ and *α*_*ij*_) were linear functions of temperature in the survival model, they were constrained to be negative, and exponential functions of temperature in the growth model.

Finally, we modelled the recruitment probability (*r*) for each previously unoccupied quadrant with a logistic regression:

(2) $$\text{logit}\left(r\right)=\alpha_{\mathrm{ii}}^{R}\left(T_{t-1}\right)∙w_{i,t-1}-\alpha_{\mathrm{ij}}^{R}\left(T_{t-1}\right)∙w_{j,t-1}+\theta^{R}\;$$

As in the growth model, neighbour effects were exponential functions of mean temperature during the previous summer (*T*_*t*−*1*_). Additionally, Equation 2, with interaction coefficients constrained to be positive, implies that recruitment is a positive function of conspecific neighbours (*w*_*i,t*−*1*_, as propagule sources), while a negative function of crowding by heterospecifics (*w*_*j,t*−*1*_) (via competition). Intraspecific limitation also emerges in this model as recruitment is limited to unoccupied quadrants. We set the value of the background recruitment rate $\theta^{R}=-5$ to ensure a low probability of recruitment in quadrants with no conspecifics in the vicinity.

We fit all the models following a Bayesian approach, using weakly regularising priors (McElreath, 2020) and sampling the posterior distribution of the parameters with adaptive Hamiltonian Monte Carlo, as implemented in Stan 2.17 (Stan Development Team, 2018). We specified and analysed the models in R (R Core Team, 2019) using the package *rethinking* (version 2.0, McElreath, 2020). We ran four chains per model, with 500 iterations for warmup and between 1000 and 5000 iterations for sampling, and assessed their mixing and convergence with the R‐hat criterion. The R code for model implementation is available in Supporting Information S2.

We attempted to fit models for all 34 perennial, flowering plant taxa with at least 1000 observations across all years and sites. Of these, we successfully fitted all models for 24 taxa (Table S1.3, details about sampling problems in section 3 of Supplementary Methods). To test whether the fitted models could reproduce the species' responses observed in the transplant experiment, we simulated the dynamics of these 24 taxa across experimental turfs. Starting with the 2017 turf maps, we projected 3 years of community dynamics under the simulated time series of weather conditions and compared the predicted versus observed log‐transformed ratio of final to initial cover of each taxon in each turf (Figure S1.4; turfs from the 1800‐m site were excluded due to their later transplantation). Downstream simulations only included the 11 taxa for which model predictions were clearly better (≤80% sum of squared residuals) than a null expectation of no changes in cover (somewhat analogous to an *R* ^2^ ≥ 0.2 for model predictions). These were generally the most abundant taxa in the community, and together accounted for 44% of total cover across years and experimental turfs. We compared the observed and predicted trajectories of these 11 taxa in different turfs with a Principal Coordinate Analysis based on Euclidean distances using the R package *ecodist* (Goslee & Urban, 2007), and compared the trajectories of taxon diversity with the Shannon index.

### Climate change scenarios

We used the CH2018 climate change scenarios for Switzerland under three contrasting emission scenarios: RCP2.6, RCP4.5 and RCP8.5 (CH2018 Project Team, 2018), corresponding to optimistic, realistic and worst‐case climate futures respectively. We used scenarios for the Chur weather station (i.e. the nearest station to our experimental sites) to predict mean summer temperature at our 2000 m study site throughout the century (details in section 7 of Supplementary Methods).

### Projecting community dynamics with an individual‐based model

Having parameterised functions describing how species' demography depended on climate and interactions with neighbours, we used those functions to project the demographic fate of individuals (and the emergent community dynamics) under different scenarios of climate change (details in section 8 of Supplementary Methods).

To isolate the effects of climate change on community dynamics from experimental artefacts or imprecision in model descriptions of demography, we first simulated dynamics under constant climatic conditions (i.e. mean summer temperature at the 2000‐m site between 2017 and 2020) allowing the abundances of each taxon to quasi‐equilibrate (defined as either a total cover growth rate <0.05 or a total cover change <0.5% of turf total area, between two subsequent years). This quasi‐equilibrium community was the starting point of our simulations of community dynamics under future climate change.

Using climate time series corresponding to different RCP scenarios, we forecasted community dynamics from 2017 to 2098. At every time step, we recorded the diversity of the simulated community with Shannon's index. To visualise the trajectories of community dynamics under the different climate change scenarios, we conducted a Principal Coordinates Analysis based on Euclidean distances. Because simulation runs are not identical due to demographic stochasticity arising from the finite population sizes, we present the median and the 5% and 95% quantiles of 20 simulated trajectories.

To compare the trajectories of community responses to gradual change versus the stepwise change simulated by most climate change experiments, we ran simulations of community dynamics under climate conditions that from the beginning of the simulations (after the communities equilibrated to current climate) had the same mean and variance as conditions expected between 2088 and 2098 under the RCP4.5 scenario.

Finally, to assess the degree to which the trajectories of community change depend on ecological lags, we ran additional simulations with no such lags. In these simulations, we let the community equilibrate with the climatic conditions experienced each year (using the same quasi‐equilibrium condition as in the pre‐climate change simulations) before projecting the next time step.

## RESULTS

### Reproducing responses to experimental climate change with demographic models

**FIGURE 1:**
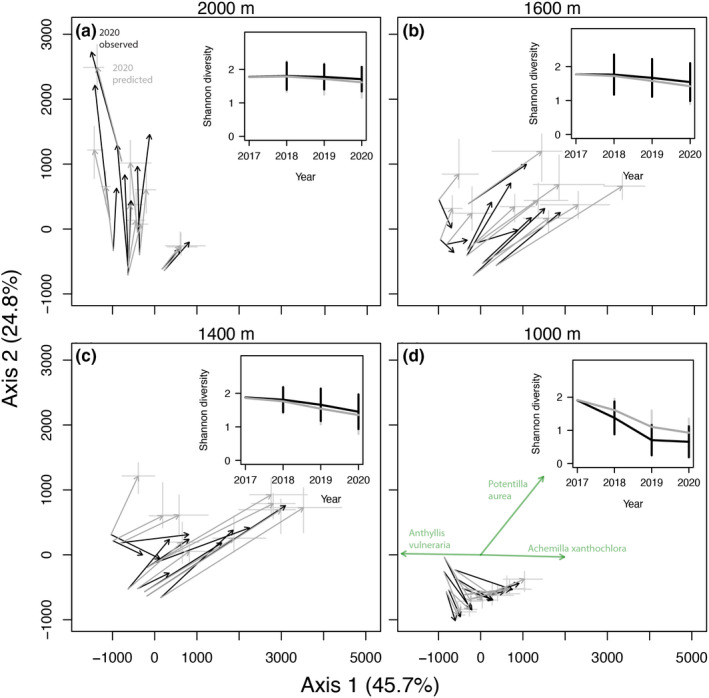
Trajectories of alpine plant communities transplanted to different elevations in the Swiss Alps to simulate climate change. The communities originated from a site at 2050 m and were transplanted to sites at (a) 2000 m, (b) 1600 m, (c) 1400 m or (d) 1000 m (which correspond to approximately 0, 2.2, 3.3 and 5.5°C of warming respectively). Panels show a Principal Coordinates Analysis ordination based on Euclidean distances of all observed and simulated turfs from 2017 to 2020, including only the 11 taxa for which models adequately predicted cover changes. The arrows show the observed (black) and median predicted (grey) changes in community structure of each turf. Grey lines centred on the grey arrowheads display uncertainty in the predicted community changes, showing the 5% and 95% quantiles across 20 simulations for each principal component axis. In panel D, the thicker green arrows starting from the origin show the taxa for which the first two ordinations axes explain more than 90% of their cover variation through time. The inset plots within each panel show the mean observed (black) and predicted (grey) Shannon diversity across the ten turfs transplanted to each elevation; vertical lines show 95% Wald confidence intervals.

The cover of most taxa declined sharply after transplantation to the lowest site (where summers are ca. 5°C warmer than at the original elevation), but responses to moderate climate change were more variable (Figure S1.4). While some taxa declined monotonically with increasing warming (e.g. *Vaccinium vitis‐idaea* and *Androsace chamaejasme*), others benefitted when transplanted to intermediate elevations (e.g. *Alchemilla xanthochlora* and *Potentilla aurea*). Our individual‐based model simulations of community dynamics predicted taxa's observed cover changes with varying success (Figure S1.4), but broadly reproduced observed changes in community structure and diversity for those 11 taxa included in the simulations under climate change (Figure 1).

### Simulated responses to 21st‐century climate change

**FIGURE 2:**
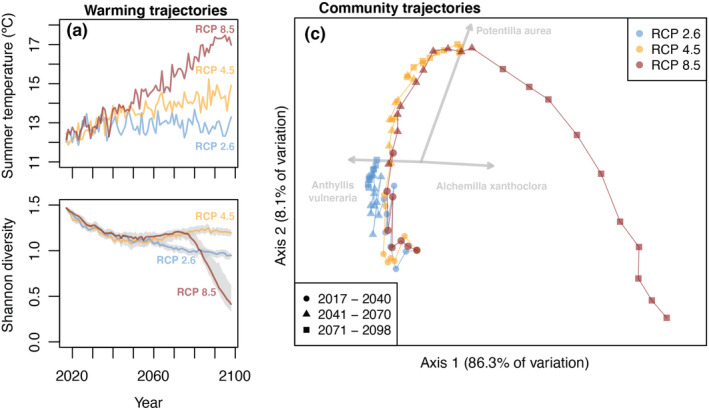
Trajectories of alpine plant community responses to contrasting climate change scenarios for the 21st century. (a) Depending on future greenhouse gas emissions, this region of the Alps could experience contrasting climate futures, warming from 0.5 (in RCP 2.6, solid line) to 2.3 (in RCP 4.5, dashed line) and potentially even 4.8°C (in RCP 8.5, dotted line) by the end of the century relative to the experimental period (2017–2020). (b) Increases in temperature under the contrasting climate change scenarios led to non‐monotonic changes in the diversity of the simulated alpine community. (c) Principal coordinates ordination based on Euclidean distances showing the trajectory of simulated alpine communities under the different climate change scenarios between years 2017 and 2098 (each point corresponds to a year in one scenario, in 3‐year time steps to reduce clutter). The grey arrows are vectors of taxa for which ordination axes explained more than 90% of variation in abundance through time. Panels (b) and (c) show the median of 20 simulated trajectories. The 5% and 95% quantiles are shown in panel (b) and ten runs of the simulations generating panel (c) are shown Figure S5.4.

Under all climate change scenarios, mean summer temperature at the 2000 m site (near the elevation of the original community) increases only modestly prior to 2040. Thereafter, temperature stabilises in RCP 2.6, warming continues through the century in RCP 4.5 and even more rapidly in RCP 8.5 (Figure 2a). These different rates and magnitudes of warming resulted in contrasting trajectories of species diversity (Figure 2b). After an initial decline during the first half of the century in all scenarios, diversity stabilised in RCP 2.6 but rebounded to nearly its initial levels in RCP 4.5 and 8.5. Since our simulations did not include immigration, this diversity rebound was driven solely by increasing evenness of species abundances (mainly due to the mid‐century rise of *Alchemilla xanthochlora*). The rebound in RCP 8.5 was followed by a precipitous decline towards the end of the century.

Species compositional change followed a common trajectory for all three warming scenarios, with the scenarios differing in how far they moved along that trajectory (Figure 2c). Most (86.3%) of the variation in community structure across time was captured by the first axis of the Principal Coordinates Analysis. This axis was positively correlated with the abundance of *Alchemilla xanthochlora*, which increased with warming (Figure 2c). Thus, the severity of warming scenarios dictated how far simulated communities moved towards the positive side of the first ordination axis. In contrast, movement along the second ordination axis was not monotonic. Instead, under modest to moderate warming, simulated communities moved towards more positive values along the second ordination axis, but this trend eventually reversed under the severe warming of the second half of the century in scenario RCP 8.5 (Figure 2c, to better visualise the trajectories through time, see interactive results for one simulation in Supporting Material 6).

**FIGURE 3:**
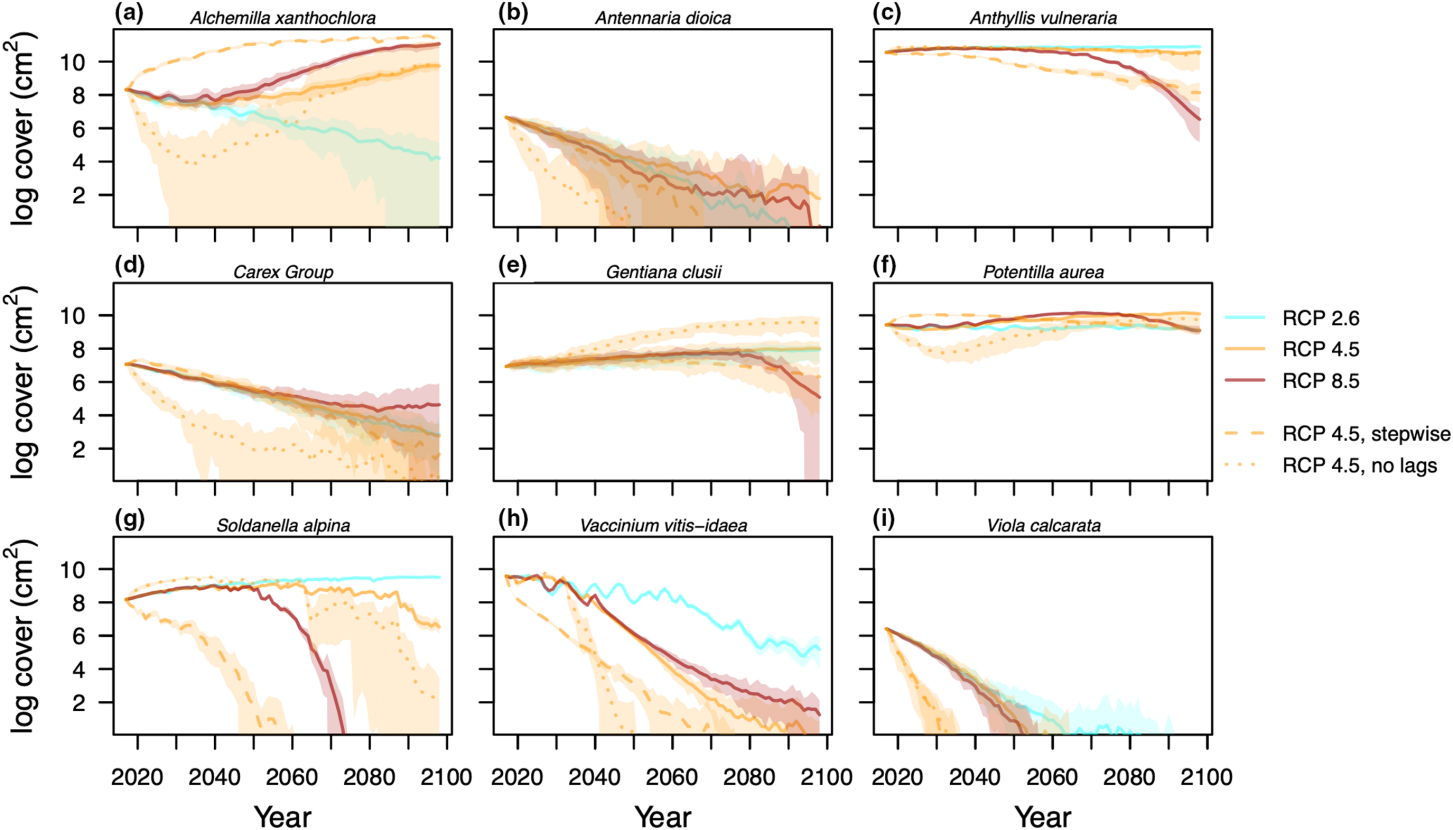
Cover dynamics of alpine plant species under contrasting climate change scenarios RCP 2.6 (cyan), RCP 4.5 (orange) and RCP 8.5 (red). Lines show the natural logarithm of the sum of the cover of all individuals in each year of the climate change simulations. The trajectories of summer temperatures in the region under these scenarios are shown in Figure 2a. For scenario RCP 4.5, dotted lines show dynamics with a reduced influence of ecological lags, while dashed lines show dynamics following a stepwise change in climate to conditions expected by the end of the century (mean conditions between 2088 and 2098). Lines show the median of 20 simulated trajectories, and shading indicates the 5% and 95% quantiles.

A closer look at the dynamics of different taxa sheds light on the nature of these contrasting community trajectories (Figure 3). First, we note that our models did not predict stable community dynamics under current climatic conditions. Some taxa went extinct during the pre‐climate change burn‐in period (*Leontodon* Group), or soon after (*Androsace chamejasme*), while others increased sharply (namely *Anthyllis vulneraria*, *Soldanella alpina* and *Vaccinium vitis‐idaea*, Figure S1.5). After the equilibrating burn‐in period, moderate warming till mid‐century (RCP 2.6) led to minor changes in community structure, namely the extinction of *Viola calarata* by mid‐century, and a gradual decline in the cover of *Vaccinium* and *Alchemilla xanthochlora*. In contrast, warming unabated throughout the century (RCP 4.5) began with similar trends as RCP 2.6, but eventually reversed *Alchemilla*'s declining trend, and accelerated *Vaccinium*'s. More severe warming (RCP 8.5) led to a faster rise in *Alchemilla* after 2050 and to the synchronous sharp decline of the initially dominant legume *Anthyllis vulneraria*.

### Simulated responses to gradual and abrupt climate change

**FIGURE 4:**
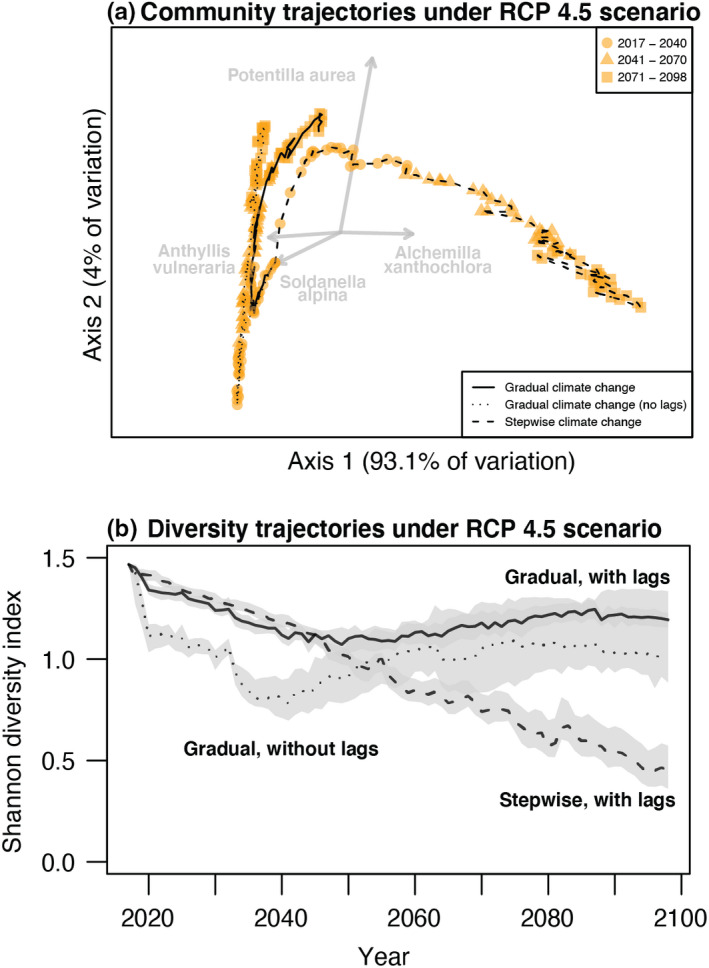
Alpine community responses to gradual versus stepwise climate change. (a) Principal coordinates analysis ordination based on Euclidean distances showing the trajectory of simulated alpine communities under stepwise (dashed line) versus gradual climate change (RCP 4.5), with either full (solid line) or reduced (dotted line) influence of ecological lags. Each point corresponds to a year. The grey arrows are vectors of taxa for which ordination axes explained more than 90% of variation in abundance through time. (b) Trajectories of Shannon diversity based on taxa's total cover in simulated communities. Panels show the median of 20 simulated trajectories. Ten runs of the simulations generating panel A are shown in Figure S5.4, and 5% and 95% quantiles are shown with shading in panel (b).

When we projected community dynamics following the common experimental approach of a stepwise change to RCP 4.5 end‐of‐century climate, the community changed more rapidly than predicted under a gradual approach to the same climate endpoint (Figure 4). More surprisingly, these two trajectories to RCP 4.5 end‐of‐century climate also differed in their species compositional longer‐term outcome. Stepwise change led to a rapid rise in the cover of *Alchemilla*, and to a decline in the cover of taxa which would have otherwise remained abundant throughout the century, such as *Anthyllis vulneraria*, *Potentilla aurea* and *Soldanella alpina* (cf. solid and dashed orange lines, Figure 3). This also resulted in a slower, but constant, diversity decline following stepwise warming, as opposed to a faster initial decline followed by a rebound under gradual warming (Figure 4b).

### Effect of ecological lags

Ecological lags had a profound influence on not only the timescale but also the compositional longer‐term outcome of community responses to climate change (Figure 4). Namely, during the first (and cooler) decade of simulated community dynamics without ecological lags, the relatively thermophilic *Alchemilla* went extinct in most simulation runs (Figure 3a). This prevented its eventual rise to dominance in these runs, and is in contrast to what is seen when lags enabled *Alchemilla* to persist through the initial cooler period and into warmer conditions later in the century. Reduced lags also precipitated the extinction of *Viola*, *Antennaria* and *Carex* Group, although this had a much smaller influence on future dynamics (Figure 3). Later in the century, as temperatures continued to rise, removing lags led to a much faster decline in the cover of *Vaccinium* and *Soldanella*, species intolerant of the warmer climate (Figure 3). These amplified responses of species to climate in the absence of lags led to a sharper decline in diversity during the first two decades of climate change simulations, which never completely recovered (Figure 4b).

## DISCUSSION

Transplanting alpine plant communities to lower elevations, where environmental conditions resemble those expected in the alpine zone in the coming decades, led to large, nonlinear changes in community structure and diversity. Despite a tendency for errors to propagate across time steps in projections of community dynamics (Adler et al., 2010), our simulations based on demographic models reproduced the trajectories of change of a subset of the community—namely the dominant forbs—observed during the experiment (Figure 1). Although this subset might not be representative of the entire community, our experimentally parameterised models offer an unprecedented tool to explore the trajectory of alpine species' dynamics under future climate change scenarios. Overall, our simulations demonstrate that (1) the fate of alpine community diversity and composition is tightly linked to the severity of climate change, (2) responses to abrupt climate manipulations miss transient community trajectories expected under gradual climate change, with lasting consequences and (3) ecological lags govern the pace, trajectory and even the long‐term outcome of community responses to climate change.

Severe warming during the second half of the 21st century under worst‐case scenarios of climate change (e.g. RCP 8.5) could profoundly reshape plant diversity in mountains (Engler et al., 2011). According to climate niche modelling, such warming could cause nearly half of European mountain plant species to lose 80% of their range by 2100 (Engler et al., 2011). Consistent with these predictions, our projections of local community dynamics under RCP 8.5 revealed a sharp loss of local species diversity by the end of the century (Figure 2b), with most taxa either extinct or declining towards extinction (Figure 3, Dullinger et al., 2012). However, while a recent space‐for‐time substitution study found that most species in high latitude communities likely face dangerous warming around 2050 (Trisos et al., 2020), we found that the timing of population decline varied by multiple decades across taxa in this alpine community (Figure 3). Some of this variation could emerge from taxon‐specific lags in demographic and ecological processes (Alexander et al., 2018).

While stepwise climate manipulations can reveal how species' demography and ecological interactions respond to future conditions, our simulations show that they are prone to mischaracterising the trajectory of non‐monotonic population dynamics (Figure 3 and Figure S.2). How prone, however, depends on the mechanism behind the non‐monotonic dynamics. If a species' demographic performance peaks at intermediate levels of warming, gradual warming may lead to an initial increase in the species' abundance followed by a decline once the optimal temperature is surpassed, whereas stepwise warming beyond the optimal temperature would lead to an immediate decline (e.g. *Soldanella alpina* in our simulations, Figure 3g and Figure S5.3). Given that the growth of alpine species often increases with warming only until water becomes limiting (Dolezal et al., 2020), this might be a common scenario in experiments imposing severe stepwise warming. Instead, if the initial population trend is reversed due to lagged changes in the abundance of competitors (Suttle et al., 2007), stepwise manipulations could detect the non‐monotonic trajectory, as long as dynamics are followed for enough time. In our simulations, *Potentilla aurea* exemplified this situation: its cover increased immediately after stepwise change, but later decreased as *Alchemilla*'s abundance increased (Figure 3). Nonetheless, our simulations show that community dynamics following an abrupt change in climate can take multiple decades to stabilise (Figure 4). Thus, such experiments may inform the direction of community responses to climate change, not their long‐term equilibria, at least in communities where ecological lags are strong (Duncan, 2021).

We have shown that beyond delaying responses to climate change, ecological lags can alter the trajectory and, under special circumstances, even the long‐term outcome of responses. In our simulations, lags buffered the decline of *Alchemilla xanthochlora* during the first decades of relatively cool climate, preventing its likely extinction (Figure 3a) and thereby completely altering future community trajectories and their longer‐term outcome (Figure 4a). While this result might stem partly from an underestimate of *Alchemilla*'s performance under cold climate (Figure S1.4), demographic lags enable populations persistence through unfavourable periods, with important implications for competitive outcomes (Adler et al., 2010; Compagnoni et al., 2021; Warner & Chesson, 1985). Lags also delayed the extinction of other taxa by multiple decades (Figure 3), thereby facilitating an extinction debt by mid‐century (Dullinger et al., 2012).

Our results come with several caveats. First, by excluding cows from the experimental sites, we altered the normal grazing regime. Thus, our data reflect the dynamics of alpine plant communities in a warmer future, albeit one without cows. Still, because cows were excluded at all sites, and climate differed across these sites, our models effectively isolate climate effects on species' demography and interactions. Second, our approach depends on projecting an individual‐based model forward in time. And though the model does a reasonable job at predicting 3 years of observed change in the community, how well it does over multiple decades is uncertain due to the likely propagation of errors through time. Since the models are based on data from a short experiment, they are unlikely to capture the full range of demographic responses to climate. For example weather peculiarities during the 4 years of study could hamper generality. Similarly, responses to warming that take multiple years to manifest, such as enhanced decomposition rates (Stuble et al., 2019) or lagged acclimation (Mulder et al., 2017), could be important, but missed determinants of future community dynamics. Third, although we modelled future dynamics as a function of average summer weather conditions, an increasing frequency and severity of droughts and other extreme weather events (Gobiet et al., 2014) and could lead to different community responses from those forecasted here. Finally, we could only model the dynamics of a subset of the species in the community, which might not be representative of the full spectrum of functional variation of alpine species. Overall, these caveats emphasise that our projections should not be interpreted as exact predictions of the future alpine community. Instead, our results inform a general understanding of the determinants of community trajectories under climate change, including the rates of warming, plant demographic responses, and lagged ecological responses.

We conclude by highlighting important questions for future research. First, phenomenological models like the ones we used to describe the climate‐dependency of species' demography and interactions do not reveal the mechanisms underlying competition. Thus, alternative modelling approaches based on eco‐physiology could provide complementary insights (e.g. Trugman et al., 2018). Second, this study focused on community dynamics driven by the changing abundances of the current resident species of the alpine. Future research should compare the timescale of the arrival of novel low‐elevation competitors (Alexander et al., 2015) to the timescale of changing dynamics among the resident species. The fast community change we observed in our experiment and in our simulations, despite the influence of ecological lags, suggests that alpine plant communities may change substantially well before low‐elevation competitors arrive. Therefore, an important future question is how the types of changes to the alpine communities forecast here will influence the arrival of novel competitors from lower elevation.

## AUTHOR CONTRIBUTIONS

SB and JML conceptualised the research, with input from all co‐authors. SB, MJM, JMA, LP and JML helped design and set up the experiment. MJM lead the development of the methods to survey vegetation with input from SB and JML. SB and MJM collected and processed the data. JIL quantified uncertainty due to demographic stochasticity in the simulations and aided in figure design. SB analysed the data and lead the writing of the manuscript, with input from all co‐authors.

## Supporting information


Appendix S1

Appendix S2

Appendix S3

Appendix S4

Appendix S5

## Data Availability

All data needed to reproduce our results are available from the Zendo repository (https://doi.org/10.5281/zenodo.6391677). All code is available in Supporting Information S2 and https://github.com/blockecology/lagging‐meadows.
