# Supplementary material for: Ecological lags govern the pace and outcome of plant community responses to 21st‐century climate change
Source: Ecol Lett. 2022 Aug 26;25(10):2156–66. doi: 10.1111/ele.14087 (PMC9804264; doi:10.1111/ele.14087)
Supplement: Supplementary file 1 — Appendix S1 [file ELE-25-2156-s005.pdf]

SM1: Supplementary Methods

We forecast trajectories of community responses to climate change by using experiments to parameterize models of climate-dependent community dynamics. This was achieved by measuring the demography and interactions of entire plant communities exposed to a suite of environments representative of what these communities are likely to experience over the next decades of climate change (Fig. S1.1). With data on such responses, we parameterized statistical models describing species' demography and interactions as continuous functions of climate. The parameters of these statistical models were then used as building blocks of climate-dependent, dynamic models of competition. Coupled with simulations of 21st century climate change, these models were used to project trajectories of community change under different time lag assumptions, and quantify the realistic timescales of climate change impacts (Fig. S1.1C).

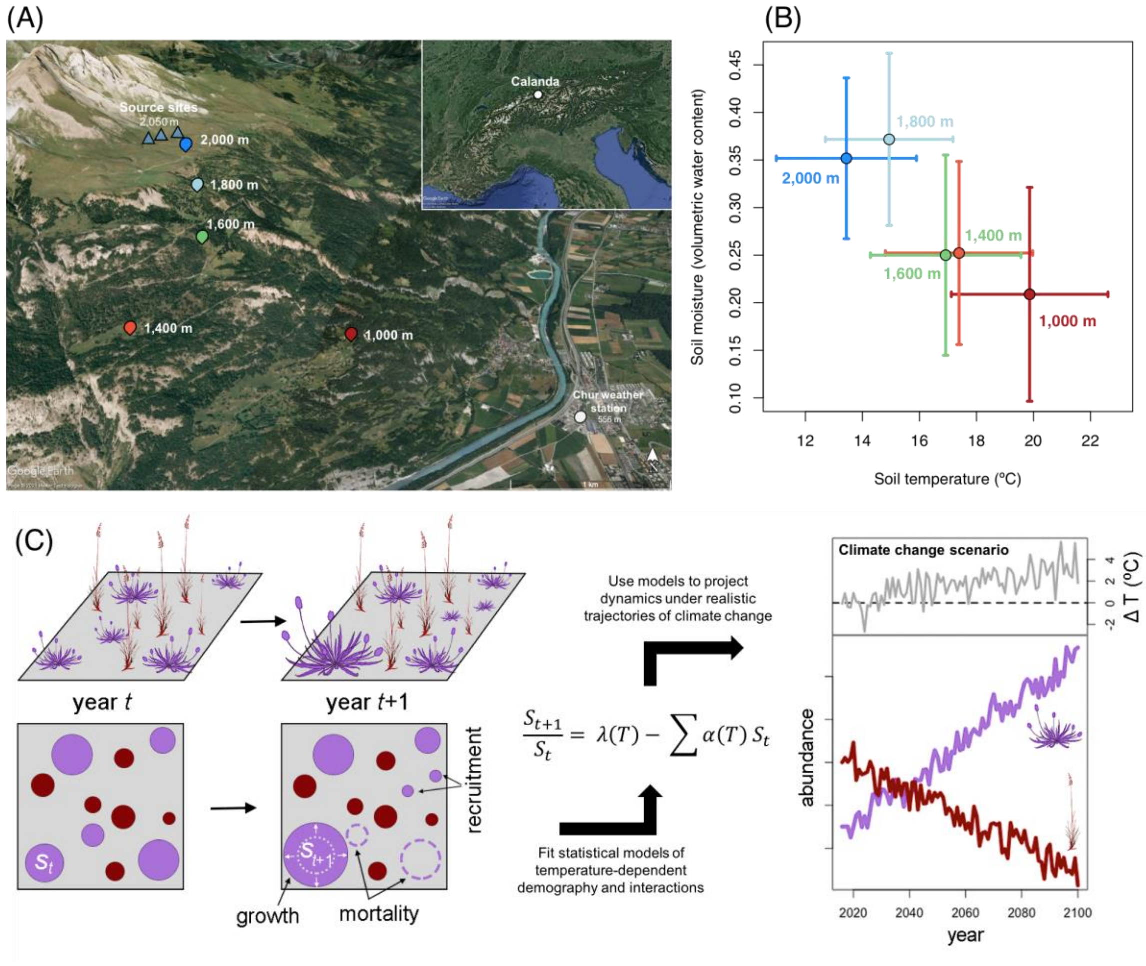

Figure S1.1. Approach to project trajectories of alpine community dynamics under climate change. (A) Google Earth imagery showing the location of the Calanda mountain in the eastern Swiss Alps (inset, map data: Google, Data SIO, NOAA, U.S. Navy, NGA, GEBCO; image: Landsat / Copernicus), as well as the location of source and transplantation sites within the mountain (map data: Google © 2021, Maxar Technologies). We transplanted replicate turfs from three source sites at 2050 m elevation to each of five sites at lower elevations (2000 m, 1800 m, 1600 m, 1400 m, 1000 m) encompassing a gradient of warmer and drier conditions. (B) Daily mean temperature and soil moisture (mean ± 1 SD) recorded at the sites during the summer months (June, July and August) of 2019 and 2020. (C) We mapped the transplanted communities yearly to estimate demographic rates. We then used statistical models to estimate the temperature-dependence of species' demography and interactions. Finally, we coupled these models with climate change simulations to project community dynamics over the 21st century.

1. Details about vegetation survey methodology

To monitor community dynamics and quantify plant demography under climate change in a way that allowed us to parameterize models, we made detailed surveys of the spatial distribution of canopy cover of all plant species in the transplanted turfs. To this end, soon after snowmelt on the spring after transplantation, we fixed four metallic grids of 0.5 x 0.5 m subdivided into 25 cells (NHBS Q2 Quadrat) covering each transplanted turf (Fig. S1.2). We used u-shaped nails to fix the grids to the turf, guaranteeing minimal movement throughout the years.

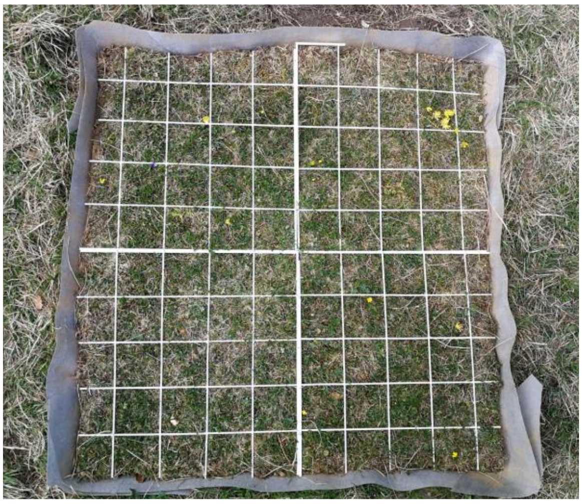

Figure S1.2 Grid system used to survey transplanted alpine communities. During surveys, we subdivided each grid cell (10 × 10 cm) into four quadrants (dotted lines) and estimated the canopy cover of all taxa

rooted in each. Because of their proximity to the margins, the outer grid cells (shaded area) were not surveyed.

Every summer for the next four years (2017 to 2020), we monitored the spatial distribution of species' covers in the transplanted turfs. We started surveys at the lowest site (1000 m) in mid-June and moved up the mountain over the summer in an attempt to map turfs at a similar stage of phenological development, close to the peak of the growing season at each site (Fig S1.3). Additional turfs and treatments unrelated to this study delayed surveys at the 1400 m site. From 2018 onwards, we used presence records from the previous year's survey to help relocate ramets during the surveys.

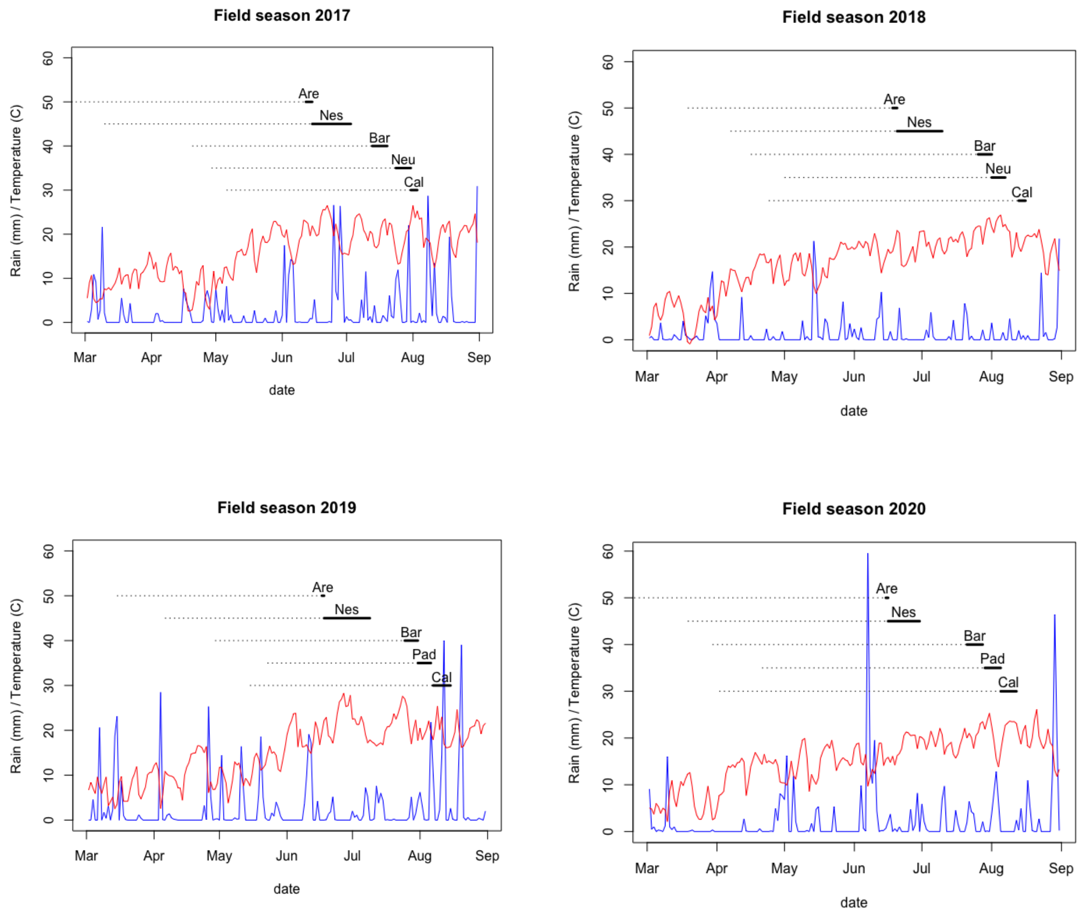

Figure S1.3 Timing of the surveys and weather in Chur during the growing season from 2017 to 2020. The dotted line indicates the snow free period at sites at different elevations ('Are' = 1000 m, 'Nes' = 1400 m, 'Bar' = 1600 m, 'Neu' and 'Pad' = 1800 m, 'Cal' = 2000 m), and the solid line shows the period of

vegetation surveys at each site. Blue lines show daily precipitation, and red lines daily mean temperature at nearby Chur weather station.

Giving the time constraints imposed by the survey's schedule, we were not able to identify all plants at the species level, and instead we had to clump some species into groups varying in taxonomic hierarchy and functional meaning (Table S1.1). For example, we only identified to genus level plants in the genera *Carex*, *Trifolium*, and *Luzula*; and we only distinguished among two kinds of grasses, those with needle-like leaves (referred as *"Festuca* group") and those with broad leaves (referred as "Grass group"). We also grouped mosses and lichens.

Table S1.1. Taxonomic groups higher than species level used in plant community surveys

**Group name**	**Description**	**Species potentially included**
Trifolium spp.	All species in the genus *Trifolium*	*Trifolium badium*, *T. pratense*, *T. repens*
Galium spp.	All species in the genus *Galium*	*Galium odoratum*
Thymus spp.	All species in the genus *Thymus*	*Thymus praecox*
Leontodon Group	All species in the genus *Leontodon* and other similar rosettes in the family Asteraceae	*Leontodon hispidus*, *Leontodon helveticu*, *Crepis aurea*
Aster-Erigeron Group	Species in the genera *Aster* and *Erigeron*	*Aster alpinus*, *Erigeron neglectus*
Gentiana Group	All the gentian species except *Gentiana clusii* and *Gentianella campestris*	
Carex Group	All species in the genus *Carex*	*Carex sempervirens*, *Carex ornithopoda*, *Carex montana*, *Carex verna*
Festuca Group	Grass species with needle-like leaves	*Festuca rubra*, *Nardus stricta*
Grass Group	Grass species with broad leaves	*Anthoxanthum odoratum*
Moss Group	All bryophytes	
Lichen Group	All lichens	

We subdivided each grid cell into four 5 x 5 cm quadrants, and surveyed the inner 256 quadrants of each transplanted turf (Fig S1.1). In each 25 cm^2 quadrant, we recorded all taxa and visually estimated their canopy covers using an ordinal scale composed of categories corresponding to fractions of the quadrant cover (Table S1.2). This helped make more consistent cover estimations. Between five and six botanically trained individual researchers participated in the surveys each field season (and 12 individuals in total from 2017 to 2020). The team spent time before and during each field season calibrating their cover estimations to minimize individual researcher biases.

Table S1.2. Cover categories used for vegetation mapping. The absolute and proportion areas are always upper bounds. For plants larger than the quadrant, we approximated cover to the nearest ¼ of quadrant area (e.g., 1.25, 1.50, 1.75 quadrants, etc.).

**Category**	**Fraction of quadrant area**	**Absolute area (cm^2)**
1	< 1/16	< 1.56
2	1/8	3.13
3	1/4	6.25
4	1/2	12.50
5	3/4	18.75
6	1	25.00

2. Modeling demographic rates as functions of climate and neighborhood crowding

We fit statistical models that quantified how a taxon's demographic rates (survival, growth, recruitment) from the plant community surveys related to climate and interactions with neighbors. Following previous work estimating species' interactions from vegetation maps (Adler et al 2010; Chu and Adler, 2015), we assumed that the crowding experienced by a focal ramet i at time t ($w_{i,t}$) depends on the sum of each neighbor k's cover ($u_{k,t}$) weighed by that neighbor's distance to the focal ramet ($d_{ik,t}$) according to a Gaussian interaction kernel:

$$w_{i,t} = \sum_k e^{-\delta\, d_{ik,t}^2} \cdot u_{k,t} \tag{1}$$

where δ determines how fast neighbor crowding declines with distance. Based on the small size of the ramets of all taxa, we used a value of $\delta = 0.02$, meaning that most crowding was exerted by neighbors within a 10-cm radius (fig. S2.1). To avoid artificially low crowding values near the edge of the surveyed area of the turfs, we fitted demographic models using only ramets in the inner 144 quadrants. In addition, to avoid overfitting the models, we pooled all heterospecific neighbors into a single crowding value w_j.

We used a logistic regression to model the probability of ramet survival (s) as a function of mean temperature during the previous summer (T_{t-1}), ramet size (u_i), and crowding from conspecific and heterospecific neighbors (w_i and w_j, respectively), as follows

$$logit\ (s) = \lambda^S(T_{t-1}) + b^S \cdot u + \alpha_{ii}^S(T_{t-1}) \cdot w_{i,t-1} + \alpha_{ij}^S(T_{t-1}) \cdot w_{j,t-1} \quad (2)$$

where $\lambda^S(T_{t-1})$ is the direct effect of temperature on survival, b is the effect of ramet size, and $\alpha_{ii}^S(T_{t-1})$ and $\alpha_{ij}^S(T_{t-1})$ are the temperature-dependent effects of interactions with conspecific and heterospecific neighbors, respectively. We assumed that λ^S was a sigmoidal function of T_{t-1}:

$$\lambda^S = \left(\frac{L}{(1 + \exp(-k(T_{t-1} - h)))}\right) + \theta^S \quad (3)$$

where L is the function's maximum, k its steepness, and h its inflection point. This functional form is consistent with temperature affecting ramet survival primarily through a thresholding mechanism, such as increased evapotranspiration leading to embolism risk (Choat *et al.* 2018), which can be important in alpine communities (Boeck *et al.* 2016). Since the temperature leading to high embolism risk likely depends on water availability, the inflection point h was a linear function of average soil moisture during the previous summer. Since $\lambda^S(T_{t-1})$ is on the log-odds scale but the minimum value of its first term is 0, we added the constant θ^S and set its value to -4 to guarantee that the survival probability of a mean-sized ramet in the absence of neighbors was low (i.e., 0.02) when T_{t-1} was far beyond the threshold represented by parameter h.

To account for the fact that the effects of neighbors on ramet survival may also depend on temperature, the interaction coefficients α_{ii} and α_{ij}, were linear functions of T_{t-1}, and could take both positive and negative values. Positive values of α_{ii} (implying net intraspecific facilitation) are particularly likely due to potential nutrient translocation between ramets connected underground (Roiloa *et al.* 2014; Liu *et al.* 2016; Guo *et al.* 2017).

We modeled ramet growth (g) as a normally distributed variable with a mean determined by a similar hierarchical function including temperature-dependent neighbor effects (α_{ii}^{G} and α_{ij}^{G}), an effect of ramet size (b^{G}), and a direct effect of temperature (λ^{G}):

$$g = \lambda^{G}(T_{L2S}) + b^{G} \cdot u - \alpha_{ii}^{G}(T_{L2S}) \cdot w_{i,t-1} - \alpha_{ij}^{G}(T_{L2S}) \cdot w_{j,t-1} \qquad (4)$$

Since growth in perennial alpine plants may depend on resources stored during previous growing seasons (Kleijn *et al.* 2005), we used the mean temperature during the previous two summers (T_{L2S}) as the climatic driver in the growth models. Thus, to model growth during the first transition, from 2017 to 2018, we used the average of the summer 2017 temperature at the different sites and the summer 2016 temperature at the 2000-m site of origin for all the turfs.

Given that we used an ordinal scale to record each taxa's cover (section 2.1.3), the model estimated ramet growth (g) as the log ratio of two estimated parameters representing the ramet's true, unknown sizes at times *t* and *t*+1 (see details in section 2.2 of Supporting Information 2). An analysis with simulated data showed that accounting for uncertainty in recorded covers enabled the model to accurately estimate the effect of temperature on demography and species' interactions (Supporting Information 3).

We assumed that, in the absence of neighbors, ramet growth was a Gaussian function of temperature:

$$\lambda^{G} = r_{max} \cdot \exp\left(\frac{-\left(T_{L2S} - T_{opt}\right)^{2}}{2\sigma^{2}}\right) \qquad (5)$$

where r_{max} is the maximum growth rate, T_{opt} is the optimal temperature for growth, and σ determines temperature tolerance.

Interaction coefficients in the growth model were exponential functions of temperature:

$$\alpha_{ii}^{G} = e^{(a+bT_{L2S})} \quad (6)$$

With an equivalent function for α_{ij}^{G}. This functional form implies that interaction coefficients in the growth model were always competitive (see eq. 4).

Finally, we used a logistic regression to model the recruitment probability (r) in each quadrant previously unoccupied by a given taxon, according to:

$$logit(r) = \alpha_{ii}^{R}(T_{t-1}) \cdot w_{i,t-1} - \alpha_{ij}^{R}(T_{t-1}) \cdot w_{j,t-1} + \theta^{R} \quad (7)$$

As in the growth model, interaction coefficients in eq. 7 were exponential functions of mean temperature during the previous summer (T_{t-1}), equivalent to eq. 6. This equation, with interaction coefficients constrained to be positive, implies that recruitment is a positive function of intraspecific neighbors ($w_{i,t-1}$, as the source of the recruiting propagules), while a negative function of crowding by heterospecifics ($w_{j,t-1}$) (e.g., via competition for space or resources). We set the value of $\theta^{R} = -5$ to ensure a low probability of recruitment in quadrants with no conspecifics (i.e., propagule sources) in the vicinity.

Ramets that were declared dead in the vicinity (< 5 cm) of areas of the turf affected by vole activity during winter and spring were excluded from the data used to fit demographic models. Also, approximately one quarter of each turf at the 2000-m and 1400-m sites was excluded due to physical disturbance by other measurements made on those turfs.

3. Details about model sampling problems and excluding species from downstream analyses

We attempted to fit the models of ramet survival, growth, and recruitment described above for all the flowering plant taxa with at least 1,000 observations across all years and sites. For seven of these taxa, the Markov chains of one of the demographic models did not converge, so we excluded them from downstream analyses. For 12 other taxa, divergent transitions arose during sampling or sampling was otherwise inefficient (i.e., low effective sample size). During our simulation tests (Supplementary Material 3), we observed that divergent transitions could sometimes arise even when models were able to adequately estimate true parameter values. Thus, when divergent transitions arose during sampling, we tested whether models were able to recover "true" parameter values from data simulated assuming that the mean of the parameters in the original sample *was* the true value (details in Supplementary Material 4). Three models (survival of *Achillea millefolium*, growth of *Homogyne alpina* and of *Salix retusa*) did not pass this *a posteriori* simulation test and were thus excluded from downstream analyses.

Table S1.3. Taxa for which we fitted demographic models, shown in order of decreasing initial cover at the 2000-m site. The 11 taxa for which demographic models successfully reproduced dynamics observed in the experiments, and were thus included in simulations of future climate change, are shown in bold.

**Taxon**	**Family**	**% cover in 2000-m turfs in 2017**	**Number of observations across years and sites**	**Number observations used to train models**		
				**S**	**G**	**R**
***Leontodon* Group**	Asteraceae	8.62	11,128	3,960	2,280	10,660
Alchemilla xanthochlora	Rosaceae	8.03	13,368	4,485	3,797	11,626
Anthyllis vulneraria	Fabaceae	7.93	9,015	3,074	2,169	13,159
Potentilla aurea	Rosaceae	6.87	20,816	7,118	5,740	8,620
Festuca Group	Poaceae	5.00	43,166	14,761	13,482	1,468
Vaccinium vitis-idaea	Ericaceae	3.01	9,294	3,481	2,568	8,674
Plantago atrata	Plantaginaceae	2.97	12,861	2,982	2,172	13,142
Viola calcarata	Violaceae	2.59	12,861	4,684	2,977	9,700
Ranunculus montanus	Ranunculaceae	2.25	11,746	3,795	2,541	11,701
Hieracium lactucella	Asteraceae	2.23	2,638	979	602	11,265
***Carex* Group**	Cyperaceae	2.01	15,896	5,306	4,497	10,839
Androsace chamejasme	Primulaceae	1.95	7,281	2,727	1,704	9,525
Ligusticum mutellina	Apiaceae	1.51	4,979	1,689	1,020	7,711
Pulsatilla vernalis	Ranunculaceae	1.49	1,558	667	455	11,917
Polygonum viviparum	Polygonaceae	1.28	3,779	1,274	794	13,485
Phyteuma orbiculare	Campanulaceae	1.03	6,069	1,817	1,200	12,268
Antennaria dioica	Asteraceae	0.92	2,687	955	572	11,216
Myosotis alpestris	Boraginaceae	0.75	3,217	1,017	637	11,101
Potentilla crantzii	Rosaceae	0.70	2,737	953	726	14,191
Lotus alpinus	Fabaceae	0.65	1,995	710	536	10,487
Gentiana clusii	Gentianaceae	0.63	1,634	584	355	12,401
Luzula spp.	Juncaceae	0.56	3,905	1,265	778	14,804
Soldanella alpina	Primulaceae	0.30	1,042	260	185	5,931
Ranunculus acris	Ranunculaceae	0.20	2,599	819	469	11,059
Leucanthemum adustum	Asteraceae	0.18	1,620	478	395	6,674

4. Comparing predicted versus observed species responses

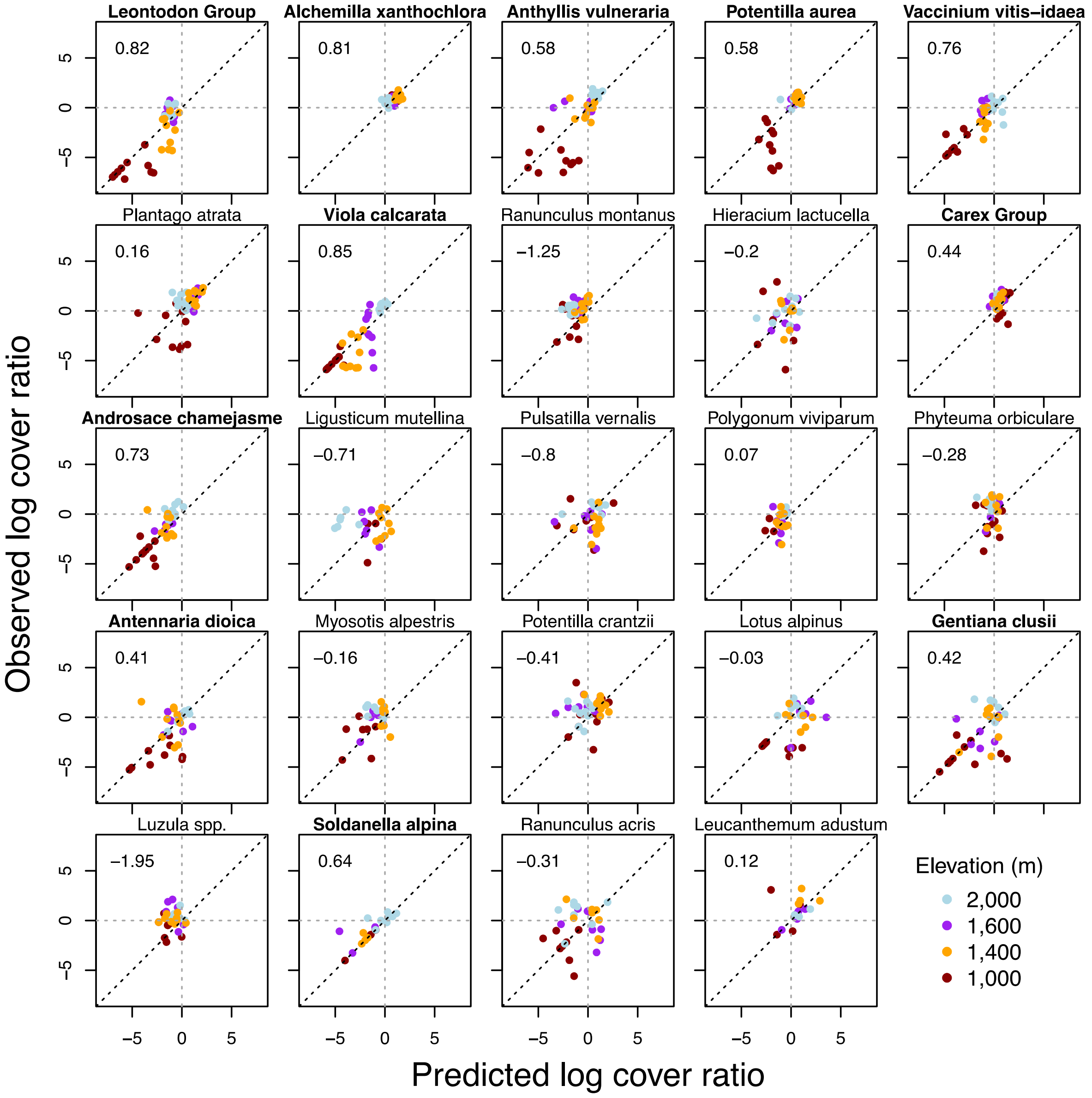

Figure S1.4 Predicted versus observed log ratios of final to initial cover of different species in turfs transplanted to different elevations. The number shown in the upper left corner of each panel is equal to 1 minus the ratio of squared residuals to squared observations; that is, positive values indicate that the predicted responses explain more variation than a null model expecting no changes in species cover. This was the case for 11 taxa, shown in bold, which were included in simulations of future climate change and account for more than half (44 %) of the cover across all transplanted turfs. Turfs from the 1800-m site were excluded due to their later transplantation.

5. Demographic models without effects of interactions with neighbors

To assess the degree to which including interactions with neighbors in the demographic models improved their predictive ability, we compared the full models described in the main text (equations 2, 4 and 7) with equivalent models without biotic interactions. In these purely 'abiotic' models, survival probability (s) was:

$$logit\ (s) = \lambda^S(T_{LS}) + b^S \cdot u + \theta^S \tag{S1}$$

where $\lambda^S(T_{LS})$ is the direct effect of temperature (given by equation 3 in the main text), b is the effect of ramet size (u), and θ^S is a constant equal to -4. In turn, growth was:

$$g = \lambda^G(T_{L2S}) + b^G \cdot ln(u) \tag{S2}$$

where, again, λ^G is the direct effect of temperature on growth (given by equation 5 in main text), and b^G is the effect of ramet size (u). Finally, recruitment probability in an unoccupied quadrant q (r_q) was:

$$logit(r_q) = \alpha_{ii}^R(T_{LS}) \cdot w_i + \theta^R \tag{S3}$$

where $\alpha_{ii}^R(T_{LS})$ is the effect of conspecific crowding w_i, and θ^R is a fixed intercept equal to -5 to ensure the probability of recruitment is low (0.007) when there are no conspecifics (i.e., propagule sources) in the neighborhood of the quadrant.

We then compared models with and without species interactions using the widely applicable information criterion (WAIC, McElreath 2020). With few exceptions, survival and growth models including species interactions were superior to equivalent abiotic-only models. In contrast, for most taxa, recruitment models with and without interspecific competition had similar WAIC values. For consistency, however, and to be able to explore the role of interaction lags in shaping trajectories of community dynamics, we simulated community dynamics under future climate change with models including biotic interactions in all demographic transitions.

5.1 Survival models

Leontodon Group

Model	WAIC	SE	dWAIC	dSE	pWAIC	weight
Competition	4723.6	46.58	0.0	NA	6.7	1
Abiotic	4843.0	44.28	119.4	18.02	6.8	0

Alchemilla xanthochlora

Model	WAIC	SE	dWAIC	dSE	pWAIC	weight
Competition	3474.9	82.62	0.0	NA	8.2	1
Abiotic	3487.5	82.55	12.6	9.19	4.1	0

Anthyllis vulneraria

Model	WAIC	SE	dWAIC	dSE	pWAIC	weight
Competition	2994.6	60.85	0.0	NA	9.1	1
Abiotic	3013.8	59.83	19.2	10.45	4.7	0

Potentilla aurea

Model	WAIC	SE	dWAIC	dSE	pWAIC	weight
Competition	4639.1	107.82	0.0	NA	8.3	1
Abiotic	4688.9	107.85	49.9	14.63	4.9	0

Vaccinium vitis-idaea

Model	WAIC	SE	dWAIC	dSE	pWAIC	weight
Competition	3190.6	65.28	0.0	NA	8.7	1
Abiotic	3253.1	65.14	62.5	17.57	4.4	0

Plantago atrata

Model	WAIC	SE	dWAIC	dSE	pWAIC	weight
Competition	3034.6	59.71	0.0	NA	9.0	1
Abiotic	3049.8	58.97	15.2	8.33	5.3	0

Viola calcarata

Model	WAIC	SE	dWAIC	dSE	pWAIC	weight
Competition	4400.8	76.11	0.0	NA	9.1	1
Abiotic	4270.3	74.17	93.6	19.75	5.1	0

Hieracium lactucella

Model	WAIC	SE	dWAIC	dSE	pWAIC	weight
Competition	1149.3	25.43	0.0	NA	6.6	0.99
Abiotic	1158.9	25.74	9.6	7.87	2.9	0.01

Carex Group

Model	WAIC	SE	dWAIC	dSE	pWAIC	weight
Competition	4232.0	90.01	0.0	NA	9.4	1
Abiotic	4270.3	89.79	38.3	13.05	5.1	0

Androsace chamaejasme

Model	WAIC	SE	dWAIC	dSE	pWAIC	weight
Competition	3223.0	42.93	0.0	NA	8.5	1
Abiotic	3265.4	40.77	42.4	13.86	4.4	0

Antennaria dioica

Model	WAIC	SE	dWAIC	dSE	pWAIC	weight
Competition	1047.4	30.40	0.0	NA	8.6	1
Abiotic	1106.0	27.32	58.6	16.41	4.4	0

Myosotis alpestris

Model	WAIC	SE	dWAIC	dSE	pWAIC	weight
Competition	1123.7	28.89	0.0	NA	6.7	0.83
Abiotic	1126.9	28.24	3.2	6.65	3.1	0.17

Lotus alpinus

Model	WAIC	SE	dWAIC	dSE	pWAIC	weight
Competition	661.7	30.98	0.0	NA	7.8	1
Abiotic	683.5	30.66	21.9	10.46	5.6	0

Gentiana clusii

Model	WAIC	SE	dWAIC	dSE	pWAIC	weight
Competition	604.3	24.94	0.0	NA	7.9	0.96
Abiotic	610.8	24.68	6.5	6.8	4.4	0.04

Ranunculus acris

Model	WAIC	SE	dWAIC	dSE	pWAIC	weight
Abiotic	888.7	27.52	0.0	NA	3.7	1
Interactions	936.0	27.83	47.3	16.25	36.1	0

5.2 Growth models

Potentilla aurea

Model	WAIC	SE	dWAIC	dSE	pWAIC	weight
Competition	13810.2	100.46	0.0	NA	298.8	1
Abiotic	13935	98.21	125.5	27.53	236.3	0

Alchemilla xanthochlora

Model	WAIC	SE	dWAIC	dSE	pWAIC	weight
Competition	9608.0	104.24	0.0	NA	139.1	1
Abiotic	9665.8	102.24	57.9	18.19	133.7	0

Anthyllis vulneraria

Model	WAIC	SE	dWAIC	dSE	pWAIC	weight
Competition	5765.1	66.71	0.0	NA	70.5	0.92
Abiotic	5770.1	66.59	5	9.98	68.4	0.08

Vaccinium vitis-idaea

Model	WAIC	SE	dWAIC	dSE	pWAIC	weight
Competition	3190.6	65.28	0.0	NA	8.7	1
Abiotic	3253.1	65.14	62.5	17.57	4.4	0

Plantago atrata

Model	WAIC	SE	dWAIC	dSE	pWAIC	weight

Competition	5873.9	61.58	0.0	NA	79.6	0.75
Abiotic	5876.2	61.65	2.3	8.46	75.4	0.25

Viola calcarata

**Model**	**WAIC**	**SE**	**dWAIC**	**dSE**	**pWAIC**	**weight**
Competition	4400.8	76.11	0.0	NA	9.1	1
Abiotic	4270.3	74.17	93.6	19.75	5.1	0

Hieracium lactucella

**Model**	**WAIC**	**SE**	**dWAIC**	**dSE**	**pWAIC**	**weight**
Abiotic	1430.5	32.27	0.0	NA	30.8	0.99
Competition	1439.5	32.95	9	5.86	31.8	0.01

Carex Group

**Model**	**WAIC**	**SE**	**dWAIC**	**dSE**	**pWAIC**	**weight**
Competition	4232.0	90.01	0.0	NA	9.4	1
Abiotic	4270.3	89.79	38.3	13.05	5.1	0

Androsace chamaejasme

**Model**	**WAIC**	**SE**	**dWAIC**	**dSE**	**pWAIC**	**weight**
Competition	3223.0	42.93	0.0	NA	8.5	1
Abiotic	3265.4	40.77	42.4	13.86	4.4	0

Antennaria dioica

**Model**	**WAIC**	**SE**	**dWAIC**	**dSE**	**pWAIC**	**weight**
Competition	1047.4	30.40	0.0	NA	8.6	1
Abiotic	1106.0	27.32	58.6	16.41	4.4	0

Myosotis alpestris

**Model**	**WAIC**	**SE**	**dWAIC**	**dSE**	**pWAIC**	**weight**
Competition	1356.5	38.41	0.0	NA	46.2	1
Abiotic	1389.8	39.61	33.3	11.26	58.5	0

Lotus alpinus

**Model**	**WAIC**	**SE**	**dWAIC**	**dSE**	**pWAIC**	**weight**
Competition	1300.9	31.42	0.0	NA	31.3	1
Abiotic	1340.4	31.76	39.5	13.17	27.2	0

Gentiana clusii

**Model**	**WAIC**	**SE**	**dWAIC**	**dSE**	**pWAIC**	**weight**
Abiotic	793.5	27.72	0.0	NA	23.5	0.54
Competition	793.8	27.63	0.3	5.57	25.0	0.46

Ranunculus acris

**Model**	**WAIC**	**SE**	**dWAIC**	**dSE**	**pWAIC**	**weight**
Competition	1196.5	32.92	0.0	NA	24.6	1
Abiotic	1209.7	31.81	13.2	7.89	22.2	0

5.3 Recruitment models

Leontodon Group

Model	WAIC	SE	dWAIC	dSE	pWAIC	weight
Abiotic	3862.3	175.65	0.0	NA	2.7	0.86
Competition	3866.0	176.02	3.7	0.53	2.7	0.14

Alchemilla xanthochlora

Model	WAIC	SE	dWAIC	dSE	pWAIC	weight
Abiotic	4728.0	177.70	0.0	NA	2.9	0.9
Competition	4732.5	178.01	4.4	0.36	2.9	0.1

Anthyllis vulneraria

Model	WAIC	SE	dWAIC	dSE	pWAIC	weight
Abiotic	4657.0	175.61	0.0	NA	3.3	0.89
Competition	4661.2	175.94	4.2	0.41	3.4	0.11

Potentilla aurea

Model	WAIC	SE	dWAIC	dSE	pWAIC	Weight
Abiotic	6551.9	198.93	0.0	NA	3.4	0.92
Competition	6556.7	199.16	4.8	0.27	3.4	0.08

Vaccinium vitis-idaea

Model	WAIC	SE	dWAIC	dSE	pWAIC	weight
Abiotic	4373.8	170.68	0.0	NA	3.7	0.91
Competition	4378.5	170.94	4.7	0.32	3.8	0.09

Plantago atrata

Model	WAIC	SE	dWAIC	dSE	pWAIC	weight
Abiotic	4221.2	182.91	0.0	NA	2.6	0.9
Competition	4225.6	183.25	4.5	0.37	2.6	0.1

Viola calcarata

Model	WAIC	SE	dWAIC	dSE	pWAIC	weight
Abiotic	3799.5	162.30	0.0	NA	3.1	0.65
Competition	3800.7	163.04	1.3	2.74	4.2	0.35

Hieracium lactucella

Model	WAIC	SE	dWAIC	dSE	pWAIC	weight
Abiotic	1546.2	102.11	0.0	NA	3.5	0.8
Competition	1549.0	103.10	2.8	1.1	3.6	0.2

Carex Group

Model	WAIC	SE	dWAIC	dSE	pWAIC	weight
Abiotic	9724.1	252.24	0.0	NA	4.8	0.93
Competition	9729.3	252.43	5.2	0.21	4.8	0.07

Androsace chamaejasme

Model	WAIC	SE	dWAIC	dSE	pWAIC	weight
Abiotic	2386.2	126.28	0.0	NA	2.1	0.66
Competition	2387.5	127.23	1.4	1.68	2.7	0.34

Antennaria dioica

Model	WAIC	SE	dWAIC	dSE	pWAIC	weight
Competition	1133.6	91.55	0.0	NA	4.2	1
Abiotic	1154.1	88.15	20.5	7.03	2.2	0

Myosotis alpestris

Model	WAIC	SE	dWAIC	dSE	pWAIC	weight
Abiotic	3209.7	160.53	0.0	NA	2.2	0.89
Competition	3213.9	160.88	4.1	0.41	2.2	0.11

Lotus alpinus

Model	WAIC	SE	dWAIC	dSE	pWAIC	weight
Competition	1446.6	98.44	0.0	NA	4.6	0.76
Abiotic	1448.9	96.26	2.3	3.83	3.0	0.24

Gentiana clusii

Model	WAIC	SE	dWAIC	dSE	pWAIC	weight
Competition	1095.4	93.66	0.0	NA	3.2	1
Abiotic	1108.1	89.65	12.7	5.99	1.8	0

Ranunculus acris

Model	WAIC	SE	dWAIC	dSE	pWAIC	weight
Abiotic	2196.7	133.37	0.0	NA	2.3	0.88
Competition	2200.6	133.88	3.9	0.54	2.3	0.12

6. Pre-climate change burn-in simulation

To disentangle the effects of climate change on community dynamics from the effects of experimental artefacts and poor model fit, we let community dynamics ran under the current climatic conditions (defined as the mean of conditions from 2017 to 2020 across RCP scenarios 2.6, 4.5, and 8.5). We considered that the community had reached a quasi-equilibrium once the absolute value of the relative growth rate of all taxa was < 0.05 or the absolute value of total cover change < 320 cm^2 (equivalent to 0.5 % of turf total area), between two subsequent years. It took 24 years of dynamics to reach this quasi-equilibrium, during which *Anthyllis vulneraria* more than tripled in cover, thereby suppressing other taxa (Fig. S1.5).

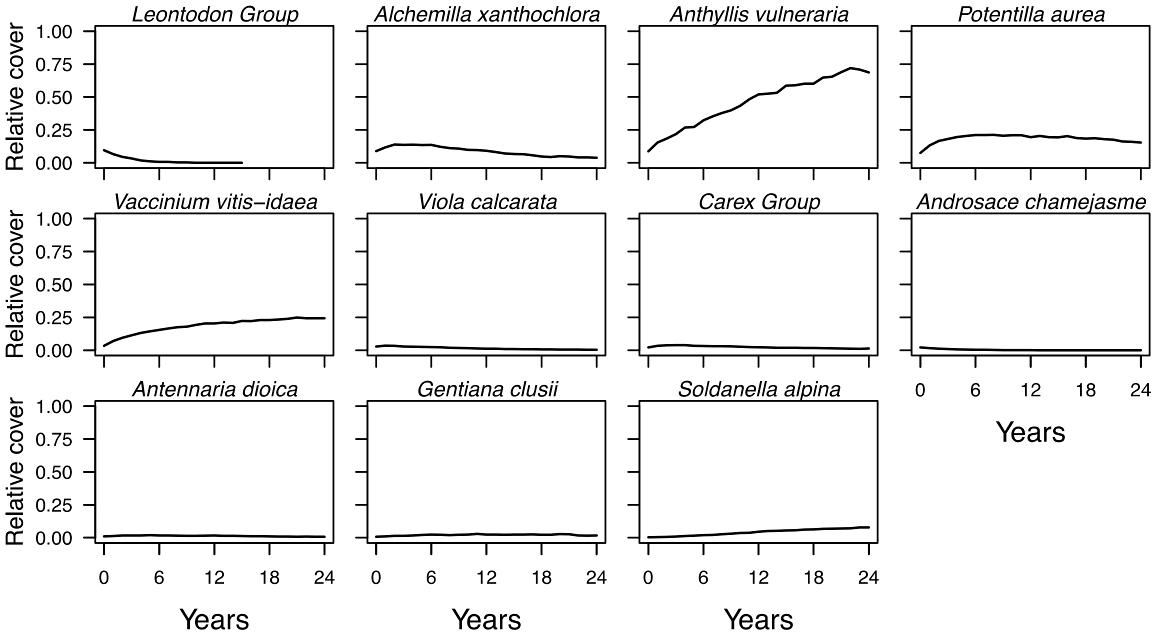

Figure S1.5 Taxa cover dynamics during burn in period to let community reach quasi-equilibrium with mean climatic conditions from 2017 to 2020 across RCP scenarios.

7. CH2018 climate change scenarios for Switzerland

We used the CH2018 climate change scenarios for Switzerland, which are based on downscaling of EURO-CORDEX climate projections under three contrasting greenhouse gas scenarios (or Representative Concentration Pathways, RCP): RCP2.6, RCP4.5, and RCP8.5 (CH2018 Project Team 2018), corresponding to optimistic, realistic, and worst-case climate futures, respectively. The CH2018 scenarios include daily time series of temperature, precipitation, and other meteorological variables for individual weather stations in Switzerland from 1981 to 2099. We used the time series for the Chur weather station (i.e., the nearest station to our experimental sites) and averaged temperature, precipitation, and relative humidity projections from different global and regional circulation models. Next, using the same regressions with which we estimated weather at the 2000-m site based on meteorological measurements in Chur (i.e., the weather data used to fit demographic models, see section 2.1.2, Supporting Information 2), we predicted air temperature and soil moisture time series at the 2000-m site under the different climate change scenarios. We noticed, however, that the CH2018 simulations for air temperature in Chur were consistently colder than the actual

temperature measurements at the weather station during the experimental period, by about 0.79 K on average (fig. S2.18). Hence, we added 0.79 K to the entire time series of simulated climate before using our regression models to predict conditions at the 2000-m site.

8. Individual-based model simulations

The starting point for the simulations was a 160 cm x 400 cm virtual turf composed of the ten 2000-m communities mapped in 2017 (arranged in a 5 × 2 tile array). For each taxon, we first simulated the survival of each ramet based on the climatic conditions, ramet size, and crowding by neighbors. Then, we simulated the growth of the surviving ramets. To avoid unrealistic ramet sizes due the growth model's tendency to overestimate variance (see simulation tests, Supporting Information 3), we set limits to the minimum (0.1 cm^2) and maximum (300 cm^2) sizes a ramet could attain. Finally, we simulated recruitment in each of the 25-cm^2 quadrants of the plot unoccupied by the taxon. If a recruit emerged, it was assigned a cover taken from a distribution of the observed cover values of that taxon's recruits.

We ran all simulations using mean parameter estimates. To avoid low crowding of ramets near the edges of the simulated turf, we assumed that their neighborhood beyond the turf's edges had the same density of both conspecifics and heterospecifics as within the turf (further details and code in Supporting Information 2, section 3.2.1).

Literature cited

Boeck, H.J.D., Bassin, S., Verlinden, M., Zeiter, M. & Hiltbrunner, E. (2016). Simulated heat waves affected alpine grassland only in combination with drought. *New Phytologist*, 209, 531–541.

CH2018 Project Team. (2018). CH2018 - Climate Scenarios for Switzerland. *National Centre for Climate Services*.

Choat, B., Brodribb, T.J., Brodersen, C.R., Duursma, R.A., López, R. & Medlyn, B.E. (2018). Triggers of tree mortality under drought. *Nature*, 558, 531–539.

Guo, Z.-W., Hu, J.-J., Chen, S.-L., Li, Y.-C., Yang, Q.-P. & Cai, H.-J. (2017). Nitrogen addition and clonal integration alleviate water stress of dependent ramets of Indocalamus decorus under heterogeneous soil water environment. *Sci Rep*, 7, 44524.

Kleijn, D., Treier, U.A. & Müller-Schärer, H. (2005). The importance of nitrogen and carbohydrate storage for plant growth of the alpine herb Veratrum album. *New Phytologist*, 166, 565–575.

Liu, F., Liu, J. & Dong, M. (2016). Ecological Consequences of Clonal Integration in Plants. *Front. Plant Sci.*, 7.

McElreath, R. (2020). *Statistical Rethinking : A Bayesian Course with Examples in R and Stan*. 2nd edn. Chapman and Hall/CRC, Boca Raton.

Roiloa, S.R., Antelo, B. & Retuerto, R. (2014). Physiological integration modifies δ15N in the clonal plant Fragaria vesca, suggesting preferential transport of nitrogen to water-stressed offspring. *Ann Bot*, 114, 399–411.
