# Supplementary material for: Ecological lags govern the pace and outcome of plant community responses to 21st‐century climate change
Source: Ecol Lett. 2022 Aug 26;25(10):2156–66. doi: 10.1111/ele.14087 (PMC9804264; doi:10.1111/ele.14087)
Supplement: Supplementary file 2 — Appendix S2 [file ELE-25-2156-s002.pdf]

SM2: Detailed methods and code for data processing, analyses, and modeling

Contents

1. Data loading, cleaning and pre-processing **1**
1.1 Weather data . . . 1
1.1.1 Chur weather data . . . 1
1.1.2 Site weather data (2019 and 2020) . . . 3
1.1.3 Predicting weather along the elevational gradient based on Chur weather . . . 8
1.2 Cleaning raw vegetation data . . . 28
1.3 Tracking individual plants (or ramets) . . . 32
1.4 Quantifying demography . . . 42
1.4.1 Survival and growth . . . 42
1.4.2 Recruitment . . . 60

2. Statistical models of plant demography **69**
2.1 Survival . . . 69
2.2 Growth . . . 75
2.3 Recruitment . . . 84

3. Simulations of community dynamics **87**
3.1 Individual-based model . . . 87
3.2.1 Preparing input data . . . 87
3.2.2 Simulator functions . . . 98
3.2 Observed vs predicted dynamics during experimental period . . . 118
3.3 Simulated dynamics under future climate change . . . 134
3.3.1 Swiss Climate Change Scenarios . . . 134
3.3.2 Simulations . . . 144
3.4 Analysing trajectories of community responses to climate change . . . 159

4. Code to reproduce result figures **161**
Figure 2 . . . 162
Figure 3 . . . 168
Figure 4 . . . 171
Supplementary figures . . . 175

The entire workflow will be maintained on GitHub.

1. Data loading, cleaning and pre-processing

1.1 Weather data

1.1.1 Chur weather data

A weather station belonging to the Swiss Meteorological Service (MeteoSchweiz) in the nearby city of Chur provided continuous weather records for the entire duration of the experiment.

```
library(readr)
# Load Chur weather station data
```

```r
wsdata <- read_delim("data/abiotic/chur_ms/chur_temp_rain.txt",
                     ";", escape_double = FALSE,
                     col_types = cols(time = col_date(format = "%Y%m%d")),
                     trim_ws = TRUE, skip = 1)

# Rename columns
names(wsdata)[3:6] <- c("temp", "temp2", "rain", "rain2")

# Keep only records after spring 2016
wsdata <- subset(wsdata, time > "2016-05-31")

# Make figure
par(mfrow=c(2,1), mar=c(0.5,4,1,1), oma = c(2,0,1,0))
plot(temp ~ time, wsdata, type = "l",  xaxt = "n", col = "darkred", las = 1,
     ylab = "Temperature (ºC)")
mtext("Daily average temperature and total precipitation from weather station in Chur",
      adj = 0, cex = 0.7, col = "darkgray")
mtext("Weather during transplant experiment",
      adj = 0, cex = 1.05, line = 1)
plot(rain ~ time, wsdata, type = "l", col = "blue", las = 1,
     ylab = "Precipitation (mm)", yaxp = c(0,60, 4))
```

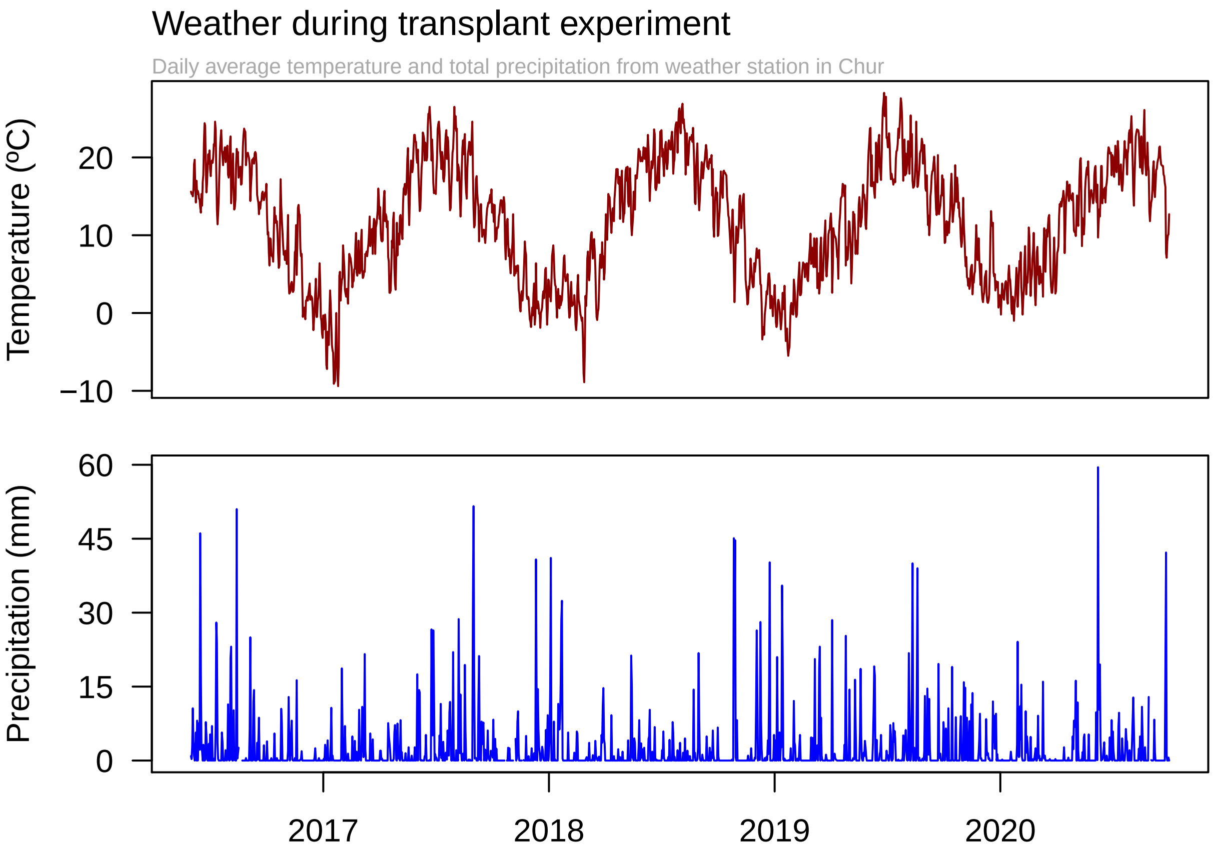

Figure S2.1 Weather conditions in Chur during the experimental period.

1.1.2 Site weather data (2019 and 2020)

To measure weather conditions across the elevational gradient, we deployed air temperature (DECAGON ECH2O Temperature/RH) and soil moisture sensors (DECAGON ECH2O TE Moisture/Temp/EC) at the five experimental sites from late May to early October in 2019 and 2020.

Here, we load and format the data from these weather stations.

```
# Load 2019 data from weather stations at the sites
# Arella (1000 m)
are19 <- read_csv("data/abiotic/weather_stations/are19.csv",
                  col_types = cols(time = col_datetime(format = "%m.%d.%Y %I:%M %p")))

# Nesselboden (1400 m)
nes19 <- read_csv("data/abiotic/weather_stations/nes19.csv")
nes19$datetime <- as.POSIXlt(nes19$time, format = "%m.%d.%y %H:%M")
nes19$datetime[is.na(nes19$datetime)] <- as.POSIXlt(nes19$time[is.na(nes19$datetime)],
                                                    format = "%m.%d.%Y %I:%M %p")


# Baerenmoss (1600 m)
bar19 <- read_csv("data/abiotic/weather_stations/bar19.csv")
bar19$datetime <- as.POSIXlt(bar19$time, format = "%m.%d.%y %H:%M")
bar19$datetime[is.na(bar19$datetime)] <- as.POSIXlt(bar19$time[is.na(bar19$datetime)],
                                                    format = "%m.%d.%Y %I:%M %p")

# Padora (1800 m)
pad19 <- read_csv("data/abiotic/weather_stations/pad19.csv")
pad19$datetime <- as.POSIXlt(pad19$time, format = "%m.%d.%y %H:%M")
pad19$datetime[is.na(pad19$datetime)] <- as.POSIXlt(pad19$time[is.na(pad19$datetime)],
                                                    format = "%m.%d.%Y %I:%M %p")

# Calanda (2000 m)
cal19 <- read_csv("data/abiotic/weather_stations/cal19.csv")
cal19$datetime <- as.POSIXct(cal19$time, format = "%m.%d.%y %H:%M")
cal19$datetime[is.na(cal19$datetime)] <- as.POSIXct(cal19$time[is.na(cal19$datetime)],
                                                    format = "%m.%d.%Y %I:%M %p")

# Create date variable
are19$date <- as.Date(are19$time)
nes19$date <- as.Date(nes19$datetime)
bar19$date <- as.Date(bar19$datetime)
pad19$date <- as.Date(pad19$datetime)
cal19$date <- as.Date(cal19$datetime)


# Load 2020 data from weather stations at the sites
# Arella (1000 m)
are20 <- read_csv("data/abiotic/weather_stations/are20.csv",
                  col_types = cols(time = col_datetime(format = "%m.%d.%Y %I:%M %p")))

# Nesselboden (1400 m)
nes20 <- read_csv("data/abiotic/weather_stations/nes20.csv")
nes20$datetime <- as.POSIXlt(nes20$time, format = "%m.%d.%y %H:%M")
nes20$datetime[is.na(nes20$datetime)] <- as.POSIXlt(nes20$time[is.na(nes20$datetime)],
```

```
                                                   format = "%m.%d.%Y %I:%M %p")

# Baerenmoss (1600 m)
bar20 <- read_csv("data/abiotic/weather_stations/bar20.csv")
bar20$datetime <- as.POSIXlt(bar20$time, format = "%m.%d.%y %H:%M")
bar20$datetime[is.na(bar20$datetime)] <- as.POSIXlt(bar20$time[is.na(bar20$datetime)],
                                                   format = "%m.%d.%Y %I:%M %p")


# Padora (1800 m)
pad20 <- read_csv("data/abiotic/weather_stations/pad20.csv")
pad20$datetime <- as.POSIXlt(pad20$time, format = "%m.%d.%y %H:%M")
pad20$datetime[is.na(pad20$datetime)] <- as.POSIXlt(pad20$time[is.na(pad20$datetime)],
                                                   format = "%m.%d.%Y %I:%M %p")

# Calanda (2000 m)
cal20 <- read_csv("data/abiotic/weather_stations/cal20.csv")
cal20$datetime <- as.POSIXlt(cal20$time, format = "%m.%d.%y %H:%M")
cal20$datetime[is.na(cal20$datetime)] <- as.POSIXlt(cal20$time[is.na(cal20$datetime)],
                                                   format = "%m.%d.%Y %I:%M %p")

# Create date variable
are20$date <- as.Date(are20$time)
nes20$date <- as.Date(nes20$datetime)
bar20$date <- as.Date(bar20$datetime)
pad20$date <- as.Date(pad20$datetime)
cal20$date <- as.Date(cal20$datetime)
```

Then we calculated daily mean values of temperature and soil moisture. For a subset of the sites (1,000 and 1,400 m in 2019, 1,400 and 2,000 m in 2020), we also measured daily precipitation.

```
## 2019
are19d <- data.frame(date = as.Date(levels(factor(as.character(are19$date)))),
                     are_temp = tapply(are19$air_temp, are19$date, mean, na.rm = T),
                     are_rain = tapply(are19$rain, are19$date, sum),
                     are_soilT = tapply(are19$soil_temp, are19$date, mean, na.rm = T),
                     are_soilM = tapply(are19$soil_moisture,
                                        are19$date, mean, na.rm = T))


nes19d <- data.frame(date = as.Date(levels(factor(as.character(nes19$date)))),
                     nes_temp = tapply(nes19$air_temp, nes19$date, mean, na.rm = T),
                     nes_rain = tapply(nes19$rain, nes19$date, sum),
                     nes_soilT = tapply(nes19$soil_temp, nes19$date, mean, na.rm = T),
                     nes_soilM = tapply(nes19$soil_moisture,
                                        nes19$date, mean, na.rm = T))


bar19d <- data.frame(date = as.Date(levels(factor(as.character(bar19$date)))),
                     bar_temp = tapply(bar19$air_temp, bar19$date, mean, na.rm = T),
                     bar_soilT = tapply(bar19$soil_temp, bar19$date, mean, na.rm = T),
                     bar_soilM = tapply(bar19$soil_moisture,
                                        bar19$date, mean, na.rm = T))
```

```r
pad19d <- data.frame(date = as.Date(levels(factor(as.character(pad19$date)))),
                     pad_temp = tapply(pad19$air_temp, pad19$date, mean, na.rm = T),
                     pad_soilT = tapply(pad19$soil_temp, pad19$date, mean, na.rm = T),
                     pad_soilM = tapply(pad19$soil_moisture,
                                        pad19$date, mean, na.rm = T))


cal19d <- data.frame(date = as.Date(levels(factor(as.character(cal19$date)))),
                     cal_temp = tapply(cal19$air_temp, cal19$date, mean, na.rm = F),
                     cal_soilT = (tapply(cal19$soil_temp,
                                         cal19$date, mean, na.rm = T) +
                                  tapply(cal19$soil_temp2,
                                         cal19$date, mean, na.rm = T))/2,
                     cal_soilM = (tapply(cal19$soil_moisture,
                                         cal19$date, mean, na.rm = T) +
                                  tapply(cal19$soil_moisture2,
                                         cal19$date, mean, na.rm = T))/2
)


## 2020
are20d <- data.frame(date = as.Date(levels(factor(as.character(are20$date)))),
                     are_temp = tapply(are20$air_temp, are20$date, mean, na.rm = F),
                     are_rain = tapply(are20$rain, are20$date, sum),
                     are_soilT = tapply(are20$soil_temp, are20$date, mean, na.rm = T),
                     are_soilM = tapply(are20$soil_moisture, are20$date, mean, na.rm = T)
                     )




nes20d <- data.frame(date = as.Date(levels(factor(as.character(nes20$date)))),
                     nes_temp = tapply(nes20$air_temp, nes20$date, mean, na.rm = F),
                     nes_rain = tapply(nes20$rain, nes20$date, sum),
                     nes_soilT = tapply(nes20$soil_temp, nes20$date, mean, na.rm = T),
                     nes_soilM = tapply(nes20$soil_moisture, nes20$date, mean, na.rm = T)
)


bar20d <- data.frame(date = as.Date(levels(factor(as.character(bar20$date)))),
                     bar_temp = tapply(bar20$air_temp, bar20$date, mean, na.rm = F),
                     bar_soilT = tapply(bar20$soil_temp, bar20$date, mean, na.rm = T),
                     bar_soilM = tapply(bar20$soil_moisture, bar20$date, mean, na.rm = T)
)


pad20d <- data.frame(date = as.Date(levels(factor(as.character(pad20$date)))),
                     pad_temp = tapply(pad20$air_temp, pad20$date, mean, na.rm = F),
                     pad_soilT = tapply(pad20$soil_temp, pad20$date, mean, na.rm = T),
                     pad_soilM = tapply(pad20$soil_moisture, pad20$date, mean, na.rm = T)
```

```
)


cal20d <- data.frame(date = as.Date(levels(factor(as.character(cal20$date)))),
                     cal_temp = tapply(cal20$air_temp, cal20$date, mean, na.rm = F),
                     cal_rain = tapply(cal20$rain, cal20$date, sum),
                     cal_soilT = (tapply(cal20$soil_temp1, cal20$date, mean, na.rm = T) +
                                  tapply(cal20$soil_temp2, cal20$date, mean, na.rm = T))
                     /2,
                     cal_soilM = (tapply(cal20$soil_moisture1, cal20$date,
                                         mean, na.rm = T) +
                                          tapply(cal20$soil_moisture2, cal20$date,
                                                 mean, na.rm = T))/2
)


# Make plot of seasonal variation in temperature and soil moisture at Arella (1,000 m)
par(mfrow=c(2,1), mar=c(1,4,1,1), oma = c(2,0,2,0))
plot(are_temp~date, are19d, type="l", xaxt = "n", bty = "n",
     col = rgb(0.75,0,0,0.8), lwd = 2, ylab = "ºC")
mtext("Air temperature", adj = 0, cex = 0.9, col = "darkred")
plot(are_soilM~date, are19d, type="l", bty = "n",
     col = rgb(0,0,1,0.8), lwd = 2, ylab = "VWC")
mtext("Soil moisture", adj = 0, cex = 0.9, col = rgb(0,0,1,0.8))
```

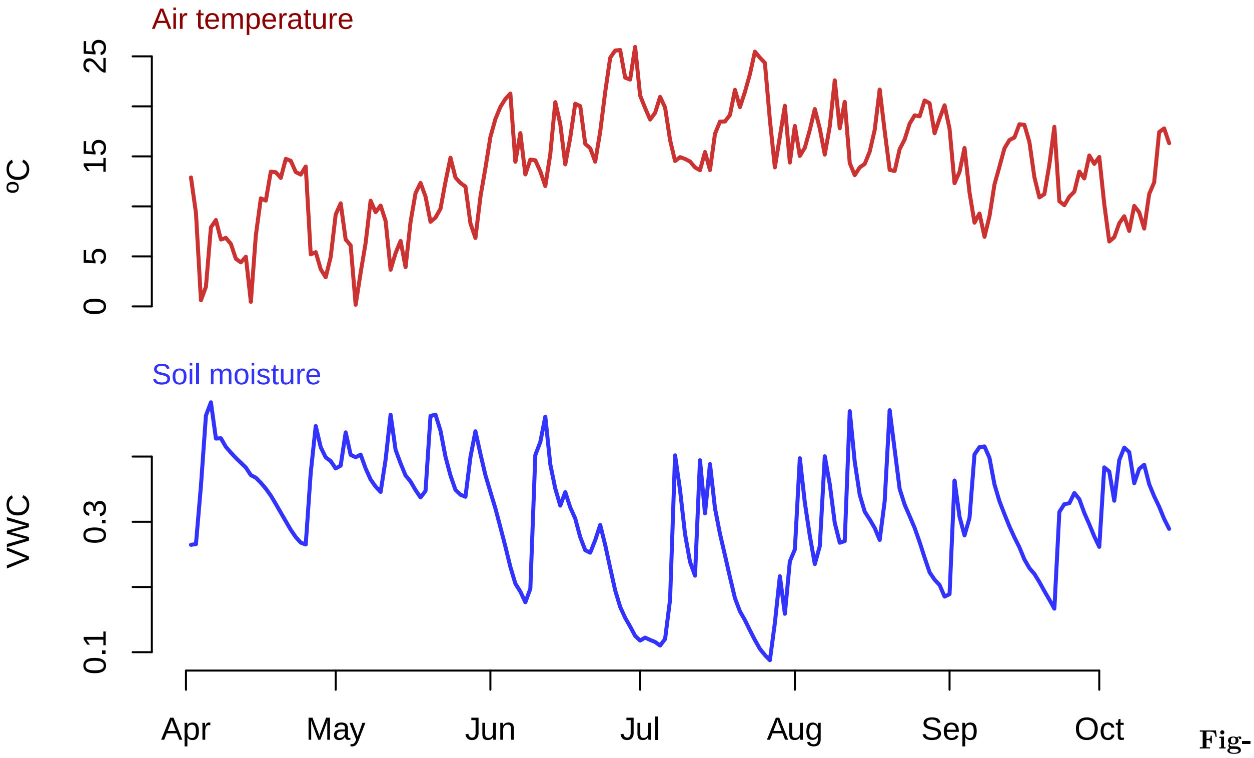

Figure S2.2 Air temperature (at 20 cm height) and soil moisture at the 1,000 m site during the 2019 growing season.

Now, let's create a data frame `calandaclim` in which to combine weather measurments at Chur and at the 5 experimental sites along the elevational gradient.

```r
# Keep date, temp, and rain columns from Chur weather station data
calandaclim <- wsdata[,c(2:3,5)]

# Keep only records after Feb 2019
calandaclim <- subset(calandaclim, time >= "2019-03-01")
names(calandaclim)[1] <- "date"

# Add columuns with data from sites
## Starting with 2019 data
calandaclim <- merge(calandaclim, are19d, all.x = T)
calandaclim <- merge(calandaclim, nes19d, all.x = T)
calandaclim <- merge(calandaclim, bar19d, all.x = T)
calandaclim <- merge(calandaclim, pad19d, all.x = T)
calandaclim <- merge(calandaclim, cal19d, all.x = T)

calandaclim$rain <- as.numeric(calandaclim$rain)

## Now 2020
calandaclim$are_temp[calandaclim$date %in% are20d$date] <-
        are20d$are_temp[are20d$date %in% calandaclim$date]

calandaclim$are_soilT[calandaclim$date %in% are20d$date] <-
        are20d$are_soilT[are20d$date %in% calandaclim$date]

calandaclim$are_soilM[calandaclim$date %in% are20d$date] <-
        are20d$are_soilM[are20d$date %in% calandaclim$date]


calandaclim$nes_temp[calandaclim$date %in% nes20d$date] <-
        nes20d$nes_temp[nes20d$date %in% calandaclim$date]

calandaclim$nes_rain[calandaclim$date %in% nes20d$date] <-
        nes20d$nes_rain[nes20d$date %in% calandaclim$date]

calandaclim$nes_soilT[calandaclim$date %in% nes20d$date] <-
        nes20d$nes_soilT[nes20d$date %in% calandaclim$date]

calandaclim$nes_soilM[calandaclim$date %in% nes20d$date] <-
        nes20d$nes_soilM[nes20d$date %in% calandaclim$date]


calandaclim$bar_temp[calandaclim$date %in% bar20d$date] <-
        bar20d$bar_temp[bar20d$date %in% calandaclim$date]

calandaclim$bar_soilT[calandaclim$date %in% bar20d$date] <-
        bar20d$bar_soilT[bar20d$date %in% calandaclim$date]

calandaclim$bar_soilM[calandaclim$date %in% bar20d$date] <-
        bar20d$bar_soilM[bar20d$date %in% calandaclim$date]


calandaclim$pad_temp[calandaclim$date %in% pad20d$date] <-
        pad20d$pad_temp[pad20d$date %in% calandaclim$date]
```

```
calandaclim$pad_soilT[calandaclim$date %in% pad20d$date] <-
        pad20d$pad_soilT[pad20d$date %in% calandaclim$date]

calandaclim$pad_soilM[calandaclim$date %in% pad20d$date] <-
        pad20d$pad_soilM[pad20d$date %in% calandaclim$date]

calandaclim$pad_temp[calandaclim$date %in% pad20d$date] <-
        pad20d$pad_temp[pad20d$date %in% calandaclim$date]


calandaclim$cal_temp[calandaclim$date %in% cal20d$date] <-
        cal20d$cal_temp[cal20d$date %in% calandaclim$date]

calandaclim$cal_soilT[calandaclim$date %in% cal20d$date] <-
        cal20d$cal_soilT[cal20d$date %in% calandaclim$date]

calandaclim$cal_soilM[calandaclim$date %in% cal20d$date] <-
        cal20d$cal_soilM[cal20d$date %in% calandaclim$date]

calandaclim$cal_rain <- rep(NA, times = nrow(calandaclim))

calandaclim$cal_rain[calandaclim$date %in% cal20d$date] <-
        cal20d$cal_rain[cal20d$date %in% calandaclim$date]
```

1.1.3 Predicting weather along the elevational gradient based on Chur weather

To complete the weather time series across the elevational gradient, we can use our weather measurementes from 2019 and 2020 to train models to predict weather at the different sites based on weather conditions at the nearby city of Chur (Fig 1A in main text).

We already have daily mean records of temperature and precipitation, but a few other covariables could influence the correlation between weather conditions in Chur (at an elevation of 556 m) and conditions along the mountain slope. Namely, we explored the potential influence of relative humidity, sunshine duration, and global radiation.

```
## Load and process covariate data
wcova <- read_delim("data/abiotic/chur_ms/chur_covariates.txt",
                    ";", escape_double = FALSE, trim_ws = TRUE,
                    col_types = cols(time = col_date(format = "%Y%m%d")),
                    skip = 1)

## Rename columns
names(wcova)[2:5] <- c("date", "radiation", "rh", "sunshine")

## Keep only data after Feb 2019
wcc <- subset(wcova, date >= "2019-03-01")[,2:5]
```

Let's merge these covariate data with our data frame `calandaclim` and save it.

```
calandaclim <- merge(calandaclim, wcc, all.x = T)
write_csv(calandaclim, 'processed-data/calandaclim.csv')
```

Modeling soil moisture

First, let's model mean daily soil moisture at the Calanda site (2000 m) as a function of mean daily temperature, total daily rain, and mean relative humidity at Chur. However, soil moisture likely also depends on the rain of previous days. Thus, let's add another couple of variables indicating the average rain in the last three and five days, respectively.

```
### Adding previous rain
## Mean over last three days
calandaclim$l3drain <- numeric(nrow(calandaclim))
for(i in 3:nrow(calandaclim)){
        calandaclim$l3drain[i] <- mean(calandaclim$rain[(i-2):i])
}

## Mean over last five days
calandaclim$l5drain <- numeric(nrow(calandaclim))
calandaclim$l5dtemp <- numeric(nrow(calandaclim))
for(i in 5:nrow(calandaclim)){
        calandaclim$l5drain[i] <- mean(calandaclim$rain[(i-4):i])
        calandaclim$l5dtemp[i] <- mean(calandaclim$temp[(i-4):i])
}
```

Now, let's fit models including either of these three variables, and compare their predictive ability with WAIC. First the model with total daily rain.

```
# We will fit Bayesian models with Stan and the library rethinking
library(rethinking)

## Rain current day
# Data for model
d_list <- list(
    cal_soilM = calandaclim$cal_soilM[
                !is.na(calandaclim$cal_soilM)& !is.na(calandaclim$l5drain)],
    temp = calandaclim$temp[!is.na(calandaclim$cal_soilM) & !is.na(calandaclim$l5drain)],
    rain =  calandaclim$rain[!is.na(calandaclim$cal_soilM) & !is.na(calandaclim$l5drain)],
    rh = calandaclim$rh[!is.na(calandaclim$cal_soilM)& !is.na(calandaclim$l5drain)]
)

#Scale variables
d_list <- list(
        cal_soilM = d_list$cal_soilM,
        temp = (d_list$temp - mean(d_list$temp))/sd(d_list$temp),
        rain =  (d_list$rain - mean(d_list$rain)/sd(d_list$rain)),
        rh = (d_list$rh - mean(d_list$rh))/sd(d_list$rh)
)

# Model 1
modcalb <- quap(alist(
        cal_soilM ~ dnorm(mu, sigma),
        mu <- bT * temp + bH*rh*temp + bR * rain + b0,
        b0 ~ dnorm(0.3, 0.05),
        bT ~ dnorm(0, 0.5),
        bR ~ dnorm(1, 0.5),
        bH ~ dnorm(0, 3),
        sigma ~ dexp(1)
), data = d_list)
```

```r
## Rain last three days
# Data for model
d_list <- list(
    cal_soilM = calandaclim$cal_soilM[
                !is.na(calandaclim$cal_soilM)& !is.na(calandaclim$l5drain)],
    temp = calandaclim$temp[!is.na(calandaclim$cal_soilM)& !is.na(calandaclim$l5drain)],
    rain =  calandaclim$l3drain[!is.na(calandaclim$cal_soilM) &
                                    !is.na(calandaclim$l5drain)],
    rh = calandaclim$rh[!is.na(calandaclim$cal_soilM)& !is.na(calandaclim$l5drain)]
)

#Scale variables
d_list <- list(
        cal_soilM = d_list$cal_soilM,
        temp = (d_list$temp - mean(d_list$temp))/sd(d_list$temp),
        rain =  sqrt(d_list$rain),
        rh = (d_list$rh - mean(d_list$rh))/sd(d_list$rh)
)

# Model 2
modcalb2 <- quap(alist(
        cal_soilM ~ dnorm(mu, sigma),
        mu <- bT * temp + bH*rh*temp + bR * rain + b0,
        b0 ~ dnorm(0.3, 0.05),
        bT ~ dnorm(0, 0.5),
        bR ~ dnorm(1, 0.5),
        bH ~ dnorm(0, 3),
        sigma ~ dexp(1)
), data = d_list)



## Rain last five days

### Data for model
d_list <- list(
        cal_soilM = calandaclim$cal_soilM[
                !is.na(calandaclim$cal_soilM)& !is.na(calandaclim$l5drain)],
        temp = calandaclim$l5dtemp[!is.na(calandaclim$cal_soilM) &
                                    !is.na(calandaclim$l5drain)],
        rain =  calandaclim$l5drain[!is.na(calandaclim$cal_soilM) &
                                     !is.na(calandaclim$l5drain)],
        rh = calandaclim$rh[!is.na(calandaclim$cal_soilM)& !is.na(calandaclim$l5drain)]
)

# Scale variables
d_list <- list(
        cal_soilM = d_list$cal_soilM,
        temp = sqrt(d_list$temp),
        rain =  sqrt(d_list$rain),
        rh = sqrt(d_list$rh)
)
```

```r
# Model 3
modcalb3 <- quap(alist(
        cal_soilM ~ dnorm(mu, sigma),
        mu <- bT * temp + bR * rain + b0,
        b0 ~ dnorm(0.3, 0.05),
        bT ~ dnorm(0, 0.5),
        bR ~ dnorm(0, 0.5),
        sigma ~ dexp(1)
), data = d_list)


compare(modcalb3, modcalb2, modcalb)
```

```
##                WAIC       SE     dWAIC      dSE    pWAIC       weight
## modcalb3 -613.3605 23.71196   0.00000       NA 4.163073 1.000000e+00
## modcalb2 -579.3042 26.75288  34.05633 14.82732 6.432883 4.024971e-08
## modcalb  -512.8400 23.36881 100.52049 17.27759 6.224196 1.486804e-22
```

Then the model with mean total rain over the last five days does the best job at predicting soil moisture at the 2000 m site. We will use this model structure to predict soil moisture in Calanda and at the other sites.

The graph below shows that the model predictions do not capture the shape of the distribution of soil moisture data, but do a remarkable job at predicting the mean (the dotted line represents the one-to-one relationship).

```r
# Predict with model 3 (rain over last 5 days)
predSM <- link(modcalb3)

# Mean predictions
predSM.mu <- apply(predSM, 2, mean)

# Plot
plot(predSM.mu, d_list$cal_soilM,
     xlab = "Soil moisture measurements (VWC)",
     ylab = "Soil moisture predictions (VWC)",
     bg = "lightblue", pch = 21)
abline(0,1, lty = 3, lwd = 1.5)
```

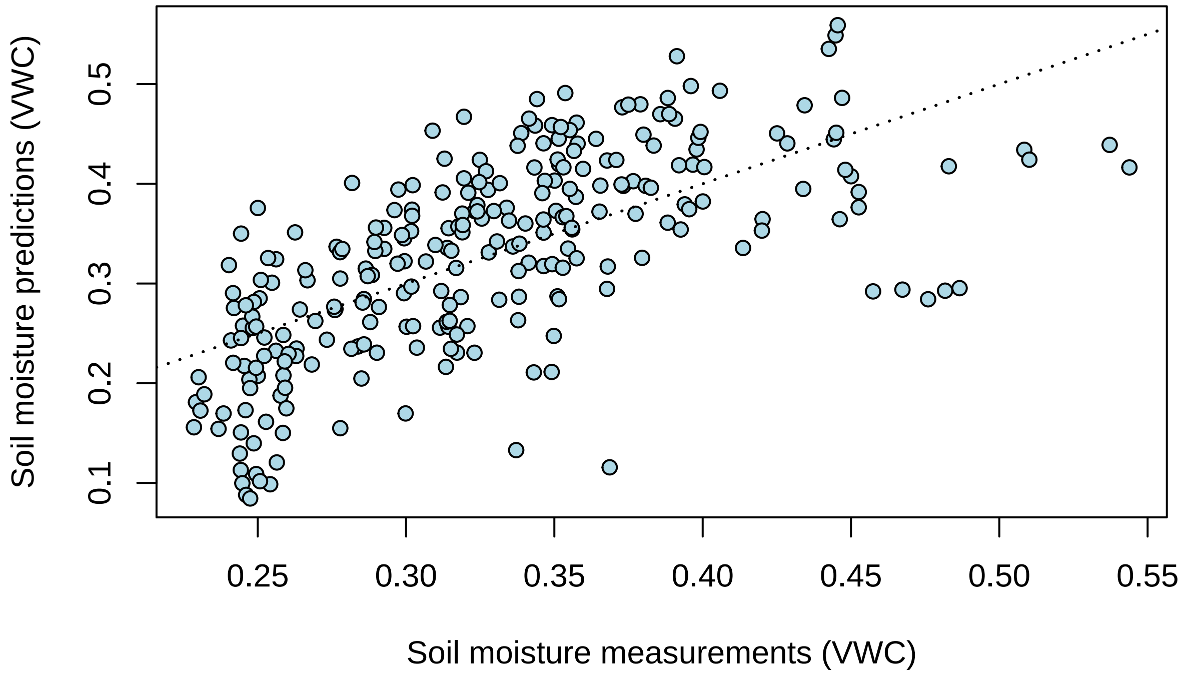

Figure S2.3 Soil moisture measurements vs model predictions at the 2,000 m site.

```r
# Correlation between predictions and measurements
cor(predSM.mu, d_list$cal_soilM)
```

```
## [1] 0.6557885
```

```r
# Summary of measurements
summary(d_list$cal_soilM)
```

```
##    Min. 1st Qu.  Median    Mean 3rd Qu.    Max.
## 0.08447 0.25724 0.33565 0.32768 0.40081 0.55905
```

```r
# Summary of predictions
summary(predSM.mu)
```

```
##    Min. 1st Qu.  Median    Mean 3rd Qu.    Max.
##  0.2285  0.2762  0.3207  0.3275  0.3597  0.5439
```

Since we aim at predicting demography as a function of mean summer conditions, we think the model can do a reasonable job describing climatic variation across sites and years.

```r
# Project Calanda soil moisture throughout experiment
names(wsdata)[2] <- "date"                ## Rename date column
wsdata <- merge(wsdata, wcova)            ## Merge Chur weather station data
wsdata$rain <- as.numeric(wsdata$rain)    ## Reformat rain as numeric variable

### Adding mean rain over previous 5 days
wsdata$l5drain <- numeric(nrow(wsdata))
wsdata$l5dtemp <- numeric(nrow(wsdata))
for(i in 5:nrow(wsdata)){
        wsdata$l5drain[i] <- mean(wsdata$rain[(i-4):i], na.rm = T)
        wsdata$l5dtemp[i] <- mean(wsdata$temp[(i-4):i], na.rm = T)
}

pred_data <- list(
```

```r
        temp = sqrt(wsdata$l5dtemp),
        rain =  sqrt(wsdata$l5drain),
        rh = sqrt(wsdata$rh))

predSM <- link(modcalb3, data = pred_data)
predSM.mu <- apply(predSM, 2, mean)

plot(wsdata$date, predSM.mu, type = "l",
     xlab = "Date", ylab = "Predicted soil moisture (VWC) at 2000 m",
     lwd = 1.5, col = col.alpha("lightblue", 0.85))

mtext("Predicted soil moisture at 2000 m throughout experimental period")
```

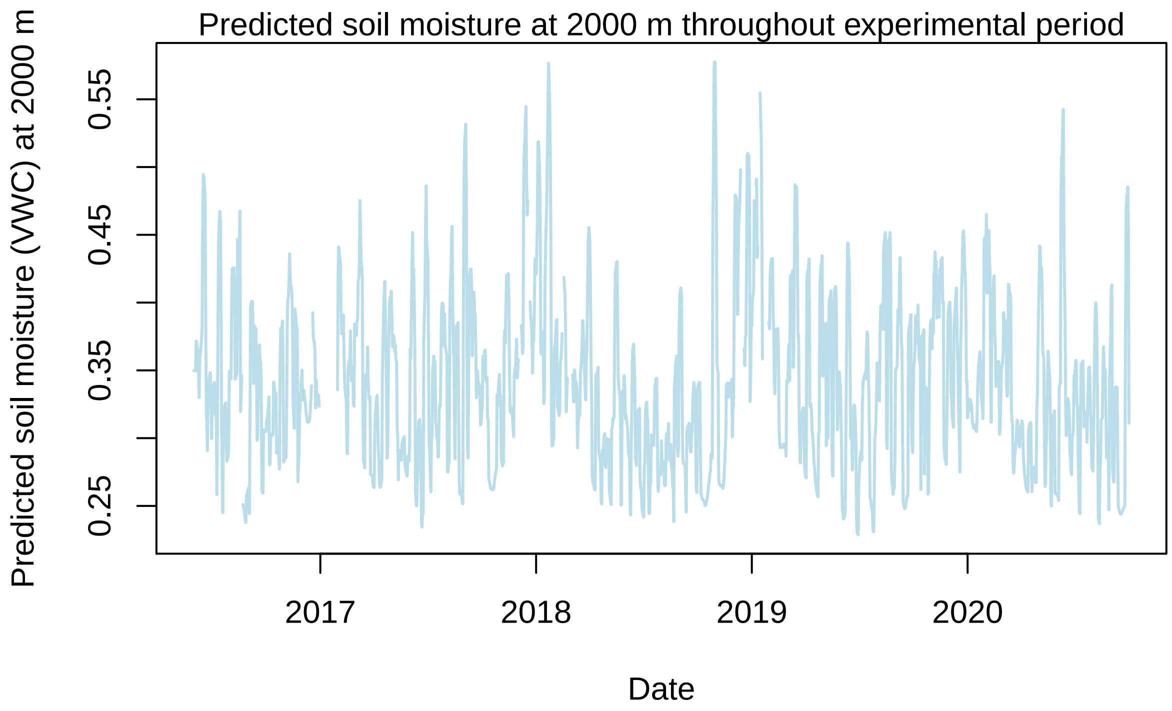


```r
wsdata$cal_predmoist <- apply(predSM, 2, mean)
```

Figure S2.4 Time series of soil moisture predictions at the 2,000 m site during the experimental period.

```r
saveRDS(modcalb3, "model-fits/abiotic/mod_cal_soilmoist.rds")
```

Next, let's fit similar models to predict soil moisture at the different experimental sites. Let's start at the lowest site, Arella, at 1000 m elevation.

```r
## Estimate SM in Arella (1000 m elevation)
d_list <- list(
        are_soilM = calandaclim$are_soilM[
                !is.na(calandaclim$are_soilM)& !is.na(calandaclim$l5drain)],
        temp = calandaclim$l5dtemp[!is.na(calandaclim$are_soilM) &
                                    !is.na(calandaclim$l5drain)],
        rain =  calandaclim$l5drain[!is.na(calandaclim$are_soilM) &
                                    !is.na(calandaclim$l5drain)],
        rh = calandaclim$rh[!is.na(calandaclim$are_soilM)& !is.na(calandaclim$l5drain)]
)
```

```r
#Scale variables
d_list <- list(
        are_soilM = d_list$are_soilM,
        temp = sqrt(d_list$temp),
        rain =  sqrt(d_list$rain),
        rh = sqrt(d_list$rh)
)

# Model
modareb3 <- quap(alist(
        are_soilM ~ dnorm(mu, sigma),
        mu <- bT * temp + bR * rain + b0,
        b0 ~ dnorm(0.3, 0.05),
        bT ~ dnorm(0, 0.5),
        bR ~ dnorm(0, 0.5),
        sigma ~ dexp(1)
), data = d_list)


# Summary of estimated parameters
precis(modareb3, depth = 2)
```

```
##               mean          sd        5.5%       94.5%
## b0      0.47939933 0.031775808  0.42861545  0.53018321
## bT     -0.08043051 0.007467208 -0.09236455 -0.06849647
## bR      0.06254718 0.004916791  0.05468920  0.07040516
## sigma   0.09212646 0.003510829  0.08651548  0.09773745
```

```r
# Predict with model
predSM <- link(modareb3)
# Calculate mean predictions
predSM.mu <- apply(predSM, 2, mean)
# Plot predictions vs measurements of soil moisture at 1000 m
plot(predSM.mu, d_list$are_soilM,
     xlab = "Soil moisture measurements (VWC)",
     ylab = "Soil moisture predictions (VWC)",
     bg = "lightblue", pch = 21)
abline(0,1, lty = 3, lwd = 1.5)
```

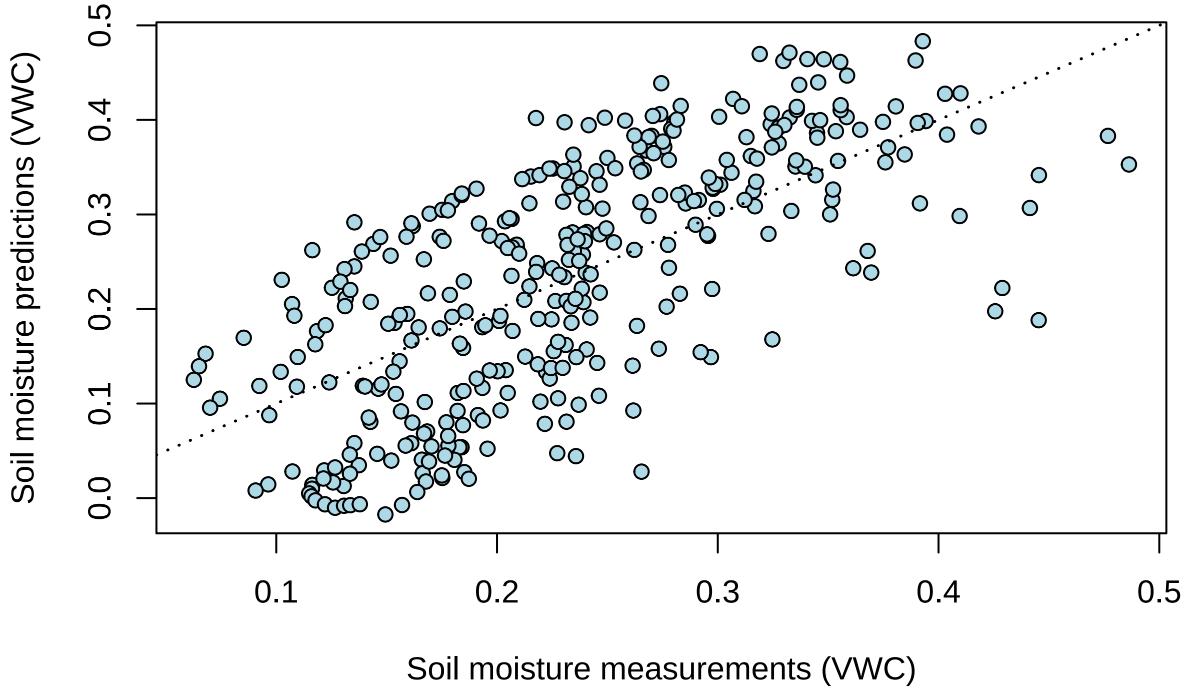

Figure S2.5 Soil moisture measurements vs model predictions at the 1,000 m site.

```
# Compare predictions
cor(predSM.mu, d_list$are_soilM)
```

```
## [1] 0.6954635
```

```
summary(predSM.mu)
```

```
##    Min. 1st Qu.  Median    Mean 3rd Qu.    Max.
## 0.06266 0.16883 0.22897 0.23344 0.28975 0.48626
```

```
summary(d_list$are_soilM)
```

```
##     Min.  1st Qu.   Median     Mean  3rd Qu.     Max.
## -0.01729  0.13382  0.25145  0.23553  0.34158  0.48323
```

```
# Predict across experimental period
pred_data <- list(
        temp = sqrt(wsdata$l5dtemp),
        rain =  sqrt(wsdata$l5drain),
        rh = sqrt(wsdata$rh))

predSM <- link(modareb3, data = pred_data)
predSM.mu <- apply(predSM, 2, mean)

wsdata$are_predmoist <- apply(predSM, 2, mean)


# Plot predicted timeseries
plot(wsdata$date, predSM.mu, type = "l",
     xlab = "Date", ylab = "Predicted soil moisture (VWC)",
     lwd = 1.5, col = col.alpha("lightblue", 0.85))
mtext("Soil moisture predictions at 1000 m throughout the experimental period",
      adj = 0, cex = 0.9)
```

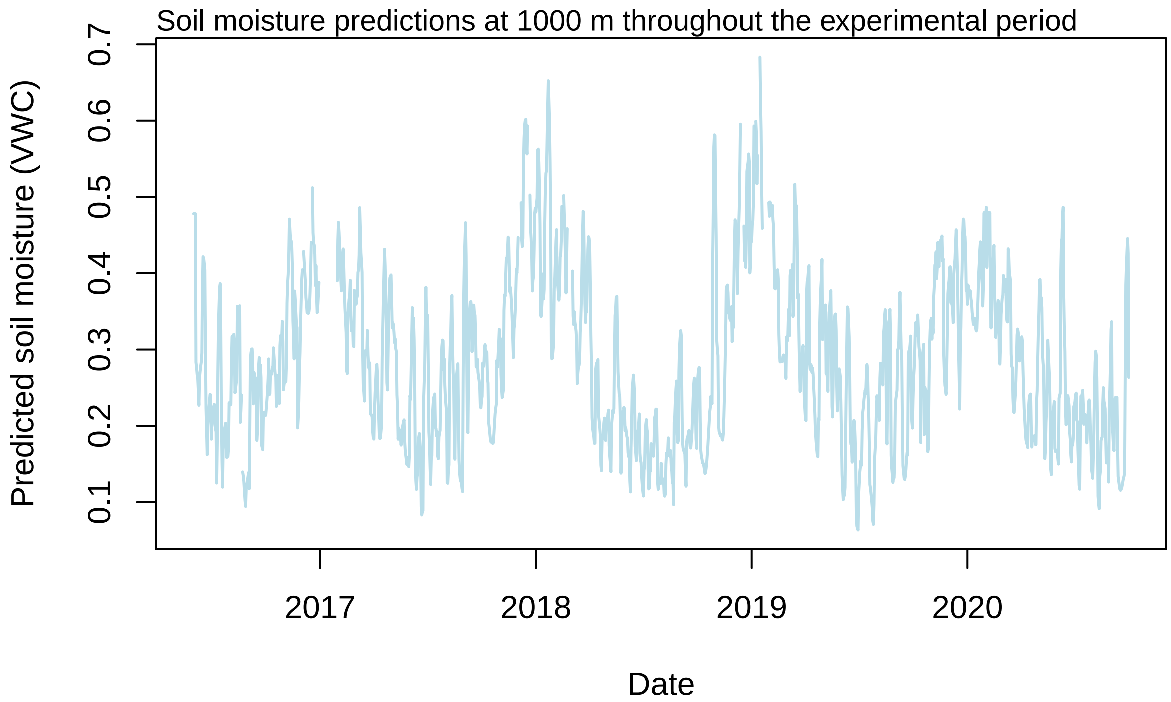

Figure S2.6 Time series of soil moisture predictions at the 1,000 m site during the experimental period.

```
saveRDS(modareb3, "model-fits/abiotic/mod_are_soilmoist.rds")
```

Not surprisingly, the predicted soil moisture at 1000 m is better predicted by weather conditions in Chur than soil moisture at 2000 m.

Next, let's do the same for soil moisture at Nesselboden, the site at 1400 m.

```
## Estimate SM in Nesselboden
d_list <- list(
        nes_soilM = calandaclim$nes_soilM[
                !is.na(calandaclim$nes_soilM)& !is.na(calandaclim$l5drain)],
        temp = calandaclim$l5dtemp[
                !is.na(calandaclim$nes_soilM)& !is.na(calandaclim$l5drain)],
        rain =  calandaclim$l5drain[
                !is.na(calandaclim$nes_soilM) & !is.na(calandaclim$l5drain)],
        rh = calandaclim$rh[!is.na(calandaclim$nes_soilM)& !is.na(calandaclim$l5drain)]
)

# Scale variables
d_list <- list(
        nes_soilM = d_list$nes_soilM,
        temp = sqrt(d_list$temp),
        rain =  sqrt(d_list$rain),
        rh = sqrt(d_list$rh)
)

# Model
modNesSM <- quap(alist(
        nes_soilM ~ dnorm(mu, sigma),
        mu <- bT * temp + bR * rain + b0,
        b0 ~ dnorm(0.3, 0.05),
        bT ~ dnorm(0, 0.5),
```

```
        bR ~ dnorm(0, 0.5),
        sigma ~ dexp(1)
), data = d_list)

# Summary of estimated parameters
precis(modNesSM, depth = 2)
```

```
##              mean          sd        5.5%       94.5%
## b0     0.35675261 0.032936624  0.30411352  0.40939169
## bT    -0.04445396 0.007624176 -0.05663886 -0.03226905
## bR     0.06476905 0.004794355  0.05710675  0.07243136
## sigma  0.08537489 0.003331653  0.08005027  0.09069952
```

```
# Predict with model
predSM <- link(modNesSM)
# Calculate mean predictions
predSM.mu <- apply(predSM, 2, mean)
# Plot predictions vs measurements of soil moisture at 1400 m
plot(predSM.mu, d_list$nes_soilM,
     xlab = "Soil moisture measurements (VWC)",
     ylab = "Soil moisture predictions (VWC)",
     bg = "lightblue", pch = 21)
abline(0,1, lty = 3, lwd = 1.5)
```

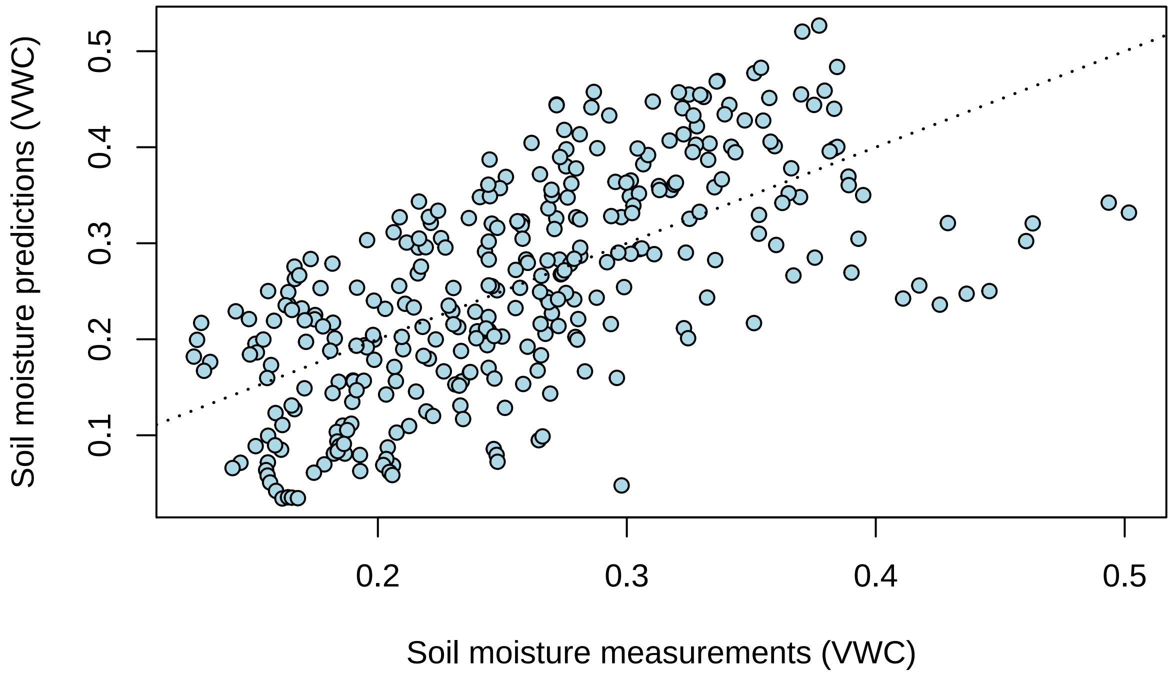

Figure S2.7 Soil moisture measurements vs model predictions at the 1,400 m site.

```
# Compare predictions
cor(predSM.mu, d_list$nes_soilM)
```

```
## [1] 0.6539737
```

```
summary(predSM.mu)
```

```
##    Min. 1st Qu.  Median    Mean 3rd Qu.    Max.
##  0.1261  0.1985  0.2511  0.2575  0.3043  0.5017
```

```r
summary(d_list$nes_soilM)
```

```
##    Min. 1st Qu.  Median    Mean 3rd Qu.    Max.
## 0.03419 0.17973 0.25354 0.25801 0.34342 0.52671
```

```r
# Predict across experimental period
pred_data <- list(
        temp = sqrt(wsdata$l5dtemp),
        rain =  sqrt(wsdata$l5drain),
        rh = sqrt(wsdata$rh))

predSM <- link(modNesSM, data = pred_data)
predSM.mu <- apply(predSM, 2, mean)

wsdata$nes_predmoist <- apply(predSM, 2, mean)

# Plot predicted timeseries
plot(wsdata$date, predSM.mu, type = "l",
     xlab = "Date", ylab = "Predicted soil moisture (VWC)",
     lwd = 1.5, col = col.alpha("lightblue", 0.85))
mtext("Soil moisture predictions at 1400 m throughout the experimental period",
      adj = 0, cex = 0.9)
```

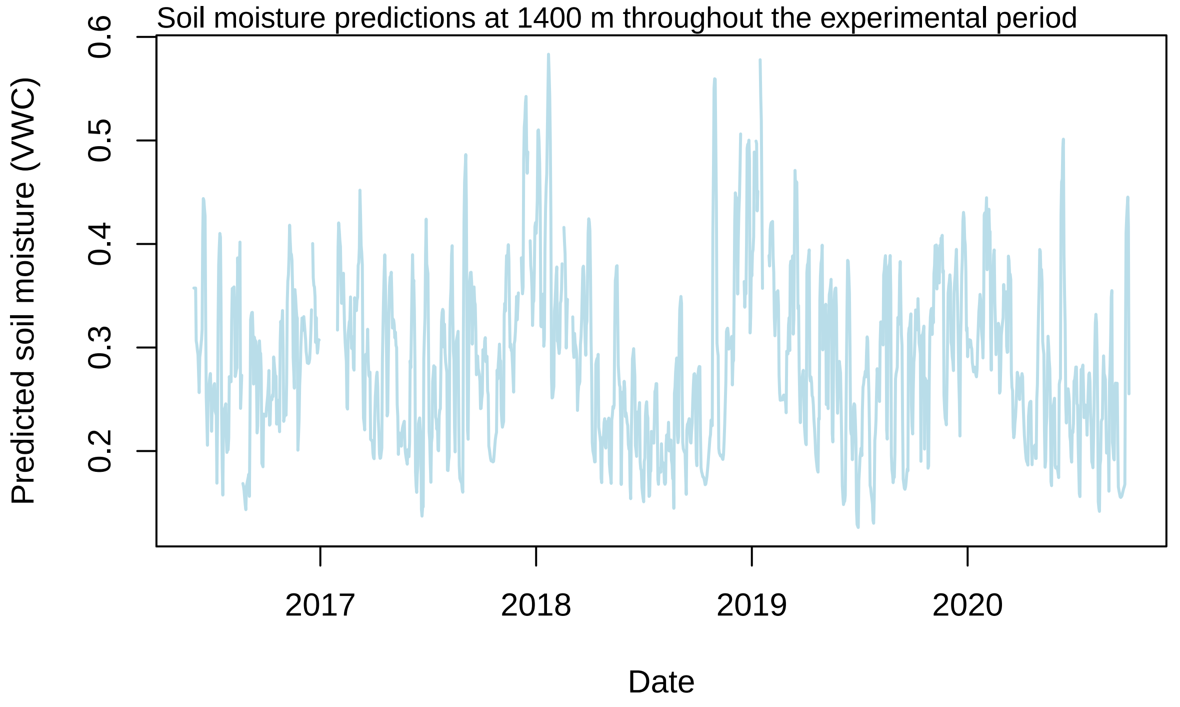

Figure S2.8 Time series of soil moisture predictions at the 1,400 m site during the experimental period.

```r
saveRDS(modNesSM, "model-fits/abiotic/mod_nes_soilmoist.rds")
```

Next, Baerenmoss, at 1600 m.

```r
## Estimate SM in Barenmoss
d_list <- list(
        bar_soilM = calandaclim$bar_soilM[!is.na(calandaclim$bar_soilM) &
                                              !is.na(calandaclim$l5drain)],
        temp = calandaclim$l5dtemp[!is.na(calandaclim$bar_soilM) &
```

```
                                        !is.na(calandaclim$l5drain)],
        rain =  calandaclim$l5drain[!is.na(calandaclim$bar_soilM) &
                                         !is.na(calandaclim$l5drain)],
        rh = calandaclim$rh[!is.na(calandaclim$bar_soilM) &
                                 !is.na(calandaclim$l5drain)]
)

#Scale variables
d_list <- list(
        bar_soilM = d_list$bar_soilM,
        temp = sqrt(d_list$temp),
        rain =  sqrt(d_list$rain),
        rh = sqrt(d_list$rh)
)

modBarSM <- quap(alist(
        bar_soilM ~ dnorm(mu, sigma),
        mu <- bT * temp + bR * rain + b0,
        b0 ~ dnorm(0.3, 0.05),
        bT ~ dnorm(0, 0.5),
        bR ~ dnorm(0, 0.5),
        sigma ~ dexp(1)
), data = d_list)

precis(modBarSM, depth = 2)
```

```
##              mean          sd        5.5%       94.5%
## b0     0.29003867 0.035588834  0.23316084  0.34691650
## bT    -0.03317637 0.008075503 -0.04608259 -0.02027016
## bR     0.06622940 0.004523053  0.05900069  0.07345812
## sigma  0.07709824 0.003166577  0.07203744  0.08215904
```

```
# Predict with model
predSM <- link(modBarSM)
# Calculate mean predictions
predSM.mu <- apply(predSM, 2, mean)
# Plot predictions vs measurements of soil moisture at 1600 m
plot(predSM.mu, d_list$bar_soilM,
     xlab = "Soil moisture measurements (VWC)",
     ylab = "Soil moisture predictions (VWC)",
     bg = "lightblue", pch = 21)
abline(0,1, lty = 3, lwd = 1.5)
```

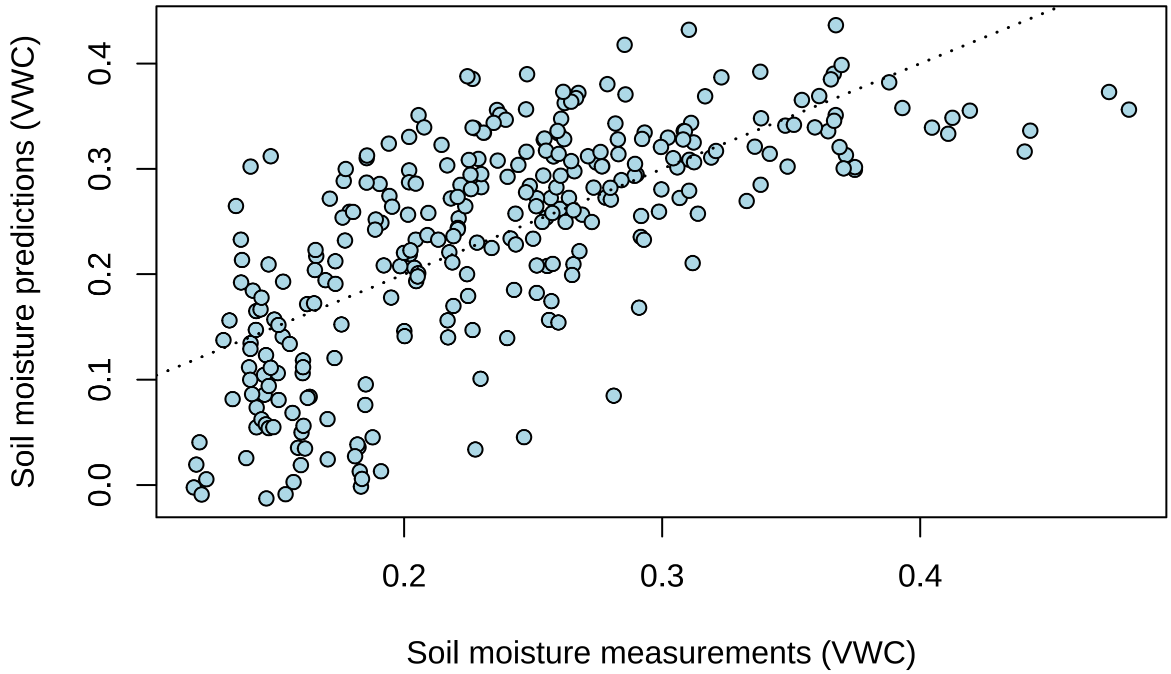

Figure S2.9 Soil moisture measurements vs model predictions at the 1,600 m site.

```
# Compare predictions
cor(predSM.mu, d_list$bar_soilM)
```

```
## [1] 0.6864424
```

```
summary(predSM.mu)
```

```
##    Min. 1st Qu.  Median    Mean 3rd Qu.    Max.
##  0.1185  0.1773  0.2292  0.2366  0.2791  0.4809
```

```
summary(d_list$bar_soilM)
```

```
##     Min.  1st Qu.   Median     Mean  3rd Qu.     Max.
## -0.01277  0.16789  0.26009  0.23631  0.31612  0.43640
```

```
# Predict across experimental period
pred_data <- list(
        temp = sqrt(wsdata$l5dtemp),
        rain =  sqrt(wsdata$l5drain),
        rh = sqrt(wsdata$rh))

predSM <- link(modBarSM, data = pred_data)
predSM.mu <- apply(predSM, 2, mean)

wsdata$bar_predmoist <- apply(predSM, 2, mean)

# Plot predicted timeseries
plot(wsdata$date, predSM.mu, type = "l",
     xlab = "Date", ylab = "Predicted soil moisture (VWC)",
     lwd = 1.5, col = col.alpha("lightblue", 0.85))
mtext("Soil moisture predictions at 1600 m throughout the experimental period",
      adj = 0, cex = 0.9)
```

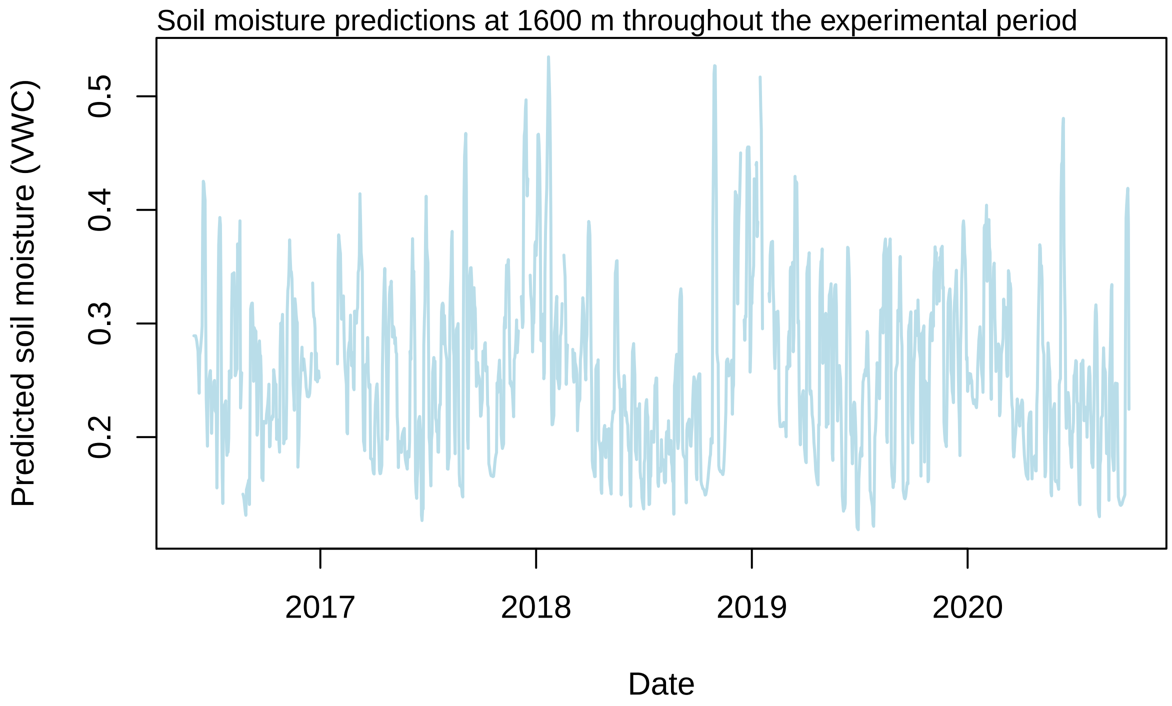

Figure S2.10 Time series of soil moisture predictions at the 1,600 m site during the experimental period.

```
saveRDS(modBarSM, "model-fits/abiotic/mod_bar_soilmoist.rds")
```

Then, Padora, at 1800 m.

```
## Estimate SM in Padenmoss
d_list <- list(
        pad_soilM = calandaclim$pad_soilM[!is.na(calandaclim$pad_soilM) &
                                      !is.na(calandaclim$l5drain)],
        temp = calandaclim$l5dtemp[!is.na(calandaclim$pad_soilM) &
                                      !is.na(calandaclim$l5drain)],
        rain =  calandaclim$l5drain[!is.na(calandaclim$pad_soilM) &
                                      !is.na(calandaclim$l5drain)],
        rh = calandaclim$rh[!is.na(calandaclim$pad_soilM)& !is.na(calandaclim$l5drain)]
)

#Scale variables
d_list <- list(
        pad_soilM = d_list$pad_soilM,
        temp = sqrt(d_list$temp),
        rain =  sqrt(d_list$rain),
        rh = sqrt(d_list$rh)
)

modPadSM <- quap(alist(
        pad_soilM ~ dnorm(mu, sigma),
        mu <- bT * temp + bR * rain + b0,
        b0 ~ dnorm(0.3, 0.05),
        bT ~ dnorm(0, 0.5),
        bR ~ dnorm(0, 0.5),
        sigma ~ dexp(1)
), data = d_list)
```

```r
precis(modPadSM, depth = 2)
```

```
##              mean          sd        5.5%       94.5%
## b0     0.38834166 0.036316440  0.33030097  0.44638234
## bT    -0.02222291 0.008182342 -0.03529988 -0.00914595
## bR     0.05714085 0.004495002  0.04995697  0.06432473
## sigma  0.07369703 0.003181981  0.06861161  0.07878245
```

```r
# Predict with model
predSM <- link(modPadSM)
# Calculate mean predictions
predSM.mu <- apply(predSM, 2, mean)
# Plot predictions vs measurements of soil moisture at 1800 m
plot(predSM.mu, d_list$pad_soilM,
     xlab = "Soil moisture measurements (VWC)",
     ylab = "Soil moisture predictions (VWC)",
     bg = "lightblue", pch = 21)
abline(0,1, lty = 3, lwd = 1.5)
```

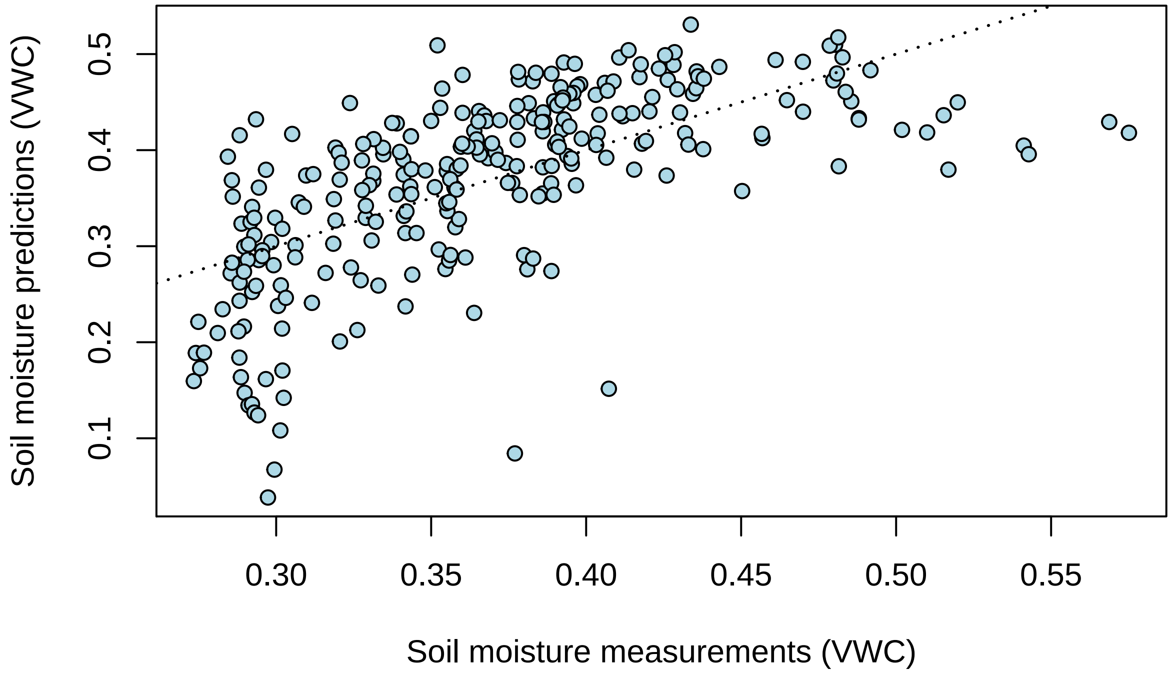

Figure S2.11 Soil moisture measurements vs model predictions at the 1,800 m site.

```r
# Compare predictions
cor(predSM.mu, d_list$pad_soilM)
```

```
## [1] 0.6424722
```

```r
summary(predSM.mu)
```

```
##    Min. 1st Qu.  Median    Mean 3rd Qu.    Max.
##  0.2734  0.3196  0.3639  0.3682  0.3983  0.5751
```

```r
summary(d_list$pad_soilM)
```

```
##    Min. 1st Qu.  Median    Mean 3rd Qu.    Max.
## 0.03833 0.31366 0.39033 0.36879 0.43819 0.53071
```

```r
# Predict across experimental period
pred_data <- list(
        temp = sqrt(wsdata$l5dtemp),
        rain =  sqrt(wsdata$l5drain),
        rh = sqrt(wsdata$rh))

predSM <- link(modPadSM, data = pred_data)
predSM.mu <- apply(predSM, 2, mean)

wsdata$pad_predmoist <- apply(predSM, 2, mean)

# Plot predicted timeseries
plot(wsdata$date, predSM.mu, type = "l",
     xlab = "Date", ylab = "Predicted soil moisture (VWC)",
     lwd = 1.5, col = col.alpha("lightblue", 0.85))
mtext("Soil moisture predictions at 1800 m throughout the experimental period",
      adj = 0, cex = 0.9)
```

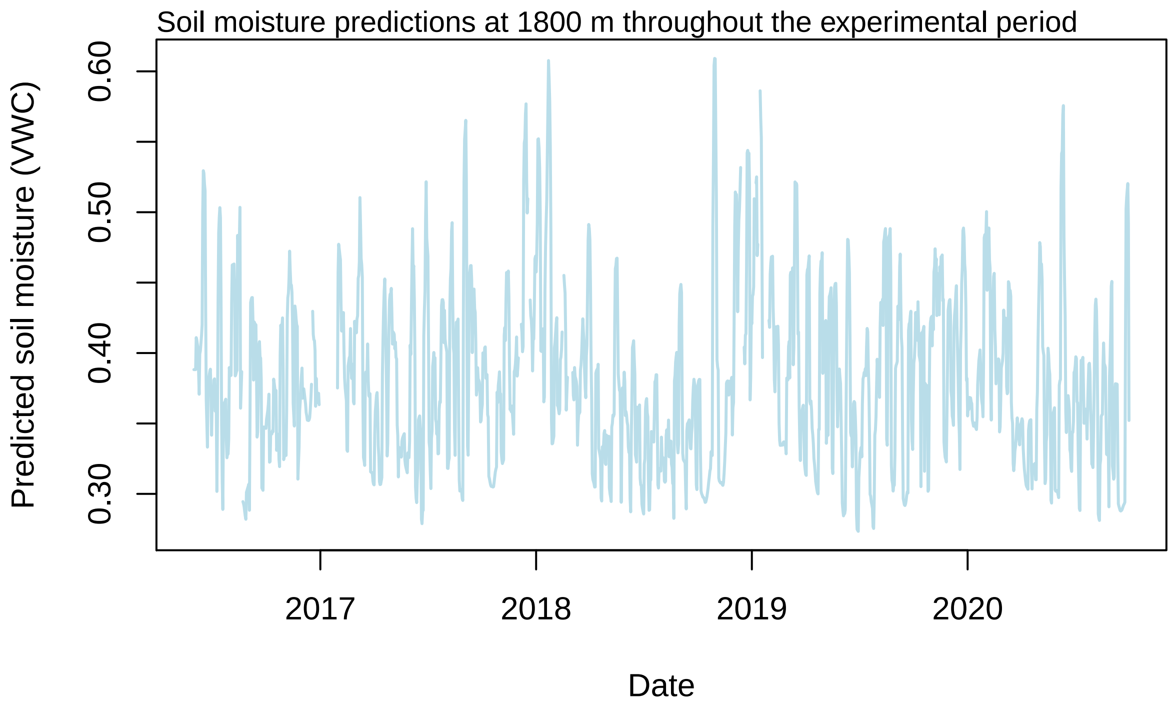

Figure S2.12 Time series of soil moisture predictions at the 1,800 m site during the experimental period.

```r
saveRDS(modPadSM, "model-fits/abiotic/mod_pad_soilmoist.rds")
```

Modeling air temperature

Having predicted soil moisture, let's now predict air temperature across the elevational gradient based on measurements at the mountain valley in Chur (556 m). We used air temperature and relative humidity as the main predictors (we also tried models with hours of sunshine and global radiation, but models with relative humidity had lower WAIC values).

```r
### Are
d_list <- list(
        are_temp = calandaclim$are_temp[!is.na(calandaclim$are_temp)],
        temp = calandaclim$temp[!is.na(calandaclim$are_temp)],
```

```r
        rh = scale(calandaclim$rh[!is.na(calandaclim$are_temp)])
)

modareb <- quap(alist(
        are_temp ~ dnorm(mu, sigma),
        mu <- bR*rh*temp + aT*temp + a,
        bR ~ dnorm(0, 3),
        aT ~ dnorm(1, 0.5),
        a ~ dnorm(-1, 3),
        sigma ~ dexp(1)
), data = d_list)

precis(modareb, depth = 2)
```

```
##                mean          sd         5.5%        94.5%
## bR    -0.005366357 0.002330422 -0.009090821 -0.001641893
## aT     1.034827108 0.008415302  1.021377830  1.048276386
## a     -2.939954130 0.143557531 -3.169386791 -2.710521469
## sigma  0.689927340 0.025639527  0.648950423  0.730904256
```

```r
### Nes
d_list <- list(
        nes_temp = calandaclim$nes_temp[!is.na(calandaclim$nes_temp)],
        temp = calandaclim$temp[!is.na(calandaclim$nes_temp)],
        rh = scale(calandaclim$rh[!is.na(calandaclim$nes_temp)])
)

modnesb2 <- quap(alist(
        nes_temp ~ dnorm(mu, sigma),
        mu <- bR*rh*temp + aT*temp + a,
        bR ~ dnorm(0, 3),
        aT ~ dnorm(1, 0.5),
        a ~ dnorm(-1, 3),
        sigma ~ dexp(1)
), data = d_list)

precis(modnesb2, depth = 2)
```

```
##                mean          sd         5.5%       94.5%
## bR     0.009206352 0.003786079  0.003155465  0.01525724
## aT     1.073028892 0.015134425  1.048841158  1.09721663
## a     -6.025551396 0.263770552 -6.447107683 -5.60399511
## sigma  1.079648680 0.041551071  1.013242043  1.14605532
```

```r
## Barenmos model
d_list <- list(
        bar_temp = calandaclim$bar_temp[!is.na(calandaclim$bar_temp)],
        temp = calandaclim$temp[!is.na(calandaclim$bar_temp)],
        rh = scale(calandaclim$rh[!is.na(calandaclim$bar_temp)])
)

modbarb2 <- quap(alist(
        bar_temp ~ dnorm(mu, sigma),
        mu <- bR*rh*temp + aT*temp + a,
        bR ~ dnorm(0, 3),
```

```r
        aT ~ dnorm(1, 0.5),
        a ~ dnorm(-1, 3),
        sigma ~ dexp(1)
), data = d_list)

precis(modbarb2, depth = 2)
```

```
##              mean          sd        5.5%       94.5%
## bR     0.01974347 0.004570362  0.01243915  0.02704779
## aT     1.11458831 0.020406496  1.08197479  1.14720184
## a     -7.61268498 0.364550549 -8.19530717 -7.03006280
## sigma  1.21701656 0.049301930  1.13822255  1.29581056
```

```r
## Padora model
d_list <- list(
        pad_temp = calandaclim$pad_temp[!is.na(calandaclim$pad_temp)],
        temp = calandaclim$temp[!is.na(calandaclim$pad_temp)],
        rh = scale(calandaclim$rh[!is.na(calandaclim$pad_temp)])
)

modpadb2 <- quap(alist(
        pad_temp ~ dnorm(mu, sigma),
        mu <- bR*rh*temp + aT*temp + a,
        bR ~ dnorm(0, 3),
        aT ~ dnorm(1, 0.5),
        a ~ dnorm(-1, 3),
        sigma ~ dexp(1)
), data = d_list)

precis(modpadb2, depth = 2)
```

```
##              mean          sd        5.5%       94.5%
## bR     0.02831059 0.004328247  0.02139322  0.03522797
## aT     1.10132417 0.020162606  1.06910044  1.13354791
## a     -8.80061218 0.365944427 -9.38546205 -8.21576230
## sigma  1.09001833 0.046107518  1.01632961  1.16370705
```

```r
## Calanda model
d_list <- list(
        cal_temp = calandaclim$cal_temp[!is.na(calandaclim$cal_temp)],
        temp = calandaclim$temp[!is.na(calandaclim$cal_temp)],
        rh = scale(calandaclim$rh[!is.na(calandaclim$cal_temp)])
)

modcalb2 <- quap(alist(
        cal_temp ~ dnorm(mu, sigma),
        mu <- bR*rh*temp + aT*temp + a,
        bR ~ dnorm(0, 3),
        aT ~ dnorm(1, 0.5),
        a ~ dnorm(-1, 3),
        sigma ~ dexp(1)
), data = d_list)

precis(modcalb2, depth = 2)
```

```
##              mean          sd        5.5%      94.5%
## bR     0.03382366 0.004839053  0.02608992  0.0415574
## aT     1.06062084 0.022850216  1.02410178  1.0971399
## a     -8.51730038 0.416919546 -9.18361833 -7.8509824
## sigma  1.21507126 0.051945683  1.13205203  1.2980905
```

Let's save the model fits.

```
saveRDS(modareb, "model-fits/abiotic/mod_are_temp.rds")
saveRDS(modnesb2, "model-fits/abiotic/mod_nes_temp.rds")
saveRDS(modbarb2, "model-fits/abiotic/mod_bar_temp.rds")
saveRDS(modpadb2, "model-fits/abiotic/mod_pad_temp.rds")
saveRDS(modcalb2, "model-fits/abiotic/mod_cal_temp.rds")
```

Now let's use models to predict across the entire data set.

```
pred.dat <- list( temp = wsdata$temp,
                  rh = scale(wsdata$rh))

# Calanda predictions
p_post <- link( modcalb2 , data=pred.dat )
wsdata$cal_predtemp <- apply( p_post , 2 , mean )

# Arella predictions
p_post <- link( modareb , data=pred.dat )
wsdata$are_predtemp <- apply( p_post , 2 , mean )

# Nesselboden predictions
p_post <- link( modnesb2 , data=pred.dat )
wsdata$nes_predtemp <- apply( p_post , 2 , mean )

# Barenmos predictions
p_post <- link( modbarb2 , data=pred.dat )
wsdata$bar_predtemp <- apply( p_post , 2 , mean )

# Padora predictions
p_post <- link( modpadb2 , data=pred.dat )
wsdata$pad_predtemp <- apply( p_post , 2 , mean )

write_csv(wsdata, "processed-data/calanda_predicted_temps.csv")
```

To test the predictive performance of the temperature models, let's train the model with the weather measurements from one season (e.g., 2019) and use to predict temperature of the other season.

```
## Calanda model trained with 2019 data

# Training data (2019)
d_list_train <- list(
        cal_temp = calandaclim$cal_temp[!is.na(calandaclim$cal_temp) &
                                        calandaclim$date < "2020-01-01"],
        temp = calandaclim$temp[!is.na(calandaclim$cal_temp) &
                                        calandaclim$date < "2020-01-01"],
        rh = scale(calandaclim$rh[!is.na(calandaclim$cal_temp) &
                                        calandaclim$date < "2020-01-01"])
)
```

```r
# Model
modcaltemp <- quap(alist(
        cal_temp ~ dnorm(mu, sigma),
        mu <- bR*rh*temp + aT*temp + a,
        bR ~ dnorm(0, 3),
        aT ~ dnorm(1, 0.5),
        a ~ dnorm(-1, 3),
        sigma ~ dexp(1)
), data = d_list_train)


# Test data (2020)
d_list_test <- list(
        cal_temp = calandaclim$cal_temp[!is.na(calandaclim$cal_temp) &
                                        calandaclim$date > "2020-01-01"],
        temp = calandaclim$temp[!is.na(calandaclim$cal_temp) &
                                        calandaclim$date > "2020-01-01"],
        rh = scale(calandaclim$rh[!is.na(calandaclim$cal_temp) &
                                        calandaclim$date > "2020-01-01"])
)


p_post <- link( modcaltemp , data=d_list_test )

# Calculate mean predictions
predT.mu <- apply(p_post, 2, mean)
# Plot predictions vs measurements of soil moisture at 1800 m
plot(d_list_test$cal_temp, predT.mu,
     xlab = "Air temperature measurements (ºC)",
     ylab = "Air temperature predictions (ºC)",
     bg = col.alpha("darkred", 0.75), pch = 21)
abline(0,1, lty = 3, lwd = 1.5)
abline(h = mean(predT.mu), lty = 3, lwd = 2.5, col = "darkgray")
abline(v = mean(d_list_test$cal_temp), lty = 3, lwd = 2.5, col = "darkgray")
```

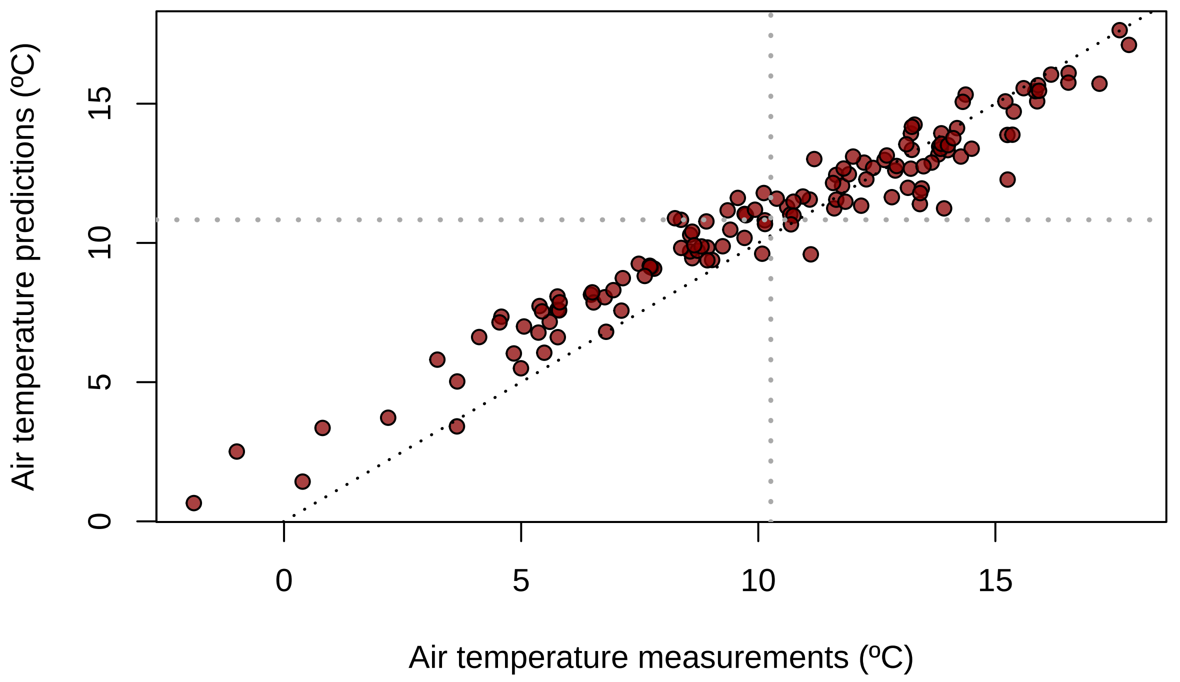

Figure S2.13 Air temperature measurements and predictions of model trained with independent data. The vertical and horizontal dotted gray lines in the plot above show the mean values of the temperature measurements and model predictions, respectively.

```
summary(predT.mu)
```

```
##    Min. 1st Qu.  Median    Mean 3rd Qu.    Max.
##  0.6568  9.0852 11.2399 10.8307 13.1020 17.6404
```

```
summary(d_list_test$cal_temp)
```

```
##    Min. 1st Qu.  Median    Mean 3rd Qu.    Max.
##  -1.902   7.633  10.682  10.265  13.413  17.817
```

While the model slightly overestimates the mean temperature at 2000 m, in gets quite close.

1.2 Cleaning raw vegetation data

The data from vegetation surveys was organized into a table (in CSV format) in which rows correspond to records of a given taxa in a given 25 cm^2 grid cell in one of the experimental turfs. Columns describe the exact location and date of the observation, the total cover of the taxa in the grid cell, as well as other metadata.

Before starting with the analyses, we cleaned the raw data by fixing some typos and solving inconsistencies in taxa's names.

```
library(readr)
library(dplyr)

# Data loading
anbc = read_csv("data/anbpc1720.csv", col_types = cols(.default = "c"))

# Keep only relevant columns
anbc = select(anbc, individual_id_year, site:quadrant, plot_id, quadrant_id,
              botanist:cover_category, cover_cm2:species_raw_changed)
```

```r
# Change class of numeric variables
anbc$y_cm = as.numeric(anbc$y_cm)
anbc$x_cm = as.numeric(anbc$x_cm)
anbc$cover_cm2 = as.numeric(anbc$cover_cm2)
anbc$year = as.numeric(anbc$year)

# Adjust y coordinates to start from 'origin' (i.e., bottom-left corner of a turf)
anbc$y = 100 - anbc$y_cm
anbc$x = anbc$x_cm

# Keep only records of 'live' biomass
anbc = anbc[anbc$is_alive_biomass == T,]
sum(anbc$is_alive_biomass == F)
sum(is.na(anbc$cover_cm2))

# Remove records with missing covers, if any
anbc = anbc[!is.na(anbc$cover_cm2),]

# Name corrections
anbc$species[anbc$species == "Alchemilla conjuncta nitida"] = "Alchemilla conjuncta"
anbc$species[anbc$species == "Alchemilla pratensis"] = "Alchemilla xanthochlora"
anbc$species[anbc$species ==
                     "Alchemilla xanthochlora pratensis"] = "Alchemilla xanthochlora"
anbc$species[anbc$species == "Campanula scheuchzerii"] = "Campanula scheuchzeri"
anbc$species[anbc$species == "Erigeron-Aster Group"] = "Aster-Erigeron Group"
anbc$species[anbc$species == "Helianthemum nummelarium"] = "Helianthemum nummularium"
anbc$species[anbc$species == "Homogyne alpestris"] = "Homogyne alpina"
anbc$species[anbc$species == "Hypericum maculatum"] = "Hypericum perforatum"
anbc$species[anbc$species == "Leontodon spp."] = "Leontodon Group"
anbc$species[anbc$species == "Leucanthemum adjustum"] = "Leucanthemum adustum"
anbc$species[anbc$species == "Moos Group"] = "Moss Group"
anbc$species[anbc$species == "Mushroom"] = "Mushroom Group"
anbc$species[anbc$species == "Nigritella nigra"] = "Nigritella spp."
anbc$species[anbc$species == "Nigritella rubra"] = "Nigritella spp."
anbc$species[anbc$species == "Phyteuma orbicularis"] = "Phyteuma orbiculare"
anbc$species[anbc$species == "Ranunculus bolbosus"] = "Ranunculus bulbosus"
anbc$species[anbc$species == "Vaccinium vitis-idea"] = "Vaccinium vitis-idaea"
anbc$species[anbc$species == "Viola hirsuta"] = "Viola hirta"


anbc$species = gsub('Astragalus alpinus', 'Astragalus spp.', anbc$species, fixed = T)
anbc$individual_id_year = gsub('Astragalus alpinus', 'Astragalus spp.',
                                anbc$individual_id_year, fixed = T)


## Missing dates
### Assign date of other observations in same turf and year
md = anbc[is.na(anbc$date),]

for (i in 1:nrow(md)) {

        g = md$grid[i]
        p = md$plot_id[i]
```

```
        y = md$year[i]

        x = anbc[anbc$year == y,]
        x = subset(x, grid == g)
        x = subset(x, plot_id == p)

        x = x[!is.na(x$date),]
        md$date[i] = x$date[1]
}

anbc$date[is.na(anbc$date)] = md$date

rm(md, x, g, i, p, y)

### CORRECT DATE TYPOS
## Homogenize date format
anbc$date = gsub("/", ".", anbc$date, fixed = T)

# In Arella (1000 m site)
anbc$date[anbc$date == "19.08.2018" &
                  anbc$year == 2018 &
                  anbc$site == "Are"] = "19.06.2018"
anbc$date[anbc$date == "18.08.2018" &
                  anbc$year == 2018 &
                  anbc$site == "Are"] = "18.06.2018"


# In Nesselboden (1400 m site)
anbc$date[anbc$date == "28.07.2017" &
          anbc$year == 2017 &
          anbc$plot_id == "Nes08.03"] = "28.06.2017"

anbc$date = gsub("2016", "2017", anbc$date, fixed = T)

anbc$date[anbc$date == "17.06.2018" &
                  anbc$year == 2018 &
                  anbc$plot_id == "Nes04.03"] = "27.06.2018"

anbc$date[anbc$date == "02.06.2018" &
                  anbc$year == 2018 &
                  anbc$plot_id == "Nes04.03"] = "02.07.2018"

anbc$date[anbc$date == "26.08.2018" &
                  anbc$year == 2018 &
                  anbc$plot_id == "Nes10.01"] = "26.06.2018"

anbc$date[anbc$date == "03.06.2019" &
                  anbc$plot_id == "Nes02.01"] = "03.07.2019"

anbc$date[anbc$date == "25.07.2019" &
                  anbc$plot_id == "Nes06.01"] = "25.06.2019"

anbc$date[anbc$date == "07.07.2019" &
```

```
                  anbc$plot_id == "Nes09.03"] = "01.07.2019"

# In Barenmoss (1600 m site)
anbc$date[anbc$date == "19.08.2017" &
                  anbc$plot_id == "Bar03.01"] = "19.07.2017"

anbc$date[anbc$date == "23.07.2019" &
                  anbc$plot_id == "Bar02.01"] = "25.07.2019"


# In Padora (1800 m site)
anbc$date[anbc$date == "08.01.2019"] = "01.08.2019"
anbc$date[anbc$date == "08.05.2019"] = "05.08.2019"
anbc$date[anbc$date == "08.06.2019"] = "06.08.2019"

anbc$date[anbc$year == 2020 & anbc$grid == "BE" &
                  anbc$plot_id == "Pad10.01"] = "29.07.2020"

anbc$date[anbc$year == 2020 & anbc$grid == "BE" &
                  anbc$cell %in% c("E1", "D1", "C1", "B1", "A1") &
                  anbc$plot_id == "Pad10.01"] = "30.07.2020"


# In Calanda (2000 m site)
anbc$date[anbc$date == "01.08.2018" &
                  anbc$plot_id == "Cal07.01"] = "16.08.2018"

anbc$date[anbc$date == "03.08.2018" &
                  anbc$plot_id == "Cal04.01"] = "13.08.2018"

anbc$date[anbc$date == "06.06.2020" &
                  anbc$plot_id == "Cal05.01"] = "06.08.2020"


## Correct wrong quadrant assignments

# correct wrong cell in Are01.01 (checked in original fieldsheet)
anbc[anbc$quadrant_id == "Are01.01.GA.A1.1","cell"] = "A2"

anbc[anbc$quadrant_id ==
             "Are01.01.GA.A1.1","quadrant_id"] = "Are01.01.GA.A2.1"

anbc[anbc$individual_id_year == "Are01.01.GA.A1.1Alchemilla xanthochlora pratensis2017",
     "individual_id_year"] = "Are01.01.GA.A2.1Alchemilla xanthochlora pratensis2017"

anbc[anbc$individual_id_year ==
             "Are01.01.GA.A2.1Alchemilla xanthochlora pratensis2017","x"] = 12.5


## Lump species that became groups
anbc$species[anbc$species == "Carex sempervirens" & (anbc$plot %in% 1:7)] = "Carex Group"
anbc$species[anbc$species == "Sesleria caerulea" & (anbc$plot %in% 1:7)] = "Grass Group"
anbc$species[anbc$species == "Nardus stricta" & (anbc$plot %in% 1:7)] = "Festuca Group"
```

```r
# Check for missing values
sum(is.na(anbc$x))
sum(is.na(anbc$species))
sum(is.na(anbc$cover_cm2))
sum(is.na(anbc$plot_id))
sum(is.na(anbc$site))


## Keep only plots without additional experimental treatments
turfinfo = read_delim("~/Dropbox/ChangA_CSV/CSV/CSV_Plot/plots(301018).csv",
                       ";", escape_double = FALSE, trim_ws = TRUE)

focalturfs = turfinfo$plot_id[turfinfo$has_focals == TRUE &
                               turfinfo$site == "Nes"]


ft = anbc[(anbc$plot_id %in% focalturfs),]
anbc = anbc[!(anbc$plot_id %in% focalturfs),]


## Correct cover typos
anbc$data_raw[anbc$individual_id_year ==
                      "Cal01.01.AL.D4.3Anthyllis vulneraria2019"] = "125"
anbc$cover_category[anbc$individual_id_year ==
                            "Cal01.01.AL.D4.3Anthyllis vulneraria2019"] = "125"
anbc$cover_cm2[anbc$individual_id_year ==
                       "Cal01.01.AL.D4.3Anthyllis vulneraria2019"] = 25*1.25

## Calculate log cover
anbc$logcov = log(anbc$cover_cm2)


write_csv(anbc, "processed-data/anbc_clean.csv")
```

1.3 Tracking individual plants (or ramets)

The data is now ready to start tracking the demographic outcome of ramets from one year to the next. We did the following stepts of the analyses for the 28 species with enough observations across the data set (> 1000) and for which all models fit without major problems. Let's define two vectors, one with the full names of the species included in the analyses (`domsp`) and one with their six-letter codes (`sppList`).

```r
# Species names
domsp = c('Leontodon Group',
           'Alchemilla xanthochlora',
           'Anthyllis vulneraria',
           'Potentilla aurea',
           'Festuca Group',
           'Helianthemum alpestre',
           'Vaccinium vitis-idaea',
           'Plantago atrata',
           'Viola calcarata',
```

```r
            'Thymus spp.',
            'Ranunculus montanus',
            'Hieracium lactucella',
            'Carex Group',
            'Androsace chamejasme',
            'Ligusticum mutellina',
            'Pulsatilla vernalis',
            'Polygonum viviparum',
            'Phyteuma orbiculare',
            'Antennaria dioica',
            'Myosotis alpestris',
            'Potentilla crantzii',
            'Lotus alpinus',
            'Gentiana clusii',
            'Luzula spp.',
            'Soldanella alpina',
            'Ranunculus acris',
            'Leucanthemum adustum',
            'Helianthemum nummularium'
)

# Species codes
sppList = c('LeoGro',
              'Alcxan',
              'Antvul',
              'Potaur',
              'FesGro',
              'Helalp',
              'Vacvit',
              'Plaatr',
              'Viocal',
              'Thyspp',
              'Ranmon',
              'Hielac',
              'CarGro',
              'Andcha',
              'Ligmut',
              'Pulver',
              'Polviv',
              'Phyorb',
              'Antdio',
              'Myoalp',
              'Potcra',
              'Lotalp',
              'Genclu',
              'Luzspp',
              'Solalp',
              'Ranacr',
              'Leuadu',
              'Helnum')
```

All the shoots of a given taxon rooted in a single 25-cm^2 quadrant were considered a 'ramet' of that taxon, and were treated as a demographic unit in downstream analyses. The coordinates of the quadrant's center were considered the location of the ramet, and its size was the total cover of the taxon in that quadrant. If

a taxon was located in the same quadrant in two consecutive years, it was considered a surviving ramet. However, the location of plants rooted at or near the edge between two quadrants was sometimes ambiguous, making it easy for a botanist to assign a different quadrant in a subsequent survey. Moreover, plant shoots may move a few centimeters when they re-sprout each year. We thus followed a series of rules to track ramets while accounting for potential small movements and for botanists assigning plants to different, neighboring quadrants. If a taxon's ramet was not recorded in the same quadrant the following year, we first searched for it in the other quadrants within the same 10 x 10 cm grid cell, since it is easier for botanists to assign a plant to a different quadrant within the same cell than to a different cell entirely. If the taxon was not recorded within the same cell, we proceeded to search for it in all the quadrants within a 5-cm radius of the original ramet's location. If the taxon was recorded in more than one of these neighboring quadrants, we randomly assigned the focal ramet's identity to one of them, weighing assignment probabilities with ramet covers. That is, larger ramets were more likely to be assigned the identity of previous year ramets and less likely to be considered new recruits (see below). If a ramet could not be tracked into the following year with this searching algorithm, it was declared dead. Ramets that could not be linked with previous-year ramets through this same process were considered new recruits.

```
for (sp in domsp) {

        sp17 = subset(anbc, species == sp & year == 2017)
        sp18 = subset(anbc, species == sp & year == 2018)
        sp19 = subset(anbc, species == sp & year == 2019)
        sp20 = subset(anbc, species == sp & year == 2020)

        genus = strsplit(tolower(sp), " ")[[1]][1]
        spepi = strsplit(tolower(sp), " ")[[1]][2]

        sp.code = paste(substr(genus, 1, 3),
                        substr(spepi, 1, 3),
                        sep = ".")


        ramets = data.frame(ramet_id = paste(sp.code, 1:nrow(sp17), sep = "_"),
                            id_17 = sp17$individual_id_year,
                            id_18 = character(nrow(sp17)),
                            id_19 = character(nrow(sp17)),
                            id_20 = character(nrow(sp17)))

        ramets$id_17 = as.character(ramets$id_17)
        ramets$id_18 = as.character(ramets$id_18)
        ramets$id_19 = as.character(ramets$id_19)
        ramets$id_20 = as.character(ramets$id_20)

        ## First transition (2017 -> 2018)

        # 1: Assign ids to plants that remain in their quadrant
        for (i in 1:nrow(ramets)) {
                quad = sp17$quadrant_id[i]

                if (quad %in% sp18$quadrant_id) {

                        r = which(sp18$quadrant_id == quad)
                        ramets$id_18[i] = as.character(sp18$individual_id_year[r])
                        rm(r)
                }
```

```r
        rm(quad)
}


# 2: Assign ids to plants that moved from their quadrant (within cell)
ghosts = which(ramets$id_18 == "")
for (i in ghosts) {

        quad = sp17$quadrant_id[i]

        if (!(quad %in% sp18$quadrant_id)) {
                xi = sp17$x[i]
                yi = sp17$y[i]
                turf = sp17$plot_id[i]

                hood = subset(sp18, plot_id == turf)
                hood = subset(hood, x < xi + 6 & x > xi - 6 &
                                        y < yi + 6 & y > yi - 6)
                hood = hood[!(hood$individual_id_year %in% ramets$id_18),]

                in.cell = sum(sp17$cell[i] == hood$cell)

                if(in.cell > 0) {
                        hood = hood[sp17$cell[i] == hood$cell,]
                        if(in.cell == 1){
                                hood = hood[hood$cell == sp17$cell[i],]
                                r = which(sp18$quadrant_id == hood$quadrant_id)
                                ramets$id_18[i] = sp18$individual_id_year[r]
                        } else {
                                probs = hood$cover_cm2/max(hood$cover_cm2)
                                random = sample(1:nrow(hood), 1, prob = probs)
                                hood = hood[random,]
                                r = which(sp18$quadrant_id == hood$quadrant_id)
                                ramets$id_18[i] = sp18$individual_id_year[r]
                        }

                        rm(xi, yi, turf, hood, r)
                }
                rm(quad)
        }
}


# 3: track plants that move outside cell
ghosts = which(ramets$id_18 == "")
for (i in ghosts){
        quad = sp17$quadrant_id[i]
        if (!(quad %in% sp18$quadrant_id)) {
                xi = sp17$x[i]
                yi = sp17$y[i]
                turf = sp17$plot_id[i]

                hood = subset(sp18, plot_id == turf)
```

```r
                hood = subset(hood, x < xi + 6 & x > xi - 6 &
                                    y < yi + 6 & y > yi - 6)
                hood = hood[!(hood$individual_id_year %in% ramets$id_18),]

                if(nrow(hood) > 1) {
                        probs = hood$cover_cm2/max(hood$cover_cm2)
                        random = sample(1:nrow(hood), 1, prob = probs)
                        hood = hood[random,]
                        r = which(sp18$quadrant_id == hood$quadrant_id)
                        ramets$id_18[i] = sp18$individual_id_year[r]
                }
                if(nrow(hood) == 1) {
                        r = which(sp18$quadrant_id == hood$quadrant_id)
                        ramets$id_18[i] = sp18$individual_id_year[r]
                } else{
                        ramets$id_18[i] = NA
                }
        }

        rm(xi, yi, turf, hood)
}


## Add ramets that recruited in 2018
nnr = sum(!(sp18$individual_id_year %in% ramets$id_18))
recs18 = data.frame(ramet_id = paste(sp.code,
                                     seq(nrow(ramets)+1,
                                         nrow(ramets)+nnr, by = 1),
                                     sep = "_"),
                    id_17 = NA,
                    id_18 = sp18$individual_id_year[
                            !(sp18$individual_id_year %in% ramets$id_18)
                            ],
                    id_19 = character(nnr),
                    id_20 = character(nnr))


ramets = rbind(ramets, recs18)
rm(recs18)

## Second transition (2018 -> 2019)

# 1: Assign ids to plants that remain in their quadrant
rams18 = which(!(is.na(ramets$id_18)))
for (i in rams18) {
        quad = subset(sp18, individual_id_year == ramets$id_18[i])$quadrant_id

        if(length(quad) > 0) {
                if (quad %in% sp19$quadrant_id) {

                        r = which(sp19$quadrant_id == quad)
                        ramets$id_19[i] = as.character(sp19$individual_id_year[r])
                        rm(r)
```

```r
                        }
                }
                rm(quad)
        }

        sum(ramets$id_19 != "")
        nrow(sp19)
        length(unique(ramets$id_19))

        # 2: Assign ids to plants that moved from their quadrant (within cell)
        ghosts = which(ramets$id_19 == "")
        for (i in ghosts) {
                q18 = subset(sp18, individual_id_year == ramets$id_18[i])
                quad = q18$quadrant_id

                if(length(quad) > 0) {
                        if (!(quad %in% sp19$quadrant_id)) {
                                xi = q18$x
                                yi = q18$y
                                turf = q18$plot_id

                                hood = subset(sp19, plot_id == turf)
                                hood = subset(hood, x < xi + 6 & x > xi - 6 &
                                                y < yi + 6 & y > yi - 6)
                                hood = hood[!(hood$individual_id_year %in% ramets$id_19),]

                                in.cell = sum(q18$cell == hood$cell)

                                if(in.cell > 0) {
                                        hood = hood[q18$cell == hood$cell,]
                                        if(in.cell == 1){
                                                r = which(
                                                        sp19$quadrant_id ==
                                                                hood$quadrant_id)

                                                ramets$id_19[i] =
                                                        sp19$individual_id_year[r]
                                        } else {
                                                probs = hood$cover_cm2/max(hood$cover_cm2)

                                                random = sample(1:nrow(hood), 1,
                                                                prob = probs)

                                                hood = hood[random,]

                                                r = which(
                                                        sp19$quadrant_id ==
                                                                hood$quadrant_id)

                                                ramets$id_19[i] =
                                                        sp19$individual_id_year[r]
                                        }
```

```
                        rm(xi, yi, turf, hood, r)
                    }
                    rm(quad, q18)
                }
        }
}


sum(ramets$id_19 != "")
nrow(sp19)
length(unique(ramets$id_19))

# 3: track plants that move outside cell
ghosts = which(ramets$id_19 == "")
for (i in ghosts){
        q18 = subset(sp18, individual_id_year == ramets$id_18[i])
        quad = q18$quadrant_id

        if(length(quad) > 0) {
                if (!(quad %in% sp19$quadrant_id)) {
                        xi = q18$x
                        yi = q18$y
                        turf = q18$plot_id

                        hood = subset(sp19, plot_id == turf)
                        hood = subset(hood, x < xi + 6 & x > xi - 6 &
                                            y < yi + 6 & y > yi - 6)
                        hood = hood[!(hood$individual_id_year %in% ramets$id_19),]

                        if(nrow(hood) > 1) {
                                probs = hood$cover_cm2/max(hood$cover_cm2)
                                random = sample(1:nrow(hood), 1, prob = probs)
                                hood = hood[random,]
                                r = which(sp19$quadrant_id == hood$quadrant_id)
                                ramets$id_19[i] = sp19$individual_id_year[r]
                        }
                        if(nrow(hood) == 1) {
                                r = which(sp19$quadrant_id == hood$quadrant_id)
                                ramets$id_19[i] = sp19$individual_id_year[r]
                        } else{
                                ramets$id_19[i] = NA
                        }

                        rm(xi, yi, turf, hood)
                }
        }
        rm(quad, q18)
}

ramets$id_19 = ifelse(is.na(ramets$id_18), NA, ramets$id_19)
sum(!(is.na(ramets$id_19)))
nrow(sp19)
length(unique(ramets$id_19))
```

```r
## Add ramets that recruited in 2019
nnr = sum(!(sp19$individual_id_year %in% ramets$id_19))
recs19 = data.frame(ramet_id = paste(sp.code,
                                     seq(nrow(ramets)+1,
                                         nrow(ramets)+nnr, by = 1),
                                     sep = "_"),
                    id_17 = NA,
                    id_18 = NA,
                    id_19 = sp19$individual_id_year[
                            !(sp19$individual_id_year %in% ramets$id_19)
                            ],
                    id_20 = character(nnr))


ramets = rbind(ramets, recs19)
rm(recs19)



## Third transition (2019 -> 2020)

# 1: Assign ids to plants that remain in their quadrant
rams19 = which(!(is.na(ramets$id_19)))
for (i in rams19) {
        quad = subset(sp19, individual_id_year == ramets$id_19[i])$quadrant_id

        if(length(quad) > 0) {
                if (quad %in% sp20$quadrant_id) {

                        r = which(sp20$quadrant_id == quad)
                        ramets$id_20[i] = as.character(sp20$individual_id_year[r])
                        rm(r)
                }
        }
        rm(quad)
}

sum(ramets$id_20 != "")
nrow(sp20)
length(unique(ramets$id_20))

# 2: Assign ids to plants that moved from their quadrant (within cell)
ghosts = which(ramets$id_20 == "")
for (i in ghosts) {
        q19 = subset(sp19, individual_id_year == ramets$id_19[i])
        quad = q19$quadrant_id

        if(length(quad) > 0) {
                if (!(quad %in% sp20$quadrant_id)) {
                        xi = q19$x
                        yi = q19$y
                        turf = q19$plot_id
```

```
                                hood = subset(sp20, plot_id == turf)
                                hood = subset(hood, x < xi + 6 & x > xi - 6 &
                                                       y < yi + 6 & y > yi - 6)
                                hood = hood[!(hood$individual_id_year %in% ramets$id_20),]

                                in.cell = sum(q19$cell == hood$cell)

                                if(in.cell > 0) {
                                        hood = hood[q19$cell == hood$cell,]
                                        if(in.cell == 1){
                                                #hood = hood[hood$cell == q19$cell,]
                                                r = which(
                                                        sp20$quadrant_id ==
                                                                hood$quadrant_id
                                                        )
                                                ramets$id_20[i] =
                                                        sp20$individual_id_year[r]
                                        } else {
                                                probs = hood$cover_cm2/max(hood$cover_cm2)
                                                random = sample(1:nrow(hood), 1,
                                                                prob = probs)
                                                hood = hood[random,]
                                                r = which(
                                                        sp20$quadrant_id ==
                                                                hood$quadrant_id
                                                        )
                                                ramets$id_20[i] =
                                                        sp20$individual_id_year[r]
                                        }

                                        rm(xi, yi, turf, hood, r)
                                }
                                rm(quad, q19)
                        }
                }
        }


        sum(ramets$id_20 != "")
        nrow(sp20)
        length(unique(ramets$id_20))

        # 3: track plants that move outside cell
        ghosts = which(ramets$id_20 == "")
        for (i in ghosts){
                q19 = subset(sp19, individual_id_year == ramets$id_19[i])
                quad = q19$quadrant_id

                if(length(quad) > 0) {
                        if (!(quad %in% sp20$quadrant_id)) {
                                xi = q19$x
                                yi = q19$y
                                turf = q19$plot_id
```

```
                        hood = subset(sp20, plot_id == turf)
                        hood = subset(hood, x < xi + 6 & x > xi - 6 &
                                            y < yi + 6 & y > yi - 6)
                        hood = hood[!(hood$individual_id_year %in% ramets$id_20),]

                        if(nrow(hood) > 1) {
                                probs = hood$cover_cm2/max(hood$cover_cm2)
                                random = sample(1:nrow(hood), 1, prob = probs)
                                hood = hood[random,]
                                r = which(sp20$quadrant_id == hood$quadrant_id)
                                ramets$id_20[i] = sp20$individual_id_year[r]
                        }
                        if(nrow(hood) == 1) {
                                r = which(sp20$quadrant_id == hood$quadrant_id)
                                ramets$id_20[i] = sp20$individual_id_year[r]
                        } else{
                                ramets$id_20[i] = NA
                        }

                        rm(xi, yi, turf, hood)
                }
        }
        rm(quad, q19)
}

ramets$id_20 = ifelse(is.na(ramets$id_19), NA, ramets$id_20)
sum(!(is.na(ramets$id_20)))
nrow(sp20)
length(unique(ramets$id_20))

## Add ramets that recruited in 2020
nnr = sum(!(sp20$individual_id_year %in% ramets$id_20))
recs20 = data.frame(ramet_id = paste(sp.code,
                                     seq(nrow(ramets)+1,
                                         nrow(ramets)+nnr, by = 1),
                                     sep = "_"),
                    id_17 = NA,
                    id_18 = NA,
                    id_19 = NA,
                    id_20 = sp20$individual_id_year[!(
                          sp20$individual_id_year %in% ramets$id_20
                          )])


ramets = rbind(ramets, recs20)
rm(recs20)

rm(rams18, rams19, spepi, ghosts, nnr, genus, i, in.cell, r, random, probs)

rpath = paste("processed-data/",
              sppList[which(sp == domsp)],
              "_ramets.csv",
              sep = "")
```

```
        write_csv(ramets, rpath)
        rm(rpath)
}
```

1.4 Quantifying demography

1.4.1 Survival and growth

The tables of ramet identities generated in the previous step can be used to quantify the survival and growth of each ramet across years. In the next step, we will build tables storing the demographic information for each ramet in each annual transition (i.e, 2017 to 2018, 2018 to 2019, and 2019 to 2020). For each transition, we score whether a ramet survives (0 or 1), its size in the first (`u1`) and second (`u2`) summer of the transition, and other covariables potentially explaining demography. Namely, we quantified the crowding experienced by each ramet as the sum of distance-weighted covers of all neighboring ramets:

$$w_i, t = \sum_k e^{-\delta * d^2_{ik,t}} * u_k, t$$

The parameter 'δ' determines how fast neighbor crowding declines with distance. We used a value of '$\delta = 0.2$', which means that most crowding occurred within 15 cm from the focal ramet. The use of squared distances ('$d^2_{ik,t}$') implies a Gaussian interaction kernel.

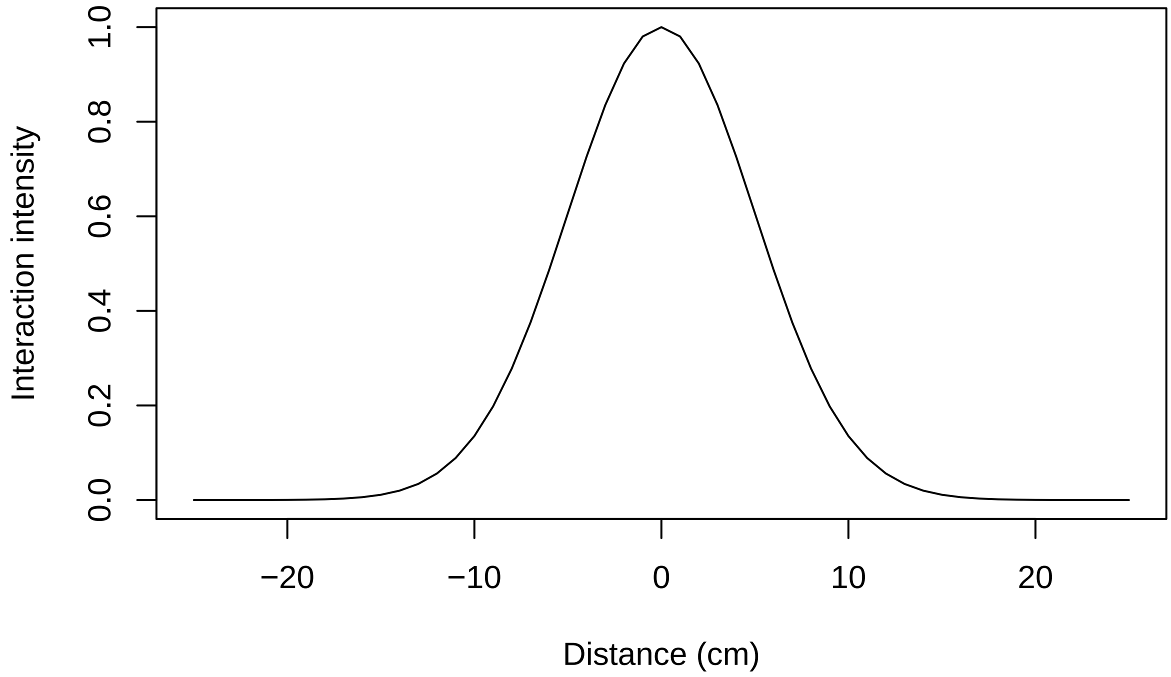

Figure S2.14 Neighbor interaction kernel.

We calculated the crowding imposed by conspecific and heterospecific neighbors separately.

```
for (sp in domsp){

        fpath_r <- paste("processed-data/",
                         sppList[which(sp==domsp)],
                         "_ramets.csv",
                         sep = "")
```

```r
ramets <- read_csv(fpath_r, col_types = cols(.default = "c"))

sp17 <- subset(anbc, species == sp & year == 2017)
sp18 <- subset(anbc, species == sp & year == 2018)
sp19 <- subset(anbc, species == sp & year == 2019)
sp20 <- subset(anbc, species == sp & year == 2020)


### ### ### ### ### ### ### ### ### ### ### ### ### ### ### ### ### ### ### ###

### ### ### ### ### ### ### ### ### ### ### ### ### ### ### ### ### ### ### ###
###              Prepare data for models of demographic rates                ###
### ### ### ### ### ### ### ### ### ### ### ### ### ### ### ### ### ### ### ###

### ### ### ### ### ### ### ### ### ### ### ### ### ### ### ### ### ### ### ###

####
# Prepare data for models of demographic rates
# 1: Prepare data


### ### ### ### ### ### ### ### ### ### ### ### ### ### ### ### ### ### ### ###

### ### ### ### ### ### ### ### ### ### ### ### ### ### ### ### ### ### ### ###
###                              First transition                            ###
### ### ### ### ### ### ### ### ### ### ### ### ### ### ### ### ### ### ### ###

### ### ### ### ### ### ### ### ### ### ### ### ### ### ### ### ### ### ### ###



ramets17 <- ramets[!is.na(ramets$id_17),]
###  a: calculate ramet survival
d17 <- data.frame(ramet = ramets17$ramet_id,
                  survival = ifelse(is.na(ramets17$id_18), 0, 1))

### Overall survival rate
sum(d17$survival/nrow(d17))

## b: add predictor variables
d17$site <- character(nrow(d17))
d17$plot <- character(nrow(d17))
d17$u1 <- NA
d17$u2 <- NA
d17$year <- numeric(nrow(d17))
d17$date <- character(nrow(d17))


for(i in 1:nrow(d17)) {
        x <- ramets17[ramets17$ramet_id == d17$ramet[i],]
        y1 <- anbc[anbc$individual_id_year == x[[2]],]
        y2 <- anbc[anbc$individual_id_year == x[[3]],]
```

```r
        d17$site[i] <- y1$site
        d17$plot[i] <- y1$plot_id
        d17$u1[i] <- y1$cover_cm2
        d17$u2[i] <- y2$cover_cm2
        d17$year[i] <- y1$year
        d17$date[i] <- y1$date
        rm(x,y1,y2)
}


## Quantify crowding (w)

# An interaction radius of ca. 15 cm seems reasonable for starters
delta <- 0.02

maxnn <- max(table(subset(sp17, species == sp)$plot_id))

rnd <- matrix(0,nrow = nrow(ramets17), ncol = maxnn)
rnc <- matrix(0,nrow = nrow(ramets17), ncol = maxnn)

for (i in 1:nrow(ramets17)) {
        plot.i <- subset(sp17, individual_id_year ==ramets17$id_17[i])$plot_id
        x <- subset(sp17, plot_id == plot.i)
        ri <- x[x$individual_id_year == ramets17$id_17[i],]
        ni <- x[x$individual_id_year != ramets17$id_17[i],]

        if(nrow(ni) > 0){
                rnd[i,1:nrow(ni)] <- sqrt((ri$x - ni$x)^2 + (ri$y - ni$y)^2)
                rnc[i,1:nrow(ni)] <- ni$cover_cm2
        }

        rm(x,ni,ri, plot.i)
}

rnd[is.na(rnd)] <- 0
rnd2 <- rnd^2

# Quantify intraspecific crowding (w)
d17$w <- numeric(nrow(d17))

for (i in 1:nrow(ramets17)){
        d17$w[i] <- sum(exp(-delta*rnd2[i,])*rnc[i,])
}

# Total community crowding (w.c)
maxncol = max(table(subset(anbc, year == 2017)$plot_id))
rnd.c <- matrix(0,nrow = nrow(ramets17), ncol = maxncol)
rnc.c <- matrix(0,nrow = nrow(ramets17), ncol = maxncol)
rm(maxncol)

for (i in 1:nrow(ramets17)) {
        ri <- subset(sp17, individual_id_year == ramets17$id_17[i])
        plot.i <- ri$plot_id
```

```
        n.ci <- subset(anbc, plot_id == plot.i & year == 2017 &
                        individual_id_year != ramets17$id_17[i])

        rnd.c[i,1:nrow(n.ci)] <- sqrt((ri$x - n.ci$x)^2 + (ri$y - n.ci$y)^2)
        rnc.c[i,1:nrow(n.ci)] <- n.ci$cover_cm2

        rm(n.ci,ri, plot.i)
}

rnd.c[is.na(rnd.c)] <- 0
rnd2.c <- rnd.c^2

d17$w.c <- numeric(nrow(d17))

for (i in 1:nrow(ramets17)){
        d17$w.c[i] <- sum(exp(-delta*rnd2.c[i,])*rnc.c[i,])
}

rm(rnd.c, rnd2.c, rnc.c)

####
# Interspecific community crowding
d17$w.cinter <- d17$w.c - d17$w

### ### ### ### ### ### ### ### ### ### ### ### ### ### ### ### ### ### ### ###

### ### ### ### ### ### ### ### ### ### ### ### ### ### ### ### ### ### ### ###
###                              Second transition                          ###
### ### ### ### ### ### ### ### ### ### ### ### ### ### ### ### ### ### ### ###

### ### ### ### ### ### ### ### ### ### ### ### ### ### ### ### ### ### ### ###

ramets18 <- ramets[!is.na(ramets$id_18),]
###  a: calculate ramet survival
d18 <- data.frame(ramet = ramets18$ramet_id,
                  survival = ifelse(is.na(ramets18$id_19), 0, 1))

### Overall survival rate
sum(d18$survival)/nrow(d18)

## b: add predictor variables
d18$site <- character(nrow(d18))
d18$plot <- character(nrow(d18))
d18$u1 <- NA
d18$u2 <- NA
d18$year <- numeric(nrow(d18))
d18$date <- character(nrow(d18))

for(i in 1:nrow(d18)) {
        x <- ramets18[ramets18$ramet_id == d18$ramet[i],]
        y1 <- anbc[anbc$individual_id_year == x[[3]],]
        y2 <- anbc[anbc$individual_id_year == x[[4]],]
```

```
        d18$site[i] <- y1$site
        d18$plot[i] <- y1$plot_id
        d18$u1[i] <- y1$cover_cm2
        d18$u2[i] <- y2$cover_cm2
        d18$year[i] <- y1$year
        d18$date[i] <- y1$date
        rm(x,y1,y2)
}


## Quantify crowding (w)
delta <- 0.02

maxnn <- max(table(subset(sp18, species == sp)$plot_id))

rnd <- matrix(0,nrow = nrow(ramets18), ncol = maxnn) # Neighbor distance matrix
rnc <- matrix(0,nrow = nrow(ramets18), ncol = maxnn) # Neighbor cover matrix

for (i in 1:nrow(ramets18)) {
        plot.i <- subset(sp18, individual_id_year ==ramets18$id_18[i])$plot_id
        x <- subset(sp18, plot_id == plot.i)
        ri <- x[x$individual_id_year == ramets18$id_18[i],]
        ni <- x[x$individual_id_year != ramets18$id_18[i],]

        if(nrow(ni) > 0){
                rnd[i,1:nrow(ni)] <- sqrt((ri$x - ni$x)^2 + (ri$y - ni$y)^2)
                rnc[i,1:nrow(ni)] <- ni$cover_cm2
        }

        rm(x,ni,ri, plot.i)
}

rnd[is.na(rnd)] <- 0
rnd2 <- rnd^2

# Quantify intraspecific crowding (w)
d18$w <- numeric(nrow(d18))

for (i in 1:nrow(ramets18)){
        d18$w[i] <- sum(exp(-delta*rnd2[i,])*rnc[i,])
}

# Total community crowding (w.c)
maxncol = max(table(subset(anbc, year == 2018)$plot_id))
rnd.c <- matrix(0,nrow = nrow(ramets18), ncol = maxncol)
rnc.c <- matrix(0,nrow = nrow(ramets18), ncol = maxncol)
rm(maxncol)

for (i in 1:nrow(ramets18)) {
        ri <- subset(sp18, individual_id_year == ramets18$id_18[i])
        plot.i <- ri$plot_id
        n.ci <- subset(anbc, plot_id == plot.i & year == 2018 &
                                individual_id_year != ramets18$id_18[i])
```

```
        rnd.c[i,1:nrow(n.ci)] <- sqrt((ri$x - n.ci$x)^2 + (ri$y - n.ci$y)^2)
        rnc.c[i,1:nrow(n.ci)] <- n.ci$cover_cm2

        rm(n.ci,ri, plot.i)
}

rnd.c[is.na(rnd.c)] <- 0
rnd2.c <- rnd.c^2

d18$w.c <- numeric(nrow(d18))

for (i in 1:nrow(ramets18)){
        d18$w.c[i] <- sum(exp(-delta*rnd2.c[i,])*rnc.c[i,])
}

rm(rnd.c, rnd2.c, rnc.c)

####
# Interspecific community crowding
maxncol = max(table(subset(anbc, year == 2018 & species != sp)$plot_id))
rnd.c <- matrix(0,nrow = nrow(ramets18), ncol = maxncol)
rnc.c <- matrix(0,nrow = nrow(ramets18), ncol = maxncol)
rm(maxncol)

for (i in 1:nrow(ramets18)) {
        ri <- subset(sp18, individual_id_year == ramets18$id_18[i])
        plot.i <- ri$plot_id
        n.ci <- subset(anbc, plot_id == plot.i & year == 2018 & species != sp)

        rnd.c[i,1:nrow(n.ci)] <- sqrt((ri$x - n.ci$x)^2 + (ri$y - n.ci$y)^2)
        rnc.c[i,1:nrow(n.ci)] <- n.ci$cover_cm2

        rm(n.ci,ri, plot.i)
}

rnd.c[is.na(rnd.c)] <- 0
rnd2.c <- rnd.c^2

# Quantify interspecific crowding (w.c)
d18$w.cinter <- numeric(nrow(d18))

for (i in 1:nrow(ramets18)){
        d18$w.cinter[i] <- sum(exp(-delta*rnd2.c[i,])*rnc.c[i,])
}

rm(rnd.c, rnd2.c, rnc.c)


### ### ### ### ### ### ### ### ### ### ### ### ### ### ### ### ### ### ### ###

### ### ### ### ### ### ### ### ### ### ### ### ### ### ### ### ### ### ### ###
###                              Third transition                             ###
### ### ### ### ### ### ### ### ### ### ### ### ### ### ### ### ### ### ### ###
```

```r
### ### ### ### ### ### ### ### ### ### ### ### ### ### ### ### ### ### ### ###

ramets19 <- ramets[!is.na(ramets$id_19),]
###  a: calculate ramet survival
d19 <- data.frame(ramet = ramets19$ramet_id,
                  survival = ifelse(is.na(ramets19$id_20), 0, 1))

### Overall survival rate
sum(d19$survival)/nrow(d19)

## b: add predictor variables
d19$site <- character(nrow(d19))
d19$plot <- character(nrow(d19))
d19$u1 <- NA
d19$u2 <- NA
d19$year <- numeric(nrow(d19))
d19$date <- character(nrow(d19))

for(i in 1:nrow(d19)) {
        x <- ramets19[ramets19$ramet_id == d19$ramet[i],]
        y1 <- anbc[anbc$individual_id_year == x[[4]],]
        y2 <- anbc[anbc$individual_id_year == x[[5]],]

        d19$site[i] <- y1$site
        d19$plot[i] <- y1$plot_id
        d19$u1[i] <- y1$cover_cm2
        d19$u2[i] <- y2$cover_cm2
        d19$year[i] <- y1$year
        d19$date[i] <- y1$date
        rm(x,y1,y2)
}


## Quantify crowding (w)
delta <- 0.02

maxnn <- max(table(subset(sp19, species == sp)$plot_id))

rnd <- matrix(0,nrow = nrow(ramets19), ncol = maxnn) # Neighbor distance matrix
rnc <- matrix(0,nrow = nrow(ramets19), ncol = maxnn) # Neighbor cover matrix

for (i in 1:nrow(ramets19)) {
        plot.i <- subset(sp19, individual_id_year ==ramets19$id_19[i])$plot_id
        x <- subset(sp19, plot_id == plot.i)
        ri <- x[x$individual_id_year == ramets19$id_19[i],]
        ni <- x[x$individual_id_year != ramets19$id_19[i],]

        if(nrow(ni) > 0){
                rnd[i,1:nrow(ni)] <- sqrt((ri$x - ni$x)^2 + (ri$y - ni$y)^2)
                rnc[i,1:nrow(ni)] <- ni$cover_cm2
        }

        rm(x,ni,ri, plot.i)
```

```
}

rnd[is.na(rnd)] <- 0
rnd2 <- rnd^2

# Quantify intraspecific crowding (w)
d19$w <- numeric(nrow(d19))

for (i in 1:nrow(ramets19)){
        d19$w[i] <- sum(exp(-delta*rnd2[i,])*rnc[i,])
}

# Total community crowding (w.c)
maxncol = max(table(subset(anbc, year == 2019)$plot_id))
rnd.c <- matrix(0,nrow = nrow(ramets19), ncol = maxncol)
rnc.c <- matrix(0,nrow = nrow(ramets19), ncol = maxncol)
rm(maxncol)

for (i in 1:nrow(ramets19)) {
        ri <- subset(sp19, individual_id_year == ramets19$id_19[i])
        plot.i <- ri$plot_id
        n.ci <- subset(anbc, plot_id == plot.i & year == 2019 &
                                individual_id_year != ramets19$id_19[i])

        rnd.c[i,1:nrow(n.ci)] <- sqrt((ri$x - n.ci$x)^2 + (ri$y - n.ci$y)^2)
        rnc.c[i,1:nrow(n.ci)] <- n.ci$cover_cm2

        rm(n.ci,ri, plot.i)
}

rnd.c[is.na(rnd.c)] <- 0
rnd2.c <- rnd.c^2

d19$w.c <- numeric(nrow(d19))

for (i in 1:nrow(ramets19)){
        d19$w.c[i] <- sum(exp(-delta*rnd2.c[i,])*rnc.c[i,])
}

rm(rnd.c, rnd2.c, rnc.c)

####
# Interspecific community crowding
maxncol = max(table(subset(anbc, year == 2019 & species != sp)$plot_id))
rnd.c <- matrix(0,nrow = nrow(ramets19), ncol = maxncol)
rnc.c <- matrix(0,nrow = nrow(ramets19), ncol = maxncol)
rm(maxncol)

for (i in 1:nrow(ramets19)) {
        ri <- subset(sp19, individual_id_year == ramets19$id_19[i])
        plot.i <- ri$plot_id
        n.ci <- subset(anbc, plot_id == plot.i & year == 2019 & species != sp)
```

```
                rnd.c[i,1:nrow(n.ci)] <- sqrt((ri$x - n.ci$x)^2 + (ri$y - n.ci$y)^2)
                rnc.c[i,1:nrow(n.ci)] <- n.ci$cover_cm2

                rm(n.ci,ri, plot.i)
        }

        rnd.c[is.na(rnd.c)] <- 0
        rnd2.c <- rnd.c^2

        # Quantify interspecific crowding (w.c)
        d19$w.cinter <- numeric(nrow(d19))

        for (i in 1:nrow(ramets19)){
                d19$w.cinter[i] <- sum(exp(-delta*rnd2.c[i,])*rnc.c[i,])
        }

        rm(rnd.c, rnd2.c, rnc.c)


        ###
        ##   COMBINE DATA
        ###
        d <- rbind(d17,d18,d19)


        d$sid <- ifelse(d$site == "Are", 1,
                        ifelse(d$site == "Nes", 2,
                               ifelse(d$site == "Bar", 3,
                                      ifelse(d$site == "Pad", 4, 5))))

        d$pid <- as.numeric(as.factor(d$plot))

        dpath <- paste("processed-data/", sppList[which(sp==domsp)], "_DMD.csv", sep = "")
        write_csv(d, dpath)
}
```

Now, let's add the x and y coordinates of each ramet.

```
for (spcode in sppList){

        fpath_d <- paste("processed-data/",spcode, "_DMD.csv", sep = "")
        d <- read_csv(fpath_d)

        fpath_r <- paste("processed-data/", spcode, "_ramets.csv", sep = "")
        ramets <- read_csv(fpath_r)


        # Get the coordinates of each ramet and add them to `d`

        d$x <- numeric(nrow(d))
        d$y <- numeric(nrow(d))


        for (i in 1:nrow(d)) {
```

```r
            yr <- d$year[i]

            if (yr == 2017) {

                    rid <- ramets$id_17[ramets$ramet_id == d$ramet[i]]
            }

            if (yr == 2018) {

                    rid <- ramets$id_18[ramets$ramet_id == d$ramet[i]]
            }

            if (yr == 2019) {

                    rid <- ramets$id_19[ramets$ramet_id == d$ramet[i]]
            }

            d$x[i] <- anbc$x[anbc$individual_id_year == rid]
            d$y[i] <- anbc$y[anbc$individual_id_year == rid]

            rm(yr, rid)
    }


    write_csv(d, fpath_d)

}
```

And finally, let's add information about the weather conditions potentially explaining ramet demography in each annual transition at the different sites. Namely, we quantified the mean temperature and soil moisture during the first summer of the transition (`templS` and `moistLS`), as well as the average of mean summer temperatures during the first year of the transition and the one before.

To do this, let's first load the predicted time series of weather conditions across the elevational gradient (see section 1.1.3).

```r
# Load weather predictions
wdat <- read_csv("processed-data/calanda_predicted_temps.csv")
wdat$rain <- as.numeric(wdat$rain)

# For the 11 missing values of daily rain total (calendar day),
# use total rain from 6 UTC to 6 UTC in the following day
wdat$rain[is.na(wdat$rain)] <- wdat$rain2[is.na(wdat$rain)]
```

Before calculating weather covariates, we estimated the period of snow cover at each site with temperature data from HOBO loggers. We assumed that if mean daily temperature was below 1 ºC and the daily temperature variance (based on measurements every 30 min), the sensor and the plants were covered in snow. Since plants would then be buffered from weather fluctuations, we excluded those dates from the weather time series.

The HOBO data is a table of daily mean and variance of ground-level temperature measured every 30 minutes. We assumed that the plants at a given site were covered in snow if the mean daily temperature recorded by the HOBO loggers was smaller than 1ºC, and the variance is also smaller than 1 (i.e., snow buffers daily temperature fluctuations).

In the plots below, the shaded blue region represents the period cover with snow at the different sites.

```r
# Load HOBO data and get dates in which sites were covered in snow
hobos <- read_csv("data/abiotic/hobos/all_daily.csv")

## Arella
arehobo <- subset(hobos, site == "Are")
aresnow <- arehobo$date[arehobo$tempvar < 1 & arehobo$temp < 1]

plot(temp~date, arehobo, type = "l",
     xlab = "Date",
     ylab = expression(paste("Temperature [",degree,"C]")))

mtext("Arella (1000 m)", line = 0.25, cex = 1.25)
abline(v = aresnow, col = rgb(0.5,0.5,0.9,0.5), lwd = 1.5)
```

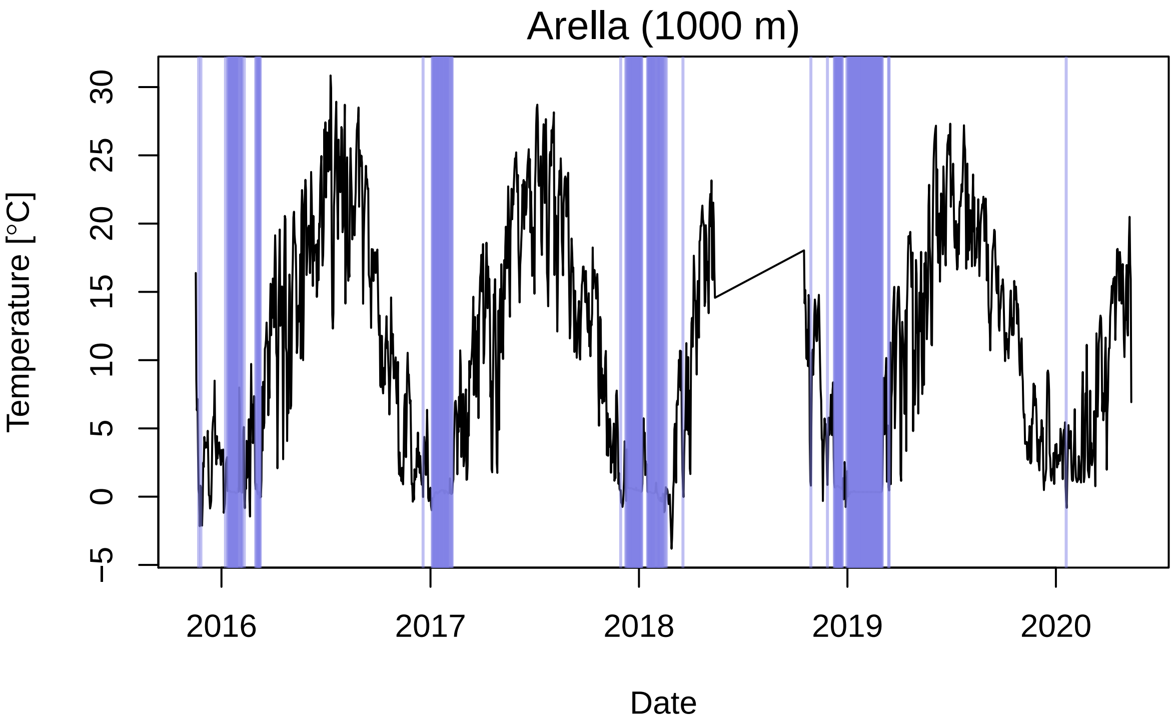


```r
rm(arehobo)

## Nesselboden
neshobo <- subset(hobos, site == "Nes")
nessnow <- neshobo$date[neshobo$tempvar < 1 & neshobo$temp < 1]

plot(temp~date, neshobo, type = "l",
     xlab = "Date",
     ylab = expression(paste("Temperature [",degree,"C]")))

mtext("Nesselboden (1400 m)", line = 0.25, cex = 1.25)
abline(v = nessnow, col = rgb(0.5,0.5,0.9,0.5), lwd = 1.5)
```

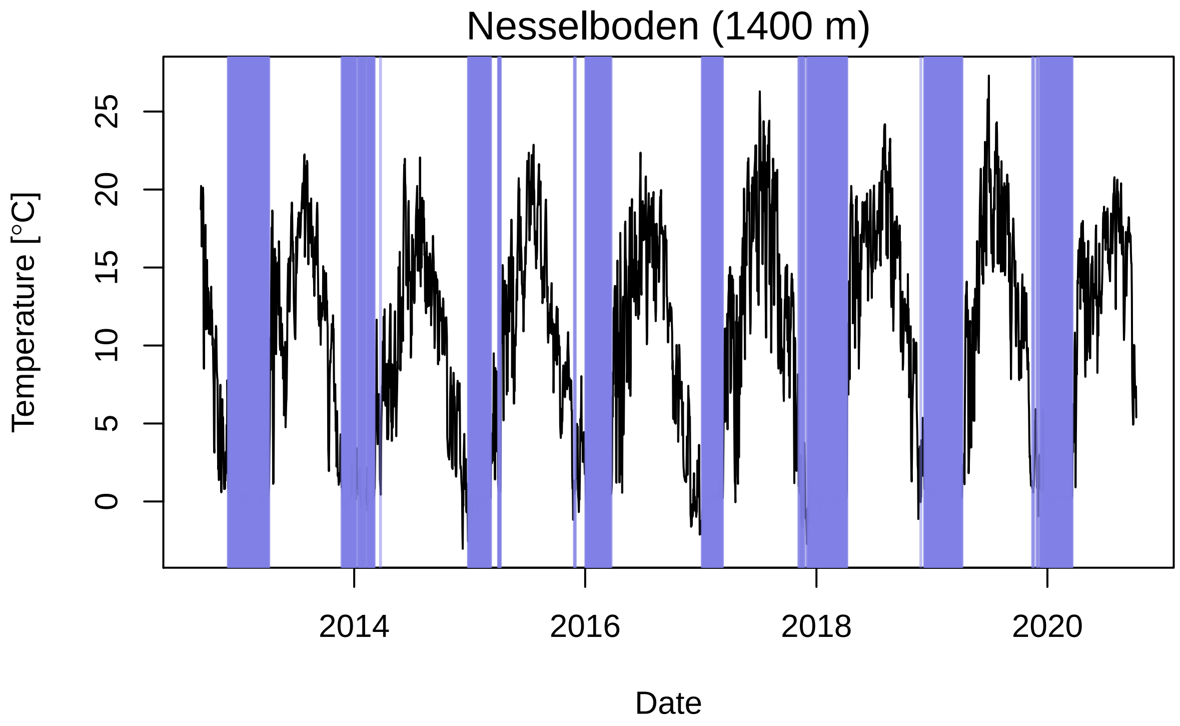


```r
rm(neshobo)


## Barenmoss
barhobo <- subset(hobos, site == "Bar")
barsnow <- barhobo$date[barhobo$tempvar < 1 & barhobo$temp < 1]

plot(temp~date, barhobo, type = "l",
     xlab = "Date",
     ylab = expression(paste("Temperature [",degree,"C]")))

mtext("Barenmoss (1600 m)", line = 0.25, cex = 1.25)
abline(v = barsnow, col = rgb(0.5,0.5,0.9,0.5), lwd = 1.5)
```

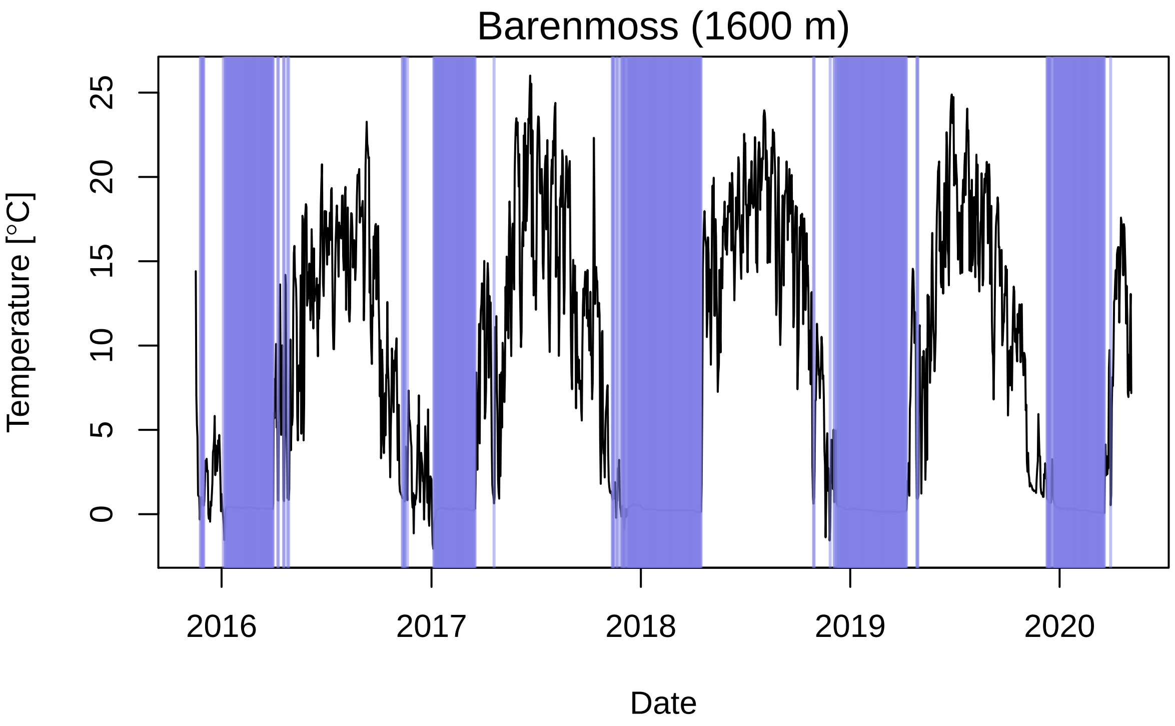


```
rm(barhobo)


## Neuensses
neuhobo <- subset(hobos, site == "Neu")
neusnow <- neuhobo$date[neuhobo$tempvar < 1 & neuhobo$temp < 1]

plot(temp~date, neuhobo, type = "l",
     xlab = "Date",
     ylab = expression(paste("Temperature [",degree,"C]")))

mtext("Neuensses (1800 m)", line = 0.25, cex = 1.25)
abline(v = neusnow, col = rgb(0.5,0.5,0.9,0.5), lwd = 1.5)
```

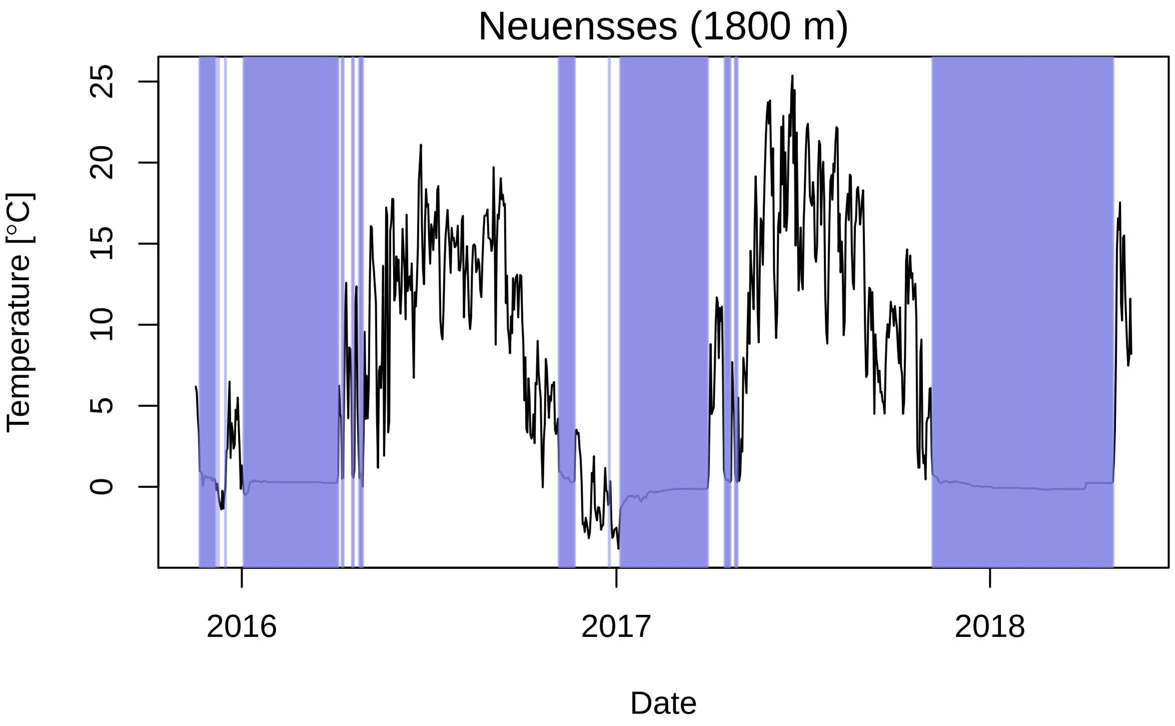


```r
rm(neuhobo)


## Padora
padhobo <- subset(hobos, site == "Pad")
padsnow <- padhobo$date[padhobo$tempvar < 1 & padhobo$temp < 1]

plot(temp~date, padhobo, type = "l",
     xlab = "Date",
     ylab = expression(paste("Temperature [",degree,"C]")))

mtext("Padora (1800 m)", line = 0.25, cex = 1.25)
abline(v = padsnow, col = rgb(0.5,0.5,0.9,0.5), lwd = 1.5)
```

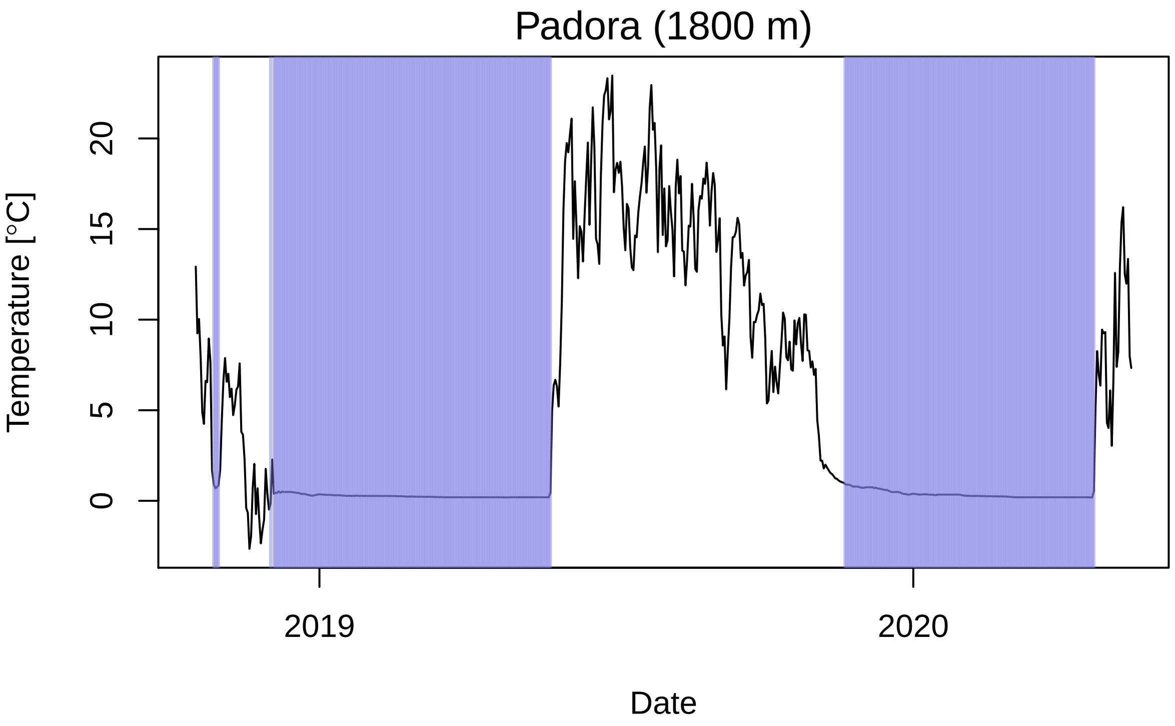


```
rm(padhobo)


## Calanda
calhobo <- subset(hobos, site == "Cal")
calsnow <- calhobo$date[calhobo$tempvar < 1 & calhobo$temp < 1]

plot(temp~date, calhobo, type = "l",
     xlab = "Date",
     ylab = expression(paste("Temperature [",degree,"C]")))

mtext("Calanda (2000 m)", line = 0.25, cex = 1.25)
abline(v = calsnow, col = rgb(0.5,0.5,0.9,0.5), lwd = 1.5)
```

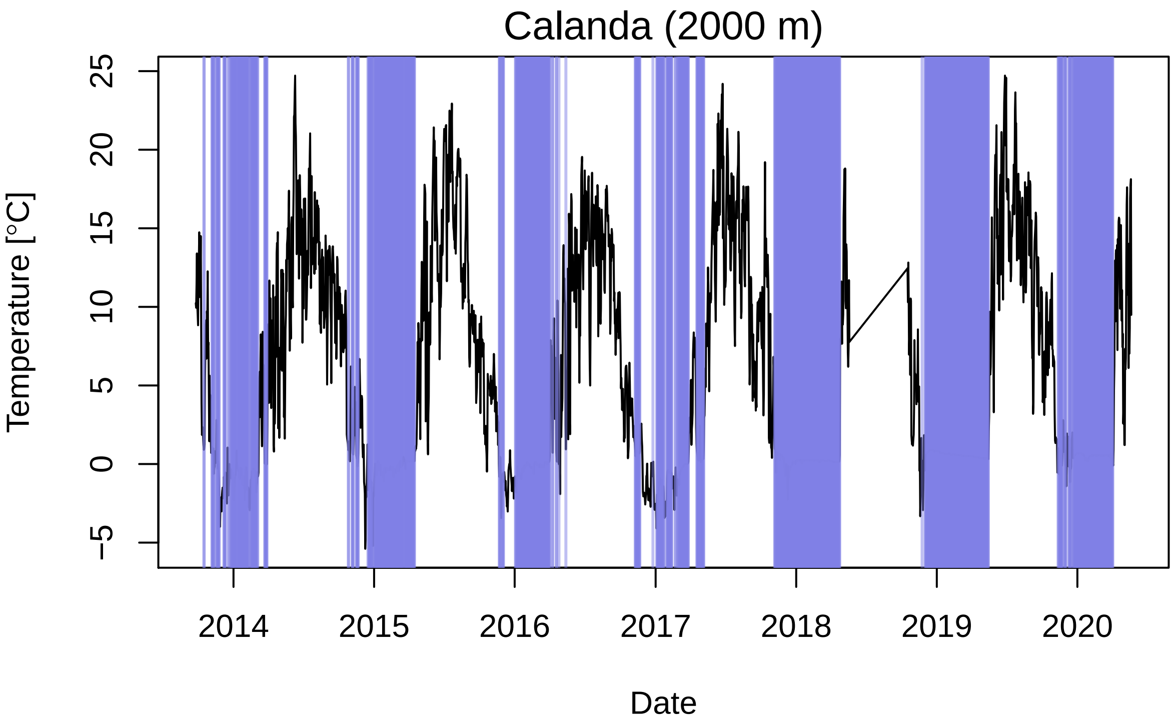


```
rm(calhobo)

rm(hobos)
```

```
for(spcode in sppList) {
        # Load data frames with ramet and demographic information
        fpath_d <- paste("processed-data/", spcode, "_DMD.csv", sep = "")
        d <- read_csv(fpath_d)

        fpath_r <- paste("processed-data/", spcode, "_ramets.csv", sep = "")
        ramets <- read_csv(fpath_r)

        # Create weather variables

        ## Summer mean temperature during the first year of the transition
        d$tempLS <- numeric(nrow(d))

        ## Mean temperature during the first summer of the transition and the year before
        d$tempL2S <- numeric(nrow(d))

        ## Mean soil moisture during the previous summer
        d$moistLS <- numeric(nrow(d))

        # Format date
        d$date <- as.character(d$date)
        d$date <- gsub("/", ".", d$date, fixed = T)
        d$date <- gsub("2016", "2017", d$date, fixed = T)

        d$date <- as.Date(d$date, format = "%d.%m.%Y")
        #d$date <- as.Date(d$date, format = "%Y-%m-%d")
```

```r
#summary(d$date)
d$site <- as.character(d$site)

for ( i in 1:nrow(d)) {

        if(d$year[i] == 2017){
                grid.i <- strsplit(subset(ramets, ramet_id == d$ramet[i])$id_17,
                        ".", fixed = T)[[1]][3]
                LS <- subset(wdat, date >= "2017-06-01" & date < "2017-09-01")
                LLS <- subset(wdat, date >= "2016-06-01" & date < "2016-09-01")
        }

        if(d$year[i] == 2018){
                grid.i <- strsplit(subset(ramets, ramet_id == d$ramet[i])$id_18,
                                   ".", fixed = T)[[1]][3]
                LS <- subset(wdat, date >= "2018-06-01" & date < "2018-09-01")
                LLS <- subset(wdat, date >= "2017-06-01" & date < "2017-09-01")
        }

        if(d$year[i] == 2019){
                grid.i <- strsplit(subset(ramets, ramet_id == d$ramet[i])$id_19,
                                   ".", fixed = T)[[1]][3]
                LS <- subset(wdat, date >= "2019-06-01" & date < "2019-09-01")
                LLS <- subset(wdat, date >= "2018-06-01" & date < "2018-09-01")
        }


        if(d$site[i] == "Are") {

                # Last summer
                LS <- LS[!(LS$date %in% aresnow),]
                LLS <- LLS[!(LLS$date %in% aresnow),]

                ## Summer temperature during the first year of the transition
                d$tempLS[i] <- mean(LS$are_predtemp)

                if (d$year[i] == 2017) {
                        d$tempL2S[i] <- mean(c(mean(LS$are_predtemp),
                                            mean(LLS$cal_predtemp)))
                } else {
                        d$tempL2S[i] <- mean(c(mean(LS$are_predtemp),
                                            mean(LLS$are_predtemp)))
                }

                ## Summer mean moisture during the first year of the transition
                d$moistLS[i] <- mean(LS$are_predmoist, na.rm = T)
        }


        if(d$site[i] == "Nes") {

                # Last summer
                LS <- LS[!(LS$date %in% nessnow),]
```

```
        LLS <- LLS[!(LLS$date %in% nessnow),]

        ## Summer  temperature during the first year of the transition
        d$tempLS[i] <- mean(LS$nes_predtemp)

        if (d$year[i] == 2017) {
                d$tempL2S[i] <- mean(c(mean(LS$nes_predtemp),
                                       mean(LLS$cal_predtemp)))
        } else {
                d$tempL2S[i] <- mean(c(mean(LS$nes_predtemp),
                                       mean(LLS$nes_predtemp)))
        }

        ## Summer mean moisture during the first year of the transition
        d$moistLS[i] <- mean(LS$nes_predmoist)

}

if(d$site[i] == "Bar") {

        # Last summer
        LS <- LS[!(LS$date %in% barsnow),]
        LLS <- LLS[!(LLS$date %in% barsnow),]

        ## Summer  temperature during the first year of the transition
        d$tempLS[i] <- mean(LS$bar_predtemp)

        if (d$year[i] == 2017) {
                d$tempL2S[i] <- mean(c(mean(LS$bar_predtemp),
                                       mean(LLS$cal_predtemp)))
        } else {
                d$tempL2S[i] <- mean(c(mean(LS$bar_predtemp),
                                       mean(LLS$bar_predtemp)))
        }

        ## Summer mean moisture during the first year of the transition
        d$moistLS[i] <- mean(LS$bar_predmoist)

}


if(d$site[i] == "Pad") {

        # Last summer
        LS <- LS[!(LS$date %in% padsnow),]
        LLS <- LLS[!(LLS$date %in% padsnow),]

        ## Summer  temperature during the first year of the transition
        d$tempLS[i] <- mean(LS$pad_predtemp)
        d$tempL2S[i] <- mean(c(mean(LS$pad_predtemp),
                               mean(LLS$cal_predtemp)))

        ## Summer mean moisture during the first year of the transition
```

```
                        d$moistLS[i] <- mean(LS$pad_predmoist)

                }

                if(d$site[i] == "Cal") {

                        # Last summer
                        LS <- LS[!(LS$date %in% calsnow),]
                        LLS <- LLS[!(LLS$date %in% calsnow),]

                        ## Summer temperature during the first year of the transition
                        d$tempLS[i] <- mean(LS$cal_predtemp)
                        d$tempL2S[i] <- mean(c(mean(LS$cal_predtemp),
                                               mean(LLS$cal_predtemp)))
                        ## Summer mean moisture during the first year of the transition
                        d$moistLS[i] <- mean(LS$cal_predmoist)

                }

        }

        write_csv(d, fpath_d)
}
```

With these tables summarizing climatic and demographic data for ramets of different taxa in our experiment, we are ready to fit statistical models of survival and growth.

1.4.2 Recruitment

In each of the 25 cm^2 grid cells unoccupied by a taxa, we modeled the probability of a new ramet emerging there the following year as a function of crowding by conspecific and heterospecific neighbors. We built tables storing this and other information for each unoccupied grid cell in each annual transition.

```
for (sp in domsp){

        fpath_d <- paste("processed-data/",
                         sppList[which(sp==domsp)],
                         "_DMD.csv", sep = "")
        d <- read_csv(fpath_d)
        fpath_r <- paste("processed-data/",
                         sppList[which(sp==domsp)],
                         "_ramets.csv", sep = "")
        ramets <- read_csv(fpath_r)

        sp17 <- subset(anbc, species == sp & year == 2017)
        sp18 <- subset(anbc, species == sp & year == 2018)
        sp19 <- subset(anbc, species == sp & year == 2019)

        if(sppList[which(sp==domsp)] == "Alcxan") {
            ramets$id_17 <- gsub("Alchemilla xanthochlora pratensis", sp, ramets$id_17)
            ramets$id_18 <- gsub("Alchemilla xanthochlora pratensis", sp, ramets$id_18)
            ramets$id_19 <- gsub("Alchemilla xanthochlora pratensis", sp, ramets$id_19)
            ramets$id_20 <- gsub("Alchemilla xanthochlora pratensis", sp, ramets$id_20)
```

```r
}

# Make list of all quadrants
allquads <- character()

for (grid in c("AL", "BE", "GA", "DE")) {
    for (row in c("A", "B", "C", "D", "E")){
        for (col in 1:5){
            for (quad in 1:4){
                newq <-  paste(grid, ".", row, col, ".", quad, sep = "")
                allquads <- c(allquads, newq)
                rm(newq)
            }
        }
    }
}
rm(grid, row, col, quad)

d <- d[!(grepl("Neu", d$plot, fixed = T)),]

plots_sp <- levels(factor(d$plot))
allquads_sp <- character()

for (p in plots_sp) {
        newpqs <- paste(p, allquads, sep = ".")
        allquads_sp <- c(allquads_sp, newpqs)
        rm(newpqs)
}

# Remove non-existent quadrants
allquads_sp <- allquads_sp[allquads_sp %in% levels(factor(anbc$quadrant_id))]


# Prepare data for recruitment model
# 1: Prepare data
##   First transition

## Filter quadrants that were "open" for recruitment in 2017
closedqs17 <- ramets$id_17
closedqs17 <- closedqs17[!is.na(closedqs17)]

spyear <- paste(sp, 2017, sep = "")
closedqs17 <- gsub(spyear, "", closedqs17)
rm(spyear)

openqs17 <- allquads_sp[!(allquads_sp %in% closedqs17)]

# Filter "open" quadrants out of the neighborhood of occupied quadrants
# and to which no old ramet was tracked
survivors <- ramets$id_18[!is.na(ramets$id_17) & !is.na(ramets$id_18)]

spyear <- paste(sp, 2017+1, sep = "")
survivors <- gsub(spyear, "", survivors)
```

```r
rquads17 <- openqs17[!(openqs17 %in% survivors)]

isPad <- logical(length(rquads17))

for(i in 1:length(rquads17)) {

        isPad[i] <- strsplit(rquads17[i], "[0-1]")[[1]][1] %in% "Pad"
}

rquads17 <- rquads17[!isPad]

recs18 <- ramets$id_18[!is.na(ramets$id_18) & is.na(ramets$id_17)]
recs18 <- gsub(spyear, "", recs18)

rm(spyear, isPad)


###  a: calculate quadrant-level recruitment
r17 <- data.frame(quadrant = rquads17,
                  recruitment = ifelse(rquads17 %in% recs18, 1, 0))

### Overall recruitment rate
sum(r17$recruitment)/nrow(r17)

## b: add predictor variables
r17$site <- character(nrow(r17))
r17$plot <- character(nrow(r17))
r17$x <- NA
r17$y <- NA
r17$year <- 2017

for(i in 1:nrow(r17)) {

        x <- subset(anbc, quadrant_id == r17$quadrant[i])

        r17$site[i] <- x$site[1]
        r17$plot[i] <- x$plot_id[1]
        r17$x[i] <- x$x[1]
        r17$y[i] <- x$y[1]
        rm(x)
}


r17$quadrant <- as.character(r17$quadrant)

spturfs <- levels(factor(subset(anbc, species == sp & year == 2017)$plot_id))
r17 <- r17[r17$plot %in% spturfs,]
rm(spturfs)

## Quantify crowding (w)

# An interaction radius of ca. 15 cm seems reasonable for starters
delta <- 0.02
```

```
rnd <- matrix(0,nrow = nrow(r17), ncol = 399)
rnc <- matrix(0,nrow = nrow(r17), ncol = 399)

for (i in 1:nrow(r17)) {

        x <- subset(sp17, plot_id == r17$plot[i])

        if(nrow(x) > 0){
                rnd[i,1:nrow(x)] <- sqrt((r17$x[i] - x$x)^2 + (r17$y[i] - x$y)^2)
                rnc[i,1:nrow(x)] <- x$cover_cm2
        }

        rm(x)
}

rnd[is.na(rnd)] <- 0
rnd2 <- rnd^2

# Quantify intraspecific crowding (w)
r17$w <- numeric(nrow(r17))

for (i in 1:nrow(r17)){
        r17$w[i] <- sum(exp(-delta*rnd2[i,])*rnc[i,])
}

rm(rnc, rnd, rnd2)

##
# Interspecific community crowding
maxncol = max(table(subset(anbc, year == 2017 & species != sp)$plot_id))
rnd.c <- matrix(0,nrow = nrow(r17), ncol = maxncol)
rnc.c <- matrix(0,nrow = nrow(r17), ncol = maxncol)

for (i in 1:nrow(r17)) {

    n.ci <- subset(anbc, plot_id == r17$plot[i] & year == 2017 & species != sp)

    rnd.c[i,1:nrow(n.ci)] <- sqrt((r17$x[i] - n.ci$x)^2 + (r17$y[i] - n.ci$y)^2)
    rnc.c[i,1:nrow(n.ci)] <- n.ci$cover_cm2

    rm(n.ci)
}

rnd.c[is.na(rnd.c)] <- 0
rnd2.c <- rnd.c^2

# Quantify interspecific crowding (w.cinter)
r17$w.cinter <- numeric(nrow(r17))

for (i in 1:nrow(r17)){
        r17$w.cinter[i] <- sum(exp(-delta*rnd2.c[i,])*rnc.c[i,])
}
```

```
rm(rnd.c, rnd2.c, rnc.c)
summary(r17$w.cinter)

#
##   Second transition

## Filter quadrants that were "open" for recruitment in 2018
closedqs18 <- ramets$id_18
closedqs18 <- closedqs18[!is.na(closedqs18)]

spyear <- paste(sp, 2018, sep = "")
closedqs18 <- gsub(spyear, "", closedqs18)
rm(spyear)

openqs18 <- allquads_sp[!(allquads_sp %in% closedqs18)]

# Filter "open" quadrants out of the neighborhood of occupied quadrants
# and to which no old ramet was tracked
survivors <- ramets$id_19[!is.na(ramets$id_18) & !is.na(ramets$id_19)]

spyear <- paste(sp, 2018+1, sep = "")
survivors <- gsub(spyear, "", survivors)


rquads18 <- openqs18[!(openqs18 %in% survivors)]

isPad <- logical(length(rquads18))

for(i in 1:length(rquads18)) {

        isPad[i] <- strsplit(rquads18[i], "[0-1]")[[1]][1] %in% c("Pad")
}

rquads18 <- rquads18[!isPad]

recs19 <- ramets$id_19[!is.na(ramets$id_19) & is.na(ramets$id_18)]
recs19 <- gsub(spyear, "", recs19)

rm(spyear, isPad)


###  a: calculate quadrant-level recruitment
r18 <- data.frame(quadrant = rquads18,
                  recruitment = ifelse(rquads18 %in% recs19, 1, 0))

### Overall recruitment rate
sum(r18$recruitment)/nrow(r18)

## b: add predictor variables
r18$site <- character(nrow(r18))
r18$plot <- character(nrow(r18))
r18$x <- NA
r18$y <- NA
```

```r
r18$year <- 2018


for(i in 1:nrow(r18)) {

        x <- subset(anbc, quadrant_id == r18$quadrant[i])

        r18$site[i] <- x$site[1]
        r18$plot[i] <- x$plot_id[1]
        r18$x[i] <- x$x[1]
        r18$y[i] <- x$y[1]
        rm(x)
}


r18$quadrant <- as.character(r18$quadrant)

spturfs <- levels(factor(subset(anbc, species == sp & year == 2018)$plot_id))
r18 <- r18[r18$plot %in% spturfs,]
rm(spturfs)

## Quantify crowding (w)

# An interaction radius of ca. 15 cm seems reasonable for starters
delta <- 0.02

rnd <- matrix(0,nrow = nrow(r18), ncol = 399)
rnc <- matrix(0,nrow = nrow(r18), ncol = 399)

for (i in 1:nrow(r18)) {

        x <- subset(sp18, plot_id == r18$plot[i])

        if(nrow(x) > 0){
                rnd[i,1:nrow(x)] <- sqrt((r18$x[i] - x$x)^2 + (r18$y[i] - x$y)^2)
                rnc[i,1:nrow(x)] <- x$cover_cm2
        }

        rm(x)
}

rnd[is.na(rnd)] <- 0
rnd2 <- rnd^2

# Quantify intraspecific crowding (w)
r18$w <- numeric(nrow(r18))

for (i in 1:nrow(r18)){
        r18$w[i] <- sum(exp(-delta*rnd2[i,])*rnc[i,])
}

rm(rnc, rnd, rnd2)
```

```r
# Interspecific community crowding
maxncol = max(table(subset(anbc, year == 2018 & species != sp)$plot_id))
rnd.c <- matrix(0,nrow = nrow(r18), ncol = maxncol)
rnc.c <- matrix(0,nrow = nrow(r18), ncol = maxncol)

for (i in 1:nrow(r18)) {

    n.ci <- subset(anbc, plot_id == r18$plot[i] & year == 2018 & species != sp)

    rnd.c[i,1:nrow(n.ci)] <- sqrt((r18$x[i] - n.ci$x)^2 + (r18$y[i] - n.ci$y)^2)
    rnc.c[i,1:nrow(n.ci)] <- n.ci$cover_cm2

    rm(n.ci)
}

rnd.c[is.na(rnd.c)] <- 0
rnd2.c <- rnd.c^2

# Quantify interspecific crowding (w.c)
r18$w.cinter <- numeric(nrow(r18))

for (i in 1:nrow(r18)){
        r18$w.cinter[i] <- sum(exp(-delta*rnd2.c[i,])*rnc.c[i,])
}

rm(rnd.c, rnd2.c, rnc.c)
summary(r18$w.cinter)

####
##   Third transition

## Filter quadrants that were "open" for recruitment in 2019
closedqs19 <- ramets$id_19
closedqs19 <- closedqs19[!is.na(closedqs19)]

spyear <- paste(sp, 2019, sep = "")
closedqs19 <- gsub(spyear, "", closedqs19)
rm(spyear)

openqs19 <- allquads_sp[!(allquads_sp %in% closedqs19)]

# Filter "open" quadrants out of the neighborhood of occupied quadrants
# and to which no old ramet was tracked
survivors <- ramets$id_20[!is.na(ramets$id_19) & !is.na(ramets$id_20)]

spyear <- paste(sp, 2019+1, sep = "")
survivors <- gsub(spyear, "", survivors)


rquads19 <- openqs19[!(openqs19 %in% survivors)]

recs20 <- ramets$id_20[!is.na(ramets$id_20) & is.na(ramets$id_19)]
recs20 <- gsub(spyear, "", recs20)
```

```r
rm(spyear, isPad)


###  a: calculate quadrant-level recruitment
r19 <- data.frame(quadrant = rquads19,
                  recruitment = ifelse(rquads19 %in% recs20, 1, 0))

### Overall recruitment rate
sum(r19$recruitment)/nrow(r19)

## b: add predictor variables
r19$site <- character(nrow(r19))
r19$plot <- character(nrow(r19))
r19$x <- NA
r19$y <- NA
r19$year <- 2019
#r19$date <- character(nrow(r19))


for(i in 1:nrow(r19)) {

        x <- subset(anbc, quadrant_id == r19$quadrant[i])

        r19$site[i] <- x$site[1]
        r19$plot[i] <- x$plot_id[1]
        r19$x[i] <- x$x[1]
        r19$y[i] <- x$y[1]
        rm(x)
}

r19$quadrant <- as.character(r19$quadrant)

spturfs <- levels(factor(subset(anbc, species == sp & year == 2019)$plot_id))
r19 <- r19[r19$plot %in% spturfs,]
rm(spturfs)

## Quantify crowding (w)

# An interaction radius of ca. 15 cm seems reasonable for starters
delta <- 0.02

rnd <- matrix(0,nrow = nrow(r19), ncol = 399)
rnc <- matrix(0,nrow = nrow(r19), ncol = 399)

for (i in 1:nrow(r19)) {

        x <- subset(sp19, plot_id == r19$plot[i])

        if(nrow(x) > 0){
                rnd[i,1:nrow(x)] <- sqrt((r19$x[i] - x$x)^2 + (r19$y[i] - x$y)^2)
                rnc[i,1:nrow(x)] <- x$cover_cm2
        }
```

```
        rm(x)
}

rnd[is.na(rnd)] <- 0
rnd2 <- rnd^2

# Quantify intraspecific crowding (w)
r19$w <- numeric(nrow(r19))

for (i in 1:nrow(r19)){
        r19$w[i] <- sum(exp(-delta*rnd2[i,])*rnc[i,])
}

rm(rnc, rnd, rnd2)


# Interspecific community crowding
maxncol = max(table(subset(anbc, year == 2019 & species != sp)$plot_id))
rnd.c <- matrix(0,nrow = nrow(r19), ncol = maxncol)
rnc.c <- matrix(0,nrow = nrow(r19), ncol = maxncol)

for (i in 1:nrow(r19)) {

        n.ci <- subset(anbc, plot_id == r19$plot[i] &
                              year == 2019 & species != sp)

        rnd.c[i,1:nrow(n.ci)] <- sqrt((r19$x[i]-n.ci$x)^2+(r19$y[i]-n.ci$y)^2)
        rnc.c[i,1:nrow(n.ci)] <- n.ci$cover_cm2

        rm(n.ci)
}

rnd.c[is.na(rnd.c)] <- 0
rnd2.c <- rnd.c^2

# Quantify interspecific crowding (w.c)
r19$w.cinter <- numeric(nrow(r19))

for (i in 1:nrow(r19)){
        r19$w.cinter[i] <- sum(exp(-delta*rnd2.c[i,])*rnc.c[i,])
}

rm(rnd.c, rnd2.c, rnc.c)
summary(r19$w.cinter)

###
##   COMBINE DATA
###
r <- rbind(r17,r18,r19)

r$sid <- ifelse(r$site == "Are", 1,
                ifelse(r$site == "Nes", 2,
                       ifelse(r$site == "Bar", 3,
```

```
                                      ifelse(r$site == "Pad", 4, 5))))


        r$pid <- as.numeric(as.factor(r$plot))

        summary(r)

        # Exclude quadrants from plots in which the species went extinct (w = 0)
        r <- r[r$w != 0, ]

        datafp <- paste("processed-data/", sppList[which(sp==domsp)], "_RD.csv",
                        sep = "")
        write_csv(r, datafp)

}
```

Let's also associate each empty quadrant with the climatic conditions at the site during the previous summer.

```
for (spcode in sppList){
        fpath_d <- paste("processed-data/", spcode, "_DMD.csv", sep = "")
        d <- read_csv(fpath_d)
        fpath_r <- paste("processed-data/", spcode, "_RD.csv", sep = "")
        r <- read_csv(fpath_r)

        # Mean temperature during first summer
        r$tempLS <- numeric(nrow(r))

        # Mean soil moisture during first summer
        r$moistLS <- numeric(nrow(r))

        for ( i in 1:nrow(r)) {

                x <- subset(d, year == r$year[i] & plot == r$plot[i])
                # Mean temperature during first summer
                r$tempLS[i] <- mean(x$tempLS)
                # Mean soil moisture during the previous summer
                r$moistLS[i] <- mean(x$moistLS)

                rm(x)
        }



        write_csv(r, fpath_r)
}
```

2. Statistical models of plant demography

2.1 Survival

We did a prior predictive simulation to select prior parameter distributions encompassing a reasonably wide range of relationships between demography, temperature, and neighborhood crowding.

With the code below, one can visualize the effect that the assumed prior variation in the parameters has on survival probability. That is, we can visualize the demographic consequences of our prior assumptions about the likelihood of different parameter values.

```
### Prior predictive simulation

# Define temperature range (scaled units)
temprange <- seq(-2, 2, length.out = 1e3)

# Define mean intra and interspecific crowding
w = 10
wC = 160

# Plot space
plot(temprange, temprange,
     type = 'n', ylim = c(0,1),
     ylab = "Survival probability",
     xlab = "Temperature")

for(i in 1:1e3){

        tef <- 6#rnorm(1, 6, 2) # Logodds survival temp-independent
        k <- rnorm(1, 0, 2) # Steepness of logistic response
        ttp_a <- 0#rnorm(1, 0, 2.5) # Thermal optimum (scaled units)
        ttp_b <- 0#rnorm(1, 0, 0.5)

        aC_0 <- 0#rnorm(1, 0, 0.1) # intercept (i.e., value at mean temperature)
        b_aC <- 0#rnorm(1, 0, 0.05) # response to temperature
        aC <- aC_0 + b_aC*temprange

        a_0 <- 0#rnorm(1, 0, 0.1) # intercept (i.e., value at mean temperature)
        b_a <- 0#rnorm(1, 0, 0.05) # response to temperature
        a <- a_0 + b_a*temprange

        rT <- tef/(1 + exp(-k*(temprange - ttp_a))) - 4
        logodds <- rT + aC*wC + a*w


        lines(temprange, inv_logit(logodds), col = col.alpha('black'))


}
```

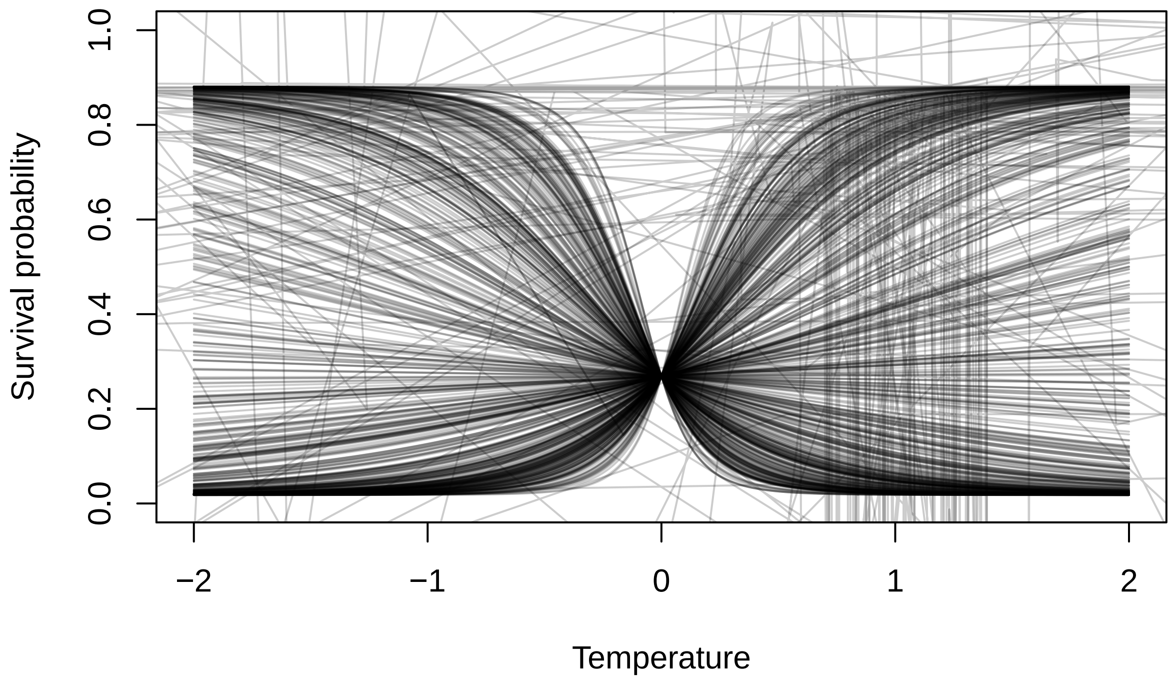

Figure S2.15 Prior predictive simulation showing effects of variation in parameter k.

We also tested the whether models with these priors could recover 'true' parameter values from simulated data (see Supporting Material X).

We modeled survival ramet i ('S_i') as follows.

$$S_i \sim Bernoulli(s_i)$$

$$logit(s_i) = \lambda^S(T_{LS}) + b^S * u + \alpha^S_{i,i}(T_{LS}) * w_i + \alpha^S_{i,j}(T_{LS}) * w_j + \theta$$

$$\lambda^S = \frac{L}{1 + exp(-k * (T_{LS} - h))}$$

$$L \sim Normal(6, 2)$$

$$k \sim Normal(0, 2)$$

$$h = h_a + h_b * M_{LS}$$

$$h_a \sim Normal(0, 2.5)$$

$$h_b \sim Normal(0, 0.5)$$

$$b \sim Normal(0, 0.5)$$

$$\alpha^S_{i,i} = a^S_{i,i} + b^S_{i,i} * T_{LS}$$

$$a^S_{i,i} \sim Normal(0, 0.1)$$

$$b^S_{i,i} \sim Normal(0, 0.05)$$

$$\alpha^S_{i,j} = a^S_{i,j} + b^S_{i,j} * T_{LS}$$

$$a^S_{i,j} \sim Normal(0, 0.1)$$

$$b^S_{i,j} \sim Normal(0, 0.05)$$

We used the value '$\theta = -4$' to guarantee that the survival probability of a mean-sized ramet in the absence of neighbors was low (i.e., 0.02) when 'T_{LS}' was far beyond the threshold represented by parameter 'h'.

With the code below, we fitted survival models for the 31 taxa with more than 1500 observations across sites and years. Divergent transitions arose during sampling for the models of some taxa, such as *Luzula* spp. and *Phyteuma orbiculare*. Since, during simulation analyses, we sometimes observed divergent transitions arising during samples even in cases when the models successfully recovered true parameter values, we tested the models with a *a posteriori* simulation analysis. That is, a simulation analysis in which the 'true' parameter values are the mean of the values sampled by the model. Like this, we tested whether, if those where indeed the true parameter values, the model would be able to estimate them from the observed data. If models in which divergent transition arose did not pass this test (see *a posteriori* simulation tests in section XI of Supporting Material), they were excluded from the analyses.

```
for (spcode in sppList) {

        # Loading and filtering demographic data
        fpath_d <- paste("processed-data/", spcode, "_DMD.csv", sep = "")
        d <- read.csv(fpath_d)

        # Keep only ramets in the center of the plot
        d <- subset(d, x > 20 & x < 80 & y > 20 & y < 80)

        # Exclude ramets in grids with collars
        ## Alpha grids in Calanda plots
        d <- d[!(d$site == "Cal" & d$x < 50 & d$y > 50), ]

        ## Alpha or gamma grids in Nes plots
        collarsG = c('Nes01.03', 'Nes02.03', 'Nes04.03', 'Nes05.01',
                     'Nes08.01', 'Nes09.03') # Nes plots with colars in grid Gamma
        collarsA = c('Nes03.01', 'Nes06.03', 'Nes07.03',
                     'Nes10.01') # Nes plots with colars in grid Alpha

        d <- d[!(d$plot %in% collarsA & d$x < 50 & d$y > 50), ]
        d <- d[!(d$plot %in% collarsG & d$x < 50 & d$y < 50), ]

        # Exclude ramets that affected by disturbance
        comments <- read.csv('processed-data/comments-and-voles(04062021).csv')
```

```
disturbances = subset(comments,
                      type %in% c('sensor', 'vole') &
                      level %in% c('cell', 'quadrant'))

disturbances$year[is.na(disturbances$year)] =
        substr(as.character(disturbances$date[is.na(disturbances$year)]), 1, 4)

for (i in 1:nrow(disturbances)) {

        if (disturbances$level[i] == 'quadrant') {

                quadid = as.character(disturbances$quadrant_id[i])
                plotid = substr(quadid, 1, 8)
                gridid = substr(quadid, 10, 11)
                rowid = substr(quadid, 13, 13)
                colid = substr(quadid, 14, 14)
                qid = as.numeric(substr(quadid, 16, 16))

                # get quad coordinates
                xi = ifelse(gridid %in% c('BE', 'DE'), 50, 0)
                yi = ifelse(gridid %in% c('BE', 'AL'), 50, 0)

                xi = xi + as.numeric(colid)*10 - 5
                yi = yi + ifelse(rowid == "A", 45,
                                 ifelse(rowid == "B", 35,
                                        ifelse(rowid == "C", 25,
                                                ifelse(rowid == "D", 15, 5))))

                xi = xi + ifelse(qid %in% c(1,3), -2.5, 2.5)
                yi = yi + ifelse(qid %in% c(3,4), -2.5, 2.5)

                rmi = which(d$plot == plotid &
                                    abs(d$x - xi) < 3 &
                                    abs(d$y - yi) < 3 &
                                    d$survival == 0 &
                                    d$year + 1 == disturbances$year[i])

                if (length(rmi)>0) d = d[-rmi, ]

                 rm(rmi, xi, yi, quadid, plotid, gridid, rowid, colid, qid)

        }


        if (disturbances$level[i] == 'cell') {

                cellid = as.character(disturbances$cell_id[i])
                plotid = substr(cellid, 1, 8)
                gridid = substr(cellid, 10, 11)
                rowid = substr(cellid, 13, 13)
                colid = substr(cellid, 14, 14)

                # get cell coordinates
```

```
                xi = ifelse(gridid %in% c('BE', 'DE'), 50, 0)
                yi = ifelse(gridid %in% c('BE', 'AL'), 50, 0)

                xi = xi + as.numeric(colid)*10 - 5
                yi = yi + ifelse(rowid == "A", 45,
                        ifelse(rowid == "B", 35,
                             ifelse(rowid == "C", 25,
                                 ifelse(rowid == "D", 15, 5))))

                rmi = which(d$plot == plotid &
                          abs(d$x - xi) < 5 &
                          abs(d$y - yi) < 5 &
                          d$survival == 0 &
                          d$year + 1 == disturbances$year[i])

                if (length(rmi)>0) d = d[-rmi, ]

                rm(rmi, xi, yi, cellid, plotid, gridid, rowid, colid)
        }

}

        # Data for model
        d_list_sm_raw <- list(
                        survival = d$survival,
                        u = (d$u1 - mean(d$u1))/sd(d$u1),
                        templS = (d$templS - mean(d$templS))/
                            sd(d$templS),
                        moistLS = (d$moistLS - mean(d$moistLS))/
                            sd(d$moistLS),
                        w = d$w,
                        wC = d$w.cinter
                        )

        # Initial values
        initVals <- list(list(ttp_a = 0,
                      ttp_b = 0.25,
                      b_a = 0,
                      b_aC = 0,
                      a_0 = 0,
                      aC_0 = 0,
                      k = -2,
                      tef = 8),
                 list(ttp_a = 0,
                      ttp_b = 0.25,
                      b_a = 0,
                      b_aC = 0,
                      a_0 = 0,
                      aC_0 = 0,
                      k = -2,
                      tef = 8),
                 list(ttp_a = 0,
                      ttp_b = 0.25,
```

```
                                b_a = 0,
                                b_aC = 0,
                                a_0 = 0,
                                aC_0 = 0,
                                k = -2,
                                tef = 8),
                           list(ttp_a = 0,
                                ttp_b = 0.25,
                                b_a = 0,
                                b_aC = 0,
                                a_0 = 0,
                                aC_0 = 0,
                                k = -2,
                                tef = 8)
                           )

                # Sample posterior
                smod <- ulam(
                                 alist(
                                   survival ~ dbern(p),
                                   logit(p) <- -4 + tempR + b*u + a*w + aC*wC,
                                   tempR <- tef/(1 + exp(-k*(tempLS - ttp))),
                                   tef ~ dnorm(6, 2),
                                   k ~ dnorm(0,2),
                                   ttp <- ttp_a + ttp_b * moistLS,
                                   ttp_a ~ dnorm(0,2.5),
                                   ttp_b ~ dnorm(0,0.5),
                                   b ~ dnorm(0, 0.5),
                                   a <- a_0 + b_a * tempLS,
                                   a_0 ~ dnorm(0, 0.1),
                                   b_a ~ dnorm(0, 0.05),
                                   aC <- aC_0 + b_aC * tempLS,
                                   aC_0 ~ dnorm(0, 0.1),
                                   b_aC ~ dnorm(0, 0.05)
                                 ),
                                 data = d_list_sm_raw, chains = 4, cores = 4,
                                 iter = 5500, warmup = 500,
                                 log_lik = F, init = initVals,
                                 control=list(adapt_delta=0.97))

                # Save model fit
                smpath <- paste("model-fits/", spcode,"_smod.rds", sep = "")
                saveRDS(smod, smpath)

}
```

2.2 Growth

As with the survival model, we started by doing a prior predictive simulation to guide our selection of prior parameter distributions.

Let's visualize the demographic consequences of our prior assumptions about the likelihood of different parameter values.

```r
### Prior predictive simulation

# Define temperature range (scaled units)
temprange <- seq(-2, 2, length.out = 1e3)

# Define mean intra and interspecific crowding
w = 10
wC = 160

u1max = 6.25
u1min = 3.125

# Plot space
plot(temprange, temprange,
     type = 'n', ylim = c(0,3),
     ylab = "Growth rate",
     xlab = "Temperature")

for(i in 1:1e3){

        tef <- rexp(1, 1) # Logodds survival temp-independent #6#
        tsd <- rexp(1, 0.5) # Steepness of logistic response
        ttp <- rnorm(1, 0, 1.5) # Thermal optimum (scaled units) #0#


        aC_0 <- rnorm(1, -6, 0.5) # intercept (i.e., value at mean temperature) #0#
        b_aC <- rnorm(1, 0, 0.25) # response to temperature #0#
        aC <- exp(aC_0 + b_aC*temprange)

        a_0 <- rnorm(1, -6, 0.5) # intercept (i.e., value at mean temperature) #0#
        b_a <- rnorm(1, 0, 0.25) # response to temperature #0#
        a <- exp(a_0 + b_a*temprange)

        rT <- tef*exp(-((temprange - ttp)^2/2*tsd^2))

        b0 <- rnorm(1, -0.7, 0.2) # -0.7 #

        b <-  rnorm(1, 0, 0.05) # 0 #

        u1 <- u1max - (u1max-u1min)/2 # dnorm((u1max - (u1max-u1min)/2, u1max/10))

        mu <- b0 + rT + log(u1)*b - aC*wC - a*w

        lines(temprange, exp(mu), col = col.alpha('black'))

}
```

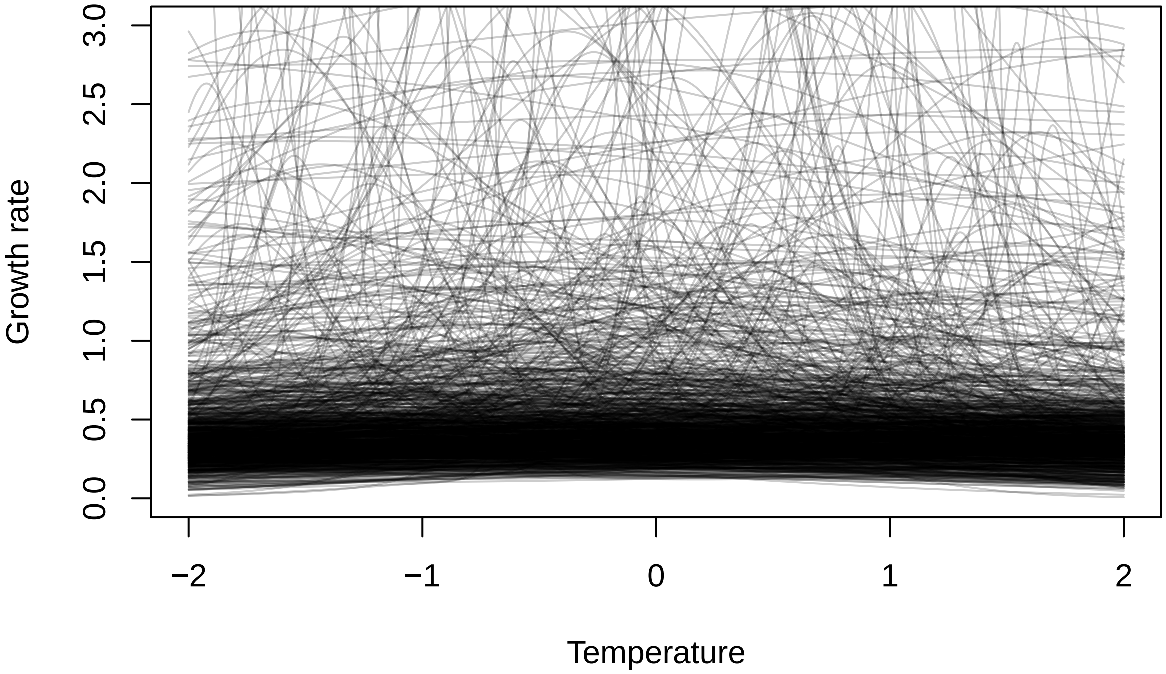

Figure S2.16 Prior predictive simulation showing the range of potential relationships between growth rates and temperature given the assumed prior distribution of the model parameters.

In general, the growth model was able to recover 'true' parameter values from simulated data (see Supporting Material X).

We modeled the growth rate ('$\frac{u_1}{u_2}$') ramet i ('g_i') as follows.

$$log(g_i) \sim Normal(\mu_i, \sigma)$$

$$g_i = b_0 + \lambda^G(T_{L2S}) + b^G * log(u_1) + \alpha^G_{i,i}(T_{LS}) * w_i + \alpha^G_{i,j}(T_{LS}) * w_j + \theta$$

$$\lambda^G = r_{max} * exp(\frac{-(T_{L2S} - T_{opt})^2}{2\sigma_T^2})$$

$$r_{max} \sim Exponential(1)$$

$$T_{opt} \sim Normal(0, 1.5)$$

$$\sigma_T \sim Exponential(0.5)$$

$$b^G \sim Normal(0, 0.05)$$

$$b_0 \sim Normal(-0.7, 0.2)$$

$$\alpha^G_{i,i} = exp(a^G_{i,i} + b^G_{i,i} * T_{L2S})$$

$$a^G_{i,i} \sim Normal(-6, 0.5)$$

$$b^G_{i,i} \sim Normal(0, 0.25)$$

$$\alpha^G_{i,j} = exp(a^G_{i,j} + b^G_{i,j} * T_{L2S})$$

$$a^G_{i,j} \sim Normal(-6, 0.5)$$

$$b^G_{i,j} \sim Normal(0, 0.25)$$

$$\sigma \sim Exponential(1)$$

With the code below, we fitted growth models for the 31 taxa with more than 1500 observations across sites and years. Divergent transitions arose during sampling for the models of some taxa, such as *Leontodon* Group, *Hieracium lactucella*, and *Helianthemum alpestre*. Like with survival models, we tested the models in which divergent transitions arose with a simulation analysis *a posteriori*. That is, a simulation analysis in which the 'true' parameter values are the mean of the values sampled by the model. Like this, we tested whether the model would be able to re-estimate the parameters from the observed data, assuming those where indeed the true parameter values. If models in which divergent transition arose did not pass this test (see *a posteriori* simulation tests in section XI of Supporting Material), they were excluded from the analyses.

```
for (spcode in sppList){
        # Load demographic data
        fpath_d <- paste("processed-data/", spcode, "_DMD.csv", sep = "")
        d <- read.csv(fpath_d)

        # Keep only ramets in the center of the plot
        d <- subset(d, x > 20 & x < 80 & y > 20 & y < 80)
        table(d$site)

        # Exclude ramets in grids with collars
        ## Alpha grids in Calanda plots
        d <- d[!(d$site == "Cal" & d$x < 50 & d$y > 50), ]

        ## Alpha or gamma grids in Nes plots
        # Nes plots with colars in grid Gamma
        collarsG = c('Nes01.03', 'Nes02.03', 'Nes04.03',
                     'Nes05.01', 'Nes08.01', 'Nes09.03')
        # Nes plots with colars in grid Alpha
        collarsA = c('Nes03.01', 'Nes06.03',
                     'Nes07.03', 'Nes10.01')

        d <- d[!(d$plot %in% collarsA & d$x < 50 & d$y > 50), ]
        d <- d[!(d$plot %in% collarsG & d$x < 50 & d$y < 50), ]


        # Exclude ramets potentially affected by disturbance
        comments <- read.csv('comments-and-voles(04062021).csv')
        disturbances = subset(comments, type %in% c('sensor', 'vole') &
```

```
                              level %in% c('cell', 'quadrant'))

disturbances$year[is.na(disturbances$year)] =
        substr(as.character(disturbances$date[is.na(disturbances$year)]), 1, 4)

for (i in 1:nrow(disturbances)) {

  if (disturbances$level[i] == 'quadrant') {

    quadid = as.character(disturbances$quadrant_id[i])
    plotid = substr(quadid, 1, 8)
    gridid = substr(quadid, 10, 11)
    rowid = substr(quadid, 13, 13)
    colid = substr(quadid, 14, 14)
    qid = as.numeric(substr(quadid, 16, 16))

    # get quad coordinates
    xi = ifelse(gridid %in% c('BE', 'DE'), 50, 0)
    yi = ifelse(gridid %in% c('BE', 'AL'), 50, 0)

    xi = xi + as.numeric(colid)*10 - 5
    yi = yi + ifelse(rowid == "A", 45,
                     ifelse(rowid == "B", 35,
                            ifelse(rowid == "C", 25,
                                   ifelse(rowid == "D", 15, 5))))

    xi = xi + ifelse(qid %in% c(1,3), -2.5, 2.5)
    yi = yi + ifelse(qid %in% c(3,4), -2.5, 2.5)

    rmi = which(d$plot == plotid & abs(d$x - xi) < 3 &
                  abs(d$y - yi) < 3 & d$survival == 0 &
                  d$year + 1 == disturbances$year[i])

    if (length(rmi)>0) d = d[-rmi, ]

    rm(rmi, xi, yi, quadid, plotid, gridid, rowid, colid, qid)

  }


  if (disturbances$level[i] == 'cell') {

    cellid = as.character(disturbances$cell_id[i])
    plotid = substr(cellid, 1, 8)
    gridid = substr(cellid, 10, 11)
    rowid = substr(cellid, 13, 13)
    colid = substr(cellid, 14, 14)

    # get cell coordinates
    xi = ifelse(gridid %in% c('BE', 'DE'), 50, 0)
    yi = ifelse(gridid %in% c('BE', 'AL'), 50, 0)

    xi = xi + as.numeric(colid)*10 - 5
```

```
    yi = yi + ifelse(rowid == "A", 45,
                     ifelse(rowid == "B", 35,
                            ifelse(rowid == "C", 25,
                                   ifelse(rowid == "D", 15, 5))))

    rmi = which(d$plot == plotid & abs(d$x - xi) < 5 &
                       abs(d$y - yi) < 5 & d$survival == 0 &
                       d$year + 1 == disturbances$year[i])

    if (length(rmi)>0) d = d[-rmi, ]

    rm(rmi, xi, yi, cellid, plotid, gridid, rowid, colid)
  }

}

##
#  Growth model
##
####

# Filter survivors
dG <- d[complete.cases(d$u2),]

# Fix erroneous cover values
if(spcode != "FesGro"){
  dG$u1[dG$u1 < 1.5625] <- 1.5625
  dG$u2[dG$u2 < 1.5625] <- 1.5625
}

dG$u1[dG$u1 < 0.390625] <- 0.390625
dG$u1[dG$u1 > 0.390625 & dG$u1 < 1.5625] <- 1.5625
dG$u1[dG$u1 > 1.5625 & dG$u1 < 3.125] <- 3.125
dG$u1[dG$u1 > 3.125 & dG$u1 < 6.25] <- 6.25
dG$u1[dG$u1 > 6.25 & dG$u1 < 12.5] <- 12.5

dG$u2[dG$u2 < 0.390625] <- 0.390625


# Recorded cover categories are upper bounds
# Let's determine the lower bounds for each category
u2min <- ifelse(dG$u2 == 0.25, 0.1,
                ifelse(dG$u2 == 0.390625, 0.1,
                       ifelse(dG$u2 == 1.5625, 0.25,
                              ifelse(dG$u2 == 3.125, 1.56,
                                     ifelse(dG$u2 == 6.25, 3.125,
                                            ifelse(dG$u2 == 12.5, 6.25,
                                                   ifelse(dG$u2 == 18.75, 12.5,
                                                          ifelse(dG$u2 == 25,
                                                                 18.75,
                                                                 dG$u2 - 6.25)
                                                          )))))))
```

```
u1min <- ifelse(dG$u1 == 0.25, 0.1,
                ifelse(dG$u1 == 0.390625, 0.1,
                       ifelse(dG$u1 == 1.5625, 0.25,
                              ifelse(dG$u1 == 3.125, 1.56,
                                     ifelse(dG$u1 == 6.25, 3.125,
                                            ifelse(dG$u1 == 12.5, 6.25,
                                                   ifelse(dG$u1 == 18.75, 12.5,
                                                          ifelse(dG$u1 == 25,
                                                                 18.75,
                                                                 dG$u1 - 6.25)
                                                          )))))))


sum(is.na(c(u2min, u1min)))

rm(d)

### Interintra model
# Data for model
d_list_gm <- list(
  u2max = dG$u2,
  u1max = dG$u1,
  u2min = u2min,
  u1min = u1min,
  wC = dG$w.cinter,
  w = dG$w,
  temp = (dG$tempL2S - mean(dG$tempL2S))/sd(dG$tempL2S)
)

# Stan code for growth model
gmodcode = "data{
        vector[howmany] u2max;
        vector[howmany] u1max;
        vector[howmany] u2min;
        vector[howmany] u1min;
        vector[howmany] wC;
        vector[howmany] w;
        vector[howmany] temp;
}
parameters{
        real b0;
        real<lower=0> tef;
        real<lower=0> tsd;
        real ttp;
        real b;
        real a_0;
        real b_a;
        real aC_0;
        real b_aC;
        real<lower=0> sigma;
        vector[howmany] u2;
        vector[howmany] u1;
}
```

```
model{
        vector[howmany] mu;
        vector[howmany] g;
        vector[howmany] rT;
        vector[howmany] a;
        vector[howmany] aC;

        tef ~ exponential( 1 );
        tsd ~ exponential( 0.5 );
        ttp ~ normal( 0 , 1.5 );

        for ( i in 1:howmany ) {
            rT[i] = tef*exp(-((temp[i] - ttp)^2/2*tsd^2));
        }

        b_aC ~ normal( 0 , 0.25 );
        aC_0 ~ normal( -6 , 0.5 );
        for ( i in 1:howmany ) {
            aC[i] = exp(aC_0 + b_aC * temp[i]);
        }

        b_a ~ normal( 0 , 0.25 );
        a_0 ~ normal( -6 , 0.5 );
        for ( i in 1:howmany ) {
            a[i] = exp(a_0 + b_a * temp[i]);
        }

        b0 ~ normal(-0.7, 0.2);

        b ~ normal(0, 0.05);

        sigma ~ exponential(1);

        u1 ~ normal(u1max - (u1max-u1min)/2, u1max/10);
        u2 ~ normal(u2max - (u2max-u2min)/2, u2max/10);

        for ( i in 1:howmany ) {
                g[i] = log(u2[i] / u1[i]);
                mu[i] = b0 + rT[i] + log(u1[i])*b - aC[i]*wC[i] - a[i]*w[i];
        }
        g ~ normal(mu , sigma );
}
"
gcodesp = gsub("howmany", nrow(dG), gmodcode, fixed = T)

N <- nrow(dG)

# Initial values
initVals_gm <- list(list(u1 = rnorm(N, dG$u1 - (dG$u1-u1min)/2, dG$u1/10),
                         mu = 0,
                         u2 = rnorm(N, dG$u2 - (dG$u2-u2min)/2, dG$u2/10),
                         a_0 = -6,
                         b_a = 0,
```

```r
                                    aC_0 = -6,
                                    b_aC = 0,
                                    tef = 1,
                                    tsd = 1,
                                    ttp = 0,
                                    b = -0.5,
                                    b0 = -0.7),
                               list(u1 = rnorm(N, dG$u1 - (dG$u1-u1min)/2, dG$u1/10),
                                    mu = 0,
                                    u2 = rnorm(N, dG$u2 - (dG$u2-u2min)/2, dG$u2/10),
                                    a_0 = -6,
                                    b_a = 0,
                                    aC_0 = -6,
                                    b_aC = 0,
                                    tef = 1,
                                    tsd = 1,
                                    ttp = 0,
                                    b = -0.5,
                                    b0 = -0.7),
                               list(u1 = rnorm(N, dG$u1 - (dG$u1-u1min)/2, dG$u1/10),
                                    mu = 0,
                                    u2 = rnorm(N, dG$u2 - (dG$u2-u2min)/2, dG$u2/10),
                                    a_0 = -6,
                                    b_a = 0,
                                    aC_0 = -6,
                                    b_aC = 0,
                                    tef = 1,
                                    tsd = 1,
                                    ttp = 0,
                                    b = -0.5,
                                    b0 = -0.7),
                               list(u1 = rnorm(N, dG$u1 - (dG$u1-u1min)/2, dG$u1/10),
                                    mu = 0,
                                    u2 = rnorm(N, dG$u2 - (dG$u2-u2min)/2, dG$u2/10),
                                    a_0 = -6,
                                    b_a = 0,
                                    aC_0 = -6,
                                    b_aC = 0,
                                    tef = 1,
                                    tsd = 1,
                                    ttp = 0,
                                    b = -0.5,
                                    b0 = -0.7))

        # Use Stan to sample posterior
        gmod <- stan(model_code = gcodesp, data = d_list_gm, chains = 4, cores = 4,
                     init = initVals_gm, iter = 5500, warmup = 500,
                     control = list(adapt_delta = 0.95, max_treedepth = 15))

        # Save fitted model
        mpath_d <- paste("model-fits/", spcode, "_gmod.rds", sep = "")
        saveRDS(gmod, mpath_d)
}
```

2.3 Recruitment

We modeled taxa's probability of recruitment in all the 5 x 5 cm unoccupied quadrants in turfs in which the taxa was present the year before. Recruitment probability was a function of neighborhood crowding. Conspecific neighbors had a positive influence on recruitment probability (i.e., they were the source of propagules) while heterospecific neighbors had a negative influence (e.g., due to competition for space and resources). Both the effect of conspecific and heterospecific neighbors were functions of temperature during the first summer of the transition.

As with the other models, we started by doing a prior predictive simulation to guide our selection of prior parameter distributions.

```
### Prior predictive simulation
#r <- read_csv("processed-data/Antvul_RD.csv")
#summary(r$w)
#summary(r$w.cinter)
# Define temperature range (scaled units)
temprange <- seq(-2, 2, length.out = 1e3)

# Define mean intra and interspecific crowding
w = 5
wC = 160

# Plot space
plot(temprange, temprange,
     type = 'n', ylim = c(0,1),
     ylab = "Recruitment probability",
     xlab = "Temperature")


for(i in 1:1e3){

        aC_0 <- rnorm(1, -5, 1.5) # intercept (i.e., value at mean temperature)
        b_aC <- rnorm(1, 0, 0.5) # response to temperature
        aC <- exp(aC_0 + b_aC*temprange)

        a_0 <- rnorm(1, -2, 1.5) # intercept (i.e., value at mean temperature)
        b_a <- rnorm(1, 0, 0.5) # response to temperature
        a <- exp(a_0 + b_a*temprange)

        logodds <- a*w - aC*wC  - 5


        lines(temprange, inv_logit(logodds), col = col.alpha('black'))
}
```

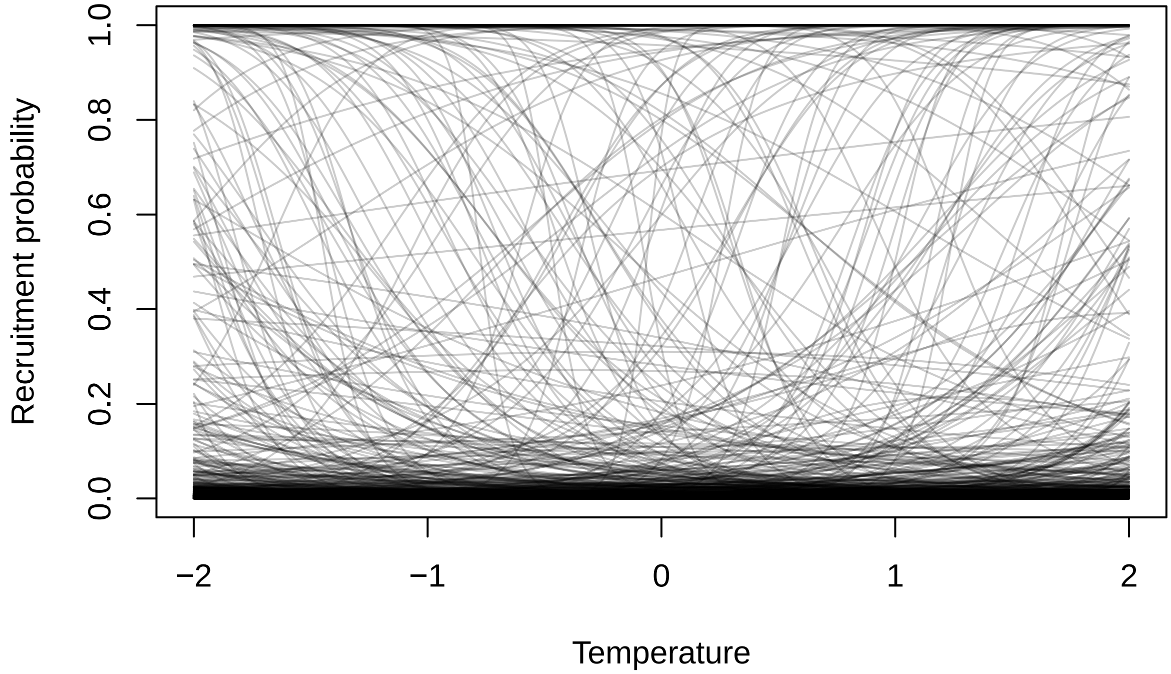

Figure S2.17 Prior predictive simulation showing the range of potential relationships between recruitment probability and temperature given the assumed prior distribution of the model parameters.

In general, the recruitment models were able to recover 'true' parameter values from simulated data (see Supporting Material X).

We modeled recruitment in each unoccupied quadrant i ('R_i') as follows.

$$R_i \sim Bernoulli(r_i)$$

$$logit(r_i) = \alpha^R_{i,i} * w_i - \alpha^R_{i,j} * w_j$$

$$\alpha^R_{i,i} = exp(a^R_{i,i} + b^R_{i,i} * T_{LS})$$

$$a^R_{i,i} \sim Normal(-2, 1.5)$$

$$b^R_{i,i} \sim Normal(0, 0.5)$$

$$\alpha^R_{i,j} = exp(a^R_{i,j} + b^R_{i,j} * T_{LS})$$

$$a^R_{i,j} \sim Normal(-5, 1.5)$$

$$b^R_{i,j} \sim Normal(0, 0.5)$$

With the code below, we fitted recruitment models for the 31 taxa with more than 1500 observations across sites and years.

```r
for(spcode in sppList){
        # Load recruitment table
        fpath_r <- paste("processed-data/", spcode, "_RD.csv", sep = "")
        r <- read.csv(fpath_r)
        r <- subset(r, x > 20 & x < 80 & y > 20 & y < 80)

        # Exclude ramets in grids with collars
        ## Alpha grids in Calanda plots
        r <- r[!(r$site == "Cal" & r$x < 50 & r$y > 50), ]

        ## Alpha or gamma grids in Nes plots
         # Nes plots with colars in grid Gamma
        collarsG = c('Nes01.03', 'Nes02.03', 'Nes04.03', 'Nes05.01',
                     'Nes08.01', 'Nes09.03')
        # Nes plots with colars in grid Alpha
        collarsA = c('Nes03.01', 'Nes06.03', 'Nes07.03', 'Nes10.01')

        r <- r[!(r$plot %in% collarsA & r$x < 50 & r$y > 50), ]
        r <- r[!(r$plot %in% collarsG & r$x < 50 & r$y < 50), ]


        # Data for model
        d_list_r <- list(recruitment = r$recruitment,
                         tempLS = (r$tempLS - mean(r$tempLS))/sd(r$tempLS),
                         w = r$w,
                         wC = r$w.cinter)

        # Starting values for sampling
        initVals <- list(list(a_0 = -2,
                              b_a = 0,
                              aC_0 = -5,
                              b_aC = 0),
                         list(a_0 = -2,
                              b_a = 0,
                              aC_0 = -5,
                              b_aC = 0))

        rmod <- ulam(
                alist(
                        recruitment ~ dbern(p),
                        logit(p) <- a*w - aC*wC - 10,
                        a <- exp(a_0 + b_a * tempLS),
                        a_0 ~ dnorm(-2, 1.5),
                        b_a ~ dnorm(0, 0.5),
                        aC <- exp(aC_0 + b_aC * tempLS),
                        aC_0 ~ dnorm(-5, 1.5),
                        b_aC ~ dnorm(0, 0.5)
                ),
                data = d_list_r, chains = 2, cores = 2, iter = 3500, warmup = 500,
                log_lik = F, init = initVals)

        # Save model fit
        rmpath <- paste("model-fits/", spcode, "_rmod.rds", sep = "")
```

```
        saveRDS(rmod,rmpath)
}
```

3. Simulations of community dynamics

Now that we have fitted statistical models describing the relationship between climate, demography, and interactions with neighbors, we can use the estimated parameters as the building blocks of an individual-based model of climate-dependent community dynamics. Next, we will test the model's ability to reproduce population and community-level responses to climate change observed in the experiment. Finally, we will project alpine community responses to upcoming decades of climate change.

3.1 Individual-based model

3.2.1 Preparing input data

Let's now prepare all the input data needed for the simulations.

Scaled model variables

We scaled some variables, such as temperature and ramet size, to facilitate model fitting. To easily transform raw variables into the correct scale used for each model, we created a data frame storing the mean and standard deviation of each variable.

```
source('scripts/spp_modfits.R')

spp_clim_scales = data.frame(spcode = sppList,
                             muT.s = numeric(length(sppList)),
                             sdT.s = numeric(length(sppList)),
                             muM.s = numeric(length(sppList)),
                             sdM.s = numeric(length(sppList)),
                             muU.s = numeric(length(sppList)),
                             sdU.s = numeric(length(sppList)),
                             muT.g = numeric(length(sppList)),
                             sdT.g = numeric(length(sppList)),
                             muT.r = numeric(length(sppList)),
                             sdT.r = numeric(length(sppList)))


comments <- read_csv("data/comments-and-voles(04062021).csv")
disturbances <- subset(comments, type %in% c('sensor', 'vole') &
                                 level %in% c('cell', 'quadrant'))

disturbances$year[is.na(disturbances$year)] <- substr(as.character(
                                            disturbances$date[is.na(disturbances$year)]),
                                                     1, 4)


for (spcode in sppList) {

    od_path = paste('processed-data/', spcode, '_DMD.csv', sep = '')
    oridatD = read_csv(od_path)
```

```r
oridatD = subset(oridatD, x > 20 & x < 80 & y > 20 & y < 80)

# Exclude ramets in grids with collars
## Alpha grids in Calanda plots
oridatD <- oridatD[!(oridatD$site == "Cal" & oridatD$x < 50 & oridatD$y > 50), ]

## Alpha or gamma grids in Nes plots
collarsG = c('Nes01.03', 'Nes02.03', 'Nes04.03',
             'Nes05.01', 'Nes08.01', 'Nes09.03')
collarsA = c('Nes03.01', 'Nes06.03', 'Nes07.03', 'Nes10.01')

oridatD <- oridatD[!(oridatD$plot %in% collarsA & oridatD$x < 50 & oridatD$y > 50), ]
oridatD <- oridatD[!(oridatD$plot %in% collarsG & oridatD$x < 50 & oridatD$y < 50), ]

# Exclude ramets that affected by disturbance

for (i in 1:nrow(disturbances)) {

        if (disturbances$level[i] == 'quadrant') {

                quadid = as.character(disturbances$quadrant_id[i])
                plotid = substr(quadid, 1, 8)
                gridid = substr(quadid, 10, 11)
                rowid = substr(quadid, 13, 13)
                colid = substr(quadid, 14, 14)
                qid = as.numeric(substr(quadid, 16, 16))

                # get quad coordinates
                xi = ifelse(gridid %in% c('BE', 'DE'), 50, 0)
                yi = ifelse(gridid %in% c('BE', 'AL'), 50, 0)

                xi = xi + as.numeric(colid)*10 - 5
                yi = yi + ifelse(rowid == "A", 45,
                                 ifelse(rowid == "B", 35,
                                        ifelse(rowid == "C", 25,
                                               ifelse(rowid == "oridatD", 15, 5))))

                xi = xi + ifelse(qid %in% c(1,3), -2.5, 2.5)
                yi = yi + ifelse(qid %in% c(3,4), -2.5, 2.5)

                rmi = which(oridatD$plot == plotid & abs(oridatD$x - xi) < 3 &
                                    abs(oridatD$y - yi) < 3 & oridatD$survival == 0 &
                                    oridatD$year + 1 == disturbances$year[i])

                if (length(rmi)>0) oridatD = oridatD[-rmi, ]

                rm(rmi, xi, yi, quadid, plotid, gridid, rowid, colid, qid)

        }


        if (disturbances$level[i] == 'cell') {
```

```r
            cellid = as.character(disturbances$cell_id[i])
            plotid = substr(cellid, 1, 8)
            gridid = substr(cellid, 10, 11)
            rowid = substr(cellid, 13, 13)
            colid = substr(cellid, 14, 14)

            # get cell coordinates
            xi = ifelse(gridid %in% c('BE', 'DE'), 50, 0)
            yi = ifelse(gridid %in% c('BE', 'AL'), 50, 0)

            xi = xi + as.numeric(colid)*10 - 5
            yi = yi + ifelse(rowid == "A", 45,
                             ifelse(rowid == "B", 35,
                                    ifelse(rowid == "C", 25,
                                           ifelse(rowid == "oridatD", 15, 5))))

            rmi = which(oridatD$plot == plotid &
                            abs(oridatD$x - xi) < 5 & abs(oridatD$y - yi) < 5 &
                                oridatD$survival == 0 &
                            oridatD$year + 1 == disturbances$year[i])

            if (length(rmi)>0) oridatD = oridatD[-rmi, ]

            rm(rmi, xi, yi, cellid, plotid, gridid, rowid, colid)
        }

}

spp_clim_scales$muT.s[which(sppList == spcode)] = mean(oridatD$tempLS)
spp_clim_scales$sdT.s[which(sppList == spcode)] = sd(oridatD$tempLS)

spp_clim_scales$muM.s[which(sppList == spcode)] = mean(oridatD$moistLS)
spp_clim_scales$sdM.s[which(sppList == spcode)] = sd(oridatD$moistLS)

spp_clim_scales$muU.s[which(sppList == spcode)] = mean(oridatD$u1)
spp_clim_scales$sdU.s[which(sppList == spcode)] = sd(oridatD$u1)

oridatDG = oridatD[oridatD$survival == 1, ]

spp_clim_scales$muT.g[which(sppList == spcode)] = mean(oridatDG$tempL2S)
spp_clim_scales$sdT.g[which(sppList == spcode)] = sd(oridatDG$tempL2S)

odr_path = paste('processed-data/', spcode, '_RD.csv', sep = '')
oridatR = read_csv(odr_path)
oridatR = subset(oridatR, x > 20 & x < 80 & y > 20 & y < 80)

# Exclude ramets in grids with collars
## Alpha grids in Calanda plots
oridatR <- oridatR[!(oridatR$site == "Cal" & oridatR$x < 50 & oridatR$y > 50), ]

## Alpha or gamma grids in Nes plots
collarsG = c('Nes01.03', 'Nes02.03', 'Nes04.03', 'Nes05.01', 'Nes08.01', 'Nes09.03')
collarsA = c('Nes03.01', 'Nes06.03', 'Nes07.03', 'Nes10.01')
```

```r
    oridatR <- oridatR[!(oridatR$plot %in% collarsA & oridatR$x < 50 & oridatR$y > 50), ]
    oridatR <- oridatR[!(oridatR$plot %in% collarsG & oridatR$x < 50 & oridatR$y < 50), ]


    spp_clim_scales$muT.r[which(sppList == spcode)] = mean(oridatR$tempLS)
    spp_clim_scales$sdT.r[which(sppList == spcode)] = sd(oridatR$tempLS)

    rm(od_path, oridatD, oridatDG, oridatR, odr_path)
}

write_csv(spp_clim_scales, 'processed-data/spp_clim_scales.csv')
```

Model parameters

Next, we create lists storing samples of all the relevant model parameters.

```r
for (spcode in sppList){

        params <- list(
                # Survival parameters
                s.tef = NA,
                s.ttp_a = NA,
                s.ttp_b = NA,
                s.k = NA,
                s.b = NA,
                s.a_0 = NA,
                s.b_a = NA,
                s.aC_0 = NA,
                s.b_aC = NA,
                # Growth parameters
                g.b0 = NA,
                g.tef = NA,
                g.tsd = NA,
                g.ttp = NA,
                g.b = NA,
                g.a_0 = NA,
                g.b_a = NA,
                g.aC_0 = NA,
                g.b_aC = NA,
                g.sigma = NA,
                # Recruitment parameters
                r.a_0 = NA,
                r.b_a = NA,
                r.aC_0 = NA,
                r.b_aC = NA
        )


        ## Survival model
        smpath <- paste("model-fits/demography/", spcode, "_smod.rds", sep = "")
        smod <- readRDS(smpath)

        params$s.tef <- extract.samples(smod, pars = "tef")$tef
```

```
        params$s.ttp_a <- extract.samples(smod, pars = "ttp_a")$ttp_a
        params$s.ttp_b <- extract.samples(smod, pars = "ttp_b")$ttp_b
        params$s.k <- extract.samples(smod, pars = "k")$k

        params$s.b <- extract.samples(smod, pars = "b")$b
        params$s.a_0 <- extract.samples(smod, pars = "a_0")$a_0
        params$s.b_a <- extract.samples(smod, pars = "b_a")$b_a
        params$s.aC_0 <- extract.samples(smod, pars = "aC_0")$aC_0
        params$s.b_aC <- extract.samples(smod, pars = "b_aC")$b_aC

        rm(smod)

        ## Growth model
        gmpath <- paste("model-fits/demography/", spcode, "_gmod.rds", sep = "")
        gmod <- readRDS(gmpath)

        params$g.b0 <- extract.samples(gmod, pars = "b0")$b0
        params$g.tef <- extract.samples(gmod, pars = "tef")$tef
        params$g.tsd <- extract.samples(gmod, pars = "tsd")$tsd
        params$g.ttp <- extract.samples(gmod, pars = "ttp")$ttp
        params$g.b <- extract.samples(gmod, pars = "b")$b
        params$g.a_0 <- extract.samples(gmod, pars = "a_0")$a_0
        params$g.b_a <- extract.samples(gmod, pars = "b_a")$b_a

        params$g.aC_0 <- extract.samples(gmod, pars = "aC_0")$aC_0
        params$g.b_aC <- extract.samples(gmod, pars = "b_aC")$b_aC
        params$g.sigma <- extract.samples(gmod, pars = "sigma")$sigma

        rm(gmod)

        ## Recruitment model
        rmpath <- paste("model-fits/demography/", spcode, "_rmod.rds", sep = "")
        rmod <- readRDS(rmpath)

        params$r.a_0 <- extract.samples(rmod, pars = "a_0")$a_0
        params$r.b_a <- extract.samples(rmod, pars = "b_a")$b_a
        params$r.aC_0 <- extract.samples(rmod, pars = "aC_0")$aC_0
        params$r.b_aC <- extract.samples(rmod, pars = "b_aC")$b_aC

        rm(rmod)
        ###

        ppath <- paste("processed-data/params_", spcode, ".rds", sep = "")
        saveRDS(params, ppath)
}
```

Recruit size distribution

Then, let's also create an object storing information about the size distribution of recruits of the different taxa.

```
source('scripts/spp_modfits.R')

recSizeDist = matrix(NA, nrow = length(sppList), ncol = 6)
```

```
for (doSpp in 1:length(sppList)) {
        rampath = paste('processed-data/', sppList[doSpp], '_ramets.csv', sep = "")

        ramets = read_csv(rampath)
        recs18  = ramets[(is.na(ramets$id_17) & !is.na(ramets$id_18)),]
        recs19  = ramets[(is.na(ramets$id_18) & !is.na(ramets$id_19)),]
        recs20  = ramets[(is.na(ramets$id_19) & !is.na(ramets$id_20)),]

        rsizes18 = subset(anbc, individual_id_year %in% recs18$id_18)$cover_cm2
        rsizes19 = subset(anbc, individual_id_year %in% recs19$id_19)$cover_cm2
        rsizes20 = subset(anbc, individual_id_year %in% recs20$id_20)$cover_cm2

        rec.sizes = c(rsizes18, rsizes19, rsizes20)

        # possible sizes
        posizes = c(1.5625, 3.125, 6.25, 12.5, 18.75, 25)

        sizeprobs = c(sum(rec.sizes == posizes[1]),
                      sum(rec.sizes == posizes[2]),
                      sum(rec.sizes == posizes[3]),
                      sum(rec.sizes == posizes[4]),
                      sum(rec.sizes == posizes[5]),
                      sum(rec.sizes >= posizes[6])) / length(rec.sizes)

        recSizeDist[doSpp, ] = sizeprobs

        rm(sizeprobs, posizes, rec.sizes, rsizes18, rsizes19,
           rsizes20, ramets, recs18, recs19, recs20, rampath)

}

saveRDS(recSizeDist, 'processed-data/recSizeDist.rds')
```

Turf ensamble

We used the 2017 maps of turfs transplanted to 2000 m (and thus with minimal climate change influence) as the starting point of our IBM simulations. To simulate the dynamics of a larger community than the individual 0.8 x 0.8 m turfs (and thus reduce the impact of stochastic extinctions of rare taxa) we created an 'ensemble' turf by patching together the 10 turfs transplanted into the 2000 m site into a rectangular arrangement of 5 x 2 turfs.

```
## Creating large meadow for simulations
### Adjust x & y coordinates to create super plot
cal17 <- subset(anbc, site == "Cal" & year == 2017)

cal17 <- subset(cal17, x > 10 & y > 10 & x < 90 & y < 90)

cal17$x[cal17$plot_id == "Cal07.01"] <- cal17$x[cal17$plot_id == "Cal07.01"] + 80
cal17$x[cal17$plot_id == "Cal08.01"] <- cal17$x[cal17$plot_id == "Cal08.01"] + 160
cal17$x[cal17$plot_id == "Cal09.01"] <- cal17$x[cal17$plot_id == "Cal09.01"] + 240
cal17$x[cal17$plot_id == "Cal10.01"] <- cal17$x[cal17$plot_id == "Cal10.01"] + 320

cal17$x[cal17$plot_id == "Cal02.01"] <- cal17$x[cal17$plot_id == "Cal02.01"] + 80
```

```r
cal17$x[cal17$plot_id == "Cal03.01"] <- cal17$x[cal17$plot_id == "Cal03.01"] + 160
cal17$x[cal17$plot_id == "Cal04.01"] <- cal17$x[cal17$plot_id == "Cal04.01"] + 240
cal17$x[cal17$plot_id == "Cal05.01"] <- cal17$x[cal17$plot_id == "Cal05.01"] + 320

cal17$y[cal17$plot_id == "Cal01.01"] <- cal17$y[cal17$plot_id == "Cal01.01"] + 80
cal17$y[cal17$plot_id == "Cal02.01"] <- cal17$y[cal17$plot_id == "Cal02.01"] + 80
cal17$y[cal17$plot_id == "Cal03.01"] <- cal17$y[cal17$plot_id == "Cal03.01"] + 80
cal17$y[cal17$plot_id == "Cal04.01"] <- cal17$y[cal17$plot_id == "Cal04.01"] + 80
cal17$y[cal17$plot_id == "Cal05.01"] <- cal17$y[cal17$plot_id == "Cal05.01"] + 80

library(readr)
write_csv(cal17, "processed-data/megaturf.csv")


qcords <- data.frame(quadrant = levels(factor(cal17$quadrant_id)))
qcords$x <- numeric(nrow(qcords))
qcords$y <- numeric(nrow(qcords))

for (i in 1:nrow(qcords)){
        qcords$x[i] <- subset(cal17, quadrant_id == qcords$quadrant[i])$x[1]
        qcords$y[i] <- subset(cal17, quadrant_id == qcords$quadrant[i])$y[1]
}

write_csv(qcords, "processed-data/qcords_megaplot.csv")
```

To avoid low crowding levels for ramets near the edges of the simulated community, we adjusted crowding for marginal ramets depending on the proportion of their neighborhood 'lost to the margin'. We assumed that the average crowding in the neighborhood area outside the margin were the same as inside the margin. This effectively makes the margins reflective.

To do this, we first visualized the neighborhood of grid cells within the 20-cm-radius neighborhood of each 'ramet'.

```r
r <- read.csv('processed-data/qcords_megaplot.csv')
r$cover_cm2 <- 1

i <- which(r$x == 212.5 & r$y == 87.5)

i_x <- r$x[i]
i_y <- r$y[i]

# Remove quadrant i to calculate intraspecific crowding
n.i <- r[-i,]

# Or not, for interspecific crowding
n.j <- r

# Keep only neighbors within 20 cm from focal ramet
n.i <- n.i[(sqrt((n.i$x - i_x)^2 + (n.i$y - i_y)^2) <=20),]
n.j <- n.j[(sqrt((n.j$x - i_x)^2 + (n.j$y - i_y)^2) <=20),]


# Plot
par(mfrow = c(1,1))
plot(n.j$x, n.j$y, pch = 21, bg = 'lightgray')
```

```r
points(i_x, i_y, pch = 0, cex = 2.5)
```

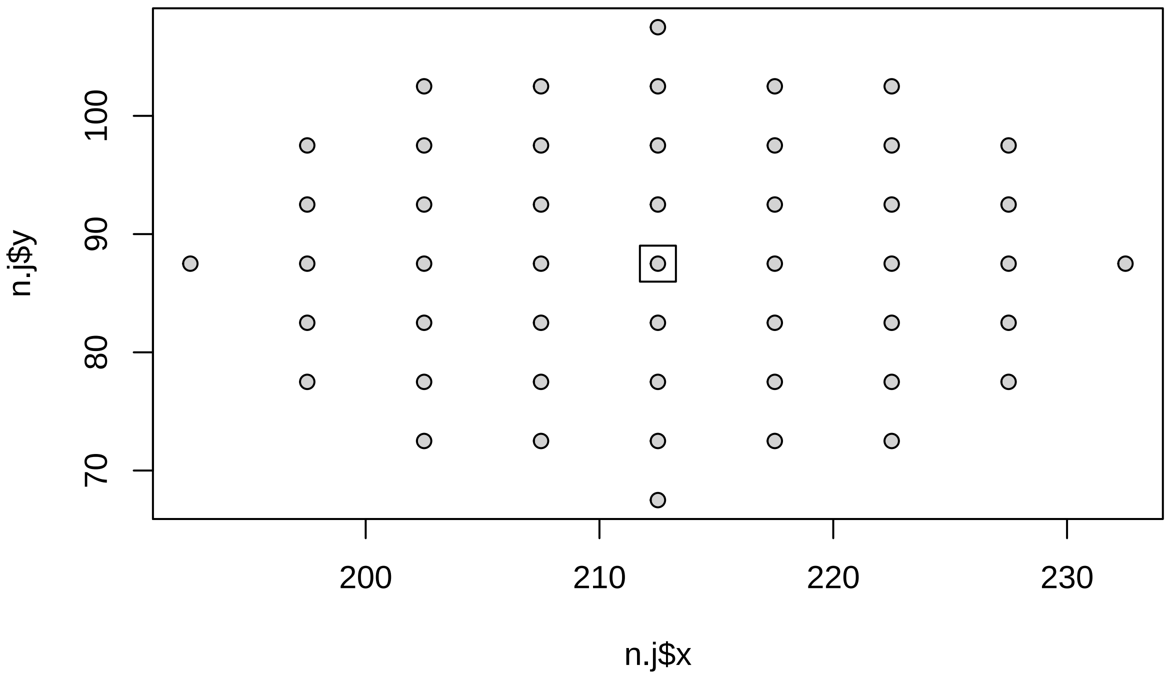

Figure S2.19 Neighbors within 20 cm of a focal ramet.

Then, we can calculate the crowding that a ramet in the center of that neighborhood (square in plot), would experience with and without a section of the neighborhood being lost to an imaginary margin.

```r
## Heterospecific crowding
# Calculate crowding in a complete neighborhood
rnd <- sqrt((r$x[i] - n.j$x)^2 + (r$y[i] - n.j$y)^2)
rnc <- n.j$cover_cm2

rnd[is.na(rnd)] <- 0
rnd2 <- rnd^2

full_wj = sum(exp(-0.02*rnd2)*rnc)


## Crowding 15 to 20 cm from one edge
x1520 = subset(n.j, x > 195)
rnd <- sqrt((r$x[i] - x1520$x)^2 + (r$y[i] - x1520$y)^2)
rnc <- x1520$cover_cm2
rnd[is.na(rnd)] <- 0
rnd2 <- rnd^2
wj1520 = sum(exp(-0.02*rnd2)*rnc)
print(paste(
"Heterospecific crowding adjustment for ramets between 15 and 20 cm from two edges:",
            round(wj1520/full_wj, 2)))
```

```
## [1] "Heterospecific crowding adjustment for ramets between 15 and 20 cm from two edges: 1"
```

```r
## Crowding 10 to 15 cm from one edge
x1015 = subset(n.j, x > 200)
rnd <- sqrt((r$x[i] - x1015$x)^2 + (r$y[i] - x1015$y)^2)
```

```r
rnc <- x1015$cover_cm2
rnd[is.na(rnd)] <- 0
rnd2 <- rnd^2
wj1015 = sum(exp(-0.02*rnd2)*rnc)
print(paste(
"Heterospecific crowding adjustment for ramets between 5 and 10 cm from two edges:",
            round(wj1015/full_wj, 2)))
```

```
## [1] "Heterospecific crowding adjustment for ramets between 5 and 10 cm from two edges: 1"
```

```r
## Crowding 5 to 10 cm from one edge (near one edge)
x510 = subset(n.j, x > 205)
rnd <- sqrt((r$x[i] - x510$x)^2 + (r$y[i] - x510$y)^2)
rnc <- x510$cover_cm2
rnd[is.na(rnd)] <- 0
rnd2 <- rnd^2
wj510 = sum(exp(-0.02*rnd2)*rnc)
print(paste(
"Heterospecific crowding adjustment for ramets between 5 and 10 cm from two edges:",
            round(wj510/full_wj, 2)))
```

```
## [1] "Heterospecific crowding adjustment for ramets between 5 and 10 cm from two edges: 0.94"
```

```r
## Crowding 0 to 5 cm from one edge (next to one edge)
x05 = subset(n.j, x > 210)
rnd <- sqrt((r$x[i] - x05$x)^2 + (r$y[i] - x05$y)^2)
rnc <- x05$cover_cm2
rnd[is.na(rnd)] <- 0
rnd2 <- rnd^2
wj05 = sum(exp(-0.02*rnd2)*rnc)
print(paste(
"Heterospecific crowding adjustment for ramets between 5 and 10 cm from two edges:",
            round(wj05/full_wj, 2)))
```

```
## [1] "Heterospecific crowding adjustment for ramets between 5 and 10 cm from two edges: 0.7"
```

```r
## Crowding 5 to 10 cm from two edges (near two edges)
xnearnear = subset(n.j, x > 205 & y < 95)
rnd <- sqrt((r$x[i] - xnearnear$x)^2 + (r$y[i] - xnearnear$y)^2)
rnc <- xnearnear$cover_cm2
rnd[is.na(rnd)] <- 0
rnd2 <- rnd^2
wjnearnear = sum(exp(-0.02*rnd2)*rnc)
print(paste(
"Heterospecific crowding adjustment for ramets between 5 and 10 cm from two edges:",
            round(wjnearnear/full_wj, 2)))
```

```
## [1] "Heterospecific crowding adjustment for ramets between 5 and 10 cm from two edges: 0.89"
```

```r
## Next to one edge, near the other
xnextnear = subset(n.j, x > 210 & y < 95)
rnd <- sqrt((r$x[i] - xnextnear$x)^2 + (r$y[i] - xnextnear$y)^2)
rnc <- xnextnear$cover_cm2
rnd[is.na(rnd)] <- 0
rnd2 <- rnd^2
wjnextnear = sum(exp(-0.02*rnd2)*rnc)
```

```r
print(paste(
"Heterospecific crowding adjustment for ramets between 5 and 10 cm from two edges:",
            round(wjnextnear/full_wj, 2))
     )
```

```
## [1] "Heterospecific crowding adjustment for ramets between 5 and 10 cm from two edges: 0.66"
```

```r
## Next to two edges
xnextnext = subset(n.j, x > 210 & y < 90)
rnd <- sqrt((r$x[i] - xnextnext$x)^2 + (r$y[i] - xnextnext$y)^2)
rnc <- xnextnext$cover_cm2
rnd[is.na(rnd)] <- 0
rnd2 <- rnd^2
wjnextnext = sum(exp(-0.02*rnd2)*rnc)
print(paste("Heterospecific crowding adjustment for ramets within 5 cm from two edges:",
            round(wjnextnext/full_wj, 2)))
```

```
## [1] "Heterospecific crowding adjustment for ramets within 5 cm from two edges: 0.49"
```

```r
### Conspecific crowding
# Calculate crowding in a complete neighborhood
rnd <- sqrt((r$x[i] - n.i$x)^2 + (r$y[i] - n.i$y)^2)
rnc <- n.j$cover_cm2

rnd[is.na(rnd)] <- 0
rnd2 <- rnd^2

full_wi = sum(exp(-0.02*rnd2)*rnc)

## Crowding 15 to 20 cm from one edge
x1520 = subset(n.i, x > 195)
rnd <- sqrt((r$x[i] - x1520$x)^2 + (r$y[i] - x1520$y)^2)
rnc <- x1520$cover_cm2
rnd[is.na(rnd)] <- 0
rnd2 <- rnd^2
wi1520 = sum(exp(-0.02*rnd2)*rnc)
wi1520/full_wi
```

```
## [1] 0.999873
```

```r
print(paste("Conspecific crowding adjustment for ramets 15 to 20 cm from the edge:",
            round(wi1520/full_wi, 2)))
```

```
## [1] "Conspecific crowding adjustment for ramets 15 to 20 cm from the edge: 1"
```

```r
## Crowding 10 to 15 cm from one edge
x1015 = subset(n.i, x > 200)
rnd <- sqrt((r$x[i] - x1015$x)^2 + (r$y[i] - x1015$y)^2)
rnc <- x1015$cover_cm2
rnd[is.na(rnd)] <- 0
rnd2 <- rnd^2
wi1015 = sum(exp(-0.02*rnd2)*rnc)
print(paste("Conspecific crowding adjustment for ramets 10 to 15 cm from the edge:",
            round(wi1015/full_wi,2)))
```

```
## [1] "Conspecific crowding adjustment for ramets 10 to 15 cm from the edge: 0.99"
```

```r
## Crowding 5 to 10 cm from one edge (near one edge)
x510 = subset(n.i, x > 205)
rnd <- sqrt((r$x[i] - x510$x)^2 + (r$y[i] - x510$y)^2)
rnc <- x510$cover_cm2
rnd[is.na(rnd)] <- 0
rnd2 <- rnd^2
wi510 = sum(exp(-0.02*rnd2)*rnc)
print(paste(
        "Conspecific crowding adjustment for ramets between 5 and 10 cm fromone edge:",
            round(wi510/full_wi, 2)))
```

```
## [1] "Conspecific crowding adjustment for ramets between 5 and 10 cm fromone edge: 0.93"
```

```r
## Crowding 0 to 5 cm from one edge (next to one edge)
x05 = subset(n.i, x > 210)
rnd <- sqrt((r$x[i] - x05$x)^2 + (r$y[i] - x05$y)^2)
rnc <- x05$cover_cm2
rnd[is.na(rnd)] <- 0
rnd2 <- rnd^2
wi05 = sum(exp(-0.02*rnd2)*rnc)
print(paste("Conspecific crowding adjustment for ramets within 5 cm of one edge:",
            round(wi05/full_wi, 2)))
```

```
## [1] "Conspecific crowding adjustment for ramets within 5 cm of one edge: 0.64"
```

```r
## Crowding 5 to 10 cm from two edges (near two edges)
xnearnear = subset(n.i, x > 205 & y < 95)
rnd <- sqrt((r$x[i] - xnearnear$x)^2 + (r$y[i] - xnearnear$y)^2)
rnc <- xnearnear$cover_cm2
rnd[is.na(rnd)] <- 0
rnd2 <- rnd^2
winearnear = sum(exp(-0.02*rnd2)*rnc)
print(paste(
        "Conspecific crowding adjustment for ramets between 5 and 10 cm from two edges:",
            round(winearnear/full_wi,2)))
```

```
## [1] "Conspecific crowding adjustment for ramets between 5 and 10 cm from two edges: 0.86"
```

```r
## Next to one edge, near the other
xnextnear = subset(n.i, x > 210 & y < 95)
rnd <- sqrt((r$x[i] - xnextnear$x)^2 + (r$y[i] - xnextnear$y)^2)
rnc <- xnextnear$cover_cm2
rnd[is.na(rnd)] <- 0
rnd2 <- rnd^2
winextnear = sum(exp(-0.02*rnd2)*rnc)
print(paste("Conspecific crowding adjustment for ramets within 5 cm of two edges:",
            round(winextnear/full_wi,2)))
```

```
## [1] "Conspecific crowding adjustment for ramets within 5 cm of two edges: 0.59"
```

```r
## Next to two edges
xnextnext = subset(n.i, x > 210 & y < 90)
rnd <- sqrt((r$x[i] - xnextnext$x)^2 + (r$y[i] - xnextnext$y)^2)
rnc <- xnextnext$cover_cm2
rnd[is.na(rnd)] <- 0
rnd2 <- rnd^2
winextnext = sum(exp(-0.02*rnd2)*rnc)
```

```r
print(paste("Conspecific crowding adjustment for ramets next to two edges:",
            round(winextnext/full_wi,2)))
```

```
## [1] "Conspecific crowding adjustment for ramets next to two edges: 0.39"
```

3.2.2 Simulator functions

We wrote two function that take a dataset of ramet covers and locations at year t (`simdat`), and projects their demography as a function of climate and neighborhood interactions. One function projects the dynamics of single turfs and was used to reproduce responses observed in the experiment. The other function projects the dynamics of the ensemble mega-turf under different scenarios of future climate change.

The single turf function is:

```r
# This function uses fitted demographic models to project ramet demography
# for one species, one time step

# Its arguments are the species name and simdat,
# a simplified version of the 'anbc' dataframe, including only one turf,
# from where it projects and returns simdat for that species the following year

ClimDemoSim_oneturf <- function(spname, simdat, rcp = "45",
                                ps = 1, meanpars = T, stepwise = F,
                                obsclim = T, final_community = F) {

    source("scripts/spp_modfits.R")
    spcode <- sppList[which(domsp == spname)]

    # 1: Prepare data
    d <- data.frame(ramet = subset(simdat, species == spname)$unique_id)

    if(nrow(d)==0) {
        return(simdat[0,])
    } else{
        d$ramet <- as.character(d$ramet)

        ## b: add predictor variables
        d$u1 <- NA
        d$x <- NA
        d$y <- NA
        d$plot <- NA

        for(i in 1:nrow(d)) {

                y1 <- simdat[simdat$unique_id == d$ramet[i],]

                d$u1[i] <- y1$cover_cm2
                d$x[i] <- y1$x
                d$y[i] <- y1$y
                d$plot[i] <- y1$plot_id
                rm(y1)
        }

        d$year <- max(simdat$year)
```

```r
## Quantify crowding

# An interaction radius of ca. 15 cm seems reasonable for starters
delta <- 0.02

# Intraspecific crowding (w)
maxnn <- 64

# Conspecific neighbor distance matrix
rnd <- matrix(0,nrow = nrow(d), ncol = maxnn-1)
# Conspecific neighbor cover matrix
rnc <- matrix(0,nrow = nrow(d), ncol = maxnn-1)

rm(maxnn)

for (i in 1:nrow(d)) {
        i_x <- d$x[i]
        i_y <- d$y[i]
        x <- subset(simdat, species == spname)
        # Keep only neighbors within 20 cm from focal ramet
        x <- x[(sqrt((x$x - i_x)^2 + (x$y - i_y)^2) <=20),]
        ri <- x[x$unique_id == d$ramet[i],]
        ni <- x[x$unique_id != d$ramet[i],]

        if (nrow(ni) > 0) {
                rnd[i,1:nrow(ni)] <- sqrt((ri$x - ni$x)^2 + (ri$y - ni$y)^2)
                rnc[i,1:nrow(ni)] <- ni$cover_cm2
        }

        rm(x,ni,ri, i_x, i_y)
}

rnd[is.na(rnd)] <- 0
rnd2 <- rnd^2

d$w <- numeric(nrow(d))

for (i in 1:nrow(d)){
        d$w[i] <- sum(exp(-delta*rnd2[i,])*rnc[i,])
}

rm(rnd, rnd2, rnc)

# Interspecific crowding
# Conspecific neighbor distance matrix
rnd <- matrix(0,nrow = nrow(d), ncol = 3385)
 # Conspecific neighbor cover matrix
rnc <- matrix(0,nrow = nrow(d), ncol = 3385)

for (i in 1:nrow(d)) {
        i_x <- d$x[i]
        i_y <- d$y[i]
```

```r
        ri <- subset(simdat, unique_id == d$ramet[i])
        x <- subset(simdat, species != spname)
        # Keep only neighbors within 20 cm from focal ramet
        x <- x[(sqrt((x$x - i_x)^2 + (x$y - i_y)^2) <=20),]

        ni <- x[x$unique_id != d$ramet[i],]

        if (nrow(ni) > 0) {
                rnd[i,1:nrow(ni)] <- sqrt((ri$x - ni$x)^2 + (ri$y - ni$y)^2)
                rnc[i,1:nrow(ni)] <- ni$cover_cm2
        }

        rm(x,ni,ri, i_x, i_y)
}

rnd[is.na(rnd)] <- 0
rnd2 <- rnd^2

d$w.cinter <- numeric(nrow(d))

for (i in 1:nrow(d)){
        d$w.cinter[i] <- sum(exp(-delta*rnd2[i,])*rnc[i,])
}

rm(rnd, rnd2, rnc)

# Increase w.cinter to account for species not included in simulations
d$w.cinter <- d$w.cinter/ifelse(final_community == T, 0.45, 0.61)

# Adjust crowding experienced by marginal ramets
## First, ramets next to two margins (i.e., at the corner)
corner <- with(d, which((x < 15 | x > 85) & (y < 15 | y > 85)))

d$w[corner] <- d$w[corner] / 0.3926553
d$w.cinter[corner] <- d$w.cinter[corner] / 0.4893565

## Then, ramets near one edge and next to other
nextnear <- with(d, which(((x < 15 | x > 85) & (y < 20 | y > 80)) |
                           ((x < 20 | x > 80) & (y < 15 | y > 85))))
nextnear <- nextnear[!(nextnear %in% corner)]

d$w[nextnear] <- d$w[nextnear] / 0.5940011
d$w.cinter[nextnear] <- d$w.cinter[nextnear] / 0.6586441

## Next, ramets near two edges
nearnear <- with(d, which(((x < 20 | x > 80) & (y < 20 | y > 80))))
nearnear <- nearnear[!((nearnear %in% corner) | (nearnear %in% nextnear))]

d$w[nearnear] <- d$w[nearnear] / 0.8650126
d$w.cinter[nearnear] <- d$w.cinter[nearnear] / 0.8865053


## Then, ramets next to only one margin
```

```r
next_to_one <- with(d, which(x < 15 | x > 85 | y < 15 | y > 85))
next_to_one <- next_to_one[!((next_to_one %in% corner) |
                                (next_to_one %in% nextnear))]
next_to_one <- next_to_one[!(next_to_one %in% nearnear)]

d$w[next_to_one] <- d$w[next_to_one] / 0.6426553
d$w.cinter[next_to_one] <- d$w.cinter[next_to_one] / 0.6995516


## Finally, ramets near to only one margin
near_to_one <- with(d, which(x < 20 | x > 80 | y < 20 | y > 80))
near_to_one <- near_to_one[!(near_to_one %in% next_to_one)]
near_to_one <- near_to_one[!((near_to_one %in% corner) |
                                (near_to_one %in% nextnear))]
near_to_one <- near_to_one[!(near_to_one %in% nearnear)]

d$w[near_to_one] <- d$w[near_to_one] / 0.9304874
d$w.cinter[near_to_one] <- d$w.cinter[near_to_one] / 0.9415551

rm(near_to_one, next_to_one, nearnear, nextnear, corner)

####
## Climate
if (obsclim == T){

        modclimdat <- read.csv("processed-data/Alcxan_DMD.csv")

        ## Mean soil moisture during the previous summer
        d$moistLS <-  max(subset(modclimdat, site == simdat$site[1] &
                                    year == max(simdat$year))$moistLS)

        ## Mean temp previous summer
        d$tempLS <-  max(subset(modclimdat, site == simdat$site[1] &
                                   year == max(simdat$year))$tempLS)

        ## Mean temp previous two summers
        d$tempL2S <- max(subset(modclimdat, site == simdat$site[1] &
                                   year == max(simdat$year))$tempL2S)

} else {
        if (stepwise == T) {
            path_climTS = paste("processed-data/swcc_rcp", rcp, ".csv", sep = "")
            climTS = read.csv(path_climTS, stringsAsFactors = F)
        } else {
            path_climTS = paste("processed-data/rcp", rcp, "TS.csv", sep = "")
            climTS = read.csv(path_climTS)
        }

        ## Mean soil moisture during the previous summer
        d$moistLS <- climTS$moistLS[climTS$year == max(d$year)]

        ## Mean temp previous summer
        d$tempLS <- climTS$tempLS[climTS$year == max(d$year)]
```

```r
        ## Mean temp previous summer
        d$tempL2S <- climTS$tempL2S[climTS$year == max(d$year)]
}

spp_clim_scales = read.csv('processed-data/spp_clim_scales.csv')


################ #
### Survival ###
################ #
spcode <- sppList[which(domsp == spname)]
p_path <- paste("processed-data/params_", spcode, ".rds", sep = "")
params <- readRDS(p_path)

spn = which(spname == domsp)

predat_s <- list(u = (d$u1 - spp_clim_scales$muU.s[spn])/
                     spp_clim_scales$sdU.s[spn],
                 tempLS = (d$tempLS - spp_clim_scales$muT.s[spn])/
                     spp_clim_scales$sdT.s[spn] ,
                 moistLS = (d$moistLS - spp_clim_scales$muM.s[spn])/
                     spp_clim_scales$sdM.s[spn],
                 w = d$w,
                 wC = d$w.cinter)

# Calculate logodds and probability of survival
## Use the mean of sampled parameters or single parameters
if (meanpars == T) {
    logodds <-  mean(params$s.tef) / (1 + exp(-mean(params$s.k) *
                                         (predat_s$tempLS -
                                         (mean(params$s.ttp_a) +
                                         mean(params$s.ttp_b) *
                                             predat_s$moistLS)))) +
                mean(params$s.b) * predat_s$u +
                (mean(params$s.a_0) + mean(params$s.b_a) *
                     predat_s$tempLS)*predat_s$w +
                (mean(params$s.aC_0)  + mean(params$s.b_aC) *
                     predat_s$tempLS)*predat_s$wC - 4
} else {
    logodds <-  params$s.tef[ps] / (1 + exp(-params$s.k[ps] *
                                    (predat_s$tempLS -
                                    (params$s.ttp_a[ps] +
                                    params$s.ttp_b[ps] *
                                        predat_s$moistLS)))) +
            params$s.b[ps] * predat_s$u +
            (params$s.a_0[ps] + params$s.b_a[ps] *
                 predat_s$tempLS )*predat_s$w +
            (params$s.aC_0[ps]  + params$s.b_aC[ps] *
                 predat_s$tempLS )*predat_s$wC - 4
}

## Survival probability
p.survival <- exp(logodds) / (exp(logodds) + 1)
```

```
# Simulate survival
d$survival <- rbinom(length(p.survival), 1, p.survival )

########## #
# Growth #
########## #
# Filter survivors
dG <- d[d$survival == 1,]

### Predict growth
gmu <- numeric(nrow(dG))

# Scale temperature with mean and sd of data used for model fitting
predT <- ((dG$tempL2S-spp_clim_scales$muT.g[spn])/spp_clim_scales$sdT.g[spn])

# Calculate mean growth rate for each surviving ramet
## Use the mean of sampled parameters or single parameters
if (meanpars == T) {

for (i in 1:nrow(dG)) {
    gmu[i] <- mean(params$g.b0) +
        mean(params$g.tef)*exp(
            -((predT[i] - mean(params$g.ttp))^2/2*mean(params$g.tsd)^2)) +
        log(dG$u1[i]) * mean(params$g.b) -
        dG$w.cinter[i] * exp(mean(params$g.aC_0) + mean(params$g.b_aC)*predT[i]) -
        dG$w[i] * exp(mean(params$g.a_0) + mean(params$g.b_a)*predT[i])
}

} else {

    for (i in 1:nrow(dG)) {
        gmu[i] <- params$g.b0[ps] +
                params$g.tef[ps]*exp(-(
                    (predT[i] - params$g.ttp[ps])^2/2*params$g.tsd[ps]^2)) +
                log(dG$u1[i]) * params$g.b[ps] -
                dG$w.cinter[i] * exp(
                    params$g.aC_0[ps] + params$g.b_aC[ps]*predT[i]) -
                dG$w[i] * exp(params$g.a_0[ps] + params$g.b_a[ps]*predT[i])
    }
}


# Simulate growth rates
## With either the mean of sigma samples, or a single sample
if (meanpars == T) gsim <- exp(rnorm(nrow(dG), gmu, mean(params$g.sigma)/1))
if (meanpars == F) gsim <- exp(rnorm(nrow(dG), gmu, params$g.sigma[ps]/1))


# Calculate cover at time t + 1
dG$u2 <- dG$u1 * gsim

# Set minimum and maximum ramet sizes to cap growth
dG$u2 <- ifelse(dG$u2 > 300, 300, dG$u2)
```

```r
dG$u2 <- ifelse(dG$u2 < 0.1, 0.1, dG$u2)

# Create data frame with ramets in year t + 1

### Keep only survivors
simdat_next_survivors <- simdat[simdat$unique_id %in% dG$ramet,]

### Make them grow
simdat_next_survivors$cover_cm2 <- dG$u2

### Update year
simdat_next_survivors$year <- simdat_next_survivors$year + 1


################# #
## Recruitment ##
################# #
## Prepare predictive data
qcords <- read.csv('processed-data/qcords_singleturfs.csv')
qcords$quadrant <- as.character(qcords$quadrant)
qcords <- qcords[grep(as.character(d$plot[1]), qcords$quadrant, fixed = T),]


# Make list of all quadrants
allquads_sp <- as.character(qcords$quadrant)

closedqs <- as.character(subset(simdat, species == spname)$quadrant_id)
openqs <- allquads_sp[!(allquads_sp %in% closedqs)]

if(length(openqs) > 0) {
    r <- data.frame(quadrant = openqs)
    r$quadrant <- as.character(r$quadrant)
    r$plot <- as.character(d$plot[1])

    ## b: add predictor variables
    r$x <- qcords$x[qcords$quadrant %in% r$quadrant]
    r$y <- qcords$y[qcords$quadrant %in% r$quadrant]

    ## Quantify crowding (w)
    rnd <- matrix(0,nrow = nrow(r), ncol = 399)
    rnc <- matrix(0,nrow = nrow(r), ncol = 399)

    for (i in 1:nrow(r)) {
        i_x <- r$x[i]
        i_y <- r$y[i]
        x <- subset(simdat, species == spname)
        # Keep only neighbors within 20 cm from focal ramet
        x <- x[(sqrt((x$x - i_x)^2 + (x$y - i_y)^2) <=20),]

        if(nrow(x) > 0){
            rnd[i,1:nrow(x)] <- sqrt((r$x[i] - x$x)^2 + (r$y[i] - x$y)^2)
            rnc[i,1:nrow(x)] <- x$cover_cm2
        }
```

```
    rm(x, i_x, i_y)
}

rnd[is.na(rnd)] <- 0
rnd2 <- rnd^2

# Quantify intraspecific crowding (w)
r$w <- numeric(nrow(r))

for (i in 1:nrow(r)){
        r$w[i] <- sum(exp(-delta*rnd2[i,])*rnc[i,])
}

rm(rnc, rnd, rnd2)

################ #
## Quantify interspecific crowding (w.cinter)
rnd <- matrix(0,nrow = nrow(r), ncol = 3385)
rnc <- matrix(0,nrow = nrow(r), ncol = 3385)

for (i in 1:nrow(r)) {
    i_x <- r$x[i]
    i_y <- r$y[i]
    x <- subset(simdat, species != spname)
    # Keep only neighbors within 20 cm from focal ramet
    x <- x[(sqrt((x$x - i_x)^2 + (x$y - i_y)^2) <=20),]

    if(nrow(x) > 0){
            rnd[i,1:nrow(x)] <- sqrt((r$x[i] - x$x)^2 + (r$y[i] - x$y)^2)
            rnc[i,1:nrow(x)] <- x$cover_cm2
    }

    rm(x, i_x, i_y)
}

rnd[is.na(rnd)] <- 0
rnd2 <- rnd^2


# Quantify interspecific crowding (w.cinter)
r$w.cinter <- numeric(nrow(r))

for (i in 1:nrow(r)){
        r$w.cinter[i] <- sum(exp(-delta*rnd2[i,])*rnc[i,])
}

rm(rnc, rnd, rnd2)


# Increase w.cinter to account for species not included in simulations
r$w.cinter <- r$w.cinter/ifelse(final_community == T, 0.45, 0.61)
```

```r
# Adjust crowding experienced by marginal ramets
## First, ramets next to two margins (i.e., at the corner)
corner <- with(r, which((x < 15 | x > 85) & (y < 15 | y > 85)))

r$w[corner] <- r$w[corner] / 0.3926553
r$w.cinter[corner] <- r$w.cinter[corner] / 0.4893565

## Then, ramets near one edge and next to other
nextnear <- with(r, which(((x < 15 | x > 85) & (y < 20 | y > 80)) |
                                  ((x < 20 | x > 80) & (y < 15 | y > 85))))
nextnear <- nextnear[!(nextnear %in% corner)]

r$w[nextnear] <- r$w[nextnear] / 0.5940011
r$w.cinter[nextnear] <- r$w.cinter[nextnear] / 0.6586441

## Next, ramets near two edges
nearnear <- with(r, which(((x < 20 | x > 80) & (y < 20 | y > 80))))
nearnear <- nearnear[!((nearnear %in% corner) | (nearnear %in% nextnear))]

r$w[nearnear] <- r$w[nearnear] / 0.8650126
r$w.cinter[nearnear] <- r$w.cinter[nearnear] / 0.8865053


## Then, ramets next to only one margin
next_to_one <- with(r, which(x < 15 | x > 85 | y < 15 | y > 85))
next_to_one <- next_to_one[!((next_to_one %in% corner) |
                                 (next_to_one %in% nextnear))]
next_to_one <- next_to_one[!(next_to_one %in% nearnear)]

r$w[next_to_one] <- r$w[next_to_one] / 0.6426553
r$w.cinter[next_to_one] <- r$w.cinter[next_to_one] / 0.6995516


## Finally, ramets near to only one margin
near_to_one <- with(r, which(x < 20 | x > 80 | y < 20 | y > 80))
near_to_one <- near_to_one[!(near_to_one %in% next_to_one)]
near_to_one <- near_to_one[!((near_to_one %in% corner) |
                                 (near_to_one %in% nextnear))]
near_to_one <- near_to_one[!(near_to_one %in% nearnear)]

r$w[near_to_one] <- r$w[near_to_one] / 0.9304874
r$w.cinter[near_to_one] <- r$w.cinter[near_to_one] / 0.9415551

rm(near_to_one, next_to_one, nearnear, nextnear, corner)



# Recruitment climate
r$tempLS <- d$tempLS[1]


### PREDICT WITH MODEL
# Scale temperature with mean and sd of data used to train the model
```

```r
predT <- ((r$tempLS) - spp_clim_scales$muT.r[spn])/spp_clim_scales$sdT.r[spn]

# Data for model predictions
predat_r <- list(temp = predT,
                 w = r$w,
                 wC = r$w.cinter)

# Calculate logodds and probability of recruitment

if (meanpars == T) {
    logodds.r <- exp(mean(params$r.a_0) + mean(params$r.b_a)*predat_r$temp) *
        predat_r$w - exp(
            mean(params$r.aC_0)  + mean(params$r.b_aC)*predat_r$temp) *
        predat_r$wC - 10
} else {
    logodds.r <- exp(params$r.a_0[ps] + params$r.b_a[ps] * predat_r$temp )*
        predat_r$w - exp(
            params$r.aC_0[ps]  + params$r.b_aC[ps]  * predat_r$temp )*
        predat_r$wC -10
}

# Probability of recruitment in each quadrant
p.recruitment <- exp(logodds.r) / (exp(logodds.r) + 1)

# Simulate recruitment
r$recruitment <- rbinom(length(p.recruitment), 1, p.recruitment )

## Recruit size model
recs <- subset(r, recruitment == 1)

recSizeDist = readRDS('processed-data/recSizeDist.rds')
posizes = c(1.5625, 3.125, 6.25, 12.5, 18.75, 25)

rec.sizes <- sample(posizes, nrow(recs), replace = T,
                    prob = recSizeDist[spn,])

### Prepare data frame to add recruits to next time step
nrec <- sum(r$recruitment)
newramets <- data.frame(species = rep(spname, nrec),
                        x = recs$x,
                        y = recs$y,
                        year = rep(simdat_next_survivors$year[1],nrec),
                        cover_cm2 = rec.sizes,
                        quadrant_id = recs$quadrant,
                        site = rep(simdat$site[1], nrec),
                        plot_id = rep(simdat$plot_id[1], nrec),
                        unique_id = character(nrec),
                        stringsAsFactors = F)

rm(nrec)

simdat_next <- rbind(simdat_next_survivors, newramets)
rm(simdat_next_survivors, newramets, recs, d, r)
```

```
        } else {
                simdat_next <- simdat_next_survivors
                rm(simdat_next_survivors, d)
        }

        return(simdat_next)
    }


}
```

The mega-turf function has arguments to perform different simulation types. The argument `climchange` determines whether simulated dynamics occur under a changing or a constant climate. If `climchange` = `TRUE` (the default), then the function inputs the weather conditions of year t from the time series corresponding the the RCP scenario indicated by argument `rcp` (default = "45"). The argument `stepwise` (default = `FALSE`) determines whether climate change is gradual, as simulated by CH2018, or stepwise, as often in climate change experiments.

If `climchange` is not TRUE, then the function simulates dynamics under constant climatic conditions. If the argument `meanclim` = `TRUE`, this constant climate is the mean temperature of soil moisture in the period 2017 to 2020 across the RCP 2.6, 4.5, and 8.5 timeseries. Otherwise, instead of the mean, the function samples at each iteration from the 12 sets of weather values (i.e., 4 years for each of the RCP scenarios).

The function also has the arguments `spname` (indicating the taxon to simulate), and `meanpars` (default = T), whether to use the mean of parameter samples, otherwise, use sample `ps`.

```
# This function uses fitted demographic models to project ramet demography
# for one species, one time step
# For different climate change scenarios for the remainder of the XXI century

# Its arguments are the species name and simdat,
# a simplified version of the 'anbc' dataframe, INCLUDING ONLY CAL SITE,
# from where it projects and returns simdat for that species the following year

# simdat = simdat_t1
# spname = domsp[1]
#  rcp = "26"

ClimDemoSim <- function(spname, simdat, rcp = "45", current_clim = F,
                        ps = 1, meanpars = T, stepwise = F, smoothline = F) {

  source("scripts/spp_modfits.R")
  spcode <- sppList[which(domsp == spname)]

  # 1: Prepare data
  d <- data.frame(ramet = subset(simdat, species == spname)$unique_id)

  if(nrow(d)==0) {
    return(simdat[0,])
  } else{
    d$ramet <- as.character(d$ramet)

    ## b: add predictor variables
    d$u1 <- NA
    d$x <- NA
```

```r
d$y <- NA

for(i in 1:nrow(d)) {

  y1 <- simdat[simdat$unique_id == d$ramet[i],]

  d$u1[i] <- y1$cover_cm2
  d$x[i] <- y1$x
  d$y[i] <- y1$y
  rm(y1)
}

d$year <- max(simdat$year)

## Quantify crowding

# An interaction radius of ca. 15 cm seems reasonable for starters
delta <- 0.02

# Intraspecific crowding (w)
maxnn <- 64

rnd <- matrix(0,nrow = nrow(d), ncol = maxnn-1) # Conspecific neighbor distance matrix
rnc <- matrix(0,nrow = nrow(d), ncol = maxnn-1) # Conspecific neighbor cover matrix

rm(maxnn)

for (i in 1:nrow(d)) {
  i_x <- d$x[i]
  i_y <- d$y[i]
  x <- subset(simdat, species == spname)
  # Keep only neighbors within 20 cm from focal ramet
  x <- x[(sqrt((x$x - i_x)^2 + (x$y - i_y)^2) <=20),]
  ri <- x[x$unique_id == d$ramet[i],]
  ni <- x[x$unique_id != d$ramet[i],]

  if (nrow(ni) > 0) {
    rnd[i,1:nrow(ni)] <- sqrt((ri$x - ni$x)^2 + (ri$y - ni$y)^2)
    rnc[i,1:nrow(ni)] <- ni$cover_cm2
  }

  rm(x,ni,ri, i_x, i_y)
}

rnd[is.na(rnd)] <- 0
rnd2 <- rnd^2

d$w <- numeric(nrow(d))

for (i in 1:nrow(d)){
  d$w[i] <- sum(exp(-delta*rnd2[i,])*rnc[i,])
}
```

```r
rm(rnd, rnd2, rnc)

# Interspecific crowding
rnd <- matrix(0,nrow = nrow(d), ncol = 3385) # Conspecific neighbor distance matrix
rnc <- matrix(0,nrow = nrow(d), ncol = 3385) # Conspecific neighbor cover matrix

for (i in 1:nrow(d)) {
  i_x <- d$x[i]
  i_y <- d$y[i]

  ri <- subset(simdat, unique_id == d$ramet[i])
  x <- subset(simdat, species != spname)
  # Keep only neighbors within 20 cm from focal ramet
  x <- x[(sqrt((x$x - i_x)^2 + (x$y - i_y)^2) <=20),]

  ni <- x[x$unique_id != d$ramet[i],]

  if (nrow(ni) > 0) {
    rnd[i,1:nrow(ni)] <- sqrt((ri$x - ni$x)^2 + (ri$y - ni$y)^2)
    rnc[i,1:nrow(ni)] <- ni$cover_cm2
  }

  rm(x,ni,ri, i_x, i_y)
}

rnd[is.na(rnd)] <- 0
rnd2 <- rnd^2

d$w.cinter <- numeric(nrow(d))

for (i in 1:nrow(d)){
  d$w.cinter[i] <- sum(exp(-delta*rnd2[i,])*rnc[i,])
}

rm(rnd, rnd2, rnc)

# Increase w.cinter to account for species not included in simulations
#d$w.cinter <- d$w.cinter/0.45

# Adjust crowding experienced by marginal ramets
## First, ramets next to two margins (i.e., at the corner)
corner <- with(d, which((x < 15 | x > 405) & (y < 15 | y > 165)))

d$w[corner] <- d$w[corner] / 0.3926553
d$w.cinter[corner] <- d$w.cinter[corner] / 0.4893565

## Then, ramets near one edge and next to other
nextnear <- with(d, which(((x < 15 | x > 405) & (y < 20 | y > 160)) |
                            ((x < 20 | x > 400) & (y < 15 | y > 165))))
nextnear <- nextnear[!(nextnear %in% corner)]

d$w[nextnear] <- d$w[nextnear] / 0.5940011
d$w.cinter[nextnear] <- d$w.cinter[nextnear] / 0.6586441
```

```
## Next, ramets near two edges
nearnear <- with(d, which(((x < 20 | x > 400) & (y < 20 | y > 160))))
nearnear <- nearnear[!((nearnear %in% corner) | (nearnear %in% nextnear))]

d$w[nearnear] <- d$w[nearnear] / 0.8650126
d$w.cinter[nearnear] <- d$w.cinter[nearnear] / 0.8865053


## Then, ramets next to only one margin
next_to_one <- with(d, which(x < 15 | x > 405 | y < 15 | y > 165))
next_to_one <- next_to_one[!((next_to_one %in% corner) | (next_to_one %in% nextnear))]
next_to_one <- next_to_one[!(next_to_one %in% nearnear)]

d$w[next_to_one] <- d$w[next_to_one] / 0.6426553
d$w.cinter[next_to_one] <- d$w.cinter[next_to_one] / 0.6995516


## Finally, ramets near to only one margin
near_to_one <- with(d, which(x < 20 | x > 400 | y < 20 | y > 160))
near_to_one <- near_to_one[!(near_to_one %in% next_to_one)]
near_to_one <- near_to_one[!((near_to_one %in% corner) | (near_to_one %in% nextnear))]
near_to_one <- near_to_one[!(near_to_one %in% nearnear)]

d$w[near_to_one] <- d$w[near_to_one] / 0.9304874
d$w.cinter[near_to_one] <- d$w.cinter[near_to_one] / 0.9415551

rm(near_to_one, next_to_one, nearnear, nextnear, corner)

####
## Climate
if (stepwise == T) {
  path_climTS = paste("processed-daata/swcc_rcp", rcp, ".csv", sep = "")
  climTS = read.csv(path_climTS, stringsAsFactors = F)
} else {

  if (current_clim) {
    set.seed(1)
    climTS = data.frame(year = 2017:2098)
    climTS$tempLS <- rnorm(82, 12.84, 0.25)
    climTS$tempL2S <- rnorm(82, 12.84, 0.25)
    climTS$moistLS <- rnorm(82, 0.32, 0.02)

  } else {
    path_climTS = paste("processed-daata/rcp", rcp, "TS.csv", sep = "")
    climTS = read.csv(path_climTS)
  }

}

if (smoothline == T) {
  climTS$moistLS <- seq(from = climTS$moistLS[1],
                        to = climTS$moistLS[82],
                        length.out = 82)
```

```r
  climTS$tempLS <- seq(from = climTS$tempLS[1],
                       to = climTS$tempLS[82],
                       length.out = 82)

  climTS$tempL2S <- seq(from = climTS$tempL2S[1],
                        to = climTS$tempL2S[82],
                        length.out = 82)
}

spp_clim_scales = read.csv('processed-daata/spp_clim_scales.csv')

## Mean soil moisture during the previous summer
d$moistLS <- climTS$moistLS[climTS$year == max(d$year)]

## Mean temp previous summer
d$tempLS <- climTS$tempLS[climTS$year == max(d$year)]

## Mean temp previous summer
d$tempL2S <- climTS$tempL2S[climTS$year == max(d$year)]


################ ##
### Survival ###
################ ##
p_path <- paste("processed-data/params_", spcode, ".rds", sep = "")
params <- readRDS(p_path)

spn = which(spname == domsp)

predat_s <- list(u = (d$u1 - spp_clim_scales$muU.s[spn])/spp_clim_scales$sdU.s[spn],
                 templS = (d$tempLS - spp_clim_scales$muT.s[spn])/
                     spp_clim_scales$sdT.s[spn] ,
                 moistLS = (d$moistLS - spp_clim_scales$muM.s[spn])/
                     spp_clim_scales$sdM.s[spn],
                 w = d$w,
                 wC = d$w.cinter)

# Calculate logodds and probability of survival
## Use the mean of sampled parameters or single parameters
if (meanpars == T) {
  logodds <-  mean(params$s.tef) / (1 + exp(-mean(params$s.k) *
                                        (predat_s$tempLS -
                                             (mean(params$s.ttp_a) +
                                                  mean(params$s.ttp_b) *
                                                  predat_s$moistLS)))) +
    mean(params$s.b) * predat_s$u +
    (mean(params$s.a_0) + mean(params$s.b_a) * predat_s$tempLS)*predat_s$w +
    (mean(params$s.aC_0)  + mean(params$s.b_aC) * predat_s$tempLS)*predat_s$wC - 4
} else {
  logodds <-  params$s.tef[ps] / (1 + exp(-params$s.k[ps] *
                                      (predat_s$tempLS -
                                           (params$s.ttp_a[ps] +
                                                params$s.ttp_b[ps] *
```

```
                                                    predat_s$moistLS)))) +
      params$s.b[ps] * predat_s$u +
      (params$s.a_0[ps] + params$s.b_a[ps] * predat_s$tempLS )*predat_s$w +
      (params$s.aC_0[ps]  + params$s.b_aC[ps] * predat_s$tempLS )*predat_s$wC - 4
  }

  ## Survival probability
  p.survival <- exp(logodds) / (exp(logodds) + 1)

  # Simulate survival
  d$survival <- rbinom(length(p.survival), 1, p.survival )

  ########## ##
  # Growth #
  ########## ##
  # Filter survivors
  dG <- d[d$survival == 1,]

  ### Predict growth
  gmu <- numeric(nrow(dG))

  # Scale temperature with mean and sd of data used for model fitting
  predT <- ((dG$tempL2S-spp_clim_scales$muT.g[spn])/spp_clim_scales$sdT.g[spn])

  # Calculate mean growth rate for each surviving ramet
  ## Use the mean of sampled parameters or single parameters
  if (meanpars == T) {

    for (i in 1:nrow(dG)) {
      gmu[i] <- mean(params$g.b0) +
        mean(params$g.tef)*exp(-((predT[i] - mean(params$g.ttp))^2/
                                   2*mean(params$g.tsd)^2)) +
        log(dG$u1[i]) * mean(params$g.b) -
        dG$w.cinter[i] * exp(mean(params$g.aC_0) + mean(params$g.b_aC)*predT[i]) -
        dG$w[i] * exp(mean(params$g.a_0) + mean(params$g.b_a)*predT[i])
    }

  } else {

    for (i in 1:nrow(dG)) {
      gmu[i] <- params$g.b0[ps] +
        params$g.tef[ps]*exp(-((predT[i] - params$g.ttp[ps])^2/2*params$g.tsd[ps]^2)) +
        log(dG$u1[i]) * params$g.b[ps] -
        dG$w.cinter[i] * exp(params$g.aC_0[ps] + params$g.b_aC[ps]*predT[i]) -
        dG$w[i] * exp(params$g.a_0[ps] + params$g.b_a[ps]*predT[i])
    }
  }


  # Simulate growth rates
  ## With either the mean of sigma samples, or a single sample
  if (meanpars == T) gsim <- exp(rnorm(nrow(dG), gmu, mean(params$g.sigma)/1))
  if (meanpars == F) gsim <- exp(rnorm(nrow(dG), gmu, params$g.sigma[ps]/1))
```

```r
# Calculate cover at time t + 1
dG$u2 <- dG$u1 * gsim

# Set minimum and maximum ramet sizes to cap growth
dG$u2 <- ifelse(dG$u2 > 300, 300, dG$u2)
dG$u2 <- ifelse(dG$u2 < 0.1, 0.1, dG$u2)

# Create data frame with ramets in year t + 1

### Keep only survivors
simdat_next_survivors <- simdat[simdat$unique_id %in% dG$ramet,]

### Make them grow
simdat_next_survivors$cover_cm2 <- dG$u2

### Update year
simdat_next_survivors$year <- simdat_next_survivors$year + 1


################# #
## Recruitment ##
################# #
## Prepare predictive data
qcords <- read.csv('processed-daata/qcords_megaplot.csv')
# Make list of all quadrants
allquads_sp <- as.character(qcords$quadrant)

closedqs <- as.character(subset(simdat, species == spname)$quadrant_id)
openqs <- allquads_sp[!(allquads_sp %in% closedqs)]

if(length(openqs) > 0) {
  r <- data.frame(quadrant = openqs)
  r$quadrant <- as.character(r$quadrant)

  ## b: add predictor variables
  r$x <- qcords$x[qcords$quadrant %in% r$quadrant]
  r$y <- qcords$y[qcords$quadrant %in% r$quadrant]

  ## Quantify crowding (w)
  rnd <- matrix(0,nrow = nrow(r), ncol = 399)
  rnc <- matrix(0,nrow = nrow(r), ncol = 399)

  for (i in 1:nrow(r)) {
    i_x <- r$x[i]
    i_y <- r$y[i]
    x <- subset(simdat, species == spname)
    x <- x[(sqrt((x$x - i_x)^2 + (x$y - i_y)^2) <=20),]

    if(nrow(x) > 0){
      rnd[i,1:nrow(x)] <- sqrt((r$x[i] - x$x)^2 + (r$y[i] - x$y)^2)
      rnc[i,1:nrow(x)] <- x$cover_cm2
    }
```

```r
  rm(x, i_x, i_y)
}

rnd[is.na(rnd)] <- 0
rnd2 <- rnd^2

# Quantify intraspecific crowding (w)
r$w <- numeric(nrow(r))

for (i in 1:nrow(r)){
  r$w[i] <- sum(exp(-delta*rnd2[i,])*rnc[i,])
}

rm(rnc, rnd, rnd2)

################# #
## Quantify interspecific crowding (w.cinter)
rnd <- matrix(0,nrow = nrow(r), ncol = 3385)
rnc <- matrix(0,nrow = nrow(r), ncol = 3385)

for (i in 1:nrow(r)) {
  i_x <- r$x[i]
  i_y <- r$y[i]
  x <- subset(simdat, species != spname)
  x <- x[(sqrt((x$x - i_x)^2 + (x$y - i_y)^2) <=20),]

  if(nrow(x) > 0){
    rnd[i,1:nrow(x)] <- sqrt((r$x[i] - x$x)^2 + (r$y[i] - x$y)^2)
    rnc[i,1:nrow(x)] <- x$cover_cm2
  }

  rm(x, i_x, i_y)
}

rnd[is.na(rnd)] <- 0
rnd2 <- rnd^2


# Quantify interspecific crowding (w.cinter)
r$w.cinter <- numeric(nrow(r))

for (i in 1:nrow(r)){
  r$w.cinter[i] <- sum(exp(-delta*rnd2[i,])*rnc[i,])
}

rm(rnc, rnd, rnd2)


# Adjust crowding experienced by marginal ramets
## First, ramets next to two margins (i.e., at the corner)
corner <- with(r, which((x < 15 | x > 405) & (y < 15 | y > 165)))

r$w[corner] <- r$w[corner] / 0.3926553
```

```r
r$w.cinter[corner] <- r$w.cinter[corner] / 0.4893565

## Then, ramets near one edge and next to other
nextnear <- with(r, which(((x < 15 | x > 405) & (y < 20 | y > 160)) |
                          ((x < 20 | x > 400) & (y < 15 | y > 165))))
nextnear <- nextnear[!(nextnear %in% corner)]

r$w[nextnear] <- r$w[nextnear] / 0.5940011
r$w.cinter[nextnear] <- r$w.cinter[nextnear] / 0.6586441

## Next, ramets near two edges
nearnear <- with(r, which(((x < 20 | x > 400) & (y < 20 | y > 160))))
nearnear <- nearnear[!((nearnear %in% corner) | (nearnear %in% nextnear))]

r$w[nearnear] <- r$w[nearnear] / 0.8650126
r$w.cinter[nearnear] <- r$w.cinter[nearnear] / 0.8865053


## Then, ramets next to only one margin
next_to_one <- with(r, which(x < 15 | x > 405 | y < 15 | y > 165))
next_to_one <- next_to_one[!((next_to_one %in% corner) |
                             (next_to_one %in% nextnear))]
next_to_one <- next_to_one[!(next_to_one %in% nearnear)]

r$w[next_to_one] <- r$w[next_to_one] / 0.6426553
r$w.cinter[next_to_one] <- r$w.cinter[next_to_one] / 0.6995516


## Finally, ramets near to only one margin
near_to_one <- with(r, which(x < 20 | x > 400 | y < 20 | y > 160))
near_to_one <- near_to_one[!(near_to_one %in% next_to_one)]
near_to_one <- near_to_one[!((near_to_one %in% corner) |
                             (near_to_one %in% nextnear))]
near_to_one <- near_to_one[!(near_to_one %in% nearnear)]

r$w[near_to_one] <- r$w[near_to_one] / 0.9304874
r$w.cinter[near_to_one] <- r$w.cinter[near_to_one] / 0.9415551

rm(near_to_one, next_to_one, nearnear, nextnear, corner)



# Recruitment climate
r$tempLS <- d$tempLS[1]


### PREDICT WITH MODEL
# Scale temperature with mean and sd of data used to train the model
predT <- ((r$tempLS) - spp_clim_scales$muT.r[spn])/spp_clim_scales$sdT.r[spn]

# Data for model predictions
predat_r <- list(temp = predT,
                 w = r$w,
```

```
                      wC = r$w.cinter)

      # Calculate logodds and probability of recruitment

      if (meanpars == T) {
        logodds.r <- exp(mean(params$r.a_0)+mean(params$r.b_a)*predat_r$temp)*predat_r$w -
          exp(mean(params$r.aC_0)  + mean(params$r.b_aC)*predat_r$temp)*predat_r$wC - 10
      } else {
        logodds.r <- exp(params$r.a_0[ps] + params$r.b_a[ps]*predat_r$temp )*predat_r$w -
          exp(params$r.aC_0[ps]  + params$r.b_aC[ps]  * predat_r$temp )*predat_r$wC - 10
      }

      # Probability of recruitment in each quadrant
      p.recruitment <- exp(logodds.r) / (exp(logodds.r) + 1)

      # Simulate recruitment
      r$recruitment <- rbinom(length(p.recruitment), 1, p.recruitment )

      ## Recruit size model
      recs <- subset(r, recruitment == 1)

      recSizeDist = readRDS('processed-data/recSizeDist.rds')
      posizes = c(1.5625, 3.125, 6.25, 12.5, 18.75, 25)

      rec.sizes <- sample(posizes, nrow(recs), replace = T, prob = recSizeDist[spn,])

      ### Prepare data frame to add recruits to next time step
      nrec <- sum(r$recruitment)
      newramets <- data.frame(species = rep(spname, nrec),
                              x = recs$x,
                              y = recs$y,
                              year = rep(simdat_next_survivors$year[1],nrec),
                              cover_cm2 = rec.sizes,
                              quadrant_id = recs$quadrant,
                              unique_id = character(nrec),
                              stringsAsFactors = F)

      rm(nrec)

      simdat_next <- rbind(simdat_next_survivors, newramets)
      rm(simdat_next_survivors, newramets, recs, d, r)

    } else {
      simdat_next <- simdat_next_survivors
      rm(simdat_next_survivors, d)
    }

    return(simdat_next)
  }

}
```

3.2 Observed vs predicted dynamics during experimental period

Using 2017 vegetation surveys as the starting point, we projected annual dynamics until year 2020 using the single-turf simulator function. We projected dynamics as a function of climatic conditions estimated at each site during the experimental period.

```
library(rethinking)
library(dplyr)

source('scripts/spp_modfits.R')
source("scripts/ClimDemoSim_oneturf.R")

transturfs <- levels(factor(anbc$plot_id))[1:40]

for (tt in 1:40){
  simdat_t1 <- subset(anbc, plot_id == transturfs[tt] & year == 2017)
  simdat_t1 <- subset(simdat_t1, species %in% domsp)
  simdat_t1 <- select(simdat_t1,
                      species, x, y, year, cover_cm2, quadrant_id, site, plot_id)
  simdat_t1$unique_id <- paste("t1_", 1:nrow(simdat_t1), sep = "")
  simdat_t1$species <- as.character(simdat_t1$species)
  simdat_t1$site <- as.character(simdat_t1$site)
  simdat_t1$plot_id <- as.character(simdat_t1$plot_id)

  allsims <- simdat_t1

  nyears = 2020 - simdat_t1$year[1]

  for (i in 1:nyears){

    simdat_t2 <- simdat_t1[0,]

    for (s in domsp) {
      sp_t2 <- ClimDemoSim_oneturf(s, simdat = simdat_t1, meanpars = T,
                                   obsclim = T)
      simdat_t2 <- rbind(simdat_t2, sp_t2)
      rm(sp_t2)
    }

    simdat_t2$year[is.na(simdat_t2$year)] <- max(simdat_t2$year, na.rm = T)

    simdat_t2$species <- as.character(simdat_t2$species)
    simdat_t2$quadrant_id <- as.character(simdat_t2$quadrant_id)
    simdat_t2$unique_id <- 1:nrow(simdat_t2)

    allsims <- rbind(allsims, simdat_t2)
    simdat_t1 <- simdat_t2
  }

  save_path <- paste("simulations/single_turfs/sims_", transturfs[tt], "_meanpars.csv",
                     sep = "")
  write.csv(allsims, save_path)
}
```

```
fc_pvso <- data.frame(plot = rep(transturfs, each = length(domsp)),
                      species = rep(domsp, 40))


fc_pvso$cov17_obs <- numeric(nrow(fc_pvso))
fc_pvso$cov18_obs <- numeric(nrow(fc_pvso))
fc_pvso$cov19_obs <- numeric(nrow(fc_pvso))
fc_pvso$cov20_obs <- numeric(nrow(fc_pvso))

fc_pvso$n17_obs <- numeric(nrow(fc_pvso))
fc_pvso$n18_obs <- numeric(nrow(fc_pvso))
fc_pvso$n19_obs <- numeric(nrow(fc_pvso))
fc_pvso$n20_obs <- numeric(nrow(fc_pvso))

fc_pvso$cov18_pred <- numeric(nrow(fc_pvso))
fc_pvso$cov19_pred <- numeric(nrow(fc_pvso))
fc_pvso$cov20_pred <- numeric(nrow(fc_pvso))

fc_pvso$n18_pred <- numeric(nrow(fc_pvso))
fc_pvso$n19_pred <- numeric(nrow(fc_pvso))
fc_pvso$n20_pred <- numeric(nrow(fc_pvso))

for(turf in transturfs){

  predat <- read.csv( paste("simulations/single_turfs/sims_", turf, "_meanpars.csv",
                            sep = ""))

  for(sp in domsp) {
    obs_17 <- subset(anbc, plot_id == turf & species == sp & year == 2017)
    obs_18 <- subset(anbc, plot_id == turf & species == sp & year == 2018)
    obs_19 <- subset(anbc, plot_id == turf & species == sp & year == 2019)
    obs_20 <- subset(anbc, plot_id == turf & species == sp & year == 2020)

    pred_18 <- subset(predat, plot_id == turf & species == sp & year == 2018)
    pred_19 <- subset(predat, plot_id == turf & species == sp & year == 2019)
    pred_20 <- subset(predat, plot_id == turf & species == sp & year == 2020)

    pred_18$covcat <- ifelse(pred_18$cover_cm2 < 1.5625, 1.5625,
                        ifelse(pred_18$cover_cm2 < 3.125, 3.125,
                          ifelse(pred_18$cover_cm2 < 6.25, 6.25,
                            ifelse(pred_18$cover_cm2 < 12.5, 12.5,
                              ifelse(pred_18$cover_cm2<18.75, 18.75,
                                ifelse(pred_18$cover_cm2<25, 25,
                                  pred_18$cover_cm2))))))

    pred_19$covcat <- ifelse(pred_19$cover_cm2 < 1.5625, 1.5625,
                        ifelse(pred_19$cover_cm2 < 3.125, 3.125,
                          ifelse(pred_19$cover_cm2 < 6.25, 6.25,
                            ifelse(pred_19$cover_cm2 < 12.5, 12.5,
                              ifelse(pred_19$cover_cm2<18.75, 18.75,
                                ifelse(pred_19$cover_cm2<25, 25,
                                  pred_19$cover_cm2))))))
```

```
    pred_20$covcat <- ifelse(pred_20$cover_cm2 < 1.5625, 1.5625,
                            ifelse(pred_20$cover_cm2 < 3.125, 3.125,
                                   ifelse(pred_20$cover_cm2 < 6.25, 6.25,
                                          ifelse(pred_20$cover_cm2 < 12.5, 12.5,
                                                 ifelse(pred_20$cover_cm2<18.75, 18.75,
                                                        ifelse(pred_20$cover_cm2<25,25,
                                                               pred_20$cover_cm2))))))


    ri <- which(fc_pvso$plot == turf & fc_pvso$species == sp)


    fc_pvso$cov17_obs[ri] <- ifelse(nrow(obs_17)>0, sum(obs_17$cover_cm2), NA)
    fc_pvso$cov18_obs[ri] <- ifelse(nrow(obs_17)>0, sum(obs_18$cover_cm2), NA)
    fc_pvso$cov19_obs[ri] <- ifelse(nrow(obs_17)>0, sum(obs_19$cover_cm2), NA)
    fc_pvso$cov20_obs[ri] <- ifelse(nrow(obs_17)>0, sum(obs_20$cover_cm2), NA)

    fc_pvso$n17_obs[ri] <- ifelse(nrow(obs_17)>0, nrow(obs_17), NA)
    fc_pvso$n18_obs[ri] <- ifelse(nrow(obs_17)>0, nrow(obs_18), NA)
    fc_pvso$n19_obs[ri] <- ifelse(nrow(obs_17)>0, nrow(obs_19), NA)
    fc_pvso$n20_obs[ri] <- ifelse(nrow(obs_17)>0, nrow(obs_20), NA)

    fc_pvso$cov18_pred[ri] <- ifelse(nrow(obs_17)>0, sum(pred_18$cover_cm2), NA)
    fc_pvso$cov19_pred[ri] <- ifelse(nrow(obs_17)>0, sum(pred_19$cover_cm2), NA)
    fc_pvso$cov20_pred[ri] <- ifelse(nrow(obs_17)>0, sum(pred_20$cover_cm2), NA)

    fc_pvso$n18_pred[ri] <- ifelse(nrow(obs_17)>0, nrow(pred_18), NA)
    fc_pvso$n19_pred[ri] <- ifelse(nrow(obs_17)>0, nrow(pred_19), NA)
    fc_pvso$n20_pred[ri] <- ifelse(nrow(obs_17)>0, nrow(pred_20), NA)


  }


}

write_csv(fc_pvso, "processed-data/finalcover_pred-vs-obs.csv")
```

We then calculated the log of ratio of final to initial total cover of each taxon in each turf (adding a small constant to avoid log(0) when a taxon go extinct), and plotted predicted versus observed values. We compared squared residuals of model predictions with those of a null-model expecting no changes in cover (i.e., a log ratio of zero). For downstream simulations, we only used taxa for which the ratio of IBM resuiduals to null-model residuals was < 0.8.

```
library(readr)
source("scripts/spp_modfits.R")

fc_pvso <- read_csv("processed-data/finalcover_pred-vs-obs.csv")
fc_pvso$site <- rep(c('Are', 'Bar', 'Cal', 'Nes'), each = length(domsp)*10)


fc_pvso$robs <- log((fc_pvso$cov20_obs + 1) / (fc_pvso$cov17_obs + 1))
fc_pvso$rpre <- log((fc_pvso$cov20_pred + 1) / (fc_pvso$cov17_obs + 1))
fc_pvso <- fc_pvso[!is.na(fc_pvso$robs),]
```

```r
pcols <- c('darkred', 'purple', 'lightblue', 'orange')

par(mfrow=c(5,5), mar = c(1.5,1.5,0,0), oma = c(3.25,3.25,1.25,1.25))
for (s in 1:length(domsp)){

        x <- subset(fc_pvso, species == domsp[s])

        plot(robs ~ rpre, x,
             col = pcols[as.integer(as.factor(x$site))],
             pch = 19, xlim = c(-8, 8), ylim = c(-8,8),
             xaxt = "n", yaxt = "n")


        if(s %in% c(1,6,11,16,21)) {
                axis(2, at = c(-5,0,5), labels = T)
        } else {axis(2, at = c(-5,0,5), labels = F)}

        if(s %in% c(21:25)) {
                axis(1, at = c(-5,0,5), labels = T)
        } else {axis(1, at = c(-5,0,5), labels = F)}


        abline(h = 0, lty = 3, col = "darkgray")
        abline(v = 0, lty = 3, col = "darkgray")

        abline(a=0,b=1, lty = 3)

        ss.res <- sum((x$robs - x$rpre)^2, na.rm = T)
        ss.tot <- sum((x$robs)^2, na.rm = T)

        rsq <- round(1 - (ss.res/ss.tot), 2)

        text(-5,6, as.character(rsq))
        mtext(domsp[s], cex = 0.65, font = ifelse(rsq > 0.2, 2, 1))


        if(s == 11) {
                mtext("Observed log cover ratio", 2, line = 3, cex = 1.25)
        }

        if(s == 23) {
                mtext("Predicted log cover ratio", 1, line = 3, cex = 1.25)
        }

        if(s == 24) {
                legend(12, -0.5, c("2,000", "1,600", "1,400", "1,000"),
                       pch = 19, col = pcols[c(3,2,4,1)], xpd = NA,
                       horiz = F, bty = "n", xjust = 0, cex = 1.25,
                       yjust = 0.5, title = "Elevation (m)")
        }

}
```

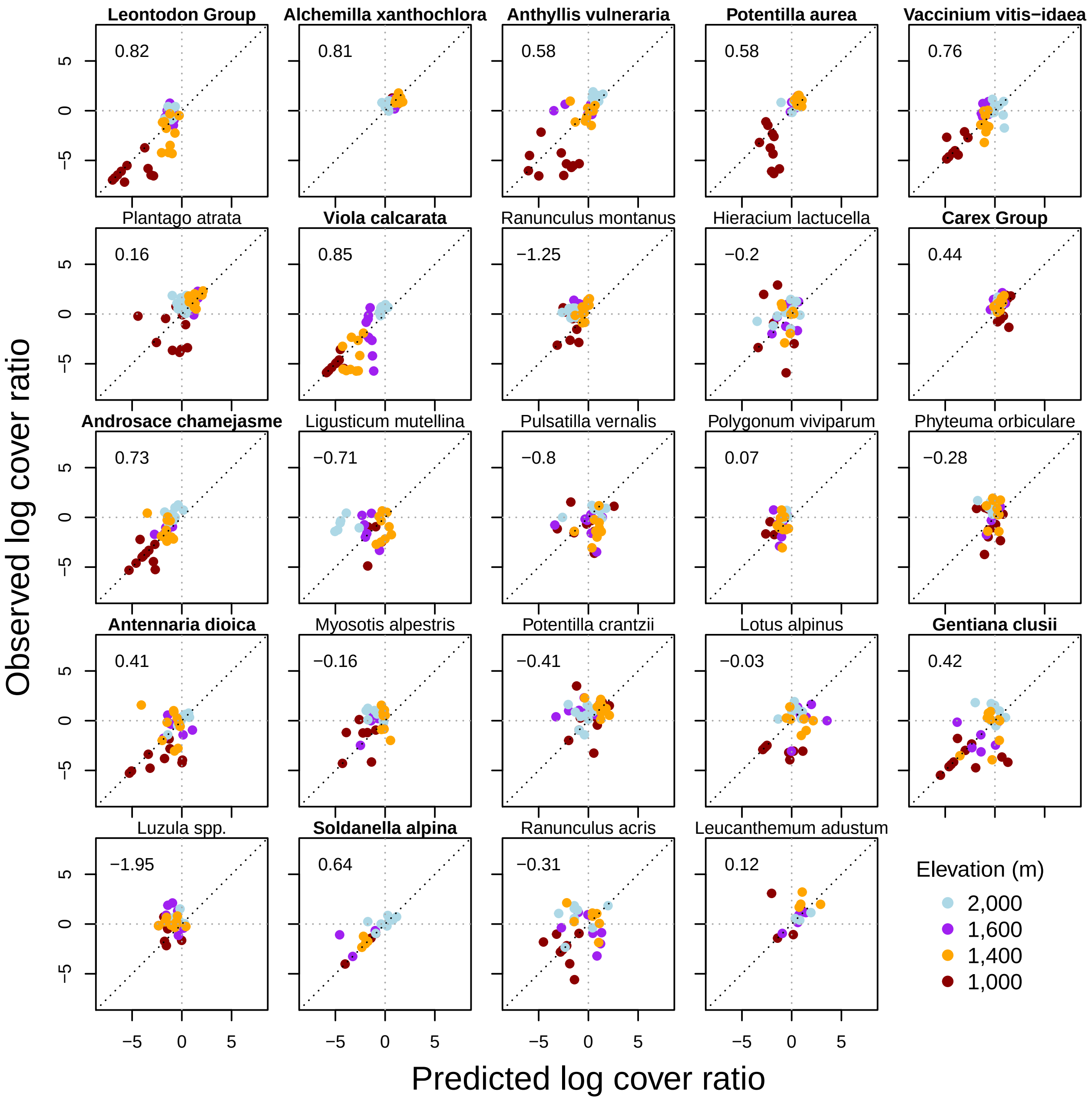

We simulated turf dynamics during the experimental period again, but this time including only the 11 taxa for which model predictions were acceptable.

```
library(rethinking)
library(dplyr)

source("scripts/spp_simul.R")
source("scripts/ClimDemoSim_oneturf.R")

transturfs <- levels(factor(anbc$plot_id))[1:40]

for (tt in 1:40){
  simdat_t1 <- subset(anbc, plot_id == transturfs[tt] & year == 2017)
  simdat_t1 <- subset(simdat_t1, species %in% spnames)
```

```
  simdat_t1 <- select(simdat_t1,
                      species, x, y, year, cover_cm2, quadrant_id, site, plot_id)
  simdat_t1$unique_id <- paste("t1_", 1:nrow(simdat_t1), sep = "")
  simdat_t1$species <- as.character(simdat_t1$species)
  simdat_t1$site <- as.character(simdat_t1$site)
  simdat_t1$plot_id <- as.character(simdat_t1$plot_id)

  allsims <- simdat_t1

  nyears = 2020 - simdat_t1$year[1]

  for (i in 1:nyears){

    simdat_t2 <- simdat_t1[0,]

    for (s in spnames) {
      sp_t2 <- ClimDemoSim_oneturf(s, simdat = simdat_t1, meanpars = T,
                                   obsclim = T, final_community = T)
      simdat_t2 <- rbind(simdat_t2, sp_t2)
      rm(sp_t2)
    }

    simdat_t2$year[is.na(simdat_t2$year)] <- max(simdat_t2$year, na.rm = T)

    simdat_t2$species <- as.character(simdat_t2$species)
    simdat_t2$quadrant_id <- as.character(simdat_t2$quadrant_id)
    simdat_t2$unique_id <- 1:nrow(simdat_t2)


    allsims <- rbind(allsims, simdat_t2)
    simdat_t1 <- simdat_t2
  }

  save_path <- paste("simulations/single_turf/sims_",
                     transturfs[tt], "_meanpars_11sp.csv", sep = "")
  write.csv(allsims, save_path)
}



fc_pvso <- data.frame(plot = rep(transturfs, each = length(spnames)),
                      species = rep(spnames, 40))


fc_pvso$cov17_obs <- numeric(nrow(fc_pvso))
fc_pvso$cov18_obs <- numeric(nrow(fc_pvso))
fc_pvso$cov19_obs <- numeric(nrow(fc_pvso))
fc_pvso$cov20_obs <- numeric(nrow(fc_pvso))

fc_pvso$n17_obs <- numeric(nrow(fc_pvso))
fc_pvso$n18_obs <- numeric(nrow(fc_pvso))
fc_pvso$n19_obs <- numeric(nrow(fc_pvso))
fc_pvso$n20_obs <- numeric(nrow(fc_pvso))
```

```r
fc_pvso$cov18_pred <- numeric(nrow(fc_pvso))
fc_pvso$cov19_pred <- numeric(nrow(fc_pvso))
fc_pvso$cov20_pred <- numeric(nrow(fc_pvso))

fc_pvso$n18_pred <- numeric(nrow(fc_pvso))
fc_pvso$n19_pred <- numeric(nrow(fc_pvso))
fc_pvso$n20_pred <- numeric(nrow(fc_pvso))

for(turf in transturfs){

  predat <- read.csv( paste("simulations/single_turf/sims_", turf, "_meanpars_11sp.csv",
                            sep = ""))

  for(sp in domsp) {
    obs_17 <- subset(anbc, plot_id == turf & species == sp & year == 2017)
    obs_18 <- subset(anbc, plot_id == turf & species == sp & year == 2018)
    obs_19 <- subset(anbc, plot_id == turf & species == sp & year == 2019)
    obs_20 <- subset(anbc, plot_id == turf & species == sp & year == 2020)

    pred_18 <- subset(predat, plot_id == turf & species == sp & year == 2018)
    pred_19 <- subset(predat, plot_id == turf & species == sp & year == 2019)
    pred_20 <- subset(predat, plot_id == turf & species == sp & year == 2020)

    pred_18$covcat <- ifelse(pred_18$cover_cm2 < 1.5625, 1.5625,
                             ifelse(pred_18$cover_cm2 < 3.125, 3.125,
                                    ifelse(pred_18$cover_cm2 < 6.25, 6.25,
                                           ifelse(pred_18$cover_cm2 < 12.5, 12.5,
                                                  ifelse(pred_18$cover_cm2 < 18.75, 18.75,
                                                         ifelse(pred_18$cover_cm2<25, 25,
                                                                pred_18$cover_cm2))))))

    pred_19$covcat <- ifelse(pred_19$cover_cm2 < 1.5625, 1.5625,
                             ifelse(pred_19$cover_cm2 < 3.125, 3.125,
                                    ifelse(pred_19$cover_cm2 < 6.25, 6.25,
                                           ifelse(pred_19$cover_cm2 < 12.5, 12.5,
                                                  ifelse(pred_19$cover_cm2 < 18.75, 18.75,
                                                         ifelse(pred_19$cover_cm2 <25,25,
                                                                pred_19$cover_cm2))))))

    pred_20$covcat <- ifelse(pred_20$cover_cm2 < 1.5625, 1.5625,
                             ifelse(pred_20$cover_cm2 < 3.125, 3.125,
                                    ifelse(pred_20$cover_cm2 < 6.25, 6.25,
                                           ifelse(pred_20$cover_cm2 < 12.5, 12.5,
                                                  ifelse(pred_20$cover_cm2 < 18.75, 18.75,
                                                         ifelse(pred_20$cover_cm2<25, 25,
                                                                pred_20$cover_cm2))))))


    ri <- which(fc_pvso$plot == turf & fc_pvso$species == sp)


    fc_pvso$cov17_obs[ri] <- ifelse(nrow(obs_17)>0, sum(obs_17$cover_cm2), NA)
    fc_pvso$cov18_obs[ri] <- ifelse(nrow(obs_17)>0, sum(obs_18$cover_cm2), NA)
```

```
    fc_pvso$cov19_obs[ri] <- ifelse(nrow(obs_17)>0, sum(obs_19$cover_cm2), NA)
    fc_pvso$cov20_obs[ri] <- ifelse(nrow(obs_17)>0, sum(obs_20$cover_cm2), NA)

    fc_pvso$n17_obs[ri] <- ifelse(nrow(obs_17)>0, nrow(obs_17), NA)
    fc_pvso$n18_obs[ri] <- ifelse(nrow(obs_17)>0, nrow(obs_18), NA)
    fc_pvso$n19_obs[ri] <- ifelse(nrow(obs_17)>0, nrow(obs_19), NA)
    fc_pvso$n20_obs[ri] <- ifelse(nrow(obs_17)>0, nrow(obs_20), NA)

    fc_pvso$cov18_pred[ri] <- ifelse(nrow(obs_17)>0, sum(pred_18$cover_cm2), NA)
    fc_pvso$cov19_pred[ri] <- ifelse(nrow(obs_17)>0, sum(pred_19$cover_cm2), NA)
    fc_pvso$cov20_pred[ri] <- ifelse(nrow(obs_17)>0, sum(pred_20$cover_cm2), NA)

    fc_pvso$n18_pred[ri] <- ifelse(nrow(obs_17)>0, nrow(pred_18), NA)
    fc_pvso$n19_pred[ri] <- ifelse(nrow(obs_17)>0, nrow(pred_19), NA)
    fc_pvso$n20_pred[ri] <- ifelse(nrow(obs_17)>0, nrow(pred_20), NA)


  }


}

write_csv(fc_pvso, "processed-data/finalcover_pred-vs-obs-11_FC.csv")
```

To compare the trajectories of compositional change predicted by our models with those observed in the experiment, we performed a Principal Coordinates Analysis based on Euclidean distances of all observed and simulated communities across years.

```
##
anbc <- read_csv('processed-data/anbc_clean.csv')
source('scripts/spp_simul.R')

vegdata <- subset(anbc, species %in% spnames)
vegdata$species <- factor(vegdata$species, levels = spnames)


oldsites <- subset(vegdata, !(site %in% c("Pad", "Neu")))
oldsites <- subset(oldsites, is.na(oldsites$appendix))
oldsites$plot_id <- factor(oldsites$plot_id)


## Observed dynamics
vegtab <- data.frame(species = levels(oldsites$species))

for(t in levels(factor(oldsites$plot_id))) {

  x17 <- subset(oldsites, year == 2017 & plot_id == t)
  x18 <- subset(oldsites, year == 2018 & plot_id == t)
  x19 <- subset(oldsites, year == 2019 & plot_id == t)
  x20 <- subset(oldsites, year == 2020 & plot_id == t)
  vegtab <- cbind(vegtab,
                  tapply(x17$cover_cm2, x17$species, sum, na.rm = T),
                  tapply(x18$cover_cm2, x18$species, sum, na.rm = T),
                  tapply(x19$cover_cm2, x19$species, sum, na.rm = T),
```

```
                  tapply(x20$cover_cm2, x20$species, sum, na.rm = T))

  rm(x17, x18, x19, x20)
}
rm(t)

## Predicted dynamics
vegtab_pred <- data.frame(species = levels(oldsites$species))

for(t in levels(factor(oldsites$plot_id))) {

  x <- read.csv( paste("simulations/single_turfs/sims_", t, "_meanpars_11sp.csv",
                       sep = ""))
  x$species <- factor(x$species, levels = spnames)
  x17 <- subset(x, year == 2017 & plot_id == t)
  x18 <- subset(x, year == 2018 & plot_id == t)
  x19 <- subset(x, year == 2019 & plot_id == t)
  x20 <- subset(x, year == 2020 & plot_id == t)
  vegtab_pred <- cbind(vegtab_pred,
                       tapply(x17$cover_cm2, x17$species, sum, na.rm = T),
                       tapply(x18$cover_cm2, x18$species, sum, na.rm = T),
                       tapply(x19$cover_cm2, x19$species, sum, na.rm = T),
                       tapply(x20$cover_cm2, x20$species, sum, na.rm = T))

  rm(x17, x18, x19, x20)
}
rm(t)

turfs <- data.frame(turf = rep(levels(as.factor(oldsites$plot_id)), each = 4),
                    year = rep(2017:2020, 40),
                    site = rep(c("Are", "Bar", "Cal", "Nes"), each = 40))

turfs$site <- factor(turfs$site, levels = c('Are', 'Nes', 'Bar', 'Cal'))

vegtab_full <- cbind(vegtab, vegtab_pred[,-1])

# Transform the data frame with species abundances into a matrix, transpose the matrix
vegmatrix <- data.matrix (vegtab_full[,-1])
rownames (vegmatrix) <- vegtab_full$species
vegmatrix <- t (vegmatrix)
vegmatrix[is.na(vegmatrix)] <- 0

saveRDS(vegmatrix, 'processed-data/vegmatrix_pvso-11.RDS')
```

Figure 1 in the main text of Block et al. (2022) shows a comparison of observed and predicted trajectories of community composition.

```
library(ecodist)
library(vegan)

## Ordination trajectories
vegmatrix <- readRDS("processed-data/vegmatrix_pvso-11.rds")

DM <- distance(vegmatrix, method="euclidean")
```

```r
PCO <- pco(DM)
axis1 <- PCO$vectors[,1] * PCO$values[1]
axis2 <- PCO$vectors[,2] * PCO$values[2]

species.arrows <- vf(PCO$vectors[,1:2],vegmatrix,nperm=0)

anbc <- read_csv("processed-data/anbc_clean.csv")
oldsites <- subset(anbc, !(site %in% c("Pad", "Neu")))
oldsites <- subset(oldsites, is.na(oldsites$appendix))
oldsites$plot_id <- factor(oldsites$plot_id)
turfs <- data.frame(turf = rep(levels(as.factor(oldsites$plot_id)), each = 4),
                    year = rep(2017:2020, 40),
                    site = rep(c("Are", "Bar", "Cal", "Nes"), each = 40))

turfs$site <- factor(turfs$site, levels = c('Are', 'Nes', 'Bar', 'Cal'))


par(mfrow = c(2,2), mar = c(1,1,1.2,0), oma = c(3.5,3.5,0.5,0.5))

# 2000 m
plot(axis1, axis2,
     xlab="",
     ylab="",
     type = "n", xaxt = "n")
mtext("2000 m")
mtext("(A)", 3, adj = 0, font = 2)
axis(1, at = c(-1e3, 0, 1e3, 2e3, 3e3), labels = rep("", 5))

## Data
arrows(x0 = axis1[turfs$year == "2017" & turfs$site == "Cal"],
       y0 = axis2[turfs$year == "2017" & turfs$site == "Cal"],
       x1 = axis1[which(turfs$year == "2020" & turfs$site == "Cal")],
       y1 = axis2[which(turfs$year == "2020" & turfs$site == "Cal")],
       col = "black", lwd = 1,
       length = 0.05, lty = 1)

## Model
arrows(x0 = axis1[which(turfs$year == "2017" & turfs$site == "Cal")],
       y0 = axis2[which(turfs$year == "2017" & turfs$site == "Cal")],
       x1 = axis1[which(turfs$year == "2020" & turfs$site == "Cal") + 160],
       y1 = axis2[which(turfs$year == "2020" & turfs$site == "Cal") + 160],
       col = "darkgray", lwd = 1,
       length = 0.05, lty = 1)



# 1600 m
plot(axis1, axis2,
     xlab="",
     ylab="",
     type = "n", yaxt = "n", xaxt = "n")
mtext("1600 m")
mtext("(B)", 3, adj = 0, font = 2)
```

```r
axis(1, at = c(-1e3, 0, 1e3, 2e3, 3e3), labels = rep("", 5))
axis(2, at = c(-3e3, -2e3, -1e3, 0, 1e3), labels = rep("", 5))

arrows(x0 = axis1[turfs$year == "2017" & turfs$site == "Bar"],
       y0 = axis2[turfs$year == "2017" & turfs$site == "Bar"],
       x1 = axis1[which(turfs$year == "2020" & turfs$site == "Bar")],
       y1 = axis2[which(turfs$year == "2020" & turfs$site == "Bar")],
       col = "black",
       length = 0.05, lty = 1)


arrows(x0 = axis1[turfs$year == "2017" & turfs$site == "Bar"],
       y0 = axis2[turfs$year == "2017" & turfs$site == "Bar"],
       x1 = axis1[which(turfs$year == "2020" & turfs$site == "Bar") + 160],
       y1 = axis2[which(turfs$year == "2020" & turfs$site == "Bar") + 160],
       col = "darkgray",
       length = 0.05, lty = 1)


plot(axis1, axis2,
     xlab="",
     ylab="",
     type = "n")
mtext("1400 m")
mtext("(C)", 3, adj = 0, font = 2)

mtext("Axis 2 (26.3%)", 2, line = 3, adj = 1.5, cex = 1.25)
mtext("Axis 1 (44.2%)", 1, line = 2.75, adj = 1.35, cex = 1.25)

arrows(x0 = axis1[turfs$year == "2017" & turfs$site == "Nes"],
       y0 = axis2[turfs$year == "2017" & turfs$site == "Nes"],
       x1 = axis1[which(turfs$year == "2020" & turfs$site == "Nes")],
       y1 = axis2[which(turfs$year == "2020" & turfs$site == "Nes")],
       col = "black",
       length = 0.05, lty = 1)


arrows(x0 = axis1[turfs$year == "2017" & turfs$site == "Nes"],
       y0 = axis2[turfs$year == "2017" & turfs$site == "Nes"],
       x1 = axis1[which(turfs$year == "2020" & turfs$site == "Nes") + 160],
       y1 = axis2[which(turfs$year == "2020" & turfs$site == "Nes") + 160],
       col = "darkgray",
       length = 0.05, lty = 1)



plot(axis1, axis2,
     xlab="",
     ylab="",
     type = "n", yaxt = "n")
mtext("1000 m")
mtext("(D)", 3, adj = 0, font = 2)
axis(2, at = c(-3e3, -2e3, -1e3, 0, 1e3), labels = rep("", 5))
```

```r
arrows(x0 = axis1[turfs$year == "2017" & turfs$site == "Are"],
       y0 = axis2[turfs$year == "2017" & turfs$site == "Are"],
       x1 = axis1[which(turfs$year == "2020" & turfs$site == "Are")],
       y1 = axis2[which(turfs$year == "2020" & turfs$site == "Are")],
       col = "black",
       length = 0.05, lty = 1)


arrows(x0 = axis1[turfs$year == "2017" & turfs$site == "Are"],
       y0 = axis2[turfs$year == "2017" & turfs$site == "Are"],
       x1 = axis1[which(turfs$year == "2020" & turfs$site == "Are") + 160],
       y1 = axis2[which(turfs$year == "2020" & turfs$site == "Are") + 160],
       col = "darkgray",
       length = 0.05, lty = 1)


plot.vf(species.arrows[species.arrows[,"r"]>0.2,], length = 0.05,
        col = rgb(0,0.5,0,0.5), lwd = 1.5, lty = 1, cex = 0.75)
```

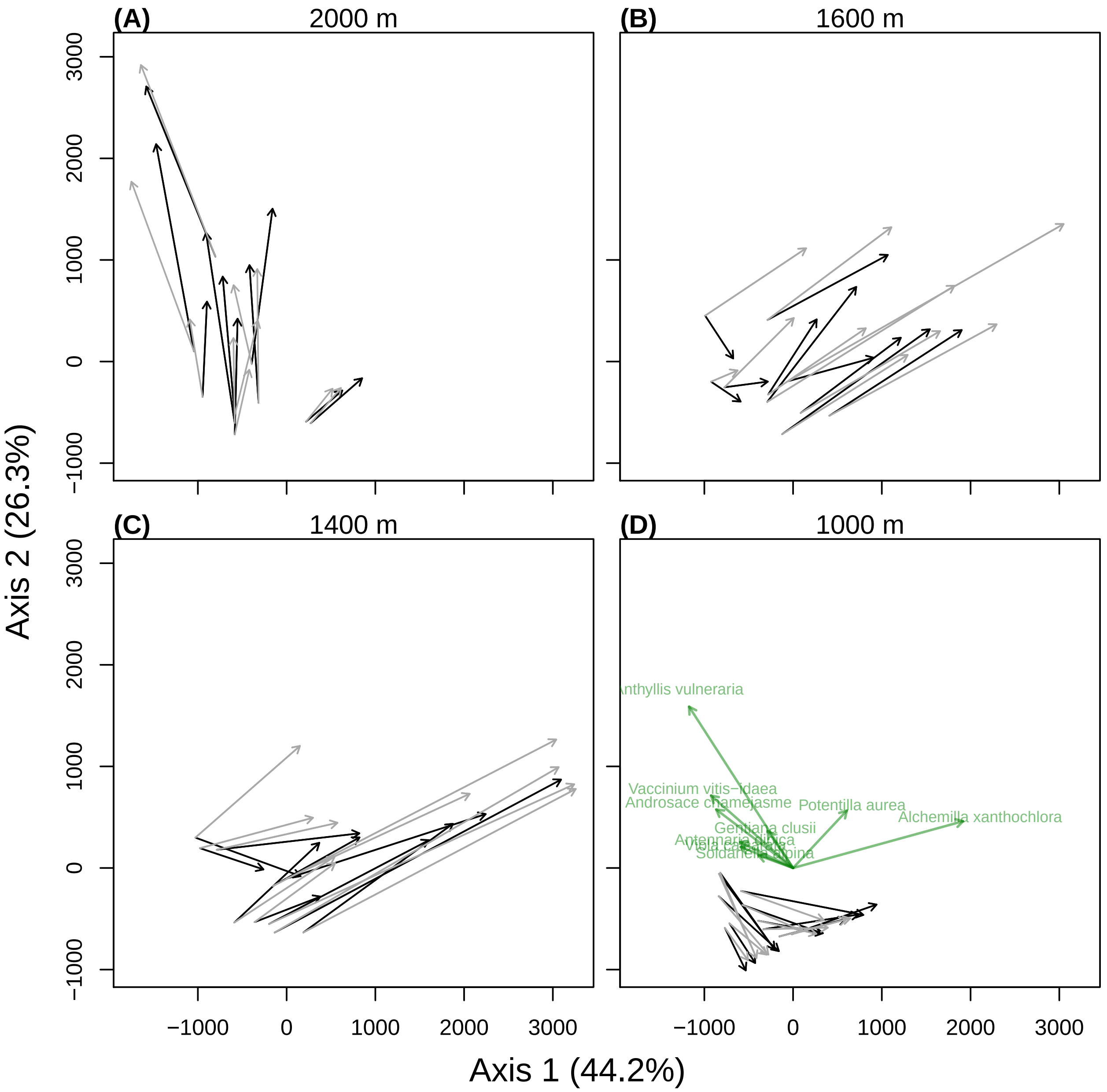

As well as the trajectories of observed vs predicted Shannon diversity averaged across all the turfs at each elevation.

```r
## Diversity trajectories
shannon <- function(x) {
        s.i <- x[x > 0]
        p.i <- s.i/sum(s.i)
        return(-sum(p.i*log(p.i)))
}


h_obs <- numeric(nrow(turfs))
h_pre <- numeric(nrow(turfs))

for(i in 1:nrow(turfs)){
```

```
        h_obs[i] <- shannon(vegmatrix[i,])
        h_pre[i] <- shannon(vegmatrix[160+i,])
}


par(mar = c(2.5,2.5,1,1),  cex.axis = 1.2, cex.lab = 1.2, las = 1)

plot(2017:2020, ylim = c(0,2), type = "n", ylab = "Shannon diversity",
     xlab = "Year", xlim = c(2017, 2020), xaxt = "n", yaxt = "n")
axis(1, at = c(2017, 2018, 2019, 2020), labels = T)
axis(2, at = c(0, 1, 2), labels = T)

lines(2017:2020,
      c(mean(h_obs[which(turfs$site == "Are" & turfs$year==2017)]),
        mean(h_obs[which(turfs$site == "Are" & turfs$year==2018)]),
        mean(h_obs[which(turfs$site == "Are" & turfs$year==2019)]),
        mean(h_obs[which(turfs$site == "Are" & turfs$year==2020)])),
      lwd = 2)

lines(2017:2020,
      c(mean(h_pre[which(turfs$site == "Are" & turfs$year==2017)]),
        mean(h_pre[which(turfs$site == "Are" & turfs$year==2018)]),
        mean(h_pre[which(turfs$site == "Are" & turfs$year==2019)]),
        mean(h_pre[which(turfs$site == "Are" & turfs$year==2020)])),
      col = "darkgray", lwd = 2)
```

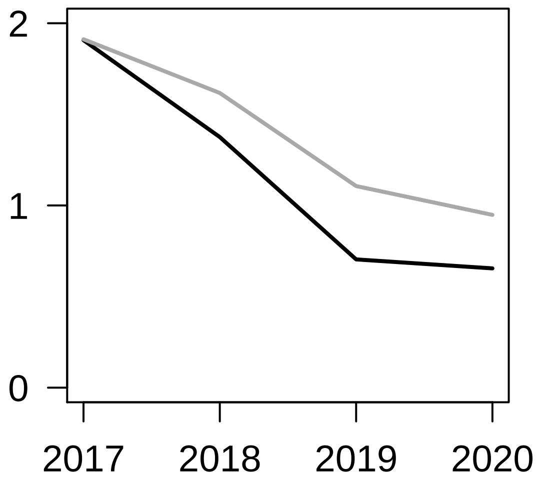


```
plot(2017:2020, ylim = c(0,2), type = "n", ylab = "Shannon diversity",
     xlab = "Year", xlim = c(2017, 2020), xaxt = "n", yaxt = "n")
axis(1, at = c(2017, 2018, 2019, 2020), labels = T)
axis(2, at = c(0, 1, 2), labels = T)

lines(2017:2020,
      c(mean(h_obs[which(turfs$site == "Nes" & turfs$year==2017)]),
        mean(h_obs[which(turfs$site == "Nes" & turfs$year==2018)]),
        mean(h_obs[which(turfs$site == "Nes" & turfs$year==2019)]),
        mean(h_obs[which(turfs$site == "Nes" & turfs$year==2020)])),
      lwd = 2)
```

```r
lines(2017:2020,
      c(mean(h_pre[which(turfs$site == "Nes" & turfs$year==2017)]),
        mean(h_pre[which(turfs$site == "Nes" & turfs$year==2018)]),
        mean(h_pre[which(turfs$site == "Nes" & turfs$year==2019)]),
        mean(h_pre[which(turfs$site == "Nes" & turfs$year==2020)])),
      col = "darkgray", lwd = 2)
```

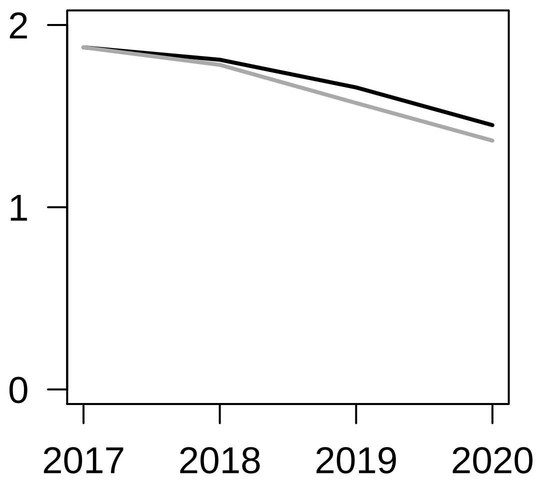


```r
plot(2017:2020, ylim = c(0,2), type = "n", ylab = "Shannon diversity",
     xlab = "Year", xlim = c(2017, 2020), xaxt = "n", yaxt = "n")
axis(1, at = c(2017, 2018, 2019, 2020), labels = T)
axis(2, at = c(0, 1, 2), labels = T)

lines(2017:2020,
      c(mean(h_obs[which(turfs$site == "Bar" & turfs$year==2017)]),
        mean(h_obs[which(turfs$site == "Bar" & turfs$year==2018)]),
        mean(h_obs[which(turfs$site == "Bar" & turfs$year==2019)]),
        mean(h_obs[which(turfs$site == "Bar" & turfs$year==2020)])),
      lwd = 2)


lines(2017:2020,
      c(mean(h_pre[which(turfs$site == "Bar" & turfs$year==2017)]),
        mean(h_pre[which(turfs$site == "Bar" & turfs$year==2018)]),
        mean(h_pre[which(turfs$site == "Bar" & turfs$year==2019)]),
        mean(h_pre[which(turfs$site == "Bar" & turfs$year==2020)])),
      col = "darkgray", lwd = 2)
```

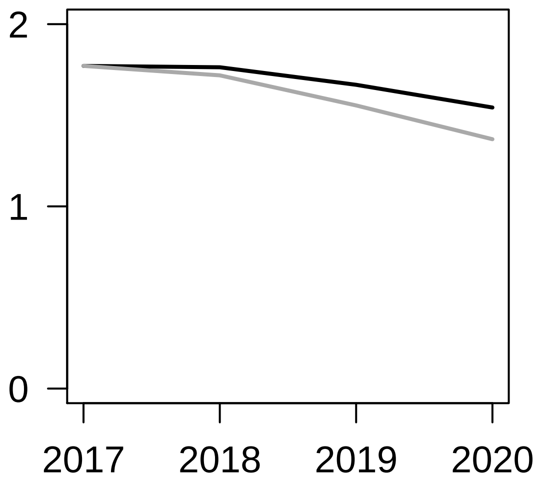


```r
plot(2017:2020, ylim = c(0,2), type = "n", ylab = "Shannon diversity",
     xlab = "Year", xlim = c(2017, 2020), xaxt = "n", yaxt = "n")
axis(1, at = c(2017, 2018, 2019, 2020), labels = T)
axis(2, at = c(0, 1, 2), labels = T)

lines(2017:2020,
      c(mean(h_obs[which(turfs$site == "Cal" & turfs$year==2017)]),
        mean(h_obs[which(turfs$site == "Cal" & turfs$year==2018)]),
        mean(h_obs[which(turfs$site == "Cal" & turfs$year==2019)]),
        mean(h_obs[which(turfs$site == "Cal" & turfs$year==2020)])),
      lwd = 2)


lines(2017:2020,
      c(mean(h_pre[which(turfs$site == "Cal" & turfs$year==2017)]),
        mean(h_pre[which(turfs$site == "Cal" & turfs$year==2018)]),
        mean(h_pre[which(turfs$site == "Cal" & turfs$year==2019)]),
        mean(h_pre[which(turfs$site == "Cal" & turfs$year==2020)])),
      col = "darkgray", lwd = 2)
```

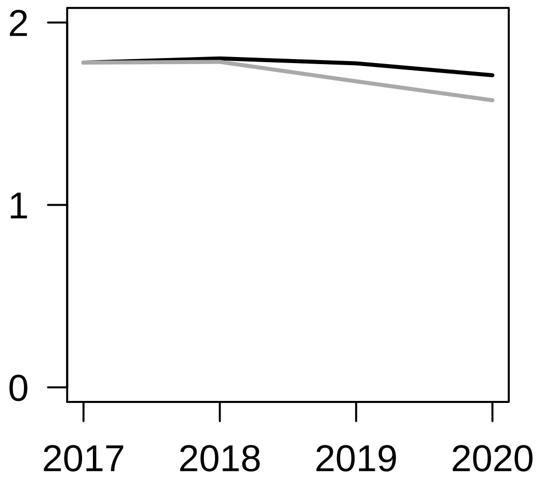

3.3 Simulated dynamics under future climate change

3.3.1 Swiss Climate Change Scenarios

We used climate forecast data from the CH2018 Climate Change Scenarios for Switzerland developed by ETH Zürich and MeteoSchweiz. In particular, we used the daily weather time series at the local scale (DAILY-LOCAL) for the weather station in Chur (the same weather station that we used to predict weather conditions across the elevational gradient).

These datasets include simulations of three, contrasting climate futures corresponding to different greenhouse emission pathways. These scenarios, adopted by the IPCC fifth report, are called Representative Concentration Pathways (RCPs), and range from optimistic (RCP 2.6), realistic, based on current trends (RCP 4.5), and pessimistic (RCP 8.5).

We used the following code to load and average the forecasts of the different models included in the CH2018 simulations.

```
library(stringr)
library(readr)

# Get Chur data
## Air temp
models <- list.files("data/abiotic/forecast/temp")
for (m in models) {

        m2 <- str_replace(m, "csv", "CHU")
        fpath <- paste("data/abiotic/forecast/temp/", m, "/", m2, ".csv", sep = "")

        x <- read_delim(fpath, ";", escape_double = FALSE,
                        trim_ws = TRUE, skip = 16)

        names(x) <- c("date", "temp")

        fpath2 <- paste("data/abiotic/forecast/chur/", m2, ".csv", sep = "")

        write_csv(x, fpath2)

        rm(fpath, fpath2)
}

## Rain
models <- list.files("data/abiotic/forecast/rain")
for (m in models) {

        m2 <- str_replace(m, "csv", "CHU")
        fpath <- paste("data/abiotic/forecast/rain/", m, "/", m2, ".csv", sep = "")

        x <- read_delim(fpath, ";", escape_double = FALSE,
                        trim_ws = TRUE, skip = 16)

        names(x) <- c("date", "rain")

        fpath2 <- paste("data/abiotic/forecast/chur/", m2, ".csv", sep = "")

        write_csv(x, fpath2)
```

```r
        rm(fpath, fpath2)
}

## Relative humidity
models <- list.files("data/abiotic/forecast/rh")
for (m in models) {

        m2 <- str_replace(m, "csv", "CHU")
        fpath <- paste("data/abiotic/forecast/rh/", m, "/", m2, ".csv", sep = "")

        x <- read_delim(fpath, ";", escape_double = FALSE,
                        trim_ws = TRUE, skip = 16)

        names(x) <- c("date", "rh")

        fpath2 <- paste("data/abiotic/forecast/chur/", m2, ".csv", sep = "")

        write_csv(x, fpath2)

        rm(fpath, fpath2)
}


# Get model averages by RCP scenario
models <- list.files("data/abiotic/forecast/chur")
temp_models <- models[grep('_tas_', models, fixed = T)]
rain_models <- models[grep('_pr_', models, fixed = T)]
rh_models <- models[grep('_hurs_', models, fixed = T)]

## RCP 2.6
temp_mod_rcp26 <-  temp_models[grep('_RCP26_', temp_models, fixed = T)]
temp_rcp26 <- numeric()
for (m in temp_mod_rcp26){

        fpath <- paste("data/abiotic/forecast/chur/", m, sep = "")
        x <- read_csv(fpath)
        temp_rcp26 <- cbind(temp_rcp26, x$temp)
        rm(x, fpath)
}
temp_rcp26_mu <- apply(temp_rcp26, 1, mean, na.rm = T)

rain_mod_rcp26 <-  rain_models[grep('_RCP26_', rain_models, fixed = T)]
rain_rcp26 <- numeric()
for (m in rain_mod_rcp26){

        fpath <- paste("data/abiotic/forecast/chur/", m, sep = "")
        x <- read_csv(fpath)
        rain_rcp26 <- cbind(rain_rcp26, x$rain)
        rm(x, fpath)
}
rain_rcp26_mu <- apply(rain_rcp26, 1, mean, na.rm = T)

rh_mod_rcp26 <-  rh_models[grep('_RCP26_', rh_models, fixed = T)]
```

```r
rh_rcp26 <- numeric()
for (m in rh_mod_rcp26){

        fpath <- paste("data/abiotic/forecast/chur/", m, sep = "")
        x <- read_csv(fpath)
        rh_rcp26 <- cbind(rh_rcp26, x$rh)
        rm(x, fpath)
}
rh_rcp26_mu <- apply(rh_rcp26, 1, mean, na.rm = T)

rcp26 <- data.frame(temp = temp_rcp26_mu,
                    rain = rain_rcp26_mu,
                    rh = rh_rcp26_mu)

fpath <- paste("data/abiotic/forecast/chur/", temp_mod_rcp26[1], sep = "")
x <- read_csv(fpath)
rcp26$date <- x$date
rm(x, rain_rcp26, rh_rcp26, temp_rcp26)

write_csv(rcp26, "processed-data/rcp26.csv")


## RCP 4.5
temp_mod_rcp45 <-  temp_models[grep('_RCP45_', temp_models, fixed = T)]
temp_rcp45 <- numeric()
for (m in temp_mod_rcp45){

        fpath <- paste("data/abiotic/forecast/chur/", m, sep = "")
        x <- read_csv(fpath)
        temp_rcp45 <- cbind(temp_rcp45, x$temp)
        rm(x, fpath)
}
temp_rcp45_mu <- apply(temp_rcp45, 1, mean, na.rm = T)

rain_mod_rcp45 <-  rain_models[grep('_RCP45_', rain_models, fixed = T)]
rain_rcp45 <- numeric()
for (m in rain_mod_rcp45){

        fpath <- paste("data/abiotic/forecast/chur/", m, sep = "")
        x <- read_csv(fpath)
        rain_rcp45 <- cbind(rain_rcp45, x$rain)
        rm(x, fpath)
}
rain_rcp45_mu <- apply(rain_rcp45, 1, mean, na.rm = T)

rh_mod_rcp45 <-  rh_models[grep('_RCP45_', rh_models, fixed = T)]
rh_rcp45 <- numeric()
for (m in rh_mod_rcp45){

        fpath <- paste("data/abiotic/forecast/chur/", m, sep = "")
        x <- read_csv(fpath)
        rh_rcp45 <- cbind(rh_rcp45, x$rh)
        rm(x, fpath)
```

```
}
rh_rcp45_mu <- apply(rh_rcp45, 1, mean, na.rm = T)

rcp45 <- data.frame(temp = temp_rcp45_mu,
                    rain = rain_rcp45_mu,
                    rh = rh_rcp45_mu)

fpath <- paste("data/abiotic/forecast/chur/", temp_mod_rcp45[1], sep = "")
x <- read_csv(fpath)
rcp45$date <- x$date
rm(x, rain_rcp45, rh_rcp45, temp_rcp45)

write_csv(rcp45, "processed-data/rcp45.csv")


## RCP 8.5
temp_mod_rcp85 <-  temp_models[grep('_RCP85_', temp_models, fixed = T)]
temp_rcp85 <- numeric()
for (m in temp_mod_rcp85){

        fpath <- paste("data/abiotic/forecast/chur/", m, sep = "")
        x <- read_csv(fpath)
        temp_rcp85 <- cbind(temp_rcp85, x$temp)
        rm(x, fpath)
}
temp_rcp85_mu <- apply(temp_rcp85, 1, mean, na.rm = T)

rain_mod_rcp85 <-  rain_models[grep('_RCP85_', rain_models, fixed = T)]
rain_rcp85 <- numeric()
for (m in rain_mod_rcp85){

        fpath <- paste("data/abiotic/forecast/chur/", m, sep = "")
        x <- read_csv(fpath)
        rain_rcp85 <- cbind(rain_rcp85, x$rain)
        rm(x, fpath)
}
rain_rcp85_mu <- apply(rain_rcp85, 1, mean, na.rm = T)

rh_mod_rcp85 <-  rh_models[grep('_RCP85_', rh_models, fixed = T)]
rh_rcp85 <- numeric()
for (m in rh_mod_rcp85){

        fpath <- paste("data/abiotic/forecast/chur/", m, sep = "")
        x <- read_csv(fpath)
        rh_rcp85 <- cbind(rh_rcp85, x$rh)
        rm(x, fpath)
}
rh_rcp85_mu <- apply(rh_rcp85, 1, mean, na.rm = T)

rcp85 <- data.frame(temp = temp_rcp85_mu,
                    rain = rain_rcp85_mu,
                    rh = rh_rcp85_mu)
```

```r
fpath <- paste("data/abiotic/forecast/chur/", temp_mod_rcp85[1], sep = "")
x <- read_csv(fpath)
rcp85$date <- x$date
rm(x, rain_rcp85, rh_rcp85, temp_rcp85)

write_csv(rcp85, "processed-data/rcp85.csv")
```

Now, we can use these simulated daily time series to predict weather conditions at the 2000 m site, where we will simulate dynamics over the next decades. However, when comparing the measured and simulated values for weather conditions in Chur, we noticed that there were some consistent biases in the simulated data. For example, CH2018 simulated temperatures were consistently colder than recorded temperatures during the experimental period.

```r
### Part 2: predict soil moisture and air temp at Calanda site
rcp26 <- read_csv("processed-data/rcp26.csv")
rcp45 <- read_csv("processed-data/rcp45.csv")
rcp85 <- read_csv("processed-data/rcp85.csv")

## Compare Chur weather in simulated and measured data
wsdata <- read_csv("processed-data/calanda_predicted_temps.csv")

## Subset summers
library(lubridate)
summers_rcp26 = rcp26[month(rcp26$date) %in% c(6,7,8), ]
summers_rcp45 = rcp45[month(rcp45$date) %in% c(6,7,8), ]
summers_rcp85 = rcp85[month(rcp85$date) %in% c(6,7,8), ]

par (mar = c(4,6,1,1))
dens(wsdata$temp[wsdata$date %in% summers_rcp26$date] -
             summers_rcp26$temp[summers_rcp26$date %in% wsdata$date],
     col = col.alpha('blue',0.8), ylim = c(0,0.15), lwd = 1.5,
     main = "", xlab = "Temperature difference (ºC)")
mtext("Difference between recorded and CH2018 simulated
      daily temperature during summer (ºC)", cex = 0.85, adj = 0)
dens(wsdata$temp[wsdata$date %in% summers_rcp45$date] -
             summers_rcp45$temp[summers_rcp45$date %in% wsdata$date],
     add = T, col = col.alpha('orange',0.8), lwd = 1.5)
dens(wsdata$temp[wsdata$date %in% summers_rcp85$date] -
             summers_rcp85$temp[summers_rcp85$date %in% wsdata$date],
     add = T, col = col.alpha('darkred',0.8), lwd = 1.5)
abline(v = 0, lty = 3, lwd = 3)
abline(v = mean(wsdata$temp[wsdata$date %in% summers_rcp26$date] -
                        summers_rcp26$temp[summers_rcp26$date %in% wsdata$date]),
       lty = 3, lwd = 3, col = col.alpha('blue',0.8))
abline(v = mean(wsdata$temp[wsdata$date %in% summers_rcp45$date] -
                        summers_rcp45$temp[summers_rcp45$date %in% wsdata$date]),
       lty = 3, lwd = 3, col = col.alpha('orange',0.8))
abline(v = mean(wsdata$temp[wsdata$date %in% summers_rcp85$date] -
                        summers_rcp85$temp[summers_rcp85$date %in% wsdata$date]),
       lty = 3, lwd = 3, col = col.alpha('darkred',0.8))
```

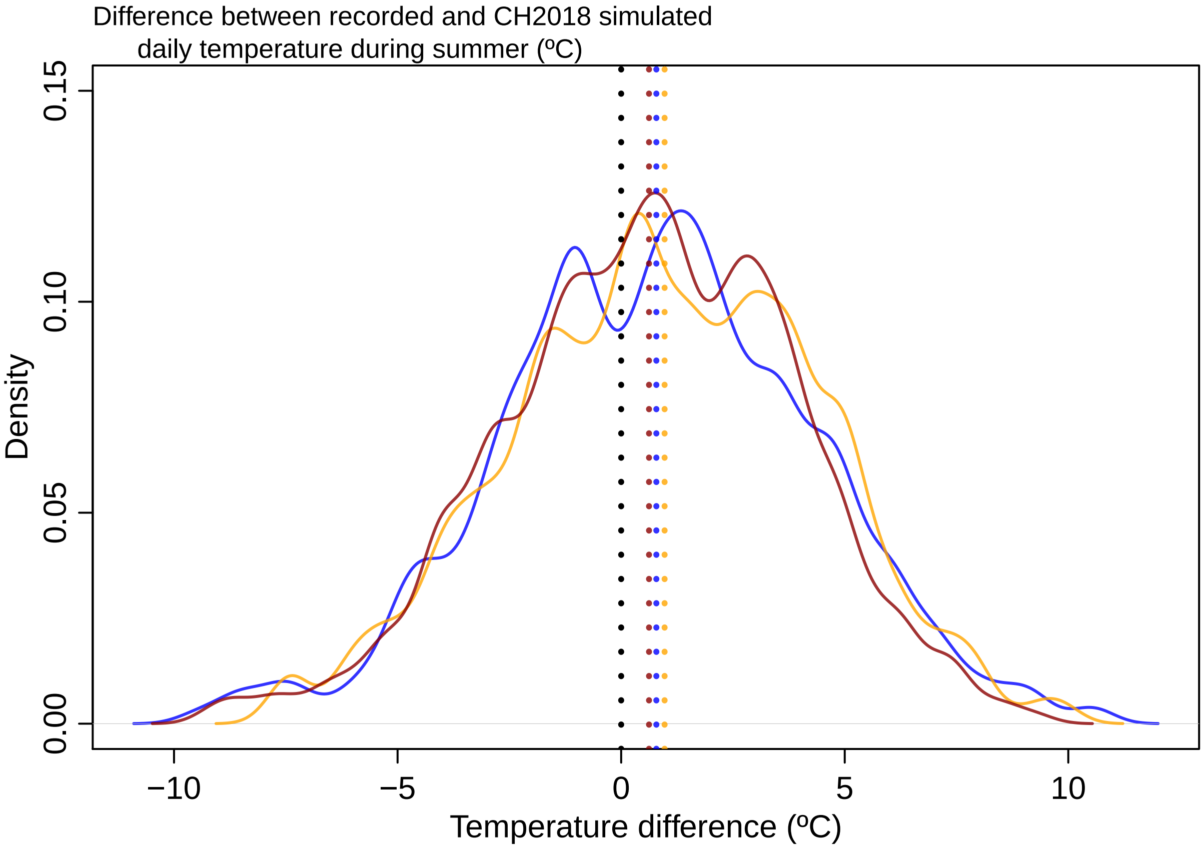

Figure S2.18 Density plot showing the distribution of differences between recorded daily mean temperatures in Chur meteorological station during the summer months (June to August) from 2017 to 2020 and the temperatures in the CH2018 simulations for the same station under climate change scenarios RCP2.6 (blue line), RCP4.5 (orange line), and RCP 8.5 (red line). On average, simulated temperatures were 0.79 K colder than recorded temperatures, as can be seen by comparing the black vertical dotted line (which indicates the expected mean difference value of 0) and the vertical colored lines (which indicate the mean temperature difference in each climate change scenario).

We therefore applied a correction to the CH2018 simulated data, to make them comparable to the data we used to train the demographic models.

```
## Calculate difference between measured and simulated temperatures
dT26 = mean(wsdata$temp[wsdata$date %in% summers_rcp26$date] -
                summers_rcp26$temp[summers_rcp26$date %in% wsdata$date])
dT45 = mean(wsdata$temp[wsdata$date %in% summers_rcp45$date] -
                summers_rcp45$temp[summers_rcp45$date %in% wsdata$date])
dT85 = mean(wsdata$temp[wsdata$date %in% summers_rcp85$date] -
                summers_rcp85$temp[summers_rcp85$date %in% wsdata$date])
temp_correction = mean(c(dT26, dT45, dT85))

rcp26$temp_corrected = rcp26$temp + temp_correction
rcp45$temp_corrected = rcp45$temp + temp_correction
rcp85$temp_corrected = rcp85$temp + temp_correction
```

We also corrected relative humidity.

```
dRH26 = mean(wsdata$rh[wsdata$date %in% summers_rcp26$date] -
                 summers_rcp26$rh[summers_rcp26$date %in% wsdata$date])
dRH45 = mean(wsdata$rh[wsdata$date %in% summers_rcp45$date] -
```

```
                 summers_rcp45$rh[summers_rcp45$date %in% wsdata$date])
dRH85 = mean(wsdata$rh[wsdata$date %in% summers_rcp85$date] -
                 summers_rcp85$rh[summers_rcp85$date %in% wsdata$date])

rh_correction = mean(c(dRH26, dRH45, dRH85))

rcp26$rh_corrected = rcp26$rh + rh_correction
rcp45$rh_corrected = rcp45$rh + rh_correction
rcp85$rh_corrected = rcp85$rh + rh_correction
```

Next, we used the CH2018 simulated daily time series to calculate the rain over the last 5 days and then we used models trained in section 1.1.3 to predict future weather conditions at the 2000 m site.

```
### Adding columns with previous rain info
rcp26$l5drain <- numeric(nrow(rcp26))
rcp26$l5dtemp <- numeric(nrow(rcp26))
for(i in 5:nrow(rcp26)){
        rcp26$l5drain[i] <- mean(rcp26$rain[(i-4):i])
        rcp26$l5dtemp[i] <- mean(rcp26$temp_corrected[(i-4):i])
}

rcp45$l5drain <- numeric(nrow(rcp45))
rcp45$l5dtemp <- numeric(nrow(rcp45))
for(i in 5:nrow(rcp45)){
        rcp45$l5drain[i] <- mean(rcp45$rain[(i-4):i])
        rcp45$l5dtemp[i] <- mean(rcp45$temp_corrected[(i-4):i])
}

rcp85$l5drain <- numeric(nrow(rcp85))
rcp85$l5dtemp <- numeric(nrow(rcp85))
for(i in 5:nrow(rcp85)){
        rcp85$l5drain[i] <- mean(rcp85$rain[(i-4):i])
        rcp85$l5dtemp[i] <- mean(rcp85$temp_corrected[(i-4):i])
}


## Estimate SM in Calanda
mod_cal_sm <- readRDS("model-fits/abiotic/mod_cal_soilmoist.rds")

pred_data <- list(
        temp = sqrt(abs(rcp26$l5dtemp)),
        rain =  sqrt(rcp26$l5drain),
        rh = sqrt(rcp26$rh))

predSM <- link(mod_cal_sm, data = pred_data)
rcp26$cal_predmoist <- apply(predSM, 2, mean)

pred_data <- list(
        temp = sqrt(abs(rcp45$l5dtemp)),
        rain =  sqrt(rcp45$l5drain),
        rh = sqrt(rcp45$rh))

predSM <- link(mod_cal_sm, data = pred_data)
rcp45$cal_predmoist <- apply(predSM, 2, mean)
```

```r
pred_data <- list(
        temp = sqrt(abs(rcp85$l5dtemp)),
        rain =  sqrt(rcp85$l5drain),
        rh = sqrt(rcp85$rh))

predSM <- link(mod_cal_sm, data = pred_data)
rcp85$cal_predmoist <- apply(predSM, 2, mean)


## Estimate temperature in Calanda
mod_cal_t <- readRDS("model-fits/abiotic/mod_cal_temp.rds")
calandaclim <- read_csv("processed-data/calandaclim.csv")

muRH = mean(calandaclim$rh[!is.na(calandaclim$cal_temp)])
sdRH = sd(calandaclim$rh[!is.na(calandaclim$cal_temp)])

pred_data <- list(temp = rcp26$temp_corrected,
                  rh = (rcp26$rh_corrected - muRH)/sdRH)

predT <- link(mod_cal_t, data = pred_data)
rcp26$cal_predtemp <- apply(predT, 2, mean)

pred_data <- list(temp = rcp45$temp_corrected,
                  rh = (rcp45$rh_corrected - muRH)/sdRH)

predT <- link(mod_cal_t, data = pred_data)
rcp45$cal_predtemp <- apply(predT, 2, mean)

pred_data <- list(temp = rcp85$temp_corrected,
                  rh = (rcp85$rh_corrected - muRH)/sdRH)


predT <- link(mod_cal_t, data = pred_data)
rcp85$cal_predtemp <- apply(predT, 2, mean)


summers_rcp26 <- rcp26[month(rcp26$date) %in% c(6,7,8), ]
summers_rcp45 <- rcp45[month(rcp45$date) %in% c(6,7,8), ]
summers_rcp85 <- rcp85[month(rcp85$date) %in% c(6,7,8), ]


#### The simulated soil moisture seems 0.02 higher than the 'measured' soil moisture

dSM26 = mean(wsdata$cal_predmoist[month(wsdata$date) %in% c(6,7,8)] -
                     summers_rcp26$cal_predmoist[year(summers_rcp26$date) %in%
                                                        2016:2020], na.rm = T)
dSM45 = mean(wsdata$cal_predmoist[month(wsdata$date) %in% c(6,7,8)] -
                     summers_rcp45$cal_predmoist[year(summers_rcp45$date) %in%
                                                        2016:2020], na.rm = T)
dSM85 = mean(wsdata$cal_predmoist[month(wsdata$date) %in% c(6,7,8)] -
                     summers_rcp85$cal_predmoist[year(summers_rcp85$date) %in%
                                                        2016:2020], na.rm = T)
```

```
sm_correction = mean(c(dSM26, dSM45, dSM85))

summers_rcp26$cal_predmoist = summers_rcp26$cal_predmoist + sm_correction
summers_rcp45$cal_predmoist = summers_rcp45$cal_predmoist + sm_correction
summers_rcp85$cal_predmoist = summers_rcp85$cal_predmoist + sm_correction

## Create time series of model variables
# RCP 2.6
rcp26TS = data.frame(year = 2017:2098,
                     moistLS = numeric(82),
                     tempLS = numeric(82),
                     tempL2S = numeric(82))

for (i in 1:82) {

        rcp26TS$moistLS[i] = mean(summers_rcp26[year(summers_rcp26$date) ==
                                                     (rcp26TS$year[i] -
                                                             1),]$cal_predmoist)
        rcp26TS$tempLS[i] = mean(summers_rcp26[year(summers_rcp26$date) ==
                                                    (rcp26TS$year[i]
                                                     - 1),]$cal_predtemp)
        rcp26TS$tempL2S[i] = mean(c(mean(summers_rcp26[year(summers_rcp26$date) ==
                                                             (rcp26TS$year[i]
                                                              - 1),]$cal_predtemp),
                                    mean(summers_rcp26[year(summers_rcp26$date) ==
                                                             (rcp26TS$year[i]
                                                              - 2),]$cal_predtemp)))
}

# RCP 4.5
rcp45TS = data.frame(year = 2017:2098,
                     moistLS = numeric(82),
                     tempLS = numeric(82),
                     tempL2S = numeric(82))

for (i in 1:82) {

        rcp45TS$moistLS[i] = mean(summers_rcp45[year(summers_rcp45$date) ==
                                                     (rcp45TS$year[i] -
                                                             1),]$cal_predmoist)
        rcp45TS$tempLS[i] = mean(summers_rcp45[year(summers_rcp45$date) ==
                                                    (rcp45TS$year[i] -
                                                            1),]$cal_predtemp)
        rcp45TS$tempL2S[i] = mean(c(mean(summers_rcp45[year(summers_rcp45$date) ==
                                                             (rcp45TS$year[i]
                                                              - 1),]$cal_predtemp),
                                    mean(summers_rcp45[year(summers_rcp45$date) ==
                                                             (rcp45TS$year[i]
                                                              - 2),]$cal_predtemp)))
}

# RCP 8.5
rcp85TS = data.frame(year = 2017:2098,
```

```
                     moistLS = numeric(82),
                     tempLS = numeric(82),
                     tempL2S = numeric(82))

for (i in 1:82) {

        rcp85TS$moistLS[i] = mean(summers_rcp85[year(summers_rcp85$date) ==
                                                   (rcp85TS$year[i] -
                                                           1),]$cal_predmoist)
        rcp85TS$tempLS[i] = mean(summers_rcp85[year(summers_rcp85$date) ==
                                                  (rcp85TS$year[i] -
                                                          1),]$cal_predtemp)
        rcp85TS$tempL2S[i] = mean(c(mean(summers_rcp85[year(summers_rcp85$date) ==
                                                          (rcp85TS$year[i]
                                                           - 1),]$cal_predtemp),
                                   mean(summers_rcp85[year(summers_rcp85$date) ==
                                                          (rcp85TS$year[i]
                                                           - 2),]$cal_predtemp)))
}


write_csv(rcp26TS, "processed-data/rcp26TS.csv")
write_csv(rcp45TS, "processed-data/rcp45TS.csv")
write_csv(rcp85TS, "processed-data/rcp85TS.csv")
```

Creating step-wise climate change time series

To simulate dynamics under stepwise climate change similar in magnitude to the one expected by the end of the century under different climate change scenarios, we created timeseries of temperatures and soil moisture by sampling from the last decade of each RCP scenario.

```
# Make temperature and soil moisture time series for stepwise climate change
for (rcp in c("26", "45","85")) {

        path_climTS = paste("processed-data/rcp", rcp, "TS.csv", sep = "")
        climTS = read.csv(path_climTS)

        # Sample conditions from last decade of RCP simulations
        set.seed(505)
        csq <- sample(73:82, size = 82, replace = T)

        swcc_rcp <- data.frame(year = climTS$year,
                               tempLS = rnorm(82,
                                              climTS$tempLS[csq],
                                              sd(climTS$tempLS[73:82])),
                               moistLS = rnorm(82,
                                               climTS$moistLS[csq],
                                               sd(climTS$moistLS[73:82]))
        )


        swcc_rcp$tempL2S <- swcc_rcp$tempLS
```

```
        for (i in 2:nrow(swcc_rcp)){
                swcc_rcp$tempL2S[i] <- mean(c(swcc_rcp$tempLS[i],
                                                swcc_rcp$tempLS[i-1]))
        }

        swcc_rcp$tempL2S[1] <- mean(c(swcc_rcp$tempLS[1],
                                      rnorm(1, mean(climTS$tempLS[1:10]),
                                            sd(climTS$tempLS[1:10]))))


        path_SWTS = paste("processed-data/swcc_rcp", rcp, ".csv", sep = "")
        write_csv(swcc_rcp, path_SWTS)

}
```

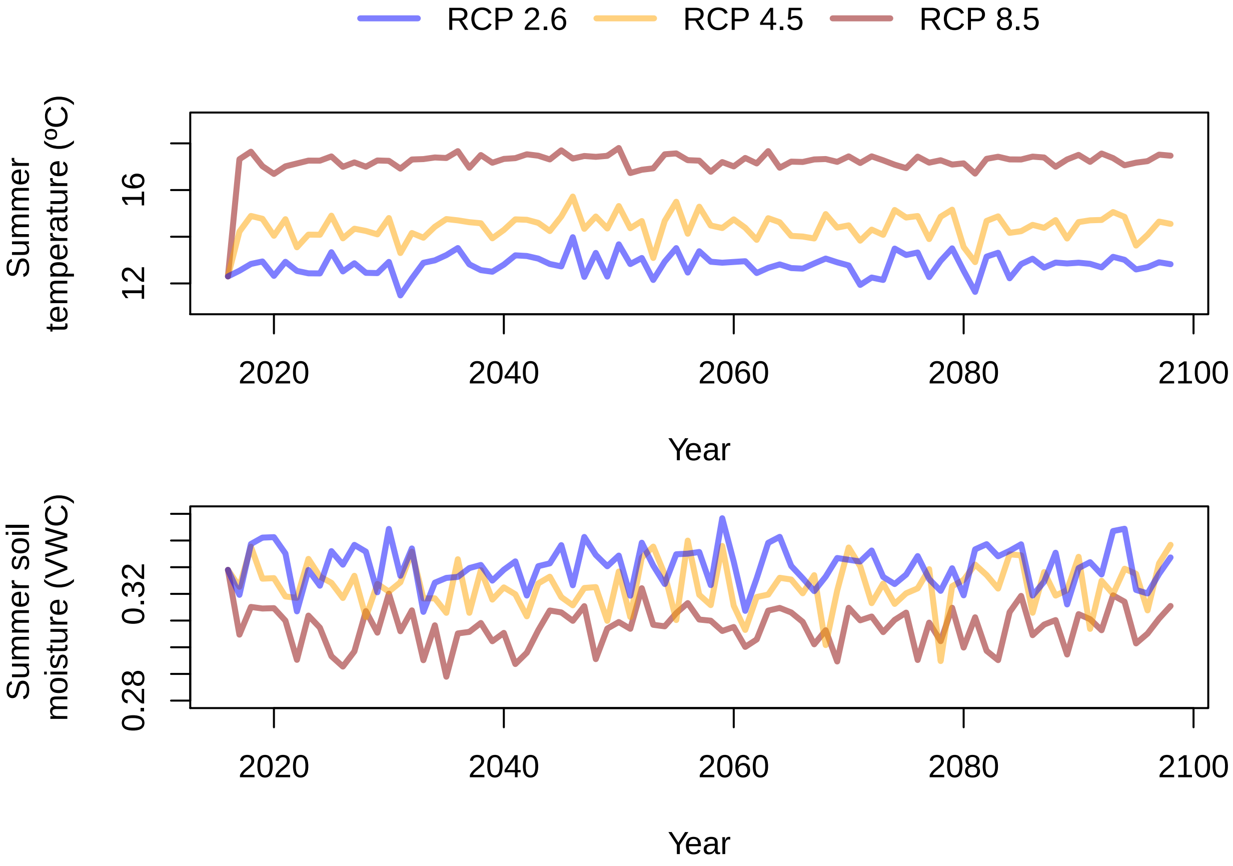

Figure S2.20 Time series of stepwise climate change corresponding to different RCP scenarios which we used for simulations.

3.3.2 Simulations

Pre-climate change simulations

We first simulated dynamics under a constant climate (mean temperature and soil moisture conditions during the summers of 2017 to 2020 simulated in CH2018 RCP 2.6, 4.5, and 8.5) until the community reached a quasi-equilibrium.

This function simulates yearly dynamics under constant climate.

```r
# This function uses fitted demographic models to project ramet demography
# for one species, one time step
# under constant average conditions between 2017 and 2020 in RCP 2.6, 4.5, and 8.5
# or under historical conditions


EqClimDemoSim <- function(spname, simdat, ps = 1, meanpars = T,
                          fixedclim = T) {

    source("scripts/spp_modfits.R")
    spcode <- sppList[which(domsp == spname)]

    # 1: Prepare data
    d <- data.frame(ramet = subset(simdat, species == spname)$unique_id)

    if(nrow(d)==0) {
        return(simdat[0,])
    } else{
        d$ramet <- as.character(d$ramet)

        ## b: add predictor variables
        d$u1 <- NA
        d$x <- NA
        d$y <- NA

        for(i in 1:nrow(d)) {

                y1 <- simdat[simdat$unique_id == d$ramet[i],]

                d$u1[i] <- y1$cover_cm2
                d$x[i] <- y1$x
                d$y[i] <- y1$y
                rm(y1)
        }

        d$year <- max(simdat$year)

        ## Quantify crowding

        # An interaction radius of ca. 15 cm seems reasonable for starters
        delta <- 0.02

        # Intraspecific crowding (w)
        maxnn <- 64

        rnd <- matrix(0,nrow = nrow(d), ncol = maxnn-1)
        rnc <- matrix(0,nrow = nrow(d), ncol = maxnn-1)

        rm(maxnn)

        for (i in 1:nrow(d)) {
                i_x <- d$x[i]
                i_y <- d$y[i]
```

```
        x <- subset(simdat, species == spname)
        x <- x[(sqrt((x$x - i_x)^2 + (x$y - i_y)^2) <=20),]
        ri <- x[x$unique_id == d$ramet[i],]
        ni <- x[x$unique_id != d$ramet[i],]

        if (nrow(ni) > 0) {
            rnd[i,1:nrow(ni)] <- sqrt((ri$x - ni$x)^2 + (ri$y - ni$y)^2)
            rnc[i,1:nrow(ni)] <- ni$cover_cm2
        }

        rm(x,ni,ri, i_x, i_y)
}

rnd[is.na(rnd)] <- 0
rnd2 <- rnd^2

d$w <- numeric(nrow(d))

for (i in 1:nrow(d)){
        d$w[i] <- sum(exp(-delta*rnd2[i,])*rnc[i,])
}

rm(rnd, rnd2, rnc)

# Interspecific crowding
rnd <- matrix(0,nrow = nrow(d), ncol = 3385)
rnc <- matrix(0,nrow = nrow(d), ncol = 3385)

for (i in 1:nrow(d)) {
        i_x <- d$x[i]
        i_y <- d$y[i]

        ri <- subset(simdat, unique_id == d$ramet[i])
        x <- subset(simdat, species != spname)
        x <- x[(sqrt((x$x - i_x)^2 + (x$y - i_y)^2) <=20),]

        ni <- x[x$unique_id != d$ramet[i],]

        if (nrow(ni) > 0) {
            rnd[i,1:nrow(ni)] <- sqrt((ri$x - ni$x)^2 + (ri$y - ni$y)^2)
            rnc[i,1:nrow(ni)] <- ni$cover_cm2
        }

        rm(x,ni,ri, i_x, i_y)
}

rnd[is.na(rnd)] <- 0
rnd2 <- rnd^2

d$w.cinter <- numeric(nrow(d))

for (i in 1:nrow(d)){
        d$w.cinter[i] <- sum(exp(-delta*rnd2[i,])*rnc[i,])
```

```r
}

rm(rnd, rnd2, rnc)

# Adjust crowding experienced by marginal ramets
## First, ramets next to two margins (i.e., at the corner)
corner <- with(d, which((x < 15 | x > 405) & (y < 15 | y > 165)))

d$w[corner] <- d$w[corner] / 0.3926553
d$w.cinter[corner] <- d$w.cinter[corner] / 0.4893565

## Then, ramets near one edge and next to other
nextnear <- with(d, which(((x < 15 | x > 405) & (y < 20 | y > 160)) |
                                  ((x < 20 | x > 400) &
                                       (y < 15 | y > 165))))
nextnear <- nextnear[!(nextnear %in% corner)]

d$w[nextnear] <- d$w[nextnear] / 0.5940011
d$w.cinter[nextnear] <- d$w.cinter[nextnear] / 0.6586441

## Next, ramets near two edges
nearnear <- with(d, which(((x < 20 | x > 400) & (y < 20 | y > 160))))
nearnear <- nearnear[!((nearnear %in% corner) | (nearnear %in% nextnear))]

d$w[nearnear] <- d$w[nearnear] / 0.8650126
d$w.cinter[nearnear] <- d$w.cinter[nearnear] / 0.8865053


## Then, ramets next to only one margin
next_to_one <- with(d, which(x < 15 | x > 405 | y < 15 | y > 165))
next_to_one <- next_to_one[!((next_to_one %in% corner) |
                                 (next_to_one %in% nextnear))]
next_to_one <- next_to_one[!(next_to_one %in% nearnear)]

d$w[next_to_one] <- d$w[next_to_one] / 0.6426553
d$w.cinter[next_to_one] <- d$w.cinter[next_to_one] / 0.6995516


## Finally, ramets near to only one margin
near_to_one <- with(d, which(x < 20 | x > 400 | y < 20 | y > 160))
near_to_one <- near_to_one[!(near_to_one %in% next_to_one)]
near_to_one <- near_to_one[!((near_to_one %in% corner) |
                                 (near_to_one %in% nextnear))]
near_to_one <- near_to_one[!(near_to_one %in% nearnear)]

d$w[near_to_one] <- d$w[near_to_one] / 0.9304874
d$w.cinter[near_to_one] <- d$w.cinter[near_to_one] / 0.9415551

rm(near_to_one, next_to_one, nearnear, nextnear, corner)


####
## Climate
```

```
#path_climTS = paste("rcp", rcp, "TS.csv", sep = "")
spp_clim_scales = read.csv('processed-data/spp_clim_scales.csv')

if(fixedclim == T) {
        rcp26 = read.csv("processed-data/rcp26TS.csv")
        rcp45 = read.csv("processed-data/rcp45TS.csv")
        rcp85 = read.csv("processed-data/rcp85TS.csv")

        ## Mean soil moisture during the previous summer
        d$moistLS <- mean(c(rcp26$moistLS[1:4],
                            rcp45$moistLS[1:4],
                            rcp85$moistLS[1:4]))

        ## Mean temp previous summer
        d$tempLS <- mean(c(rcp26$tempLS[1:4],
                           rcp45$tempLS[1:4],
                           rcp85$tempLS[1:4]))

        ## Mean temp previous summer
        d$tempL2S <- mean(c(rcp26$tempL2S[1:4],
                            rcp45$tempL2S[1:4],
                            rcp85$tempL2S[1:4]))

} else {
        churTS = read.csv("processed-data/chur_climhist.csv")
        climyear <- sample(1:nrow(churTS), 1)

        ## Mean soil moisture during the previous summer
        d$moistLS <- churTS$moistLS[climyear]

        ## Mean temp previous summer
        d$tempLS <- churTS$tempLS[climyear]

        ## Mean temp previous summer
        d$tempL2S <- churTS$tempL2S[climyear]

}

################ #
### Survival ###
################ #
p_path <- paste("processed-data/params_", spcode, ".rds", sep = "")
params <- readRDS(p_path)

spn = which(spname == domsp)

predat_s <- list(u = (d$u1 - spp_clim_scales$muU.s[spn])/
                    spp_clim_scales$sdU.s[spn],
                 tempLS = (d$tempLS - spp_clim_scales$muT.s[spn])/
                    spp_clim_scales$sdT.s[spn] ,
                 moistLS = (d$moistLS - spp_clim_scales$muM.s[spn])/
                    spp_clim_scales$sdM.s[spn],
                 w = d$w,
```

```
                wC = d$w.cinter)

# Calculate logodds and probability of survival
## Use the mean of sampled parameters or single parameters
if (meanpars == T) {
    logodds <- mean(params$s.tef) / (1 + exp(-mean(params$s.k) *
                                                 (predat_s$tempLS -
                                                   (mean(params$s.ttp_a) +
                                                    mean(params$s.ttp_b) *
                                                        predat_s$moistLS)))) +
            mean(params$s.b) * predat_s$u +
            (mean(params$s.a_0) + mean(params$s.b_a) *
                 predat_s$tempLS)*predat_s$w +
            (mean(params$s.aC_0)  + mean(params$s.b_aC) *
                 predat_s$tempLS)*predat_s$wC - 4
} else {
        logodds <-  params$s.tef[ps] / (1 + exp(-params$s.k[ps] *
                                                   (predat_s$tempLS -
                                                    (params$s.ttp_a[ps] +
                                                     params$s.ttp_b[ps] *
                                                         predat_s$moistLS)))) +
                params$s.b[ps] * predat_s$u +
                (params$s.a_0[ps] + params$s.b_a[ps] * predat_s$tempLS )*
            predat_s$w +
                (params$s.aC_0[ps]  + params$s.b_aC[ps] * predat_s$tempLS )*
            predat_s$wC - 4
}

## Survival probability
p.survival <- exp(logodds) / (exp(logodds) + 1)

# Simulate survival
d$survival <- rbinom(length(p.survival), 1, p.survival )

########## #
# Growth #
########## #
# Filter survivors
dG <- d[d$survival == 1,]

### Predict growth
gmu <- numeric(nrow(dG))

# Scale temperature with mean and sd of data used for model fitting
predT <- ((dG$tempL2S-spp_clim_scales$muT.g[spn])/spp_clim_scales$sdT.g[spn])


# Calculate mean growth rate for each surviving ramet
## Use the mean of sampled parameters or single parameters
if (meanpars == T) {

    for (i in 1:nrow(dG)) {
        gmu[i] <- mean(params$g.b0) +
```

```
                mean(params$g.tef)*exp(-((predT[i] - mean(params$g.ttp))^2/
                                             2*mean(params$g.tsd)^2)) +
                log(dG$u1[i]) * mean(params$g.b) -
                dG$w.cinter[i] * exp(mean(params$g.aC_0) + mean(params$g.b_aC)*
                                         predT[i]) -
                dG$w[i] * exp(mean(params$g.a_0) + mean(params$g.b_a)*predT[i])
    }

} else {

        for (i in 1:nrow(dG)) {
                gmu[i] <- params$g.b0[ps] +
                        params$g.tef[ps]*exp(-((predT[i] - params$g.ttp[ps])^2/
                                                    2*params$g.tsd[ps]^2)) +
                        log(dG$u1[i]) * params$g.b[ps] -
                        dG$w.cinter[i] * exp(
                            params$g.aC_0[ps] + params$g.b_aC[ps]*predT[i]) -
                        dG$w[i] * exp(params$g.a_0[ps] + params$g.b_a[ps]*
                                          predT[i])
        }
}


# Simulate growth rates
## With either the mean of sigma samples, or a single sample
if (meanpars == T) gsim <- exp(rnorm(nrow(dG), gmu, mean(params$g.sigma)/1))
if (meanpars == F) gsim <- exp(rnorm(nrow(dG), gmu, params$g.sigma[ps]/1))


# Calculate cover at time t + 1
dG$u2 <- dG$u1 * gsim

# Set minimum and maximum ramet sizes to cap growth
dG$u2 <- ifelse(dG$u2 > 300, 300, dG$u2)
dG$u2 <- ifelse(dG$u2 < 0.1, 0.1, dG$u2)

# Create data frame with ramets in year t + 1

### Keep only survivors
simdat_next_survivors <- simdat[simdat$unique_id %in% dG$ramet,]

### Make them grow
simdat_next_survivors$cover_cm2 <- dG$u2

### Update year
simdat_next_survivors$year <- simdat_next_survivors$year + 1


################# #
## Recruitment ##
################# #

## Prepare predictive data
```

```r
qcords <- read.csv('processed-data/qcords_megaplot.csv')
# Make list of all quadrants
allquads_sp <- as.character(qcords$quadrant)

closedqs <- as.character(subset(simdat, species == spname)$quadrant_id)
openqs <- allquads_sp[!(allquads_sp %in% closedqs)]

if(length(openqs) > 0) {
        r <- data.frame(quadrant = openqs)
        r$quadrant <- as.character(r$quadrant)

        ## b: add predictor variables
        r$x <- qcords$x[qcords$quadrant %in% r$quadrant]
        r$y <- qcords$y[qcords$quadrant %in% r$quadrant]

        ## Quantify crowding (w)
        rnd <- matrix(0,nrow = nrow(r), ncol = 399)
        rnc <- matrix(0,nrow = nrow(r), ncol = 399)

        for (i in 1:nrow(r)) {
                i_x <- r$x[i]
                i_y <- r$y[i]
                x <- subset(simdat, species == spname)
                # Keep only neighbors within 20 cm from focal ramet
                x <- x[(sqrt((x$x - i_x)^2 + (x$y - i_y)^2) <=20),]

                if(nrow(x) > 0){
                    rnd[i,1:nrow(x)] <- sqrt((r$x[i] - x$x)^2 + (r$y[i] - x$y)^2)
                    rnc[i,1:nrow(x)] <- x$cover_cm2
                }

                rm(x, i_x, i_y)
        }

        rnd[is.na(rnd)] <- 0
        rnd2 <- rnd^2

        # Quantify intraspecific crowding (w)
        r$w <- numeric(nrow(r))

        for (i in 1:nrow(r)){
                r$w[i] <- sum(exp(-delta*rnd2[i,])*rnc[i,])
        }

        rm(rnc, rnd, rnd2)

        ################ #
        ## Quantify interspecific crowding (w.cinter)
        rnd <- matrix(0,nrow = nrow(r), ncol = 3385)
        rnc <- matrix(0,nrow = nrow(r), ncol = 3385)

        for (i in 1:nrow(r)) {
                i_x <- r$x[i]
```

```
        i_y <- r$y[i]
        x <- subset(simdat, species != spname)
        # Keep only neighbors within 20 cm from focal ramet
        x <- x[(sqrt((x$x - i_x)^2 + (x$y - i_y)^2) <=20),]

        if(nrow(x) > 0){
            rnd[i,1:nrow(x)] <- sqrt((r$x[i] - x$x)^2 + (r$y[i] - x$y)^2)
            rnc[i,1:nrow(x)] <- x$cover_cm2
        }

        rm(x, i_x, i_y)
}

rnd[is.na(rnd)] <- 0
rnd2 <- rnd^2


# Quantify interspecific crowding (w.cinter)
r$w.cinter <- numeric(nrow(r))

for (i in 1:nrow(r)){
        r$w.cinter[i] <- sum(exp(-delta*rnd2[i,])*rnc[i,])
}

rm(rnc, rnd, rnd2)


# Increase w.cinter to account for species not included in simulations
#r$w.cinter <- r$w.cinter/0.45


# Adjust crowding experienced by marginal ramets
## First, ramets next to two margins (i.e., at the corner)
corner <- with(r, which((x < 15 | x > 405) & (y < 15 | y > 165)))

r$w[corner] <- r$w[corner] / 0.3926553
r$w.cinter[corner] <- r$w.cinter[corner] / 0.4893565

## Then, ramets near one edge and next to other
nextnear <- with(r, which(((x < 15 | x > 405) & (y < 20 | y > 160)) |
                              ((x < 20 | x > 400) & (y < 15 | y > 165))))
nextnear <- nextnear[!(nextnear %in% corner)]

r$w[nextnear] <- r$w[nextnear] / 0.5940011
r$w.cinter[nextnear] <- r$w.cinter[nextnear] / 0.6586441

## Next, ramets near two edges
nearnear <- with(r, which(((x < 20 | x > 400) & (y < 20 | y > 160))))
nearnear <- nearnear[!((nearnear %in% corner) | (nearnear %in% nextnear))]

r$w[nearnear] <- r$w[nearnear] / 0.8650126
r$w.cinter[nearnear] <- r$w.cinter[nearnear] / 0.8865053
```

```r
## Then, ramets next to only one margin
next_to_one <- with(r, which(x < 15 | x > 405 | y < 15 | y > 165))
next_to_one <- next_to_one[!((next_to_one %in% corner) |
                                 (next_to_one %in% nextnear))]
next_to_one <- next_to_one[!(next_to_one %in% nearnear)]

r$w[next_to_one] <- r$w[next_to_one] / 0.6426553
r$w.cinter[next_to_one] <- r$w.cinter[next_to_one] / 0.6995516


## Finally, ramets near to only one margin
near_to_one <- with(r, which(x < 20 | x > 400 | y < 20 | y > 160))
near_to_one <- near_to_one[!(near_to_one %in% next_to_one)]
near_to_one <- near_to_one[!((near_to_one %in% corner) |
                                 (near_to_one %in% nextnear))]
near_to_one <- near_to_one[!(near_to_one %in% nearnear)]

r$w[near_to_one] <- r$w[near_to_one] / 0.9304874
r$w.cinter[near_to_one] <- r$w.cinter[near_to_one] / 0.9415551

rm(near_to_one, next_to_one, nearnear, nextnear, corner)



# Recruitment climate
r$tempLS <- d$tempLS[1]


### PREDICT WITH MODEL
# Scale temperature with mean and sd of data used to train the model
predT <- ((r$tempLS) - spp_clim_scales$muT.r[spn])/
    spp_clim_scales$sdT.r[spn]

# Data for model predictions
predat_r <- list(temp = predT,
                 w = r$w,
                 wC = r$w.cinter)

# Calculate logodds and probability of recruitment

if (meanpars == T) {
    logodds.r <- exp(mean(params$r.a_0) +
                        mean(params$r.b_a)*predat_r$temp) * predat_r$w -
            exp(mean(params$r.aC_0) +
                mean(params$r.b_aC)*predat_r$temp) * predat_r$wC - 10
} else {
        logodds.r <- exp(params$r.a_0[ps] +
                            params$r.b_a[ps] * predat_r$temp )*predat_r$w -
                exp(params$r.aC_0[ps]  +
                    params$r.b_aC[ps]  * predat_r$temp )*predat_r$wC - 10
}

# Probability of recruitment in each quadrant
```

```
            p.recruitment <- exp(logodds.r) / (exp(logodds.r) + 1)

            # Simulate recruitment
            r$recruitment <- rbinom(length(p.recruitment), 1, p.recruitment )

            ## Recruit size model
            recs <- subset(r, recruitment == 1)

            recSizeDist = readRDS('processed-data/recSizeDist.rds')
            posizes = c(1.5625, 3.125, 6.25, 12.5, 18.75, 25)

            rec.sizes <- sample(posizes, nrow(recs),
                                replace = T, prob = recSizeDist[spn,])

            ### Prepare data frame to add recruits to next time step
            nrec <- sum(r$recruitment)
            newramets <- data.frame(species = rep(spname, nrec),
                                    x = recs$x,
                                    y = recs$y,
                                    year = rep(simdat_next_survivors$year[1],nrec),
                                    cover_cm2 = rec.sizes,
                                    quadrant_id = recs$quadrant,
                                    unique_id = character(nrec),
                                    stringsAsFactors = F)

            rm(nrec)

            simdat_next <- rbind(simdat_next_survivors, newramets)
            rm(simdat_next_survivors, newramets, recs, d, r)

        } else {
            simdat_next <- simdat_next_survivors
            rm(simdat_next_survivors, d)
        }



        return(simdat_next)
    }

}
```

This function simulates community dynamics until a quasi-equilibrium is reached with the current climate.

```
# This function projects dynamics of original community until it reaches quasiequilibrium with the curr

AlpEqSim <- function(simdat, meanpars = T, ps = 1) {

        simdat_t1 <- simdat
        allsims <- simdat_t1

        equi = F

        while (equi == F){
```

```r
                simdat_t2 <- simdat_t1[0,]

                for (s in spnames) {
                        sp_t2 <- EqClimDemoSim(s, simdat = simdat_t1, meanpars = meanpars,
                                               ps = ps)
                        simdat_t2 <- rbind(simdat_t2, sp_t2)
                        rm(sp_t2)
                }

                simdat_t2$year[is.na(simdat_t2$year)] <- max(simdat_t2$year, na.rm = T)

                simdat_t2$species <- as.character(simdat_t2$species)
                simdat_t2$quadrant_id <- as.character(simdat_t2$quadrant_id)
                simdat_t2$unique_id <- as.character(simdat_t2$unique_id)

                for(i in 1:nrow(simdat_t2)) {
                        simdat_t2$unique_id[i] <- paste("t2_", i, sep="")
                }

                allsims <- rbind(allsims, simdat_t2)

                # Check if equilibrium has been reached
                simdat_t1$species <- factor(simdat_t1$species, levels = spnames)
                simdat_t2$species <- factor(simdat_t2$species, levels = spnames)
                cov1 <- with(simdat_t1, tapply(cover_cm2, species, sum))
                cov1[is.na(cov1)] <- 0

                cov2 <- with(simdat_t2, tapply(cover_cm2, species, sum))
                cov2[is.na(cov2)] <- 0

                gr <- (cov2-cov1)/cov1

                equi <- (sum(abs(gr)<0.05 | abs(cov1 - cov2) < 320, na.rm = T) +
                                sum(is.infinite(gr))) == length(spnames)

                simdat_t1 <- simdat_t2

                print(max(simdat_t1$year)-2017)
        }

        simdat_t1$year <- 2017

        output <- list(allsims, simdat_t1)
        return(output)

}
```

Starting with the megaturf ensemble, we let the community quasi-equilibrate and save it to be the starting point of our simulations.

```r
## Load simulation starting point
## (ensemble community composed of 2017 maps of turfs transplanted to 2000-m)
simdat_t1 <- read.csv("processed-data/megaturf.csv")
# keep taxa with good model predictions
```

```r
simdat_t1 <- subset(simdat_t1, species %in% spnames)
# keep relevant variables
simdat_t1 <- select(simdat_t1, species, x, y, year, cover_cm2, quadrant_id)
simdat_t1$unique_id <- paste("t1_", 1:nrow(simdat_t1), sep = "")
simdat_t1$species <- as.character(simdat_t1$species)


# Burn in period under mean climate from 2017-2020 across all scenarios
equilibrium <- AlpEqSim(simdat_t1)
save_path <- paste("simulations/megaturf/simdat_equi_meanpars.csv", sep = "")
write.csv(equilibrium[[2]], save_path)

save_path <- paste("simulations/megaturf/journey-to-equi_meanpars.csv", sep = "")
write.csv(equilibrium[[1]], save_path)
```

Climate change simulations

We used the quasi-equilibrium community from the previous step as the starting point of our climate change simulations. We simulated dynamics from 2017 until 2098 under the RCP 2.6, 4.5, and 8.5 scenarios.

This function projects dynamics under gradual or stepwise change, with or without a full influence of demographic and competitive lags.

```r
## This function projects community dynamics under climate change
## and returns a dataframe of ramets in the simulated community throughout time

AlpSim <- function(simdat, scenario = "45", until = 2098, quasieq = F,
                   meanpars = T, ps = 1, stepwise = F, smoothline = F,
                   current_clim = F) {

    simdat_t1 <- simdat
    allsims <- simdat_t1

    nyears = until - simdat_t1$year[1]

    if(quasieq) {

            lags = numeric(nyears)

            for (yr in 1:nyears){

                equi = F
                teq <- 1

                while (equi == F){

                    simdat_t2 <- simdat_t1[0,]

                    for (s in spnames) {
                        sp_t2 <- ClimDemoSim(s, simdat = simdat_t1,
                                             meanpars = meanpars, rcp = scenario,
                                             ps = ps, stepwise = stepwise,
                                             smoothline = smoothline,
                                             current_clim = current_clim)
```

```
                    simdat_t2 <- rbind(simdat_t2, sp_t2)
                    rm(sp_t2)
                }

                simdat_t2$year[is.na(simdat_t2$year)] <- max(simdat_t2$year,
                                                             na.rm = T)

                simdat_t2$year <- max(simdat_t2$year) - 1

                simdat_t2$species <- as.character(simdat_t2$species)
                simdat_t2$quadrant_id <- as.character(simdat_t2$quadrant_id)
                simdat_t2$unique_id <- as.character(simdat_t2$unique_id)

                for(i in 1:nrow(simdat_t2)) {
                        simdat_t2$unique_id[i] <- paste("t2_", i, sep="")
                }

                simdat_t1$species <- factor(simdat_t1$species, levels = spnames)
                simdat_t2$species <- factor(simdat_t2$species, levels = spnames)
                cov1 <- with(simdat_t1, tapply(cover_cm2, species, sum))
                cov1[is.na(cov1)] <- 0

                cov2 <- with(simdat_t2, tapply(cover_cm2, species, sum))
                cov2[is.na(cov2)] <- 0
                gr <- (cov2-cov1)/cov1

                equi <- (sum(abs(gr)<0.05 | abs(cov1 - cov2) < 320, na.rm = T) +
                             sum(is.infinite(gr))) == length(spnames)


                simdat_t1 <- simdat_t2
                lags[yr] <- lags[yr] + 1
            }

            simdat_t2$year <- 2017 + yr

            allsims <- rbind(allsims, simdat_t2)
            simdat_t1 <- simdat_t2
            print(yr)
    }

    output <- list(allsims, lags)
    return(output)
} else{

    for (i in 1:nyears){

        simdat_t2 <- simdat_t1[0,]

        for (s in spnames) {
            sp_t2 <- ClimDemoSim(s, simdat = simdat_t1, meanpars = meanpars,
                                 rcp = scenario, ps = ps, stepwise = stepwise,
                                 smoothline = smoothline,
```

```
                                    current_clim = current_clim)
                simdat_t2 <- rbind(simdat_t2, sp_t2)
                rm(sp_t2)
            }

            simdat_t2$year[is.na(simdat_t2$year)] <- max(simdat_t2$year, na.rm = T)

            simdat_t2$species <- as.character(simdat_t2$species)
            simdat_t2$quadrant_id <- as.character(simdat_t2$quadrant_id)
            simdat_t2$unique_id <- 1:nrow(simdat_t2)

            allsims <- rbind(allsims, simdat_t2)
            simdat_t1 <- simdat_t2
            print(max(simdat_t2$year, na.rm = T))
        }

        return(allsims)
    }


}
```

```
## Load equilibrium megaturf
simdat_t1 <- read.csv("simulations/megaturf/simdat_equi_meanpars.csv")[-1]
simdat_t1$species <- as.character(simdat_t1$species)


## Dynamics with lags under gradual change
### RCP 2.6
future26 <- AlpSim(simdat_t1, scenario = "26")
save_path <- paste("simulations/megaturf/sims_26_slowCC_MT_meanpars.csv", sep = "")
write.csv(future26, save_path)

### RCP 4.5
future45 <- AlpSim(simdat_t1, scenario = "45")
save_path <- paste("simulations/megaturf/sims_45_slowCC_MT_meanpars.csv", sep = "")
write.csv(future45, save_path)

### RCP 8.5
future85 <- AlpSim(simdat_t1, scenario = "85")
save_path <- paste("simulations/megaturf/sims_85_slowCC_MT_meanpars.csv", sep = "")
write.csv(future85, save_path)
```

Stepwise climate change simulations

Then, we used these stepwise climate change time series to simulate dynamics from 2017 to 2098 for all taxa.

```
## Dynamics with lags after stepwise change
### RCP 2.6
future26 <- AlpSim(simdat_t1, scenario = "26", stepwise = T)
save_path <- paste("simulations/megaturf/sims_26_stepCC_MT_meanpars.csv", sep = "")
write.csv(future26, save_path)

### RCP 4.5
```

```r
future45 <- AlpSim(simdat_t1, scenario = "45", stepwise = T)
save_path <- paste("simulations/megaturf/sims_45_stepCC_MT_meanpars.csv", sep = "")
write.csv(future45, save_path)

### RCP 8.5
future85 <- AlpSim(simdat_t1, scenario = "85", stepwise = T)
save_path <- paste("simulations/megaturf/sims_85_stepCC_MT_meanpars.csv", sep = "")
write.csv(future85, save_path)
```

No-lag simulations

To quantify the effect of demographic and competitive lags on the timescales and trajectories of community dynamics, we simulated dynamics under the RCP scenarios letting the community reach a quasi-equilibrium at the weather conditions of each year in the time series.

```r
## Dynamics without lags
### RCP 2.6
allsims <- AlpSim(simdat_t1, scenario = "26", quasieq = T)
save_path <- paste("simulations/megaturf/sims_26_slowCC_nolags_MT_meanpars.csv", sep = "")
write.csv(allsims[[1]], save_path)
save_path <- paste("simulations/megaturf/lags_26_slowCC_nolags_MT_meanpars.csv", sep = "")
write.csv(allsims[[2]], save_path)

### RCP 4.5
allsims <- AlpSim(simdat_t1, scenario = "45", quasieq = T)
save_path <- paste("simulations/megaturf/sims_45_slowCC_nolags_MT_meanpars.csv", sep = "")
write.csv(allsims[[1]], save_path)
save_path <- paste("simulations/megaturf/lags_45_slowCC_nolags_MT_meanpars.csv", sep = "")
write.csv(allsims[[2]], save_path)

### RCP 8.5
allsims <- AlpSim(simdat_t1, scenario = "85", quasieq = T)
save_path <- paste("simulations/megaturf/sims_85_slowCC_nolags_MT_meanpars.csv", sep = "")
write.csv(allsims[[1]], save_path)
save_path <- paste("simulations/megaturf/lags_85_slowCC_nolags_MT_meanpars.csv", sep = "")
write.csv(allsims[[2]], save_path)
```

3.4 Analysing trajectories of community responses to climate change

To better understand and visualize trajectories of community responses to climate change, we did a Principal Coordinates Analysis (PCoA) based on a matrix of Euclidean distances (we also used Bray-Curtis distances, without changes in the main results). The first step was to build matrices storing the total cover of taxa in each year of the simulations (i.e., 2017 to 2098).

We built matrices combining dynamics under different scenarios of gradual climate change.

```r
source('scripts/spp_simul.R')

rcps <- c('26', '45', '85')
vegmatrix <- matrix(0, ncol = length(spnames), nrow = 0)
for (scenario in rcps) {

        fpath <- paste("simulations/megaturf/sims_",
                       scenario,
```

```
                        "_slowCC_MT_meanpars.csv")

        future <- read_csv(fpath)[,-1]

        future$species <- factor(future$species, levels = spnames)

        vegmat <- matrix(0, ncol = length(spnames),
                            nrow = length(levels(factor(future$year))))

        for (yr in 1:length(levels(factor(future$year))) ){
                vegmat[yr,] <- with(
                        subset(future, year == levels(factor(future$year))[yr]),
                                      tapply(cover_cm2, species, sum))
        }

        vegmat[is.na(vegmat)] <- 0

        vegmatrix <- rbind(vegmatrix, vegmat)
}

colnames(vegmatrix) <- spnames
saveRDS(vegmatrix, "processed-data/ordidyn-rcp/vegmatrix_MT.rds")
```

And also, for each climate change scenario, we built matrices combining dynamics under gradual climate change (with and without lags), as well as under stepwise climate change.

```
source('scripts/spp_simul.R')

rcps <- c('26', '45', '85')

for (scenario in rcps) {

        pathfuture <- paste("simulations/megaturf/sims_",
                            scenario, "_stepCC_MT_meanpars.csv", sep = "")
        future <- read_csv(pathfuture)[,-1]
        future$species <- factor(future$species, levels = spnames)

        vegmat_swcc <- matrix(0,
                              ncol = length(spnames),
                              nrow = length(levels(factor(future$year))))


        for (yr in 1:length(levels(factor(future$year))) ){
                vegmat_swcc[yr,] <- with(subset(future,
                                                year == levels(factor(future$year))[yr]),
                                         tapply(cover_cm2, species, sum))
        }

        vegmat_swcc[is.na(vegmat_swcc)] <- 0



        pathfuture <- paste("simulations/megaturf/sims_",
                            scenario, "_slowCC_MT_meanpars.csv", sep = "")
```

```
        future <- read_csv(pathfuture)[,-1]
        future$species <- factor(future$species, levels = spnames)

        vegmat_gradcc <- matrix(0,
                                ncol = length(spnames),
                                nrow = length(levels(factor(future$year))))


        for (yr in 1:length(levels(factor(future$year))) ){
                vegmat_gradcc[yr,] <- with(subset(future,
                                                  year == levels(
                                                          factor(future$year))[yr]),
                                           tapply(cover_cm2, species, sum))
        }

        vegmat_gradcc[is.na(vegmat_gradcc)] <- 0




        pathfuture <- paste("simulations/megaturf/sims_",
                            scenario,
                            "_slowCC_nolags_MT_meanpars.csv", sep = "")
        future <- read_csv(pathfuture)[,-1]
        future$species <- factor(future$species, levels = spnames)

        vegmat_gradcc_nolags <- matrix(0,
                                       ncol = length(spnames),
                                       nrow = length(levels(factor(future$year))))


        for (yr in 1:length(levels(factor(future$year))) ){
                vegmat_gradcc_nolags[yr,] <- with(subset(
                        future, year == levels(factor(future$year))[yr]),
                                                  tapply(cover_cm2, species, sum))
        }

        vegmat_gradcc_nolags[is.na(vegmat_gradcc_nolags)] <- 0


        # Combine matrices from different simulation types
        vegmatrix <- rbind(vegmat_gradcc, vegmat_swcc, vegmat_gradcc_nolags)
        colnames(vegmatrix) <- spnames
        pathmat <- paste("processed-data/ordidyn-drivers/megaturf",
                         scenario, ".rds", sep = "")
        saveRDS(vegmatrix, pathmat)

}
```

4. Code to reproduce result figures

Here is the code to reproduce figures 2 to 5 in the results section of the main text.

Figure 2

Trajectories of alpine plant community responses to contrasting climate change scenarios for the 21st century.

```r
# Load climate time series (RCP scenarios)
clim26 <- read_csv("processed-data/rcp26TS.csv")
clim45 <- read_csv("processed-data/rcp45TS.csv")
clim85 <- read_csv("processed-data/rcp85TS.csv")

# Load time series of future community dynamics
future26 <- read_csv(
        "simulations/megaturf/sims_26_slowCC_MT_meanpars.csv")[,-1]
future45 <- read_csv(
        "simulations/megaturf/sims_45_slowCC_MT_meanpars.csv")[,-1]
future85 <- read_csv(
        "simulations/megaturf/sims_85_slowCC_MT_meanpars.csv")[,-1]

# Calculate Shannon diversity index
source('scripts/h.R')
h26 <- shannon(future26)
h45 <- shannon(future45)
h85 <- shannon(future85)

# Figure 2AB
rcp_cols <- c(rgb(50,155,255,150, maxColorValue = 255),
              rgb(255,165,0,150, maxColorValue = 255),
              rgb(139,0,0,150, maxColorValue = 255))


par(mfrow=c(2,1), mar = c(1.5,3.2,0,1), oma = c(2.2,0,1,0), cex.lab = 0.85,
    mgp = c(2, 0.75, 0), cex.axis = 0.85, xpd = NA)


plot(NA, xlim = c(2017, 2098), ylim = c(11, 18),
     xlab = "", ylab = "Summer temperature (ºC)",
     xaxt = 'n')
axis(1, at = c(2020, 2030, 2040, 2050, 2060, 2070, 2080, 2090, 2100),
     cex.axis= 0.5, labels = F)


lines(clim26$year, clim26$tempLS,
      col = rcp_cols[1],
      lwd = 1.5, lty = 1)
lines(clim45$year, clim45$tempLS,
      col = rcp_cols[2],
      lwd = 1.5, lty = 1)
lines(clim85$year, clim85$tempLS,
      col = rcp_cols[3],
      lwd = 1.5, lty = 1)


text(2090, 11.8, 'RCP 2.6',
     col = rcp_cols[1],
     font = 2, cex = 0.6)
```

```r
text(2090, 15.7, 'RCP 4.5',
     col = rcp_cols[2],
     font = 2, cex = 0.6)


text(2090, 17.8, 'RCP 8.5',
     col = rcp_cols[3],
     font = 2, cex = 0.6)

mtext('(A) Warming trajectories', adj = 0, cex = 0.75, font = 2)

# Species diversity
plot(NA, type = "n",
     ylab = "Shannon diversity", xlab = "Year",
     xlim = c(1,82), ylim = c(0, 1.5), xaxt = "n", xpd =NA)

lines(1:82, h26,
      col = rcp_cols[1], lwd = 1.5, lty = 1)
lines(1:82, h45,
      col = rcp_cols[2], lwd = 1.5, lty = 1)
lines(1:82, h85,
      col = rcp_cols[3], lwd = 1.5, lty = 1)

text(65, 0.9, 'RCP 2.6',
     col = rcp_cols[1],
     font = 2, cex = 0.6)

text(74, 1.38, 'RCP 4.5',
     col = rcp_cols[2],
     font = 2, cex = 0.6)


text(74, 0.55, 'RCP 8.5',
     col = rcp_cols[3],
     font = 2, cex = 0.6)

axis(1, at = c(4, 24, 44, 64, 84), labels = c('2020', '2040', '2060', '2080', '2100'))
axis(1, at = c(14, 34, 54, 74), cex.axis= 0.5, labels = F)
mtext('(B) Diversity trajectories', adj = 0, cex = 0.75, font = 2)
```

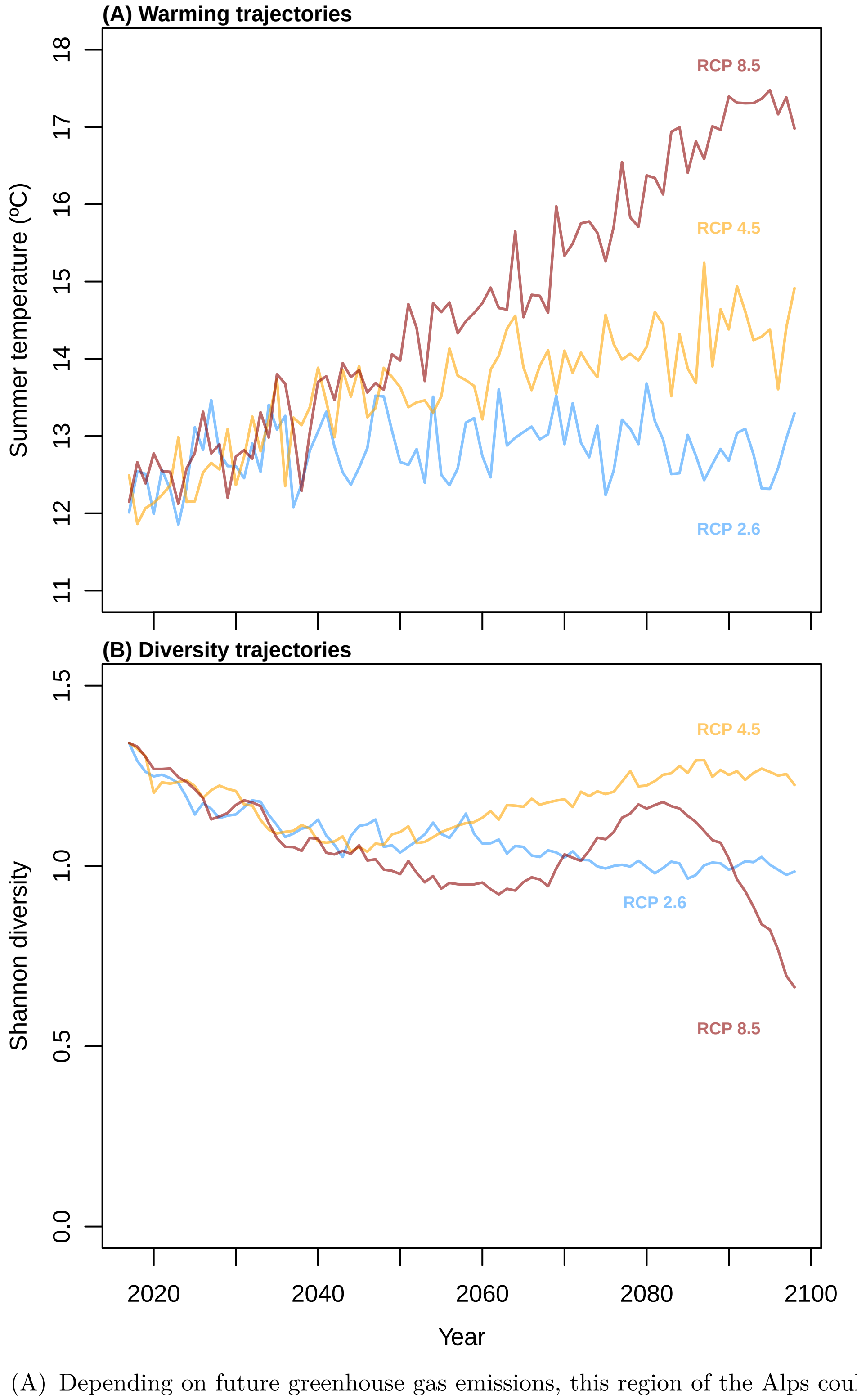

(A) Depending on future greenhouse gas emissions, this region of the Alps could experience contrasting climate futures, warming from 0.5 (in RCP 2.6, solid line) to 2.3 (in RCP 4.5, dashed line) and potentially even 4.8 ºC (in RCP 8.5, dotted line) by the end of the century relative to experimental period (2017 to 2020). (B) Increases in temperature under the contrasting climate change scenarios were paralleled by decreases in the diversity of the simulated alpine communities.

```r
library(ecodist)
library(rethinking)
library(readr)

clim26 <- read_csv('processed-data/rcp26TS.csv')
clim45 <- read_csv('processed-data/rcp45TS.csv')
clim85 <- read_csv('processed-data/rcp85TS.csv')

vegmatrix <- readRDS("processed-data/ordidyn-rcp/vegmatrix_MT.rds")

# Calculate distance matrix and use it to do a principal coordinate analysis
DM <- distance(vegmatrix, method="euclidean")
PCO <- pco(DM)

# Coordinates in ordination = eigenvectors*eigenvalues
axis1 <- PCO$vectors[,1] * PCO$values[1]
axis2 <- PCO$vectors[,2] * PCO$values[2]

# Species vectors
species.arrows <- vf(PCO$vectors[,1:2], vegmatrix, nperm=0)

par(mfrow = c(1,1), mar = c(2.7,0.1,0.1,0.5), oma = c(0.5, 1.2, 1,1),
    cex.axis = 0.5, xpd = NA)

plot(axis1, axis2,
     xlab="",
     ylab="",
     type = "n",
     xlim = c(min(axis1)*2, max(axis1)*1.1),
     ylim = c(min(axis2)*1.1, max(axis2)*1.1),
     xaxt = "n", yaxt = "n") # c(-200, 120)


# Taxa's vectors
plot.vf(species.arrows[species.arrows[,"r"]>0.9,],
        ascale = 1.5, cex = 0.0001, length = 0.05,
        lwd = 2.5,
        col = col.alpha('gray', 0.75))

mtext("(C) Community trajectories", adj = 0, font = 2, cex = 0.85)

timecolor <- colorRamp(c('cyan', 'darkred'), alpha = F, interpolate = 'linear')


# RCP26
for (y in seq(from=1, to = 24, by = 3)) {
        points(axis1[y],
               axis2[y],
               col = rcp_cols[1],
               pch = 16, cex = 0.8)
}
```

```r
for (y in seq(from=25, to = 54, by = 3)) {
        points(axis1[y],
               axis2[y],
               col = rcp_cols[1],
               pch = 17, cex = 0.8)
}

for (y in seq(from=55, to = 82, by = 3)) {
        points(axis1[y],
               axis2[y],
               col = rcp_cols[1],
               pch = 15, cex = 0.8)
}

lines(axis1[seq(from=1, to = 82, by = 3)],
      axis2[seq(from=1, to = 82, by = 3)],
      col = rcp_cols[1], lwd = 0.75)

# RCP 4.5
for (y in seq(from=1, to = 24, by = 3)) {
        points(axis1[y+82],
               axis2[y+82],
               col = rcp_cols[2],
               pch = 16, cex = 0.8)
}

for (y in seq(from=25, to = 54, by = 3)) {
        points(axis1[y+82],
               axis2[y+82],
               col = rcp_cols[2],
               pch = 17, cex = 0.8)
}

for (y in seq(from=55, to = 82, by = 3)) {
        points(axis1[y+82],
               axis2[y+82],
               col = rcp_cols[2],
               pch = 15, cex = 0.8)
}

lines(axis1[seq(from=1, to = 82, by = 3)+82],
      axis2[seq(from=1, to = 82, by = 3)+82],
      col = rcp_cols[2], lwd = 0.75)


# RCP 8.5
for (y in seq(from=1, to = 24, by = 3)) {
        points(axis1[y+164],
               axis2[y+164],
               col = rcp_cols[3],
               pch = 19, cex = 0.8)
}
```

```
for (y in seq(from=25, to = 54, by = 3)) {
        points(axis1[y+164],
               axis2[y+164],
               col = rcp_cols[3],
               pch = 17, cex = 0.8)
}


for (y in seq(from=55, to = 82, by = 3)) {
        points(axis1[y+164],
               axis2[y+164],
               col = rcp_cols[3],
               pch = 15, cex = 0.8)
}

lines(axis1[seq(from=1, to = 82, by = 3)+164],
      axis2[seq(from=1, to = 82, by = 3)+164],
      col = rcp_cols[3], lwd = 0.75)

mtext(paste("Axis 2 (", round((100*PCO$values/sum(PCO$values))[2], 1),
            "% of variation)", sep = ""),
      2, adj = 0.5, line = 0.25, cex = 0.85)

mtext(paste("Axis 1 (", round((100*PCO$values/sum(PCO$values))[1], 1),
            "% of variation)", sep = ""),
      1, line = 0.25, cex = 0.85)


# Add symbol legend
legend("topright",
       c('RCP 2.6', 'RCP 4.5', 'RCP 8.5'),
       pch = 19, col = c(rcp_cols[1],
                         rcp_cols[2],
                         rcp_cols[3]),
       cex = 0.75)


legend("bottomleft",
       c('2017 - 2040', '2041 - 2070', '2071 - 2098'),
       pch = c(16, 17, 15),
       cex = 0.75)
```

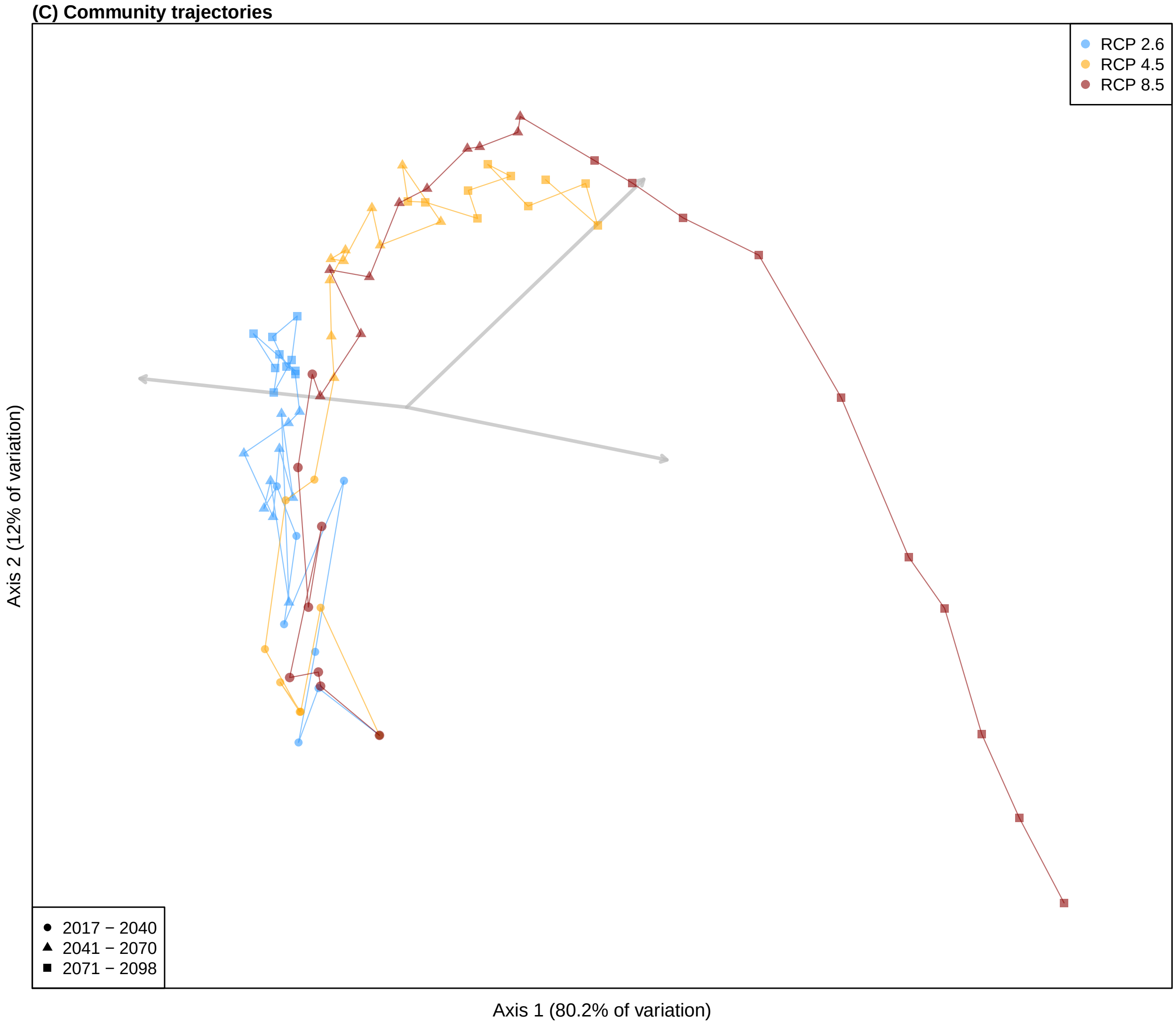

Principal coordinates ordination based on Euclidean distances showing the trajectory of simulated alpine communities under the different climate change scenarios. Each point corresponds to a year.

Figure 3

Dynamics of total cover for different taxa under different climate change scenarios. These trajectories of summer temperatures in the region under these scenarios is shown in figure 2A. For RCP 4.5, dynamics without lags are shown with dotted lines, while dynamics following stepwise change are shown with dashed lines.

```
library(readr)
library(rethinking)
source("scripts/spp_simul.R")
spdyn26 <- read_csv('simulations/megaturf/sims_26_slowCC_MT_meanpars.csv')
spdyn45 <- read_csv('simulations/megaturf/sims_45_slowCC_MT_meanpars.csv')
spdyn85 <- read_csv('simulations/megaturf/sims_85_slowCC_MT_meanpars.csv')
spdyn85_nolag <- read_csv("simulations/megaturf/sims_45_slowCC_nolags_MT_meanpars.csv")
spdyn85_step <- read_csv("simulations/megaturf/sims_45_stepCC_MT_meanpars.csv")
```

```r
spnames <- levels(factor(spdyn26$species))[-2]

rcp_cols <- c(rgb(0,255,255,150, maxColorValue = 255),
              rgb(255,165,0,150, maxColorValue = 255),
              rgb(139,0,0,150, maxColorValue = 255))

panel_letters <- c('(A)', '(B)', '(C)',
                   '(D)','(E)', '(F)',
                   '(G)', '(H)', '(I)')


par(mfrow = c(3,3), mar = c(1,1,1,0.5), oma = c(3 , 3.1, 0, 9), cex.lab = 1.25,
    cex.axis = 1)

for (s in spnames){
        plot(NA, xlim = c(2017,2098), ylim = c(0.5, 11.5),
             ylab = "", xlab = '', xaxt = 'n', yaxt = 'n')
        axis(1, at = seq(2020, 2100, by=10), labels = F)
        axis(2, at = c(2,4,6,8,10), cex.axis= 0.5, labels = F)
        mtext(s, adj = 0.5, cex = 0.5, font = 3)
        mtext(panel_letters[which(spnames==s)],
              font = 2, adj = 0, cex = 0.5)


        if(which(spnames==s) %in% c(1,4,7)){
                mtext(expression(paste('log cover (', cm^2, ')')), 2, line = 2.5,
                      cex = 0.8)
                axis(2, at = c(2,4,6,8,10), labels = T, las = 1)
        }


        if(which(spnames==s) %in% c(7:9)){
                mtext("Year", 1, line = 2.5, cex = 0.8)
                axis(1, at = seq(2020, 2100, by = 20), labels = T)
        }


        if(which(spnames==s)==6){
                legend(2100, 8, xpd = NA,
                       legend = c("RCP 2.6",
                                  "RCP 4.5",
                                  "RCP 8.5"),
                       lty = c(1,1,1), lwd = 1.5,
                       col = rcp_cols,
                       bty = 'n', xjust = 0, yjust = 0.5,
                       y.intersp = 1.25, cex = 0.9)


                legend(2100, 2, xpd = NA,
                       legend = c("RCP 4.5, stepwise",
                                  "RCP 4.5, no lags"),
                       lty = c(2,3), lwd = 1.5,
                       col = rcp_cols[2],
```

```
                      bty = 'n', xjust = 0, yjust = 0.5,
                      y.intersp = 1.25, cex = 0.9)
        }

        if(sum(spdyn26$species==s) > 0) {

                lines(2017:(max(subset(spdyn26, species == s)$year)),
                      log(with(subset(spdyn26, species == s),
                               tapply(cover_cm2, year, sum))),
                      col = rcp_cols[1], lwd = 1.5)

                lines(2017:(max(subset(spdyn45, species == s)$year)),
                      log(with(subset(spdyn45, species == s),
                               tapply(cover_cm2, year, sum))),
                      col = rcp_cols[2], lwd = 1.5)

                lines(2017:(max(subset(spdyn85, species == s)$year)),
                      log(with(subset(spdyn85, species == s),
                               tapply(cover_cm2, year, sum))),
                      col = rcp_cols[3], lwd = 1.5)

                lines(2017:(max(subset(spdyn85_nolag, species == s)$year)),
                      log(with(subset(spdyn85_nolag, species == s),
                               tapply(cover_cm2, year, sum))),
                      col = rcp_cols[2], lwd = 1.5, lty = 3)


                lines(2017:(max(subset(spdyn85_step, species == s)$year)),
                      log(with(subset(spdyn85_step, species == s),
                               tapply(cover_cm2, year, sum))),
                      col = rcp_cols[2], lwd = 1.5, lty = 2)
        }

}
```

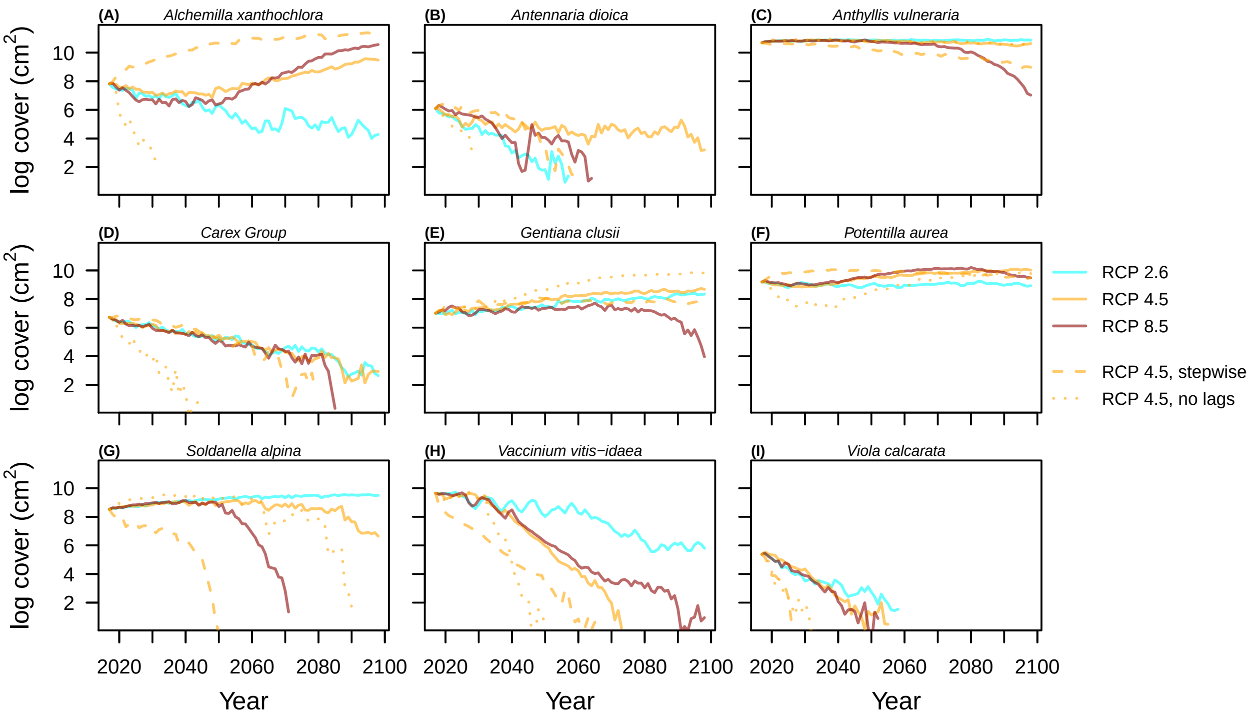

Figure 4

Principal Coordinates Analysis based on Euclidean distances of simulated community dynamics from 2017 to 2098 under (1) RCP 4.5 climate with full influence of demographic and competitive lags, (2) RCP 4.5 climate with a reduced influence of lags, in which the community reaches quasiequilibrium with annual climatic conditions, and (3) stepwise change to end-of-century RCP 4.5 conditions from the beginning of simulations.

```r
# Euclidean ordination plot
# Calculate distance matrix and use it to do a principal coordinate analysis
vegmatrix <- readRDS("processed-data/ordidyn-drivers/megaturf45_11sp.rds")
DM <- distance(vegmatrix, method="euclidean")
PCO <- pco(DM)

# Coordinates in ordination = eigenvectors*eigenvalues
axis1 <- PCO$vectors[,1] * PCO$values[1]
axis2 <- PCO$vectors[,2] * PCO$values[2]

species.arrows <- vf(PCO$vectors[,1:2], vegmatrix, nperm=0)

par(mfrow = c(1,1), mar = c(0.5,0.1,0.1,0), oma = c(0.5, 0.9, 0.5,0.2),
    cex.axis = 0.5, xpd = NA)

plot(axis1, axis2,
     xlab="",
     ylab="",
     type = "n",
     xlim = c(min(axis1)*2, max(axis1)*1.1),
     ylim = c(min(axis2)*1.1, max(axis2)*1.1),
     xaxt = 'n',
     yaxt = 'n')
```

```r
# Taxa's vectors
plot.vf(species.arrows[species.arrows[,"r"]>0.9,],
        ascale = 1, cex = 0.0001, length = 0.05,
        lwd = 1.5,
        col = col.alpha('gray', 0.75))

# Gradual, lags
for (y in 1:24) {
        points(axis1[y],
               axis2[y],
               col = rgb(255,165,0,150, maxColorValue = 255),
               pch = 16, cex = 0.8)
}


for (y in 25:54) {
        points(axis1[y],
               axis2[y],
               col = rgb(255,165,0,150, maxColorValue = 255),
               pch = 17, cex = 0.8)
}

for (y in 55:82) {
        points(axis1[y],
               axis2[y],
               col = rgb(255,165,0,150, maxColorValue = 255),
               pch = 15, cex = 0.8)
}

lines(axis1[1:82],
      axis2[1:82])


# Stepwise, lags
for (y in 1:24) {
        points(axis1[y+82],
               axis2[y+82],
               col = rgb(255,165,0,150, maxColorValue = 255),
               pch = 16, cex = 0.8)
}


for (y in 25:54) {
        points(axis1[y+82],
               axis2[y+82],
               col = rgb(255,165,0,150, maxColorValue = 255),
               pch = 17, cex = 0.8)
}

for (y in 55:82) {
        points(axis1[y+82],
               axis2[y+82],
```

```
                col = rgb(255,165,0,150, maxColorValue = 255),
                pch = 15, cex = 0.8)
}

lines(axis1[(1:82)+82],
      axis2[(1:82)+82], lty = 2)

# Gradual, no lags
for (y in 1:24) {
        points(axis1[y+164],
               axis2[y+164],
               col = rgb(255,165,0,150, maxColorValue = 255),
               pch = 16, cex = 0.8)
}


for (y in 25:54) {
        points(axis1[y+164],
               axis2[y+164],
               col = rgb(255,165,0,150, maxColorValue = 255),
               pch = 17, cex = 0.8)
}

for (y in 55:82) {
        points(axis1[y+164],
               axis2[y+164],
               col = rgb(255,165,0,150, maxColorValue = 255),
               pch = 15, cex = 0.8)
}

lines(axis1[(1:82)+164],
      axis2[(1:82)+164], lty = 3)

mtext(paste("Axis 2 (", round((100*PCO$values/sum(PCO$values))[2], 1), "% of variation)",
            sep = ""),
      2, adj = 0.5, line = 0.1, cex = 0.75)

mtext(paste("Axis 1 (", round((100*PCO$values/sum(PCO$values))[1], 1), "% of variation)",
            sep = ""),
      1, line = 0.1, cex = 0.75)


# Add symbol legend
legend("bottomright",
       c('Gradual climate change',
         'Gradual climate change (no lags)',
         'Stepwise climate change'),
       lty = c(1, 3, 2), cex = 0.4, pt.cex = 0.8, y.intersp = 1.2)

legend("topright",
       c('2017 - 2040', '2041 - 2070', '2071 - 2098'),
       pch = c(16, 17, 15), col = rgb(255,165,0,150, maxColorValue = 255),
       cex = 0.4, pt.cex = 0.8, y.intersp = 1.2)
```

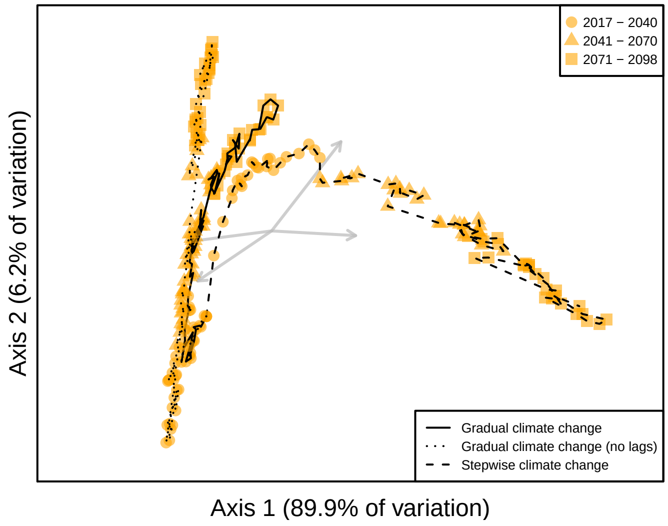

And the corresponding trajectories of diversity change.

```
future_slow <- read_csv("simulations/megaturf/sims_45_slowCC_MT_meanpars.csv")
future_step <- read_csv("simulations/megaturf/sims_45_stepCC_MT_meanpars.csv")
future_nolag <- read_csv("simulations/megaturf/sims_45_slowCC_nolags_MT_meanpars.csv")

source("scripts/h.R")
source("scripts/spp_simul.R")

hslow <- shannon(future_slow)
hstep <- shannon(future_step)
hnolag <- shannon(future_nolag)

linecol <- 'black'
linecol2 <- 'darkgray'

# Figure
par(mfrow=c(1,1), mar = c(2,2.4,0.6,0.5), cex.lab = 1,  cex.axis= 0.75,
    xpd = NA, las = 1)

plot(NA, type = "n",
     ylab = "Shannon diversity index", xlab = "Year",
     xlim = c(1,82), ylim = c(0, 1.5), xaxt = "n", xpd =NA)

lines(1:82, hslow,
      col = col.alpha(linecol, 0.75), lwd = 1.5, lty = 1)
lines(1:82, hstep,
      col = col.alpha(linecol, 0.75), lwd = 1.5, lty = 2)
lines(1:82, hnolag,
      col = col.alpha(linecol, 0.75), lwd = 1.5, lty = 3)

axis(1, at = c(4, 24, 44, 64, 84), cex.axis= 0.75,
     labels = c('2020', '2040', '2060', '2080', '2100'))
axis(1, at = c(14, 34, 54, 74), labels = F)
```

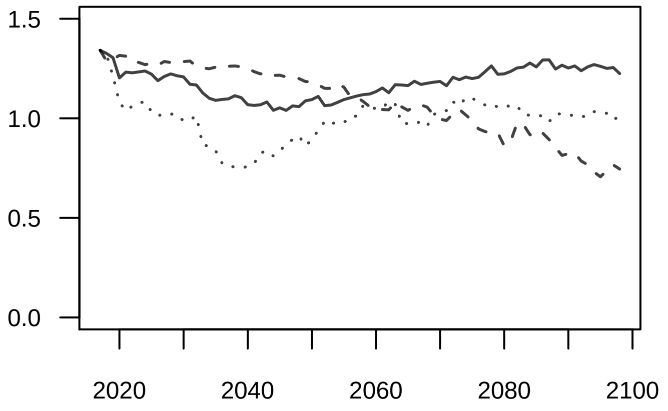

Supplementary figures

Taxa cover dynamics during burn-in period under constant climate (mean conditions 2017-2020 across scenarios).

```
burnin <- read_csv('simulations/megaturf/journey-to-equi_meanpars.csv')
source('scripts/spp_simul.R')

relaxt <- range(burnin$year)[2] - range(burnin$year)[1]

par(mfrow = c(3,4), mar = c(1,1,1,0.5), oma = c(4, 4, 1.5, 1), cex.lab = 1.25,
    cex.axis = 1)

for (s in spnames){
        plot(NA, xlim = c(0,relaxt), ylim = c(0, 1),
             ylab = "", xlab = '', xaxt = 'n', yaxt = 'n')
        axis(1, at = seq(0, relaxt, by = 6), cex.axis= 0.5, labels = F)
        axis(2, at = c(0,0.25,0.5,0.75,1), cex.axis= 0.5, labels = F)
        mtext(s, adj = 0.5, cex = 0.75, font = 3)

        if(which(spnames==s) %in% c(1,5,9)){
                mtext("Relative cover", 2, line = 3, cex = 0.8)
                axis(2, at = c(0,0.25,0.5,0.75,1), labels = T, las = 1)
        }

        if(which(spnames==s) %in% c(8:11)){
                mtext("Years", 1, line = 3)
                axis(1, at = seq(0, relaxt, by = 6), labels = F)
                axis(1, at = seq(0, relaxt, by = 6), labels = T)
        }

        lines(0:(length(unique(subset(burnin, species == s)$year))-1),
             (with(subset(burnin, species == s), tapply(cover_cm2, year, sum)))/64000,
             lwd = 1.5)

}
```

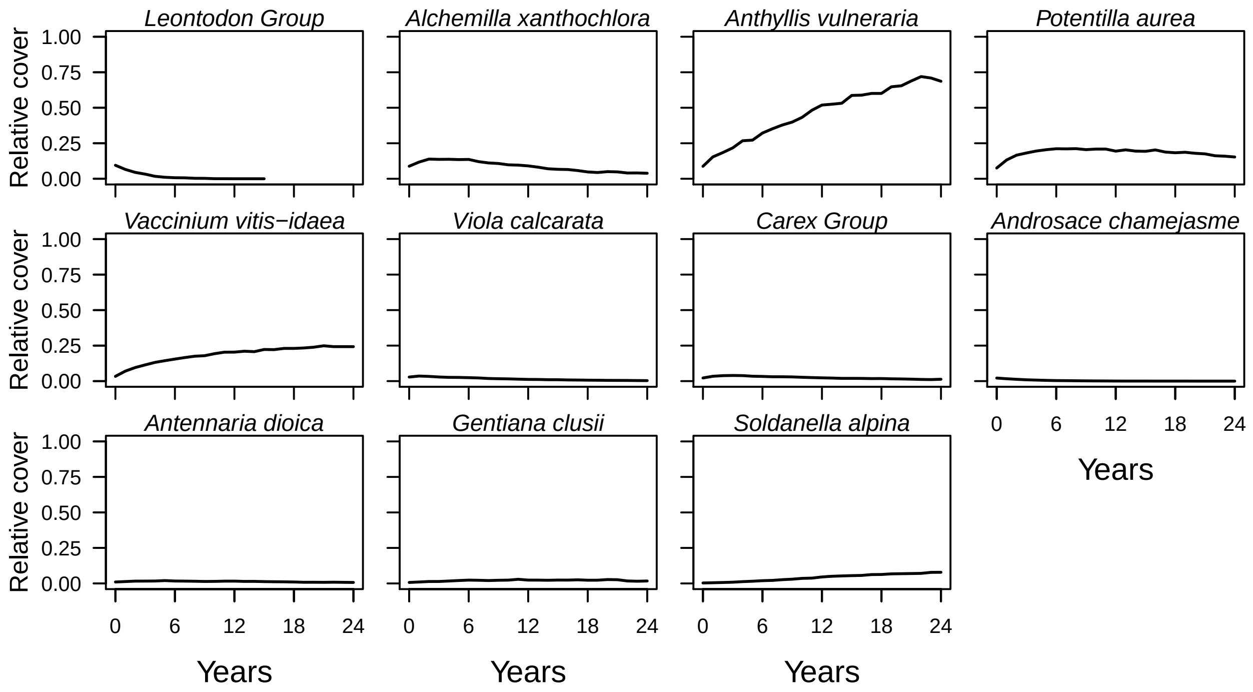

Taxa cover and frequency dynamics under different RCP scenarios. For each scenario, dynamics are shown under gradual change with and without lags, as well as following stepwise change to end-of-cenntury conditions.

```
future_slow26 <- read_csv("simulations/megaturf/sims_26_slowCC_MT_meanpars.csv")
```

```
## Warning: Missing column names filled in: 'X1' [1]

## Parsed with column specification:
## cols(
##   X1 = col_double(),
##   species = col_character(),
##   x = col_double(),
##   y = col_double(),
##   year = col_double(),
##   cover_cm2 = col_double(),
##   quadrant_id = col_character(),
##   unique_id = col_character()
## )
```

```
future_step26 <- read_csv("simulations/megaturf/sims_26_stepCC_MT_meanpars.csv")
```

```
## Warning: Missing column names filled in: 'X1' [1]

## Parsed with column specification:
## cols(
##   X1 = col_double(),
##   species = col_character(),
##   x = col_double(),
##   y = col_double(),
##   year = col_double(),
##   cover_cm2 = col_double(),
##   quadrant_id = col_character(),
##   unique_id = col_character()
## )
```

```r
future_nolag26 <- read_csv("simulations/megaturf/sims_26_slowCC_nolags_MT_meanpars.csv")
```

```
## Warning: Missing column names filled in: 'X1' [1]
```

```
## Parsed with column specification:
## cols(
##   X1 = col_double(),
##   species = col_character(),
##   x = col_double(),
##   y = col_double(),
##   year = col_double(),
##   cover_cm2 = col_double(),
##   quadrant_id = col_character(),
##   unique_id = col_character()
## )
```

```r
future_slow45 <- read_csv("simulations/megaturf/sims_45_slowCC_MT_meanpars.csv")
```

```
## Warning: Missing column names filled in: 'X1' [1]
```

```
## Parsed with column specification:
## cols(
##   X1 = col_double(),
##   species = col_character(),
##   x = col_double(),
##   y = col_double(),
##   year = col_double(),
##   cover_cm2 = col_double(),
##   quadrant_id = col_character(),
##   unique_id = col_character()
## )
```

```r
future_step45 <- read_csv("simulations/megaturf/sims_45_stepCC_MT_meanpars.csv")
```

```
## Warning: Missing column names filled in: 'X1' [1]
```

```
## Parsed with column specification:
## cols(
##   X1 = col_double(),
##   species = col_character(),
##   x = col_double(),
##   y = col_double(),
##   year = col_double(),
##   cover_cm2 = col_double(),
##   quadrant_id = col_character(),
##   unique_id = col_character()
## )
```

```r
future_nolag45 <- read_csv("simulations/megaturf/sims_45_slowCC_nolags_MT_meanpars.csv")
```

```
## Warning: Missing column names filled in: 'X1' [1]
```

```
## Parsed with column specification:
## cols(
##   X1 = col_double(),
##   species = col_character(),
##   x = col_double(),
##   y = col_double(),
```

```
##   year = col_double(),
##   cover_cm2 = col_double(),
##   quadrant_id = col_character(),
##   unique_id = col_character()
## )
```

```r
future_slow85 <- read_csv("simulations/megaturf/sims_85_slowCC_MT_meanpars.csv")
```

```
## Warning: Missing column names filled in: 'X1' [1]

## Parsed with column specification:
## cols(
##   X1 = col_double(),
##   species = col_character(),
##   x = col_double(),
##   y = col_double(),
##   year = col_double(),
##   cover_cm2 = col_double(),
##   quadrant_id = col_character(),
##   unique_id = col_character()
## )
```

```r
future_step85 <- read_csv("simulations/megaturf/sims_85_stepCC_MT_meanpars.csv")
```

```
## Warning: Missing column names filled in: 'X1' [1]

## Parsed with column specification:
## cols(
##   X1 = col_double(),
##   species = col_character(),
##   x = col_double(),
##   y = col_double(),
##   year = col_double(),
##   cover_cm2 = col_double(),
##   quadrant_id = col_character(),
##   unique_id = col_character()
## )
```

```r
future_nolag85 <- read_csv("simulations/megaturf/sims_85_slowCC_nolags_MT_meanpars.csv")
```

```
## Warning: Missing column names filled in: 'X1' [1]

## Parsed with column specification:
## cols(
##   X1 = col_double(),
##   species = col_character(),
##   x = col_double(),
##   y = col_double(),
##   year = col_double(),
##   cover_cm2 = col_double(),
##   quadrant_id = col_character(),
##   unique_id = col_character()
## )
```

```r
spnames <- levels(factor(future_step26$species))

panel_letters <- c('(A)', '(B)', '(C)',
                   '(D)','(E)', '(F)',
```

```r
                   '(G)', '(H)', '(I)',
                   '(J)', '(K)', '(L)',
                   '(M)', '(N)', '(O)')


par(mfrow = c(10,3), mar = c(0.5,1,0.5,0.5), oma = c(3 , 3.1, 1.5, 9), cex.lab = 1.25,
    cex.axis = 1)
for (s in spnames){

        # RCP 2.6
        plot(NA, xlim = c(2017,2098), ylim = c(0.5, 11.5),
             ylab = "", xlab = '', xaxt = 'n', yaxt = 'n')
        axis(1, at = seq(2020, 2100, by=10), labels = F)
        mtext(expression(paste('log cover (', cm^2, ')')), 2, line = 2.5, cex = 0.5)
        axis(2, at = c(2,4,6,8,10), labels = T, las = 1)

        lines(2017:(max(subset(future_slow26, species == s)$year)),
              log(with(subset(future_slow26, species == s),
                       tapply(cover_cm2, year, sum))),
              lwd = 1.5)

        lines(2017:(max(subset(future_step26, species == s)$year)),
              log(with(subset(future_step26, species == s),
                       tapply(cover_cm2, year, sum))),
              lwd = 1.5, lty = 2)


        if(which(spnames==s) == 1){ mtext("RCP 2.6", line = 0.25, font = 2, cex = 0.85) }
        if(which(spnames==s) == 10){
                mtext("Year", 1, line = 2.5, cex = 0.8)
                axis(1, at = seq(2020, 2100, by = 20), labels = T)
        }

        # RCP 4.5
        plot(NA, xlim = c(2017,2098), ylim = c(0.5, 11.5),
             ylab = "", xlab = '', xaxt = 'n', yaxt = 'n')
        axis(1, at = seq(2020, 2100, by=10), labels = F)
        axis(2, at = c(2,4,6,8,10), cex.axis= 0.5, labels = F)

        lines(2017:(max(subset(future_slow45, species == s)$year)),
              log(with(subset(future_slow45, species == s),
                       tapply(cover_cm2, year, sum))),
              lwd = 1.5)

        lines(2017:(max(subset(future_step45, species == s)$year)),
              log(with(subset(future_step45, species == s),
                       tapply(cover_cm2, year, sum))),
              lwd = 1.5, lty = 2)

        lines(2017:(max(subset(future_nolag45, species == s)$year)),
              log(with(subset(future_nolag45, species == s),
                       tapply(cover_cm2, year, sum))),
              lwd = 1.5, lty = 3)
```

```
        if(which(spnames==s) == 1){ mtext("RCP 4.5", line = 0.25, font = 2, cex = 0.85) }
        if(which(spnames==s) == 10){
                mtext("Year", 1, line = 2.5, cex = 0.8)
                axis(1, at = seq(2020, 2100, by = 20), labels = T)
        }

        # RCP 8.5
        plot(NA, xlim = c(2017,2098), ylim = c(0.5, 11.5),
             ylab = "", xlab = '', xaxt = 'n', yaxt = 'n')
        axis(1, at = seq(2020, 2100, by=10), labels = F)
        axis(2, at = c(2,4,6,8,10), cex.axis= 0.5, labels = F)

        lines(2017:(max(subset(future_slow85, species == s)$year)),
              log(with(subset(future_slow85, species == s),
                       tapply(cover_cm2, year, sum))),
               lwd = 1.5)

        lines(2017:(max(subset(future_step85, species == s)$year)),
              log(with(subset(future_step85, species == s),
                       tapply(cover_cm2, year, sum))),
               lwd = 1.5, lty = 2)

        lines(2017:(max(subset(future_nolag85, species == s)$year)),
              log(with(subset(future_nolag85, species == s),
                       tapply(cover_cm2, year, sum))),
               lwd = 1.5, lty = 3)


        if(which(spnames==s) == 1){ mtext("RCP 8.5", line = 0.25, font = 2, cex = 0.85) }

        mtext(s, 4, adj = 0.5, cex = 0.6, font = 3, las = 2, line = 5)



        if(which(spnames==s) == 10){
                mtext("Year", 1, line = 2.5, cex = 0.8)
                axis(1, at = seq(2020, 2100, by = 20), labels = T)
        }


}
```

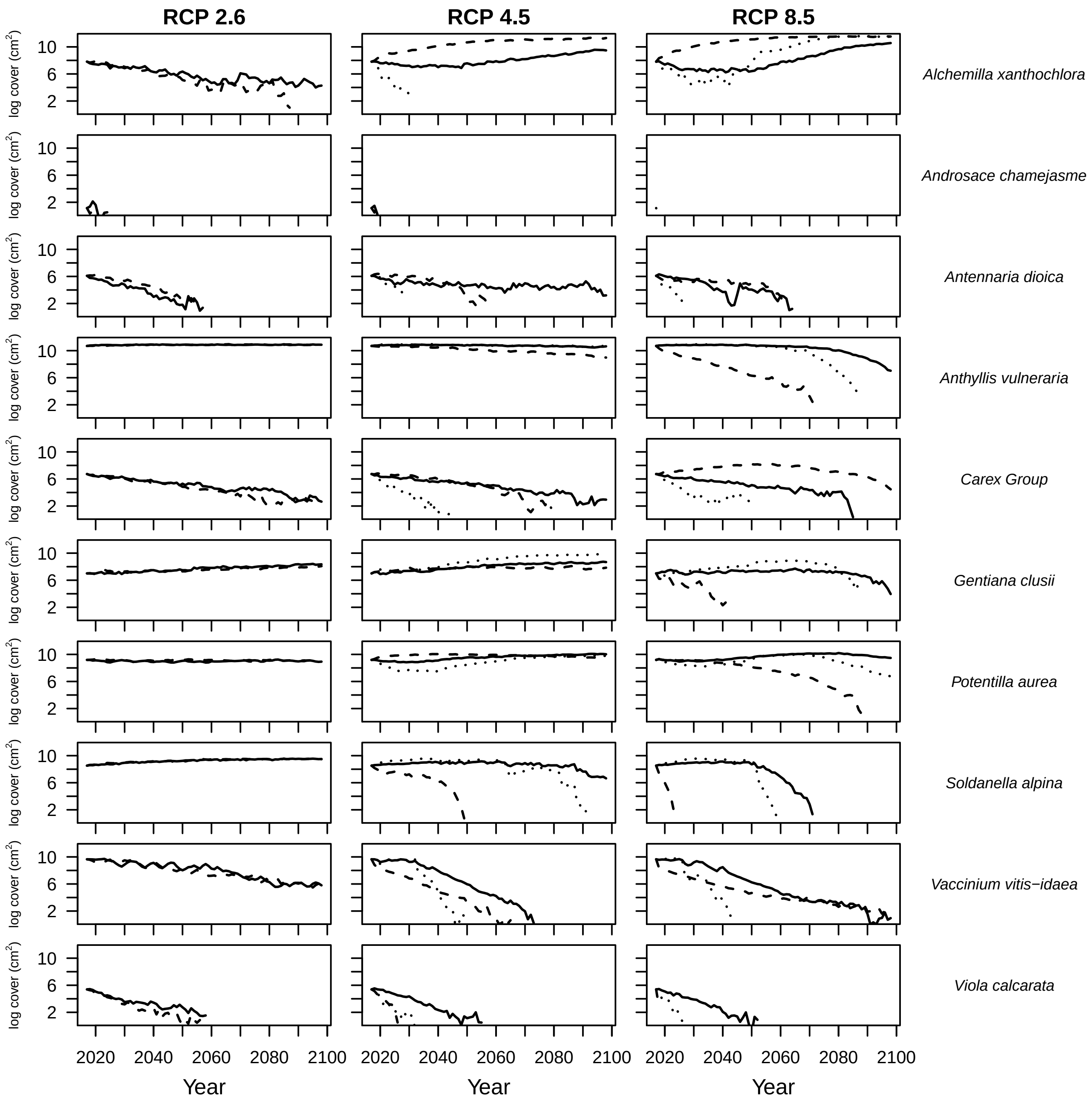


```
par(mfrow = c(10,3), mar = c(0.5,1,0.5,0.5), oma = c(3 , 3.1, 1.5, 9), cex.lab = 1.25,
    cex.axis = 1)

for (s in spnames){

        # RCP 2.6
        plot(NA, xlim = c(2017,2098), ylim = c(0, 1),
             ylab = "", xlab = '', xaxt = 'n', yaxt = 'n')
        axis(1, at = seq(2020, 2100, by=10), labels = F)
        mtext("Frequency", 2, line = 2.5, cex = 0.6)
        axis(2, at = c(0,0.5,1), labels = T, las = 1)

        lines(2017:(max(subset(future_slow26, species == s)$year)),
              (with(subset(future_slow26, species == s), table(year)))/2560,
```

```
        lwd = 1.5)

  lines(2017:(max(subset(future_step26, species == s)$year)),
        (with(subset(future_step26, species == s), table(year)))/2560,
        lwd = 1.5, lty = 2)


  if(which(spnames==s) == 1){ mtext("RCP 2.6", line = 0.25, font = 2, cex = 0.85) }
  if(which(spnames==s) == 10){
          mtext("Year", 1, line = 2.5, cex = 0.8)
          axis(1, at = seq(2020, 2100, by = 20), labels = T)
  }

  # RCP 4.5
  plot(NA, xlim = c(2017,2098), ylim = c(0, 1),
       ylab = "", xlab = '', xaxt = 'n', yaxt = 'n')
  axis(1, at = seq(2020, 2100, by=10), labels = F)
  axis(2, at = c(0,0.5,1), cex.axis= 0.5, labels = F)

  lines(2017:(max(subset(future_slow45, species == s)$year)),
        (with(subset(future_slow45, species == s), table(year)))/2560,
        lwd = 1.5)

  lines(2017:(max(subset(future_step45, species == s)$year)),
        (with(subset(future_step45, species == s), table(year)))/2560,
        lwd = 1.5, lty = 2)

  lines(2017:(max(subset(future_nolag45, species == s)$year)),
        (with(subset(future_nolag45, species == s), table(year)))/2560,
        lwd = 1.5, lty = 3)


  if(which(spnames==s) == 1){ mtext("RCP 4.5", line = 0.25, font = 2, cex = 0.85) }
  if(which(spnames==s) == 10){
          mtext("Year", 1, line = 2.5, cex = 0.8)
          axis(1, at = seq(2020, 2100, by = 20), labels = T)
  }

  # RCP 8.5
  plot(NA, xlim = c(2017,2098), ylim = c(0, 1),
       ylab = "", xlab = '', xaxt = 'n', yaxt = 'n')
  axis(1, at = seq(2020, 2100, by=10), labels = F)
  axis(2, at = c(0,0.5,1), cex.axis= 0.5, labels = F)

  lines(2017:(max(subset(future_slow85, species == s)$year)),
        (with(subset(future_slow85, species == s), table(year)))/2560,
        lwd = 1.5)

  lines(2017:(max(subset(future_step85, species == s)$year)),
        (with(subset(future_step85, species == s), table(year)))/2560,
        lwd = 1.5, lty = 2)

  lines(2017:(max(subset(future_nolag85, species == s)$year)),
```

```
                (with(subset(future_nolag85, species == s), table(year)))/2560,
                lwd = 1.5, lty = 3)


        if(which(spnames==s) == 1){ mtext("RCP 8.5", line = 0.25, font = 2, cex = 0.85) }

        mtext(s, 4, adj = 0.5, cex = 0.6, font = 3, las = 2, line = 5)



        if(which(spnames==s) == 10){
                mtext("Year", 1, line = 2.5, cex = 0.8)
                axis(1, at = seq(2020, 2100, by = 20), labels = T)
        }


}
```

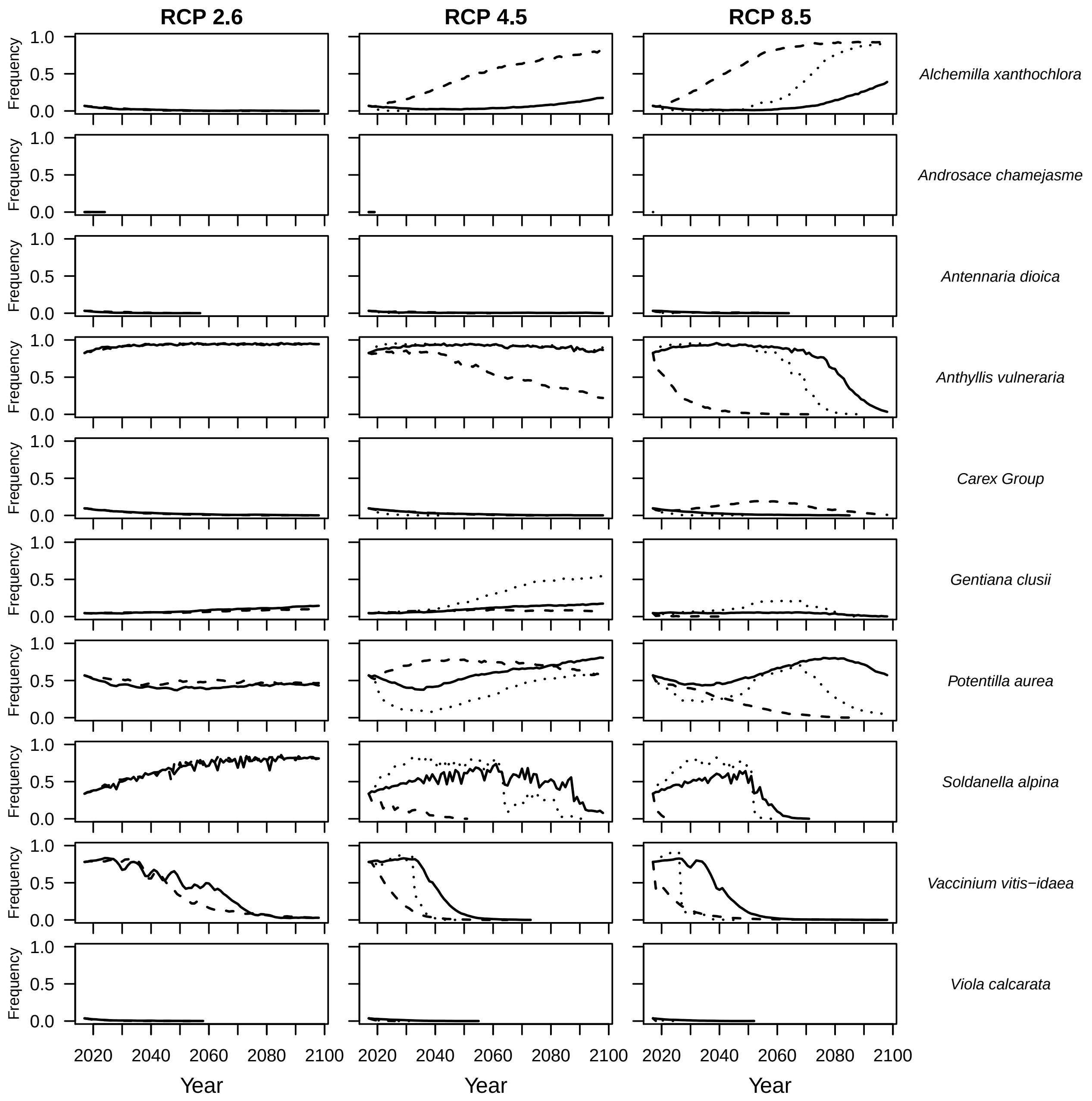

Climate-dependency of taxa demography and interactions with neighbors.

```
library(readr)

source("scripts/spp_modfits.R")

Nspp <- length(sppList)

# survival parameters
Spars=list(tef = matrix(NA,1e3,Nspp),
           ttp_a = matrix(NA,1e3,Nspp),
           ttp_b = matrix(NA,1e3,Nspp),
           k = matrix(NA,1e3,Nspp),
           b = matrix(NA,1e3,Nspp),
```

```
           a_0 = matrix(NA,1e3,Nspp),
           b_a = matrix(NA,1e3,Nspp),
           aC_0 = matrix(NA,1e3,Nspp),
           b_aC = matrix(NA,1e3,Nspp))

for(i in 1:Nspp){
        infile=paste("processed-data/params_",sppList[i],".rds",sep="")
        Sdata=readRDS(infile)

        Spars$tef[,i] = Sdata$s.tef[1:1e3]
        Spars$ttp_a[,i] = Sdata$s.ttp_a[1:1e3]
        Spars$ttp_b[,i] = Sdata$s.ttp_b[1:1e3]
        Spars$k[,i] = Sdata$s.k[1:1e3]
        Spars$b[,i] = Sdata$s.b[1:1e3]
        Spars$a_0[,i] = Sdata$s.a_0[1:1e3]
        Spars$b_a[,i] = Sdata$s.b_a[1:1e3]
        Spars$aC_0[,i] = Sdata$s.aC_0[1:1e3]
        Spars$b_aC[,i] = Sdata$s.b_aC[1:1e3]

} # next i

rm(Sdata)

# growth parameters
Nspp <- length(sppList)

# growth parameters
Gpars=list(b0 = matrix(NA,1e3,Nspp),
           tef = matrix(NA,1e3,Nspp),
           tsd = matrix(NA,1e3,Nspp),
           ttp = matrix(NA,1e3,Nspp),
           b = matrix(NA,1e3,Nspp),
           a_0 = matrix(NA,1e3,Nspp),
           b_a = matrix(NA,1e3,Nspp),
           aC_0 = matrix(NA,1e3,Nspp),
           b_aC = matrix(NA,1e3,Nspp),
           sigma = matrix(NA,1e3,Nspp))

for(i in 1:Nspp){
        infile=paste("processed-data/params_",sppList[i],".rds",sep="")
        Gdata=readRDS(infile)

        Gpars$b0[,i] = Gdata$g.b0[1:1e3]
        Gpars$tef[,i] = Gdata$g.tef[1:1e3]
        Gpars$tsd[,i] = Gdata$g.tsd[1:1e3]
        Gpars$ttp[,i] = Gdata$g.ttp[1:1e3]
        Gpars$b[,i] = Gdata$g.b[1:1e3]
        Gpars$a_0[,i] = Gdata$g.a_0[1:1e3]
        Gpars$b_a[,i] = Gdata$g.b_a[1:1e3]
        Gpars$aC_0[,i] = Gdata$g.aC_0[1:1e3]
        Gpars$b_aC[,i] = Gdata$g.b_aC[1:1e3]
        Gpars$sigma[,i] = Gdata$g.sigma[1:1e3]
```

```
} # next i

rm(Gdata)

# recruitment parameters
Rpars=list(a_0 = matrix(NA,1e3,Nspp),
           b_a = matrix(NA,1e3,Nspp),
           aC_0 = matrix(NA,1e3,Nspp),
           b_aC = matrix(NA,1e3,Nspp))

for(i in 1:Nspp){
        infile=paste("processed-data/params_",sppList[i],".rds",sep="")
        Gdata=readRDS(infile)


        Rpars$a_0[,i] = Gdata$r.a_0[1:1e3]
        Rpars$b_a[,i] = Gdata$r.b_a[1:1e3]
        Rpars$aC_0[,i] = Gdata$r.aC_0[1:1e3]
        Rpars$b_aC[,i] = Gdata$r.b_aC[1:1e3]

} # next i

rm(Gdata)


anbc <- read_csv("processed-data/anbc_clean.csv")
comments <- read_csv("data/comments-and-voles(04062021).csv")
disturbances = subset(comments, type %in% c('sensor', 'vole') &
                          level %in% c('cell', 'quadrant'))
disturbances$year[is.na(disturbances$year)] = substr(as.character(disturbances$date[
                                                        is.na(disturbances$year)]), 1, 4)

spp_clim_scales = read_csv('processed-data/spp_clim_scales.csv')


for (p in 1:4){

        spp_ids <- ((p*5 - 5) + 1):(p*5)

        par(oma = c(4.2,5.5,1,6.5), mar=c(1,0,0.5,0), mfrow = c(3,5), cex.axis = 1.5)

        ## Survival
        for (doSpp in spp_ids) {

            modspID <- which(sppList[doSpp] == sppList)

            dfile = paste('processed-data/', sppList[doSpp], '_DMD.csv', sep = '')
            d = read_csv(dfile)
            d = subset(d, x > 20 & x < 80 & y > 20 & y < 80)

            # Exclude ramets in grids with collars
            ## Alpha grids in Calanda plots
            d <- d[!(d$site == "Cal" & d$x < 50 & d$y > 50), ]
```

```
## Alpha or gamma grids in Nes plots
collarsG = c('Nes01.03', 'Nes02.03', 'Nes04.03',
             'Nes05.01', 'Nes08.01', 'Nes09.03')
collarsA = c('Nes03.01', 'Nes06.03', 'Nes07.03', 'Nes10.01')

d <- d[!(d$plot %in% collarsA & d$x < 50 & d$y > 50), ]
d <- d[!(d$plot %in% collarsG & d$x < 50 & d$y < 50), ]


# Exclude ramets that affected by disturbance

for (i in 1:nrow(disturbances)) {

        if (disturbances$level[i] == 'quadrant') {

                quadid = as.character(disturbances$quadrant_id[i])
                plotid = substr(quadid, 1, 8)
                gridid = substr(quadid, 10, 11)
                rowid = substr(quadid, 13, 13)
                colid = substr(quadid, 14, 14)
                qid = as.numeric(substr(quadid, 16, 16))

                # get quad coordinates
                xi = ifelse(gridid %in% c('BE', 'DE'), 50, 0)
                yi = ifelse(gridid %in% c('BE', 'AL'), 50, 0)

                xi = xi + as.numeric(colid)*10 - 5
                yi = yi + ifelse(rowid == "A", 45,
                             ifelse(rowid == "B", 35,
                                    ifelse(rowid == "C", 25,
                                           ifelse(rowid == "d", 15, 5))))

                xi = xi + ifelse(qid %in% c(1,3), -2.5, 2.5)
                yi = yi + ifelse(qid %in% c(3,4), -2.5, 2.5)

                rmi = which(d$plot == plotid & abs(d$x - xi) < 3 &
                              abs(d$y - yi) < 3 &
                              d$survival == 0 &
                                d$year + 1 == disturbances$year[i])

                if (length(rmi)>0) d = d[-rmi, ]

                rm(rmi, xi, yi, quadid, plotid,
                   gridid, rowid, colid, qid)

        }


        if (disturbances$level[i] == 'cell') {

                cellid = as.character(disturbances$cell_id[i])
                plotid = substr(cellid, 1, 8)
                gridid = substr(cellid, 10, 11)
```

```
                    rowid = substr(cellid, 13, 13)
                    colid = substr(cellid, 14, 14)

                    # get cell coordinates
                    xi = ifelse(gridid %in% c('BE', 'DE'), 50, 0)
                    yi = ifelse(gridid %in% c('BE', 'AL'), 50, 0)

                    xi = xi + as.numeric(colid)*10 - 5
                    yi = yi + ifelse(rowid == "A", 45,
                                 ifelse(rowid == "B", 35,
                                        ifelse(rowid == "C", 25,
                                               ifelse(rowid == "d", 15, 5))))

                    rmi = which(d$plot == plotid & abs(d$x - xi) < 5 &
                                  abs(d$y - yi) < 5 & d$survival == 0 &
                                  d$year + 1 == disturbances$year[i])

                    if (length(rmi)>0) d = d[-rmi, ]

                    rm(rmi, xi, yi, cellid, plotid, gridid, rowid, colid)
            }

    }


    cold = (12 - spp_clim_scales$muT.s[modspID])/
        spp_clim_scales$sdT.s[modspID]
    hot = (19 - spp_clim_scales$muT.s[modspID])/
        spp_clim_scales$sdT.s[modspID]

    wet = 0

    ## Survival
    plot(NA, type = "n",
         xlim = c(cold, hot), ylim = c(0,1), xlab = "",
         ylab = "", xaxt = "n", yaxt = "n")

    ticks = (c(13,15,17) - spp_clim_scales$muT.s[modspID])/
        spp_clim_scales$sdT.s[modspID]
    axis(1, at = ticks, labels = F)

    abline(v = (12.3 - spp_clim_scales$muT.s[modspID])/
               spp_clim_scales$sdT.s[modspID],
           lty = 3, col = 'darkgray', lwd = 1)

    cold26 = (12.3 + 0.9 - spp_clim_scales$muT.s[modspID])/
        spp_clim_scales$sdT.s[modspID]
    hot26 = (12.3 + 2.9 - spp_clim_scales$muT.s[modspID])/
        spp_clim_scales$sdT.s[modspID]
    polygon(c(cold26, cold26, hot26, hot26),
            c(-1,2,2,-1), col = col.alpha('blue'), border = NA)
    abline(v = (12.3 + 1.6 - spp_clim_scales$muT.s[modspID])/
               spp_clim_scales$sdT.s[modspID],
```

```
       lty = 3, col = col.alpha('blue', 0.5), lwd = 1)

cold45 = (12.3 + 2 - spp_clim_scales$muT.s[modspID])/
    spp_clim_scales$sdT.s[modspID]
hot45 = (12.3 + 4.2 - spp_clim_scales$muT.s[modspID])/
    spp_clim_scales$sdT.s[modspID]
polygon(c(cold45, cold45, hot45, hot45),
        c(-1,2,2,-1), col = col.alpha('orange'), border = NA)
abline(v = (12.3 + 2.9 - spp_clim_scales$muT.s[modspID])/
           spp_clim_scales$sdT.s[modspID],
       lty = 3, col = col.alpha('orange', 0.5), lwd = 1)

cold85 = (12.3 + 4.1 - spp_clim_scales$muT.s[modspID])/
    spp_clim_scales$sdT.s[modspID]
hot85 = (12.3 + 7.2 - spp_clim_scales$muT.s[modspID])/
    spp_clim_scales$sdT.s[modspID]
polygon(c(cold85, cold85, hot85, hot85),
        c(-1,2,2,-1), col = col.alpha('red'), border = NA)
abline(v = (12.3 + 5.3 - spp_clim_scales$muT.s[modspID])/
           spp_clim_scales$sdT.s[modspID],
       lty = 3, col = col.alpha('red', 0.5), lwd = 1)


if(doSpp == spp_ids[1]) {
        mtext("Survival \nprobability", side = 2, line = 3, cex = 0.75)
        axis(2, at = c(0,0.25, 0.5, 0.75, 1),
             labels = c("0.00","", "0.50", "", "1.00"),
             cex.axis = 1, las = 1)

}


mtext(domsp[modspID], 3, font = 3, cex = 0.4, line = 0.3,
      adj = nchar(domsp[modspID])/30)

panel_labels = c('(A)', '(B)', '(C)', '(D)', '(E)')
mtext(panel_labels[which(doSpp == spp_ids)],
      3, font = 2, cex = 0.65, adj = 0, line = 0.2)


d$temp_f = factor((d$tempLS - mean(d$tempLS))/sd(d$tempLS))

points(as.numeric(names(tapply(d$survival, d$temp_f, mean))),
       as.numeric(tapply(d$survival, d$temp_f, mean)),
       pch = 19, cex = (sqrt(table(d$temp_f))/50)*4,
       col = col.alpha('black', 0.2))
points(as.numeric(names(tapply(d$survival, d$temp_f, mean))),
       as.numeric(tapply(d$survival, d$temp_f, mean)),
       cex = (sqrt(table(d$temp_f))/50 )*4, col = "darkgray")



## Add model predictions
```

```r
        w_intra = mean(d$w)
        w_inter = summary(d$w.cinter)[c(1,4,6)]

        u_mu = 0
        temp = seq(cold, hot, length.out = 1e2)
        moist = wet

        for (crow in 1:length(w_inter)) {

            xposib = matrix(NA, nrow = 1e3, ncol = length(temp))
            for (ps in 1:1e3) {
                ttp = Spars$ttp_a[ps,doSpp] + Spars$ttp_b[ps,doSpp] * moist
                xposib[ps,] = Spars$tef[ps,doSpp] / (1 + exp(-Spars$k[ps,doSpp] *
                                                     (temp - ttp))) +
                        Spars$b[ps,doSpp] *  u_mu +
                        (Spars$a_0[ps,doSpp] + Spars$b_a[ps,doSpp] * temp)*w_intra +
                        (Spars$aC_0[ps,doSpp]  + Spars$b_aC[ps,doSpp] * temp)*
                    w_inter[crow] - 4


            }
            x.mu = apply(xposib, 2, mean)
            x.ci = apply(xposib, 2, PI)

            lty_crow = c(3,1,2)[crow]

            shade(exp(x.ci)/(1+exp(x.ci)), temp, col = col.alpha("gray", 0.25))
            lines(temp, exp(x.mu)/(1+exp(x.mu)),
                  lwd = 1.25, lty = lty_crow)


        }

    }

    ## Growth

    for (doSpp in spp_ids) {

        modspID <- which(sppList[doSpp] == sppList)

        dfile = paste('processed-data/', sppList[doSpp], '_DMD.csv', sep = '')
        d = read_csv(dfile)
        d = subset(d, x > 20 & x < 80 & y > 20 & y < 80)

        # Exclude ramets in grids with collars
        ## Alpha grids in Calanda plots
        d <- d[!(d$site == "Cal" & d$x < 50 & d$y > 50), ]

        ## Alpha or gamma grids in Nes plots
        collarsG = c('Nes01.03', 'Nes02.03', 'Nes04.03',
                     'Nes05.01', 'Nes08.01', 'Nes09.03')
        collarsA = c('Nes03.01', 'Nes06.03', 'Nes07.03', 'Nes10.01')
```

```r
d <- d[!(d$plot %in% collarsA & d$x < 50 & d$y > 50), ]
d <- d[!(d$plot %in% collarsG & d$x < 50 & d$y < 50), ]


# Exclude ramets that affected by disturbance
for (i in 1:nrow(disturbances)) {

        if (disturbances$level[i] == 'quadrant') {

                quadid = as.character(disturbances$quadrant_id[i])
                plotid = substr(quadid, 1, 8)
                gridid = substr(quadid, 10, 11)
                rowid = substr(quadid, 13, 13)
                colid = substr(quadid, 14, 14)
                qid = as.numeric(substr(quadid, 16, 16))

                # get quad coordinates
                xi = ifelse(gridid %in% c('BE', 'DE'), 50, 0)
                yi = ifelse(gridid %in% c('BE', 'AL'), 50, 0)

                xi = xi + as.numeric(colid)*10 - 5
                yi = yi + ifelse(rowid == "A", 45,
                                 ifelse(rowid == "B", 35,
                                        ifelse(rowid == "C", 25,
                                               ifelse(rowid == "D", 15, 5))))

                xi = xi + ifelse(qid %in% c(1,3), -2.5, 2.5)
                yi = yi + ifelse(qid %in% c(3,4), -2.5, 2.5)

                rmi = which(d$plot == plotid & abs(d$x - xi) < 3 &
                              abs(d$y - yi) < 3 & d$survival == 0 &
                              d$year + 1 == disturbances$year[i])

                if (length(rmi)>0) d = d[-rmi, ]

                rm(rmi, xi, yi, quadid, plotid, gridid, rowid, colid, qid)

        }


        if (disturbances$level[i] == 'cell') {

                cellid = as.character(disturbances$cell_id[i])
                plotid = substr(cellid, 1, 8)
                gridid = substr(cellid, 10, 11)
                rowid = substr(cellid, 13, 13)
                colid = substr(cellid, 14, 14)

                # get cell coordinates
                xi = ifelse(gridid %in% c('BE', 'DE'), 50, 0)
                yi = ifelse(gridid %in% c('BE', 'AL'), 50, 0)

                xi = xi + as.numeric(colid)*10 - 5
```

```
                 yi = yi + ifelse(rowid == "A", 45,
                                  ifelse(rowid == "B", 35,
                                         ifelse(rowid == "C", 25,
                                                ifelse(rowid == "D", 15, 5))))

                 rmi = which(d$plot == plotid & abs(d$x - xi) < 5 &
                               abs(d$y - yi) < 5 & d$survival == 0 &
                               d$year + 1 == disturbances$year[i])

                 if (length(rmi)>0) d = d[-rmi, ]

                 rm(rmi, xi, yi, cellid, plotid, gridid, rowid, colid)
        }

}

# For now, let's use only survivors
dG <- d[complete.cases(d$u2),]

# Fix erroneous cover values
if(sppList[doSpp] != "FesGro"){
        dG$u1[dG$u1 < 1.5625] <- 1.5625
        dG$u2[dG$u2 < 1.5625] <- 1.5625
}


dG$u1[dG$u1 < 0.390625] <- 0.390625
dG$u1[dG$u1 > 0.390625 & dG$u1 < 1.5625] <- 1.5625
dG$u1[dG$u1 > 1.5625 & dG$u1 < 3.125] <- 3.125
dG$u1[dG$u1 > 3.125 & dG$u1 < 6.25] <- 6.25
dG$u1[dG$u1 > 6.25 & dG$u1 < 12.5] <- 12.5

dG$u2[dG$u2 < 0.390625] <- 0.390625


cold = (12 - spp_clim_scales$muT.g[modspID])/spp_clim_scales$sdT.g[modspID]
hot = (19 - spp_clim_scales$muT.g[modspID])/spp_clim_scales$sdT.g[modspID]

plot(NA,
     xlim = c(cold, hot), ylim = c(0,3), xlab = "",
     ylab = "Growth", xaxt = "n", yaxt = "n")
ticks = (c(13,15,17) - spp_clim_scales$muT.g[modspID])/
    spp_clim_scales$sdT.g[modspID]
axis(1, at = ticks, labels = F)

abline(v = (12.3 - spp_clim_scales$muT.g[modspID])/
           spp_clim_scales$sdT.g[modspID],
       lty = 3, col = 'darkgray', lwd = 1)

cold26 = (12.3 + 0.9 - spp_clim_scales$muT.g[modspID])/
    spp_clim_scales$sdT.g[modspID]
hot26 = (12.3 + 2.9 - spp_clim_scales$muT.g[modspID])/
    spp_clim_scales$sdT.g[modspID]
```

```
polygon(c(cold26, cold26, hot26, hot26),
        c(-1,10,10,-1), col = col.alpha('blue'), border = NA)
abline(v = (12.3 + 1.6 - spp_clim_scales$muT.g[modspID])/
           spp_clim_scales$sdT.g[modspID],
       lty = 3, col = col.alpha('blue', 0.5), lwd = 1)

cold45 = (12.3 + 2 - spp_clim_scales$muT.g[modspID])/
    spp_clim_scales$sdT.g[modspID]
hot45 = (12.3 + 4.2 - spp_clim_scales$muT.g[modspID])/
    spp_clim_scales$sdT.g[modspID]
polygon(c(cold45, cold45, hot45, hot45),
        c(-1,12,12,-1), col = col.alpha('orange'), border = NA)
abline(v = (12.3 + 2.9 - spp_clim_scales$muT.g[modspID])/
           spp_clim_scales$sdT.g[modspID],
       lty = 3, col = col.alpha('orange', 0.5), lwd = 1)

cold85 = (12.3 + 4.1 - spp_clim_scales$muT.g[modspID])/
    spp_clim_scales$sdT.g[modspID]
hot85 = (12.3 + 7.2 - spp_clim_scales$muT.g[modspID])/
    spp_clim_scales$sdT.g[modspID]
polygon(c(cold85, cold85, hot85, hot85),
        c(-1,12,12,-1), col = col.alpha('red'), border = NA)
abline(v = (12.3 + 5.3 - spp_clim_scales$muT.g[modspID])/
           spp_clim_scales$sdT.g[modspID],
       lty = 3, col = col.alpha('red', 0.5), lwd = 1)

abline( h = 1, lty = 1, col = "darkgray")

if(doSpp == spp_ids[1]) {
        mtext("Growth \nrate", side = 2, line = 3, cex = 0.75)
        axis(2, at = c(0,1, 2, 3),
             labels = c("0","1", "2", "3"),
             cex.axis = 1, las = 1)
}

if(doSpp == spp_ids[5]) {
        legend(hot*1.3, 1.2, y.intersp = 1.5, x.intersp = 2,
               legend = c("10", "100", "250", "500", "750", "1,000"),
               pch = 19, col = col.alpha('black', 0.2),
               pt.cex = (sqrt(c(10, 100, 250, 500, 750, 1000))/50)*4,
               horiz = F, xpd = NA,
               bty = "n",
               box.lwd = 0.75,  cex = 0.75)
        legend(hot*1.3, 1.2, y.intersp = 1.5, x.intersp = 2,
               legend = c("", "", "", "", "", ""),
               pch = 1, col = "darkgray",
               pt.cex = (sqrt(c(10, 100, 250, 500, 750, 1000))/50)*4,
               horiz = F, xpd = NA,
               bty = "n",
               box.lwd = 0.75,  cex = 0.75)
        text(hot*1.65, 1.2,"Sample size", font = 2, cex = 0.85, xpd = NA)
```

```r
        legend(hot*1.25, 4.7, y.intersp = 1.5, x.intersp = 1.25,
               legend = c("Min.", "Mean", "Max."),
               lty = c(3,1,2), lwd = 2,
               horiz = F, xpd = NA,
               bty = "n",
               box.lwd = 0.75,  cex = 0.75)

        text(hot*1.75, 5,"Heterospecific \n crowding", font = 2,
             cex = 0.85, xpd = NA)
}

panel_labels = c('(F)', '(G)', '(H)', '(I)', '(J)')
mtext(panel_labels[which(doSpp == spp_ids)],
      3, font = 2, cex = 0.65, adj = 0, line = 0.2)

temp = seq(cold, hot, length.out = 100)


dG$temp_f = factor((dG$tempL2S - mean(dG$tempL2S))/sd(dG$tempL2S))
dG$growth = log(dG$u2/dG$u1)

points(as.numeric(names(tapply(dG$growth, dG$temp_f, mean))),
       exp(as.numeric(tapply(dG$growth, dG$temp_f, mean))),
       pch = 19, cex = (sqrt(table(dG$temp_f))/50 )*4,
       col = col.alpha('black', 0.2))
points(as.numeric(names(tapply(dG$growth, dG$temp_f, mean))),
       exp(as.numeric(tapply(dG$growth, dG$temp_f, mean))),
       cex = (sqrt(table(dG$temp_f))/50 )*4, col = "darkgray")


# Add model mean prediction
# Predictions with median values
w_inter = summary(dG$w.cinter)[c(1,4,6)]
w_intra = mean(dG$w)

u_mu  = mean(dG$u1)

# Add model mean predictions
for (crow in 1:length(w_inter)) {
    xposib = matrix(NA, nrow = 1e3, ncol = length(temp))
    for (ps in 1:1e3) {
        xposib[ps,] = Gpars$b0[ps,doSpp] +
                Gpars$tef[ps,doSpp] *
          exp(-((temp - Gpars$ttp[ps,doSpp])^2/2*Gpars$tsd[ps,doSpp]^2)) +
                log(u_mu) * Gpars$b[ps,doSpp] -
                w_inter[crow] * exp(Gpars$aC_0[ps,doSpp] +
                                    Gpars$b_aC[ps,doSpp] * temp) -
                w_intra * exp(
                    Gpars$a_0[ps,doSpp] + Gpars$b_a[ps,doSpp] * temp)
    }

    x.mu = apply(xposib, 2, mean)
    x.ci = apply(xposib, 2, PI)
```

```
        lty_crow = c(3,1,2)[crow]


        shade(exp(x.ci), temp, col = col.alpha("gray", 0.25))
        lines(temp, exp(x.mu), lwd = 1.25, lty = lty_crow)
    }




}

# Recruitment

for (doSpp in spp_ids) {

    modspID <- which(sppList[doSpp] == sppList)

    dfile = paste('processed-data/', sppList[doSpp], '_RD.csv', sep = '')
    d = read_csv(dfile)
    d = subset(d, x > 20 & x < 80 & y > 20 & y < 80)

    # Exclude ramets in grids with collars
    ## Alpha grids in Calanda plots
    d <- d[!(d$site == "Cal" & d$x < 50 & d$y > 50), ]

    ## Alpha or gamma grids in Nes plots
    collarsG = c('Nes01.03', 'Nes02.03', 'Nes04.03',
                 'Nes05.01', 'Nes08.01', 'Nes09.03')
    collarsA = c('Nes03.01', 'Nes06.03', 'Nes07.03', 'Nes10.01')

    d <- d[!(d$plot %in% collarsA & d$x < 50 & d$y > 50), ]
    d <- d[!(d$plot %in% collarsG & d$x < 50 & d$y < 50), ]

    cold = (12 - spp_clim_scales$muT.r[modspID])/spp_clim_scales$sdT.r[modspID]
    hot = (19 - spp_clim_scales$muT.r[modspID])/spp_clim_scales$sdT.r[modspID]

    plot(NA,
         xlim = c(cold, hot), ylim = c(0,0.2), xlab = "",
         ylab = "", xaxt = "n", yaxt = "n")
    ticks = (c(13,15,17) - spp_clim_scales$muT.r[modspID])/
        spp_clim_scales$sdT.r[modspID]
    axis(1, at = ticks, labels = c(13,15,17), cex.axis = 1)

    abline(v = (12.3 - spp_clim_scales$muT.r[modspID])/
               spp_clim_scales$sdT.r[modspID],
           lty = 3, col = 'darkgray', lwd = 1)

    cold26 = (12.3 + 0.9 - spp_clim_scales$muT.r[modspID])/
        spp_clim_scales$sdT.r[modspID]
    hot26 = (12.3 + 2.9 - spp_clim_scales$muT.r[modspID])/
        spp_clim_scales$sdT.r[modspID]
    polygon(c(cold26, cold26, hot26, hot26),
```

```
        c(-1,2,2,-1), col = col.alpha('blue'), border = NA)
abline(v = (12.3 + 1.6 - spp_clim_scales$muT.r[modspID])/
           spp_clim_scales$sdT.r[modspID],
       lty = 3, col = col.alpha('blue', 0.5), lwd = 1)

cold45 = (12.3 + 2 - spp_clim_scales$muT.r[modspID])/
    spp_clim_scales$sdT.r[modspID]
hot45 = (12.3 + 4.2 - spp_clim_scales$muT.r[modspID])/
    spp_clim_scales$sdT.r[modspID]
polygon(c(cold45, cold45, hot45, hot45),
        c(-1,2,2,-1), col = col.alpha('orange'), border = NA)
abline(v = (12.3 + 2.9 - spp_clim_scales$muT.r[modspID])/
           spp_clim_scales$sdT.r[modspID],
       lty = 3, col = col.alpha('orange', 0.5), lwd = 1)

cold85 = (12.3 + 4.1 - spp_clim_scales$muT.r[modspID])/
    spp_clim_scales$sdT.r[modspID]
hot85 = (12.3 + 7.2 - spp_clim_scales$muT.r[modspID])/
    spp_clim_scales$sdT.r[modspID]
polygon(c(cold85, cold85, hot85, hot85),
        c(-1,2,2,-1), col = col.alpha('red'), border = NA)
abline(v = (12.3 + 5.3 - spp_clim_scales$muT.r[modspID])/
           spp_clim_scales$sdT.r[modspID],
       lty = 3, col = col.alpha('red', 0.5), lwd = 1)



mtext("Summer \ntemperature (ºC)", side = 1, line = 3.7, cex = 0.75)


if(doSpp == spp_ids[1]) {
        mtext("Recruitment \nprobability", side = 2, line = 3, cex = 0.75)
        axis(2, at = c(0,0.2, 0.4, 0.6),
             labels = c("0.0","0.2", "0.4", "0.6"),
             cex.axis = 1, las = 1)
}

panel_labels = c('(K)', '(L)', '(M)', '(N)', '(O)')
mtext(panel_labels[which(doSpp == spp_ids)],
      3, font = 2, cex = 0.65, adj = 0, line = 0.2)

d$temp_f = factor((d$tempLS - mean(d$tempLS))/sd(d$tempLS))

points(as.numeric(names(tapply(d$recruitment, d$temp_f, mean))),
       as.numeric(tapply(d$recruitment, d$temp_f, mean)),
       pch = 19, cex = (sqrt(table(d$temp_f))/50 )*4,
       col = col.alpha('black',0.2))
points(as.numeric(names(tapply(d$recruitment, d$temp_f, mean))),
       as.numeric(tapply(d$recruitment, d$temp_f, mean)),
       cex = (sqrt(table(d$temp_f))/50 )*4, col = "darkgray")


# Add model predictions
```

```
            temp = seq(cold, hot, length.out = 100)
            w_inter = summary(d$w.cinter)[c(1,4,6)]
            w_intra = quantile(d$w, 0.95)


            for (crow in 1:length(w_inter)) {
                xposib = matrix(NA, nrow = 1e3, ncol = length(temp))
                for (ps in 1:1e3) {
                    xposib[ps,] = exp(Rpars$a_0[ps,doSpp] + Rpars$b_a[ps,doSpp] * temp )*
                        w_intra -
                            exp(Rpars$aC_0[ps,doSpp]  + Rpars$b_aC[ps,doSpp]  * temp )*
                        w_inter[crow] -10
                }

                x.mu = apply(xposib, 2, mean)
                x.ci = apply(xposib, 2, PI)

                lty_crow <- c(3,1,2)[crow]


                shade(exp(x.ci)/(1+exp(x.ci)), temp, col = col.alpha('gray', 0.5))
                lines(temp, exp(x.mu)/(1+exp(x.mu)), lwd = 1.25, lty = lty_crow)
            }


        }

}
```

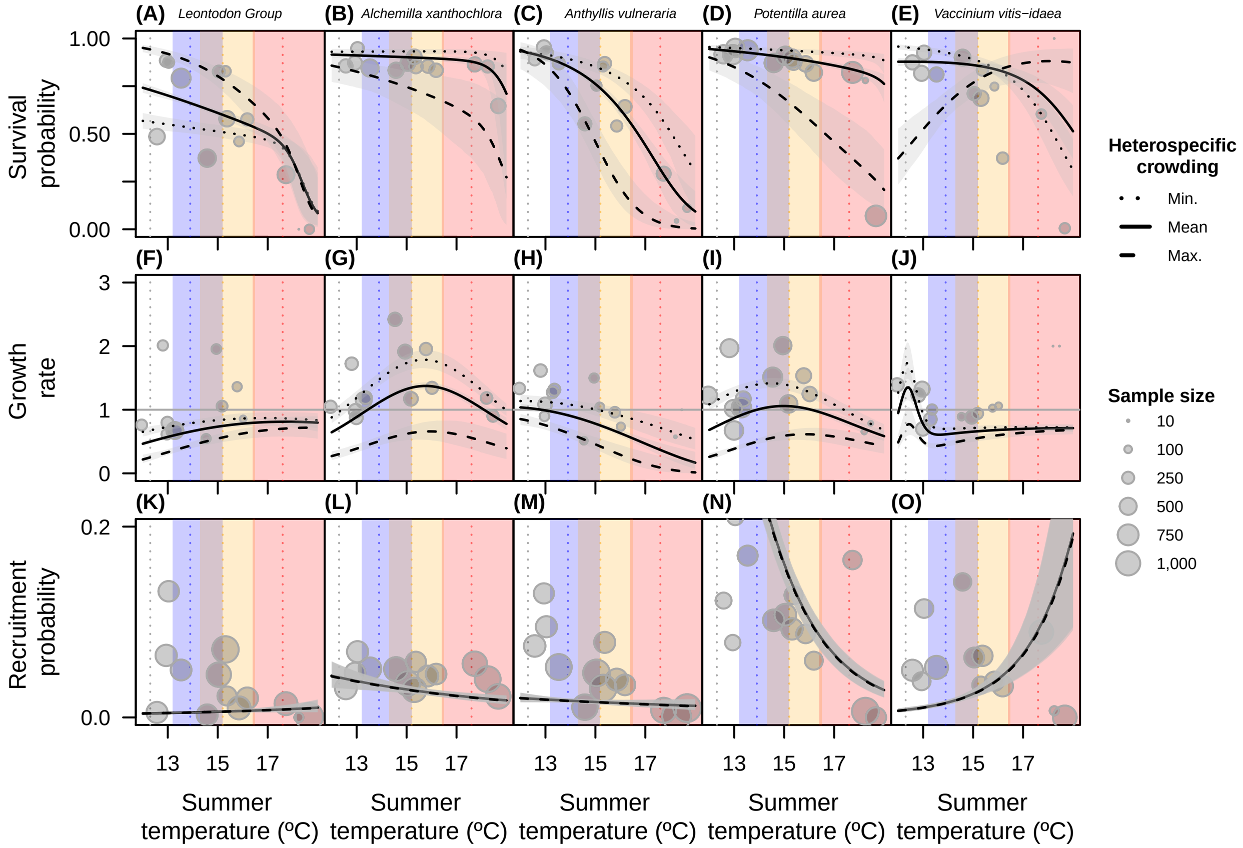

(A) Leontodon Group
(B) Alchemilla xanthochlora
(C) Anthyllis vulneraria
(D) Potentilla aurea
(E) Vaccinium vitis−idaea
Survival probability
Growth rate
Recruitment probability
Heterospecific crowding
Min.
Mean
Max.
Sample size
10
100
250
500
750
1,000
Summer temperature (ºC)

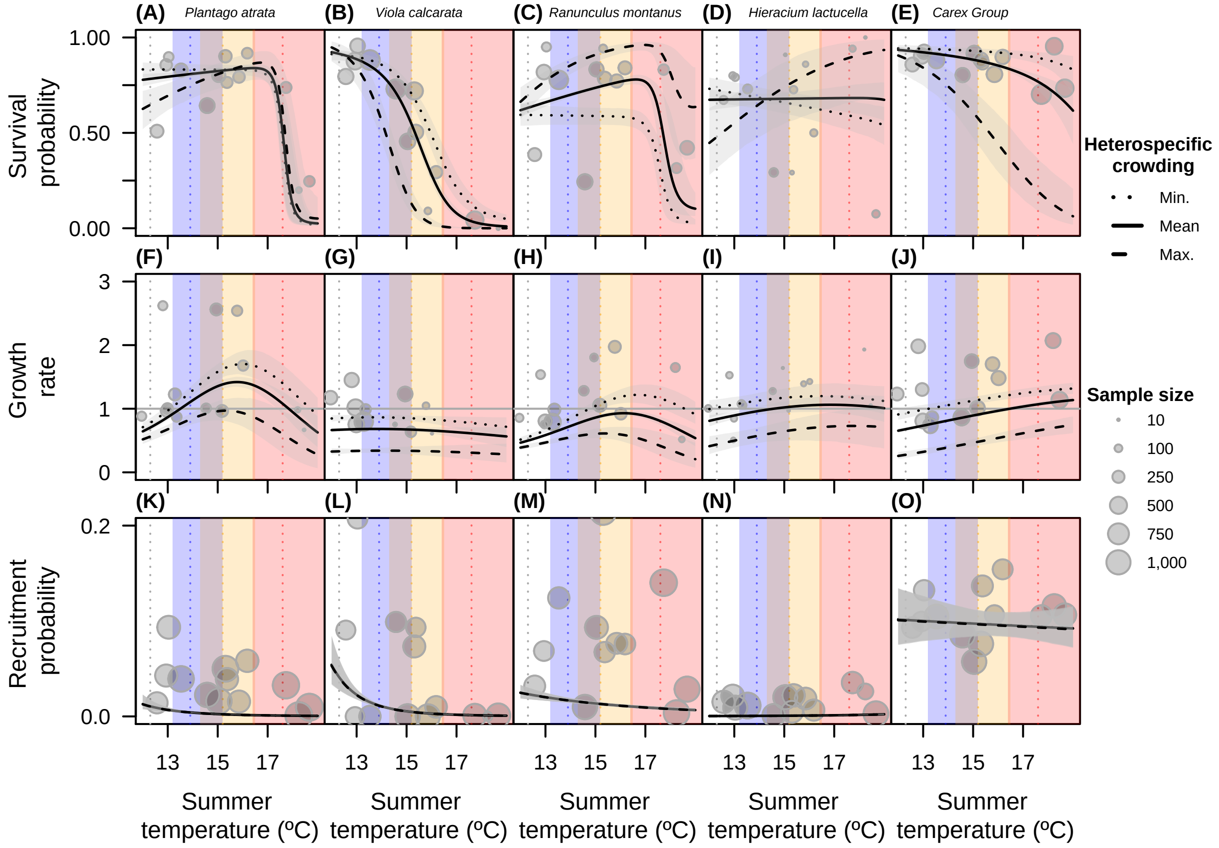

(A) Plantago atrata
(B) Viola calcarata
(C) Ranunculus montanus
(D) Hieracium lactucella
(E) Carex Group
Survival probability
Growth rate
Recruitment probability
Heterospecific crowding
Min.
Mean
Max.
Sample size
10
100
250
500
750
1,000
Summer temperature (ºC)

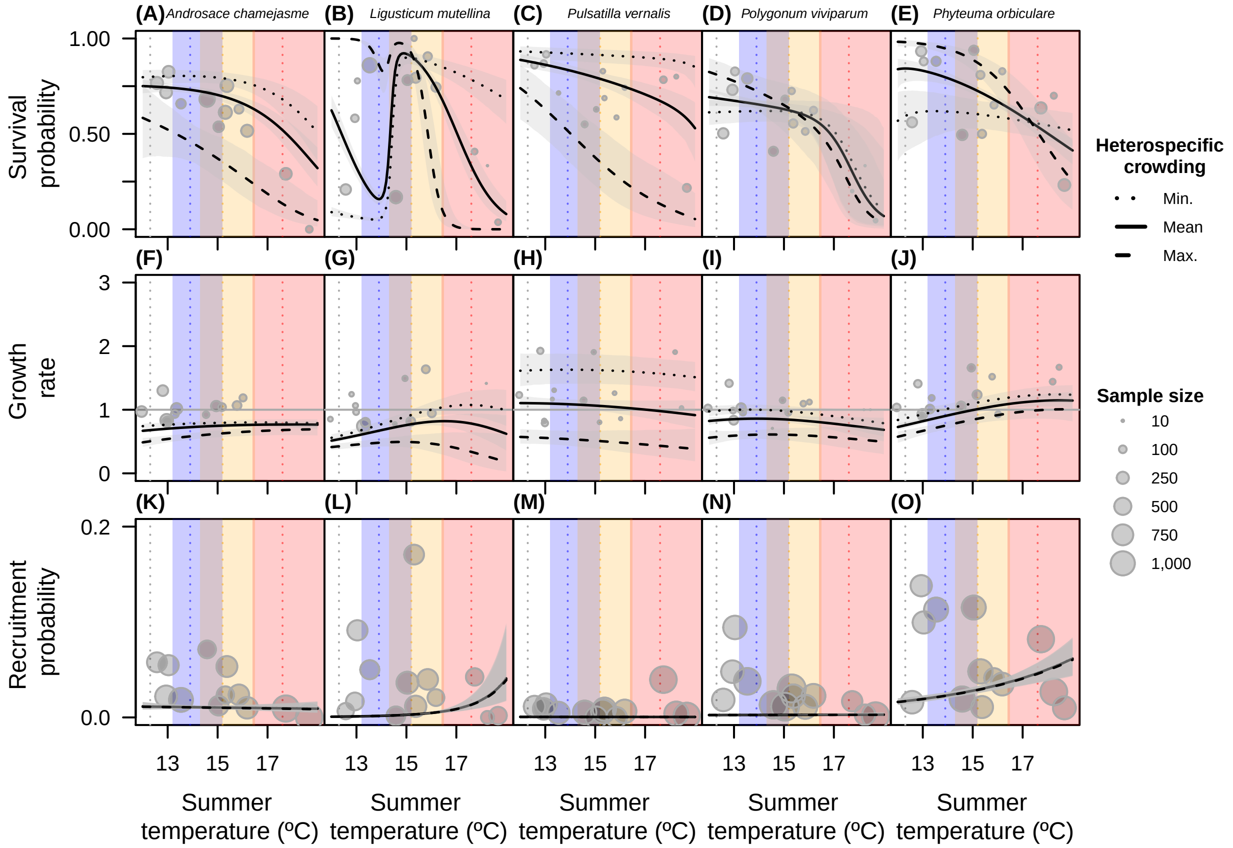
(A) Androsace chamejasme
(B) Ligusticum mutellina
(C) Pulsatilla vernalis
(D) Polygonum viviparum
(E) Phyteuma orbiculare
Survival probability
Growth rate
Recruitment probability
Heterospecific crowding
Min.
Mean
Max.
Sample size
10
100
250
500
750
1,000
Summer temperature (ºC)

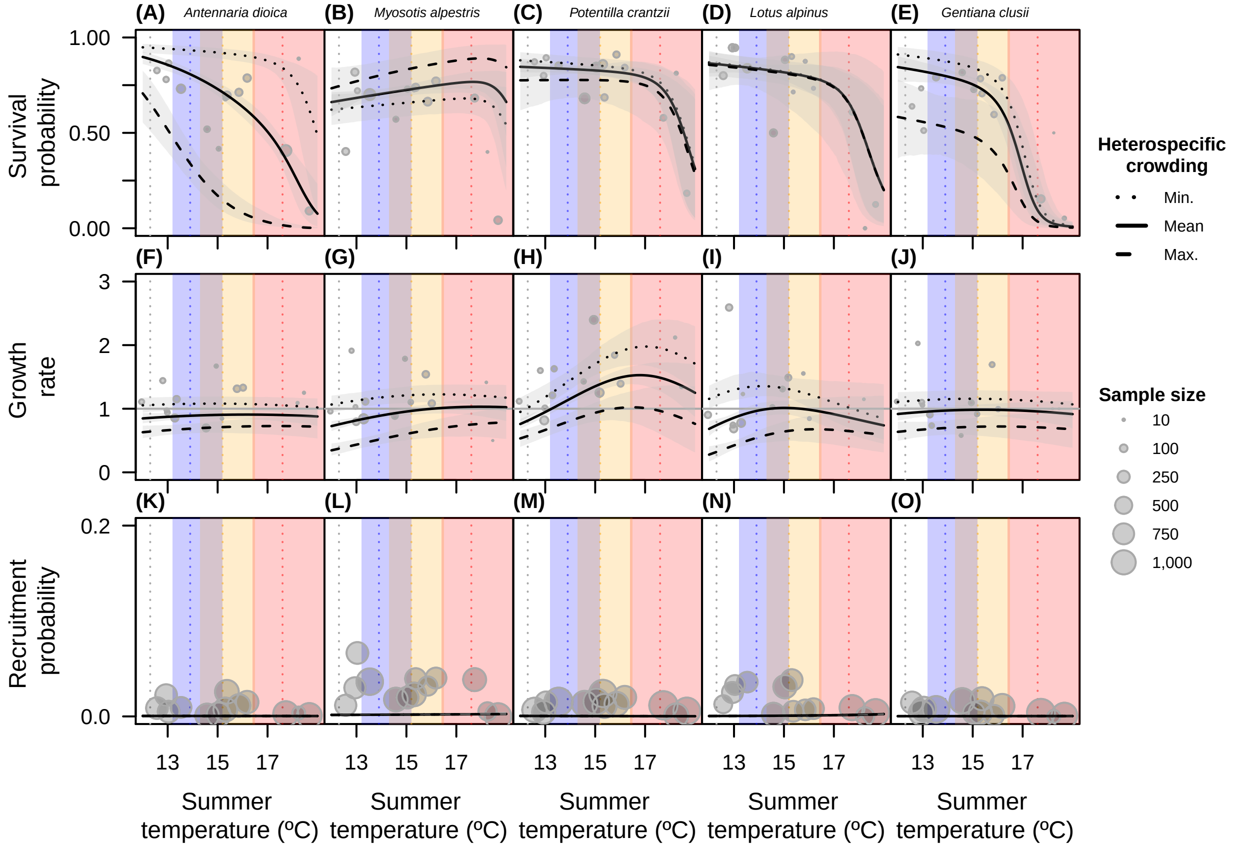
(A) Antennaria dioica
(B) Myosotis alpestris
(C) Potentilla crantzii
(D) Lotus alpinus
(E) Gentiana clusii
Survival probability
Growth rate
Recruitment probability
Heterospecific crowding
Min.
Mean
Max.
Sample size
10
100
250
500
750
1,000
Summer temperature (ºC)

```r
spp_ids = 21:24
par(oma = c(4.2,5.5,1,6.5), mar=c(1,0,0.5,0), mfrow = c(3,4), cex.axis = 1.5)

## Survival
for (doSpp in spp_ids) {

    modspID <- which(sppList[doSpp] == sppList)

    dfile = paste('processed-data/', sppList[doSpp], '_DMD.csv', sep = '')
    d = read_csv(dfile)
    d = subset(d, x > 20 & x < 80 & y > 20 & y < 80)

    # Exclude ramets in grids with collars
    ## Alpha grids in Calanda plots
    d <- d[!(d$site == "Cal" & d$x < 50 & d$y > 50), ]

    ## Alpha or gamma grids in Nes plots
    collarsG = c('Nes01.03', 'Nes02.03', 'Nes04.03',
                 'Nes05.01', 'Nes08.01', 'Nes09.03')
    collarsA = c('Nes03.01', 'Nes06.03', 'Nes07.03', 'Nes10.01')

    d <- d[!(d$plot %in% collarsA & d$x < 50 & d$y > 50), ]
    d <- d[!(d$plot %in% collarsG & d$x < 50 & d$y < 50), ]


    # Exclude ramets that affected by disturbance

    for (i in 1:nrow(disturbances)) {

            if (disturbances$level[i] == 'quadrant') {

                    quadid = as.character(disturbances$quadrant_id[i])
                    plotid = substr(quadid, 1, 8)
                    gridid = substr(quadid, 10, 11)
                    rowid = substr(quadid, 13, 13)
                    colid = substr(quadid, 14, 14)
                    qid = as.numeric(substr(quadid, 16, 16))

                    # get quad coordinates
                    xi = ifelse(gridid %in% c('BE', 'DE'), 50, 0)
                    yi = ifelse(gridid %in% c('BE', 'AL'), 50, 0)

                    xi = xi + as.numeric(colid)*10 - 5
                    yi = yi + ifelse(rowid == "A", 45,
                                     ifelse(rowid == "B", 35,
                                            ifelse(rowid == "C", 25,
                                                   ifelse(rowid == "d", 15, 5))))

                    xi = xi + ifelse(qid %in% c(1,3), -2.5, 2.5)
                    yi = yi + ifelse(qid %in% c(3,4), -2.5, 2.5)

                    rmi = which(d$plot == plotid &
                                abs(d$x - xi) < 3 &
```

```
                                   abs(d$y - yi) < 3 &
                                   d$survival == 0 &
                                   d$year + 1 == disturbances$year[i])

                if (length(rmi)>0) d = d[-rmi, ]

                rm(rmi, xi, yi, quadid, plotid, gridid, rowid, colid, qid)

        }


        if (disturbances$level[i] == 'cell') {

                cellid = as.character(disturbances$cell_id[i])
                plotid = substr(cellid, 1, 8)
                gridid = substr(cellid, 10, 11)
                rowid = substr(cellid, 13, 13)
                colid = substr(cellid, 14, 14)

                # get cell coordinates
                xi = ifelse(gridid %in% c('BE', 'DE'), 50, 0)
                yi = ifelse(gridid %in% c('BE', 'AL'), 50, 0)

                xi = xi + as.numeric(colid)*10 - 5
                yi = yi + ifelse(rowid == "A", 45,
                                 ifelse(rowid == "B", 35,
                                        ifelse(rowid == "C", 25,
                                               ifelse(rowid == "d", 15, 5))))

                rmi = which(d$plot == plotid & abs(d$x - xi) < 5 & abs(d$y - yi) < 5 &
                             d$survival == 0 &
                             d$year + 1 == disturbances$year[i])

                if (length(rmi)>0) d = d[-rmi, ]

                rm(rmi, xi, yi, cellid, plotid, gridid, rowid, colid)
        }

}


cold = (12 - spp_clim_scales$muT.s[modspID])/spp_clim_scales$sdT.s[modspID]
hot = (19 - spp_clim_scales$muT.s[modspID])/spp_clim_scales$sdT.s[modspID]

wet = 0

## Survival
plot(NA, type = "n",
     xlim = c(cold, hot), ylim = c(0,1), xlab = "",
     ylab = "", xaxt = "n", yaxt = "n")

ticks = (c(13,15,17) - spp_clim_scales$muT.s[modspID])/
    spp_clim_scales$sdT.s[modspID]
```

```r
axis(1, at = ticks, labels = F)

abline(v = (12.3 - spp_clim_scales$muT.s[modspID])/
            spp_clim_scales$sdT.s[modspID],
       lty = 3, col = 'darkgray', lwd = 1)

cold26 = (12.3 + 0.9 - spp_clim_scales$muT.s[modspID])/
    spp_clim_scales$sdT.s[modspID]
hot26 = (12.3 + 2.9 - spp_clim_scales$muT.s[modspID])/
    spp_clim_scales$sdT.s[modspID]
polygon(c(cold26, cold26, hot26, hot26),
        c(-1,2,2,-1), col = col.alpha('blue'), border = NA)
abline(v = (12.3 + 1.6 - spp_clim_scales$muT.s[modspID])/
            spp_clim_scales$sdT.s[modspID],
       lty = 3, col = col.alpha('blue', 0.5), lwd = 1)

cold45 = (12.3 + 2 - spp_clim_scales$muT.s[modspID])/
    spp_clim_scales$sdT.s[modspID]
hot45 = (12.3 + 4.2 - spp_clim_scales$muT.s[modspID])/
    spp_clim_scales$sdT.s[modspID]
polygon(c(cold45, cold45, hot45, hot45),
        c(-1,2,2,-1), col = col.alpha('orange'), border = NA)
abline(v = (12.3 + 2.9 - spp_clim_scales$muT.s[modspID])/
            spp_clim_scales$sdT.s[modspID],
       lty = 3, col = col.alpha('orange', 0.5), lwd = 1)

cold85 = (12.3 + 4.1 - spp_clim_scales$muT.s[modspID])/
    spp_clim_scales$sdT.s[modspID]
hot85 = (12.3 + 7.2 - spp_clim_scales$muT.s[modspID])/
    spp_clim_scales$sdT.s[modspID]
polygon(c(cold85, cold85, hot85, hot85),
        c(-1,2,2,-1), col = col.alpha('red'), border = NA)
abline(v = (12.3 + 5.3 - spp_clim_scales$muT.s[modspID])/
            spp_clim_scales$sdT.s[modspID],
       lty = 3, col = col.alpha('red', 0.5), lwd = 1)


if(doSpp == spp_ids[1]) {
        mtext("Survival \nprobability", side = 2, line = 3, cex = 0.75)
        axis(2, at = c(0,0.25, 0.5, 0.75, 1),
             labels = c("0.00","", "0.50", "", "1.00"),
             cex.axis = 1, las = 1)

}


mtext(domsp[modspID], 3, font = 3, cex = 0.4, line = 0.3,
      adj = nchar(domsp[modspID])/30)

panel_labels = c('(A)', '(B)', '(C)', '(D)', '(E)')
mtext(panel_labels[which(doSpp == spp_ids)],
      3, font = 2, cex = 0.65, adj = 0, line = 0.2)
```

```
    d$temp_f = factor((d$tempLS - mean(d$tempLS))/sd(d$tempLS))

    points(as.numeric(names(tapply(d$survival, d$temp_f, mean))),
           as.numeric(tapply(d$survival, d$temp_f, mean)),
           pch = 19, cex = (sqrt(table(d$temp_f))/50)*4,
           col = col.alpha('black', 0.2))
    points(as.numeric(names(tapply(d$survival, d$temp_f, mean))),
           as.numeric(tapply(d$survival, d$temp_f, mean)),
           cex = (sqrt(table(d$temp_f))/50 )*4, col = "darkgray")



    ## Add model predictions

    w_intra = mean(d$w)
    w_inter = summary(d$w.cinter)[c(1,4,6)]

    u_mu = 0
    temp = seq(cold, hot, length.out = 1e2)
    moist = wet

    for (crow in 1:length(w_inter)) {

        xposib = matrix(NA, nrow = 1e3, ncol = length(temp))
        for (ps in 1:1e3) {
            ttp = Spars$ttp_a[ps,doSpp] + Spars$ttp_b[ps,doSpp] * moist
            xposib[ps,] = Spars$tef[ps,doSpp] / (1 + exp(-Spars$k[ps,doSpp] *
                                                     (temp - ttp))) +
                    Spars$b[ps,doSpp] *  u_mu +
                    (Spars$a_0[ps,doSpp] + Spars$b_a[ps,doSpp] * temp)*w_intra +
                    (Spars$aC_0[ps,doSpp]  + Spars$b_aC[ps,doSpp] * temp)*
                w_inter[crow] - 4


        }
        x.mu = apply(xposib, 2, mean)
        x.ci = apply(xposib, 2, PI)

        lty_crow = c(3,1,2)[crow]

        shade(exp(x.ci)/(1+exp(x.ci)), temp, col = col.alpha("gray", 0.25))
        lines(temp, exp(x.mu)/(1+exp(x.mu)),
              lwd = 1.25, lty = lty_crow)


    }

}

## Growth

for (doSpp in spp_ids) {
```

```
modspID <- which(sppList[doSpp] == sppList)

dfile = paste('processed-data/', sppList[doSpp], '_DMD.csv', sep = '')
d = read_csv(dfile)
d = subset(d, x > 20 & x < 80 & y > 20 & y < 80)

# Exclude ramets in grids with collars
## Alpha grids in Calanda plots
d <- d[!(d$site == "Cal" & d$x < 50 & d$y > 50), ]

## Alpha or gamma grids in Nes plots
collarsG = c('Nes01.03', 'Nes02.03', 'Nes04.03',
             'Nes05.01', 'Nes08.01', 'Nes09.03')
collarsA = c('Nes03.01', 'Nes06.03', 'Nes07.03', 'Nes10.01')

d <- d[!(d$plot %in% collarsA & d$x < 50 & d$y > 50), ]
d <- d[!(d$plot %in% collarsG & d$x < 50 & d$y < 50), ]


# Exclude ramets that affected by disturbance
for (i in 1:nrow(disturbances)) {

        if (disturbances$level[i] == 'quadrant') {

                quadid = as.character(disturbances$quadrant_id[i])
                plotid = substr(quadid, 1, 8)
                gridid = substr(quadid, 10, 11)
                rowid = substr(quadid, 13, 13)
                colid = substr(quadid, 14, 14)
                qid = as.numeric(substr(quadid, 16, 16))

                # get quad coordinates
                xi = ifelse(gridid %in% c('BE', 'DE'), 50, 0)
                yi = ifelse(gridid %in% c('BE', 'AL'), 50, 0)

                xi = xi + as.numeric(colid)*10 - 5
                yi = yi + ifelse(rowid == "A", 45,
                                 ifelse(rowid == "B", 35,
                                        ifelse(rowid == "C", 25,
                                               ifelse(rowid == "D", 15, 5))))

                xi = xi + ifelse(qid %in% c(1,3), -2.5, 2.5)
                yi = yi + ifelse(qid %in% c(3,4), -2.5, 2.5)

                rmi = which(d$plot == plotid & abs(d$x - xi) < 3 &
                              abs(d$y - yi) < 3 & d$survival == 0 &
                                d$year + 1 == disturbances$year[i])

                if (length(rmi)>0) d = d[-rmi, ]

                rm(rmi, xi, yi, quadid, plotid, gridid, rowid, colid, qid)

        }
```

```
        if (disturbances$level[i] == 'cell') {

                cellid = as.character(disturbances$cell_id[i])
                plotid = substr(cellid, 1, 8)
                gridid = substr(cellid, 10, 11)
                rowid = substr(cellid, 13, 13)
                colid = substr(cellid, 14, 14)

                # get cell coordinates
                xi = ifelse(gridid %in% c('BE', 'DE'), 50, 0)
                yi = ifelse(gridid %in% c('BE', 'AL'), 50, 0)

                xi = xi + as.numeric(colid)*10 - 5
                yi = yi + ifelse(rowid == "A", 45,
                                 ifelse(rowid == "B", 35,
                                        ifelse(rowid == "C", 25,
                                               ifelse(rowid == "D", 15, 5))))

                rmi = which(d$plot == plotid &
                              abs(d$x - xi) < 5 &
                              abs(d$y - yi) < 5 &
                              d$survival == 0 &
                              d$year + 1 == disturbances$year[i])

                if (length(rmi)>0) d = d[-rmi, ]

                rm(rmi, xi, yi, cellid, plotid, gridid, rowid, colid)
        }

}

# For now, let's use only survivors
dG <- d[complete.cases(d$u2),]

# Fix erroneous cover values
if(sppList[doSpp] != "FesGro"){
        dG$u1[dG$u1 < 1.5625] <- 1.5625
        dG$u2[dG$u2 < 1.5625] <- 1.5625
}


dG$u1[dG$u1 < 0.390625] <- 0.390625
dG$u1[dG$u1 > 0.390625 & dG$u1 < 1.5625] <- 1.5625
dG$u1[dG$u1 > 1.5625 & dG$u1 < 3.125] <- 3.125
dG$u1[dG$u1 > 3.125 & dG$u1 < 6.25] <- 6.25
dG$u1[dG$u1 > 6.25 & dG$u1 < 12.5] <- 12.5

dG$u2[dG$u2 < 0.390625] <- 0.390625


cold = (12 - spp_clim_scales$muT.g[modspID])/spp_clim_scales$sdT.g[modspID]
hot = (19 - spp_clim_scales$muT.g[modspID])/spp_clim_scales$sdT.g[modspID]
```

```
plot(NA,
     xlim = c(cold, hot), ylim = c(0,3), xlab = "",
     ylab = "Growth", xaxt = "n", yaxt = "n")
ticks = (c(13,15,17) - spp_clim_scales$muT.g[modspID])/
    spp_clim_scales$sdT.g[modspID]
axis(1, at = ticks, labels = F)

abline(v = (12.3 - spp_clim_scales$muT.g[modspID])/
           spp_clim_scales$sdT.g[modspID],
       lty = 3, col = 'darkgray', lwd = 1)

cold26 = (12.3 + 0.9 - spp_clim_scales$muT.g[modspID])/
    spp_clim_scales$sdT.g[modspID]
hot26 = (12.3 + 2.9 - spp_clim_scales$muT.g[modspID])/
    spp_clim_scales$sdT.g[modspID]
polygon(c(cold26, cold26, hot26, hot26),
        c(-1,10,10,-1), col = col.alpha('blue'), border = NA)
abline(v = (12.3 + 1.6 - spp_clim_scales$muT.g[modspID])/
           spp_clim_scales$sdT.g[modspID],
       lty = 3, col = col.alpha('blue', 0.5), lwd = 1)

cold45 = (12.3 + 2 - spp_clim_scales$muT.g[modspID])/
    spp_clim_scales$sdT.g[modspID]
hot45 = (12.3 + 4.2 - spp_clim_scales$muT.g[modspID])/
    spp_clim_scales$sdT.g[modspID]
polygon(c(cold45, cold45, hot45, hot45),
        c(-1,12,12,-1), col = col.alpha('orange'), border = NA)
abline(v = (12.3 + 2.9 - spp_clim_scales$muT.g[modspID])/
           spp_clim_scales$sdT.g[modspID],
       lty = 3, col = col.alpha('orange', 0.5), lwd = 1)

cold85 = (12.3 + 4.1 - spp_clim_scales$muT.g[modspID])/
    spp_clim_scales$sdT.g[modspID]
hot85 = (12.3 + 7.2 - spp_clim_scales$muT.g[modspID])/
    spp_clim_scales$sdT.g[modspID]
polygon(c(cold85, cold85, hot85, hot85),
        c(-1,12,12,-1), col = col.alpha('red'), border = NA)
abline(v = (12.3 + 5.3 - spp_clim_scales$muT.g[modspID])/
           spp_clim_scales$sdT.g[modspID],
       lty = 3, col = col.alpha('red', 0.5), lwd = 1)

abline( h = 1, lty = 1, col = "darkgray")

if(doSpp == spp_ids[1]) {
        mtext("Growth \nrate", side = 2, line = 3, cex = 0.75)
        axis(2, at = c(0,1, 2, 3),
             labels = c("0","1", "2", "3"),
             cex.axis = 1, las = 1)
}

if(doSpp == spp_ids[4]) {
        legend(hot*1.3, 1.2, y.intersp = 1.5, x.intersp = 2,
               legend = c("10", "100", "250", "500", "750", "1,000"),
```

```
                pch = 19, col = col.alpha('black', 0.2),
                pt.cex = (sqrt(c(10, 100, 250, 500, 750, 1000))/50)*4,
                horiz = F, xpd = NA,
                bty = "n",
                box.lwd = 0.75,  cex = 0.75)
         legend(hot*1.3, 1.2, y.intersp = 1.5, x.intersp = 2,
                legend = c("", "", "", "", "", ""),
                pch = 1, col = "darkgray",
                pt.cex = (sqrt(c(10, 100, 250, 500, 750, 1000))/50)*4,
                horiz = F, xpd = NA,
                bty = "n",
                box.lwd = 0.75,  cex = 0.75)
         text(hot*1.65, 1.2,"Sample size", font = 2, cex = 0.85, xpd = NA)


         legend(hot*1.25, 4.7, y.intersp = 1.5, x.intersp = 1.25,
                legend = c("Min.", "Mean", "Max."),
                lty = c(3,1,2), lwd = 2,
                horiz = F, xpd = NA,
                bty = "n",
                box.lwd = 0.75,  cex = 0.75)

         text(hot*1.75, 5,"Heterospecific \n crowding", font = 2,
              cex = 0.85, xpd = NA)
}

panel_labels = c('(F)', '(G)', '(H)', '(I)', '(J)')
mtext(panel_labels[which(doSpp == spp_ids)],
      3, font = 2, cex = 0.65, adj = 0, line = 0.2)

temp = seq(cold, hot, length.out = 100)


dG$temp_f = factor((dG$tempL2S - mean(dG$tempL2S))/sd(dG$tempL2S))
dG$growth = log(dG$u2/dG$u1)

points(as.numeric(names(tapply(dG$growth, dG$temp_f, mean))),
       exp(as.numeric(tapply(dG$growth, dG$temp_f, mean))),
       pch = 19, cex = (sqrt(table(dG$temp_f))/50 )*4,
       col = col.alpha('black', 0.2))
points(as.numeric(names(tapply(dG$growth, dG$temp_f, mean))),
       exp(as.numeric(tapply(dG$growth, dG$temp_f, mean))),
       cex = (sqrt(table(dG$temp_f))/50 )*4, col = "darkgray")


# Add model mean prediction
# Predictions with median values
w_inter = summary(dG$w.cinter)[c(1,4,6)]
w_intra = mean(dG$w)

u_mu  = mean(dG$u1)

# Add model mean predictions
```

```r
        for (crow in 1:length(w_inter)) {
            xposib = matrix(NA, nrow = 1e3, ncol = length(temp))
            for (ps in 1:1e3) {
                xposib[ps,] = Gpars$b0[ps,doSpp] +
                        Gpars$tef[ps,doSpp] *
                  exp(-((temp - Gpars$ttp[ps,doSpp])^2/2*Gpars$tsd[ps,doSpp]^2)) +
                        log(u_mu) * Gpars$b[ps,doSpp] -
                        w_inter[crow] * exp(Gpars$aC_0[ps,doSpp] +
                                            Gpars$b_aC[ps,doSpp] * temp) -
                        w_intra * exp(Gpars$a_0[ps,doSpp] + Gpars$b_a[ps,doSpp] * temp)
            }

            x.mu = apply(xposib, 2, mean)
            x.ci = apply(xposib, 2, PI)

            lty_crow = c(3,1,2)[crow]


            shade(exp(x.ci), temp, col = col.alpha("gray", 0.25))
            lines(temp, exp(x.mu), lwd = 1.25, lty = lty_crow)
        }

}

# Recruitment

for (doSpp in spp_ids) {

        modspID <- which(sppList[doSpp] == sppList)

        dfile = paste('processed-data/', sppList[doSpp], '_RD.csv', sep = '')
        d = read_csv(dfile)
        d = subset(d, x > 20 & x < 80 & y > 20 & y < 80)

        # Exclude ramets in grids with collars
        ## Alpha grids in Calanda plots
        d <- d[!(d$site == "Cal" & d$x < 50 & d$y > 50), ]

        ## Alpha or gamma grids in Nes plots
        collarsG = c('Nes01.03', 'Nes02.03', 'Nes04.03',
                     'Nes05.01', 'Nes08.01', 'Nes09.03')
        collarsA = c('Nes03.01', 'Nes06.03', 'Nes07.03', 'Nes10.01')

        d <- d[!(d$plot %in% collarsA & d$x < 50 & d$y > 50), ]
        d <- d[!(d$plot %in% collarsG & d$x < 50 & d$y < 50), ]

        cold = (12 - spp_clim_scales$muT.r[modspID])/spp_clim_scales$sdT.r[modspID]
        hot = (19 - spp_clim_scales$muT.r[modspID])/spp_clim_scales$sdT.r[modspID]

        plot(NA,
             xlim = c(cold, hot), ylim = c(0,0.2), xlab = "",
             ylab = "", xaxt = "n", yaxt = "n")
        ticks = (c(13,15,17) - spp_clim_scales$muT.r[modspID])/
```

```
    spp_clim_scales$sdT.r[modspID]
axis(1, at = ticks, labels = c(13,15,17), cex.axis = 1)

abline(v = (12.3 - spp_clim_scales$muT.r[modspID])/
           spp_clim_scales$sdT.r[modspID],
       lty = 3, col = 'darkgray', lwd = 1)

cold26 = (12.3 + 0.9 - spp_clim_scales$muT.r[modspID])/
    spp_clim_scales$sdT.r[modspID]
hot26 = (12.3 + 2.9 - spp_clim_scales$muT.r[modspID])/
    spp_clim_scales$sdT.r[modspID]
polygon(c(cold26, cold26, hot26, hot26),
        c(-1,2,2,-1), col = col.alpha('blue'), border = NA)
abline(v = (12.3 + 1.6 - spp_clim_scales$muT.r[modspID])/
           spp_clim_scales$sdT.r[modspID],
       lty = 3, col = col.alpha('blue', 0.5), lwd = 1)

cold45 = (12.3 + 2 - spp_clim_scales$muT.r[modspID])/
    spp_clim_scales$sdT.r[modspID]
hot45 = (12.3 + 4.2 - spp_clim_scales$muT.r[modspID])/
    spp_clim_scales$sdT.r[modspID]
polygon(c(cold45, cold45, hot45, hot45),
        c(-1,2,2,-1), col = col.alpha('orange'), border = NA)
abline(v = (12.3 + 2.9 - spp_clim_scales$muT.r[modspID])/
           spp_clim_scales$sdT.r[modspID],
       lty = 3, col = col.alpha('orange', 0.5), lwd = 1)

cold85 = (12.3 + 4.1 - spp_clim_scales$muT.r[modspID])/
    spp_clim_scales$sdT.r[modspID]
hot85 = (12.3 + 7.2 - spp_clim_scales$muT.r[modspID])/
    spp_clim_scales$sdT.r[modspID]
polygon(c(cold85, cold85, hot85, hot85),
        c(-1,2,2,-1), col = col.alpha('red'), border = NA)
abline(v = (12.3 + 5.3 - spp_clim_scales$muT.r[modspID])/
           spp_clim_scales$sdT.r[modspID],
       lty = 3, col = col.alpha('red', 0.5), lwd = 1)



mtext("Summer \ntemperature (ºC)", side = 1, line = 3.7, cex = 0.75)


if(doSpp == spp_ids[1]) {
        mtext("Recruitment \nprobability", side = 2, line = 3, cex = 0.75)
        axis(2, at = c(0,0.2, 0.4, 0.6),
             labels = c("0.0","0.2", "0.4", "0.6"),
             cex.axis = 1, las = 1)
}

panel_labels = c('(K)', '(L)', '(M)', '(N)', '(O)')
mtext(panel_labels[which(doSpp == spp_ids)],
      3, font = 2, cex = 0.65, adj = 0, line = 0.2)
```

```
        d$temp_f = factor((d$tempLS - mean(d$tempLS))/sd(d$tempLS))

        points(as.numeric(names(tapply(d$recruitment, d$temp_f, mean))),
               as.numeric(tapply(d$recruitment, d$temp_f, mean)),
               pch = 19, cex = (sqrt(table(d$temp_f))/50 )*4,
               col = col.alpha('black',0.2))
        points(as.numeric(names(tapply(d$recruitment, d$temp_f, mean))),
               as.numeric(tapply(d$recruitment, d$temp_f, mean)),
               cex = (sqrt(table(d$temp_f))/50 )*4, col = "darkgray")


        # Add model predictions
        temp = seq(cold, hot, length.out = 100)
        w_inter = summary(d$w.cinter)[c(1,4,6)]
        w_intra = quantile(d$w, 0.95)


        for (crow in 1:length(w_inter)) {
            xposib = matrix(NA, nrow = 1e3, ncol = length(temp))
            for (ps in 1:1e3) {
                xposib[ps,] = exp(Rpars$a_0[ps,doSpp] + Rpars$b_a[ps,doSpp] * temp )*
                    w_intra -
                        exp(Rpars$aC_0[ps,doSpp]  + Rpars$b_aC[ps,doSpp]  * temp )*
                    w_inter[crow] -10
            }

            x.mu = apply(xposib, 2, mean)
            x.ci = apply(xposib, 2, PI)

            lty_crow <- c(3,1,2)[crow]


            shade(exp(x.ci)/(1+exp(x.ci)), temp, col = col.alpha('gray', 0.5))
            lines(temp, exp(x.mu)/(1+exp(x.mu)), lwd = 1.25, lty = lty_crow)
        }


}
```

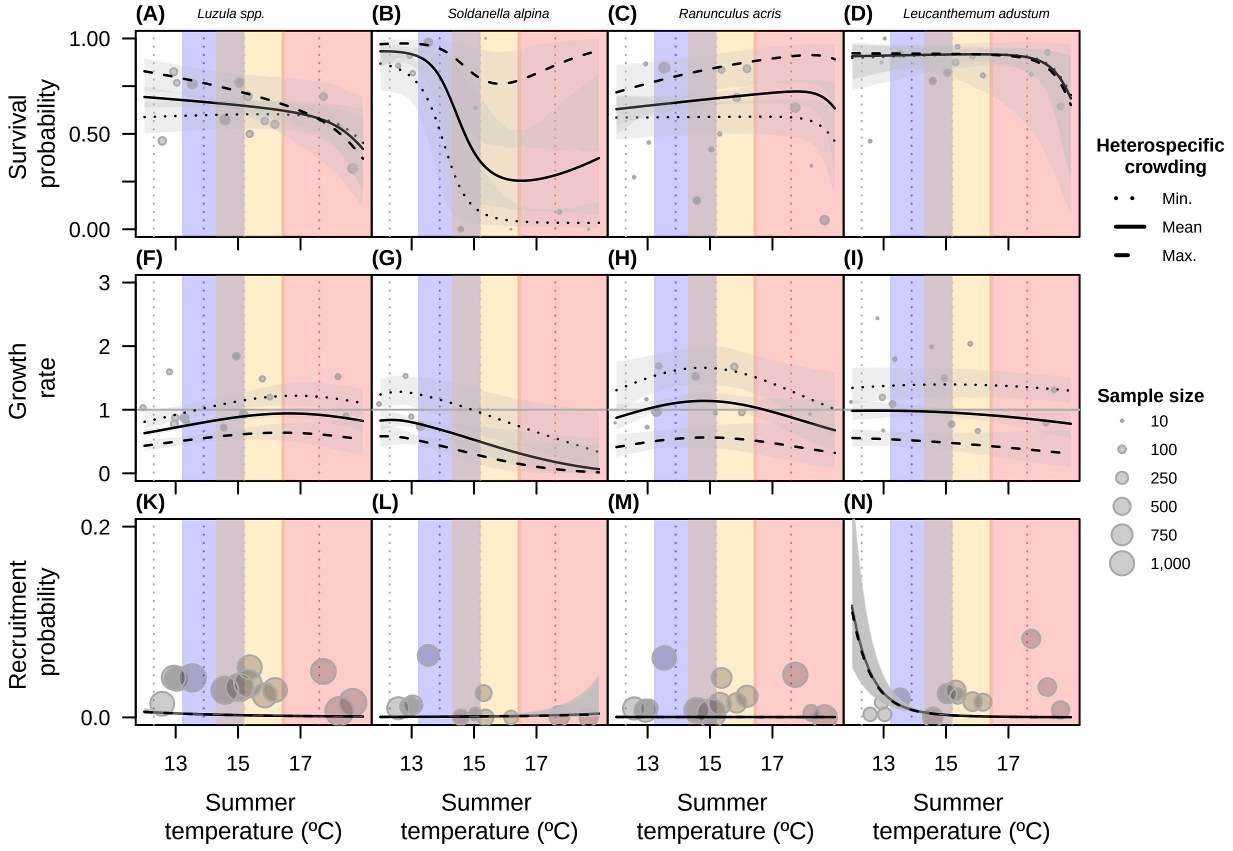

(A) Luzula spp.
(B) Soldanella alpina
(C) Ranunculus acris
(D) Leucanthemum adustum
Survival probability
1.00
0.50
0.00
Heterospecific crowding
Min.
Mean
Max.
(F)
(G)
(H)
(I)
Growth rate
3
2
1
0
Sample size
10
100
250
500
750
1,000
(K)
(L)
(M)
(N)
Recruitment probability
0.2
0.0
13
15
17
Summer temperature (ºC)
