# Supplementary material for: Ecological lags govern the pace and outcome of plant community responses to 21st‐century climate change
Source: Ecol Lett. 2022 Aug 26;25(10):2156–66. doi: 10.1111/ele.14087 (PMC9804264; doi:10.1111/ele.14087)

SM3: Simulation analysis to test model performance

If we assume a certain demographic process, we can simulate data and test our models' ability to recover the true parameter values. Here, we do this for the our models of survival, growth, and recruitment.

```
library(rethinking)
```

```
## Loading required package: rstan

## Loading required package: StanHeaders

## Loading required package: ggplot2

## Warning: package 'ggplot2' was built under R version 3.6.2

## rstan (Version 2.19.2, GitRev: 2e1f913d3ca3)

## For execution on a local, multicore CPU with excess RAM we recommend calling
## options(mc.cores = parallel::detectCores()).
## To avoid recompilation of unchanged Stan programs, we recommend calling
## rstan_options(auto_write = TRUE)

## Loading required package: parallel

## Loading required package: dagitty

## rethinking (Version 2.00)

##
## Attaching package: 'rethinking'

## The following object is masked from 'package:stats':
##
##     rstudent
```

Survival

First, let's generate data representing the true sizes of a set of ramets, trying to recreate the size distribution of one of the species in the experiment: *Ranunculus acris*.

```
## Load data of species to recreate
spcode <- "Ranacr"
fpath_d <- paste("processed-data/",spcode, "_DMD.csv", sep = "") # file path
d <- read.csv(fpath_d) # read file
dcore <- subset(d, x > 20 & x < 80 & y > 20 & y < 80) # exclude ramets near margins
sp17 <- subset(dcore, year == 2017) # data from first transition (2017 - 2018)

## Create vector of true covers
N <- nrow(dcore)
set.seed(123)
u1 <- exp(rnorm(N, 1.3, 0.6))
dens(u1, xlab = "")
mtext(expression(paste("True cover (cm"^2* ")")), 1,line = 1.8)
```

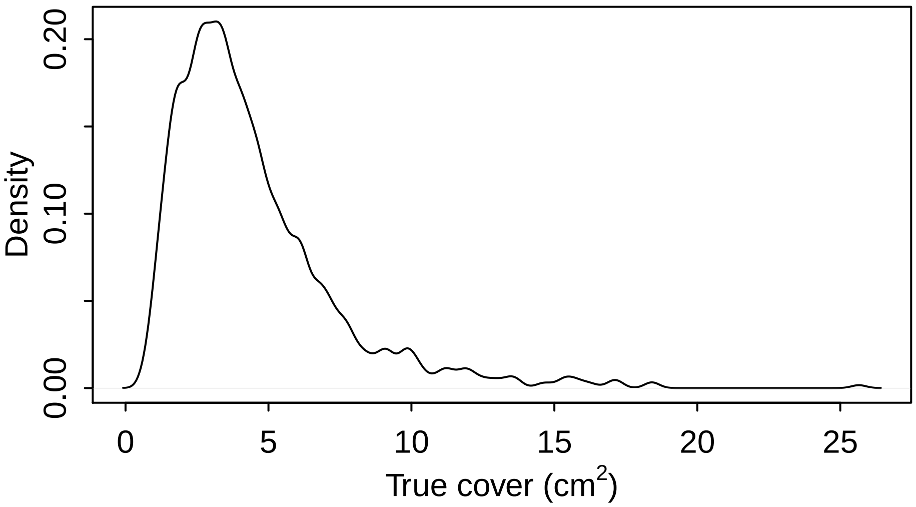

To account for the correlations between predictor variables in our dataset, we will sample predictor variables from the observed data.

```
# Sample predictor variables from the real data to keep their correlations
set.seed(321)
samp_id <- sample(1:nrow(dcore), N, replace = T)

## Temperature
temp <- dcore$tempLS[samp_id] # Sample temperature values
temp <- (temp - mean(temp))/sd(temp) # Scale values

## Moisture
moist <- dcore$moistLS[samp_id] # Sample moisterature values
moist <- (moist - mean(moist))/sd(moist) # Scale values

## Heterospecific crowding
wC <- dcore$w.cinter[samp_id]

## Conspecific crowding
w <- dcore$w[samp_id]
```

Now, let's set the true parameter values. First, the intrinsic response to temperature.

```
## Intercept
b0 <- -4 # value assumed by model, small changes do not affect model estimates

## Temperature effect
tef <- 7 # Overall magnitude of temperature response
k <- -3 # Steepness of logistic response
ttp_a <- 0.25 # Thermal optimum (scaled units)
ttp_b <- -0.5

### Plot hypothetical temperature effect
curve(tef/(1 + exp(-k*(x - ttp_a))) + b0, -1.5, 2,
      ylab = "Effect on survival", xlab = "Temperature")
```

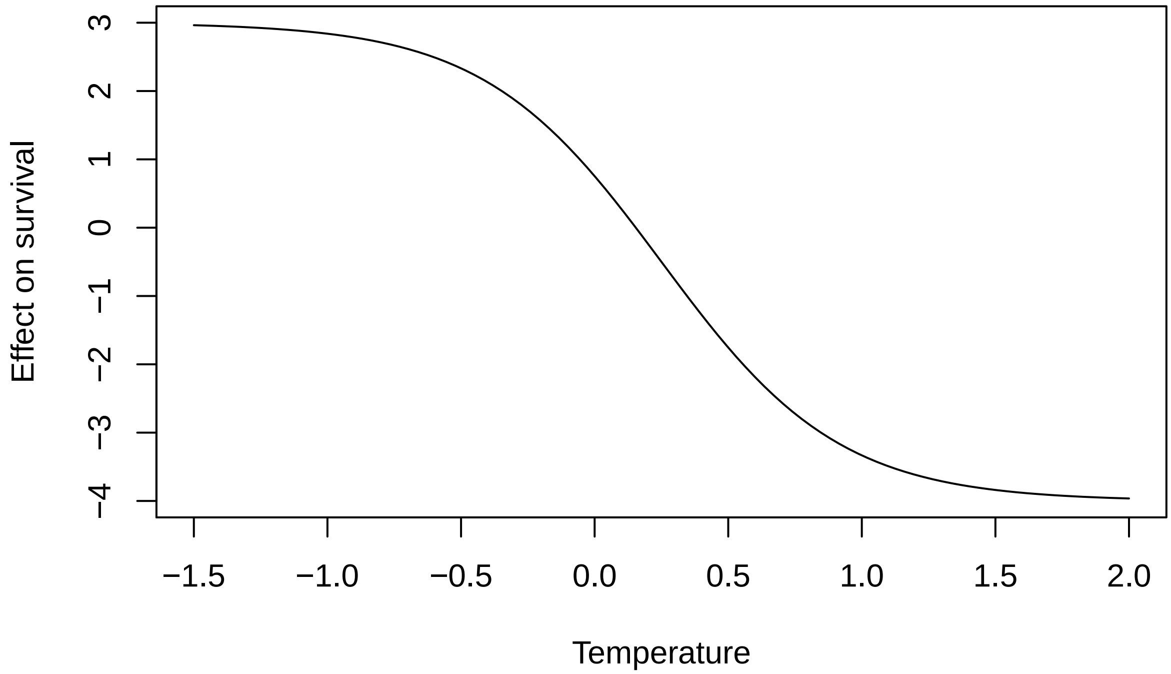


```
rT <- tef/(1 + exp(-k*(temp - (ttp_a + ttp_b*moist)))) + b0
```

Now, the temperature-dependent effects of interactions with neighbors.

```
## Response to heterospecific neighbors
aC_0 <- -0.01 # intercept (i.e., value at mean temperature)
b_aC <- -0.001 # response to temperature
aC <- aC_0 + b_aC*temp
summary(aC * mean(wC))
```

```
##    Min. 1st Qu.  Median    Mean 3rd Qu.    Max.
##  -2.346  -2.058  -2.020  -1.999  -1.872  -1.639
```

```
## Response to conspecific neighbors
a_0 <- 0.04 # intercept (i.e., value at mean temperature)
b_a <- -0.02 # response to temperature #                           # -0.01
a <- a_0 + b_a*temp
summary(a * mean(w)) # summary(a*mean(w))
```

```
##    Min. 1st Qu.  Median    Mean 3rd Qu.    Max.
##  0.0614  0.3938  0.4379  0.4614  0.6082  0.8767
```

Finally, the effect of ramet cover.

```
## Effect of ramet cover
b <- 0.6
```

Let's visualize the relationship between temperature and survival for an average-sized ramet under average crowding conditions and average moisture.

```
temprange <- seq(min(temp), max(temp), length.out = 1e3)
logoddrange <- tef/(1 + exp(5*(temprange - (ttp_a)))) + b0 +
               (a_0 + b_a*temprange)*mean(w)  +
               (aC_0 + b_aC*temprange)*mean(wC)
prange <- inv_logit(logoddrange)
```

```r
plot(temprange, prange, type = 'l', lwd = 2, ylim = c(0,1),
     xlab = 'Temperature (scaled)', ylab = 'Survival probability')
```

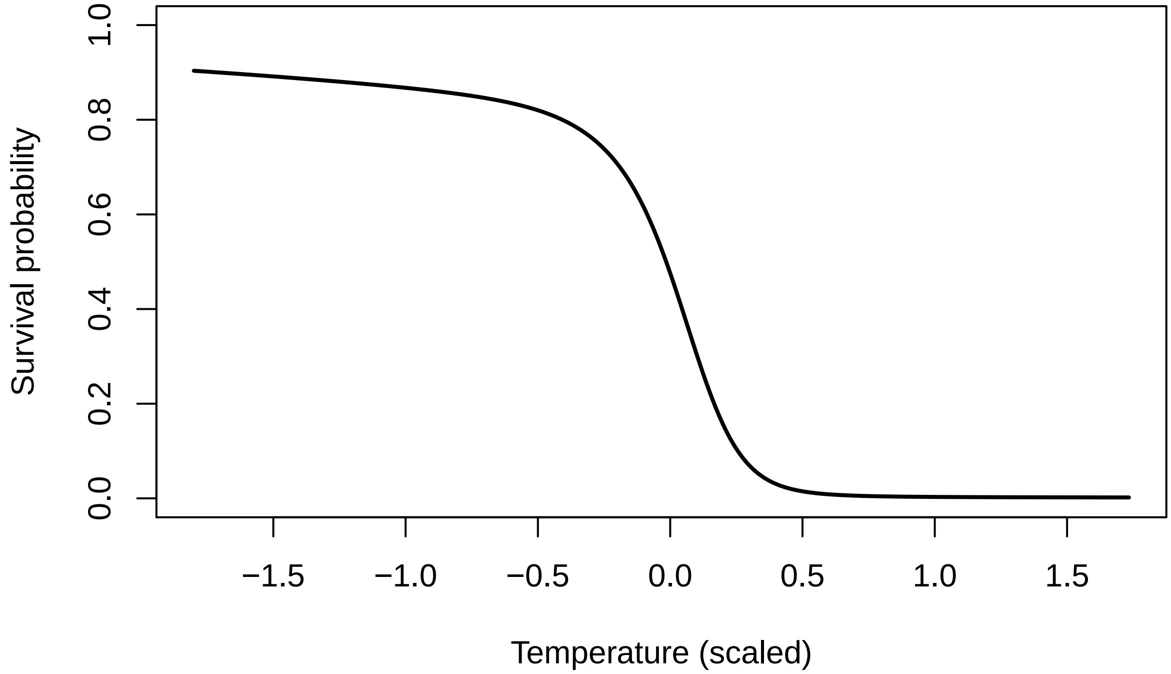

Now we can simulate the survival process of a set of ramets with our simulated cover values.

```r
# Simulate survival process
logodds <- rT + scale(u1)*b + a*w  + aC*wC
p <- inv_logit(logodds)
summary(p[,1])
```

```
##     Min.  1st Qu.   Median     Mean  3rd Qu.     Max.
## 0.002125 0.115080 0.450001 0.426420 0.690254 0.985437
```

```r
survival <- rbern(N,p)
```

Next, we simulate the data collection process.

```r
## Create vector of observed covers at time 1, assuming observation error proportional to the square ro
uobs1 <- u1 + rnorm(N, 0, sqrt(u1))

## Create vector of categorized observed covers (i.e., the available data)
ut1 <- ifelse(uobs1 < 1.56, 1.56,
              ifelse(uobs1 < 3.125, 3.125,
                     ifelse(uobs1 < 6.25, 6.25,
                            ifelse(uobs1 < 12.5, 12.5,
                                   ifelse(uobs1 < 18.75, 18.75,
                                          ifelse(uobs1 < 25, 25,
                                                 25 + 6.25*ceiling((uobs1-25)/6.25)))))))
```

Finally, we can feed these simulated data to our survival model to test its ability to recover the true parameter values.

```r
# Data to train model
d_list_sm <- list(survival = survival,
                  u = (ut1 - mean(ut1))/sd(ut1),
```

```r
                   tempLS = temp,
                   moistLS = moist,
                   w = w,
                   wC = wC)

initVals <- list(list(
                       ttp_a = 0,
                       ttp_b = 0.25,
                       b_a = 0,
                       b_aC = 0,
                       a_0 = 0,
                       aC_0 = 0,
                       k = -2,
                       tef = 8),
                  list(
                       ttp_a = 0,
                       ttp_b = 0.25,
                       b_a = 0,
                       b_aC = 0,
                       a_0 = 0,
                       aC_0 = 0,
                       k = -2,
                       tef = 8))


smod <- ulam(
        alist(
                survival ~ dbern(p),
                logit(p) <- -4 + tempR + b*u + a*w + aC*wC,
                tempR <- tef/(1 + exp(-k*(tempLS - ttp))),
                tef ~ dnorm(6, 2),
                k ~ dnorm(0,2),
                ttp <- ttp_a + ttp_b * moistLS,
                ttp_a ~ dnorm(0,2.5),
                ttp_b ~ dnorm(0,0.5),
                b ~ dnorm(0, 0.5),
                a <- a_0 + b_a * tempLS,
                a_0 ~ dnorm(0, 0.1),
                b_a ~ dnorm(0, 0.05),
                aC <- aC_0 + b_aC * tempLS,
                aC_0 ~ dnorm(0, 0.1),
                b_aC ~ dnorm(0, 0.05)
        ),
        data = d_list_sm, chains = 2, cores = 2, iter = 5500, warmup = 500,
        log_lik = T, init = initVals, control=list(adapt_delta=0.97))
```

Let’s sample parameters from the posterior and prior distributions.

```r
postparams <- extract.samples(smod, n = 1e3)
priorparams <- extract.prior(smod)
```

```
##
## SAMPLING FOR MODEL 'a58c45fbf5c516f5333fdfdd598f6b29' NOW (CHAIN 1).
## Chain 1:
```

```
## Chain 1: Gradient evaluation took 0.001061 seconds
## Chain 1: 1000 transitions using 10 leapfrog steps per transition would take 10.61 seconds.
## Chain 1: Adjust your expectations accordingly!
## Chain 1:
## Chain 1:
## Chain 1: Iteration:    1 / 2000 [  0%]  (Warmup)
## Chain 1: Iteration:  200 / 2000 [ 10%]  (Warmup)
## Chain 1: Iteration:  400 / 2000 [ 20%]  (Warmup)
## Chain 1: Iteration:  600 / 2000 [ 30%]  (Warmup)
## Chain 1: Iteration:  800 / 2000 [ 40%]  (Warmup)
## Chain 1: Iteration: 1000 / 2000 [ 50%]  (Warmup)
## Chain 1: Iteration: 1001 / 2000 [ 50%]  (Sampling)
## Chain 1: Iteration: 1200 / 2000 [ 60%]  (Sampling)
## Chain 1: Iteration: 1400 / 2000 [ 70%]  (Sampling)
## Chain 1: Iteration: 1600 / 2000 [ 80%]  (Sampling)
## Chain 1: Iteration: 1800 / 2000 [ 90%]  (Sampling)
## Chain 1: Iteration: 2000 / 2000 [100%]  (Sampling)
## Chain 1:
## Chain 1:  Elapsed Time: 8.32112 seconds (Warm-up)
## Chain 1:                2.85187 seconds (Sampling)
## Chain 1:                11.173 seconds (Total)
## Chain 1:
```

And in the plots below, let's compare the true parameter values (dashed vertical lines) with the distributions of parameters sampled from the prior (light gray) and posterior (dark gray).

```r
# Figure prior, posterior, truth
par(mfrow = c(2,5))

#dens(postparams$b0, xlim = c(-5,0.25), type = 'n')
#polygon(density(priorparams$b0),
#        col = col.alpha("gray", 0.65))
#polygon(density(postparams$b0),
#        col = col.alpha("black", 0.65))
#abline(v=b0, lty = 3, lwd = 1.5)
#mtext('Intercept (b0)', cex = 0.75)
#text(0.4, 3.8, "Posterior")
#text(0.55, 0.7, "Prior", col = "darkgray")
#text(b0-0.1, 2.5, "True parameter value", srt = 90)

dens(postparams$tef, xlim = c(0,12), type = 'n')
polygon(density(priorparams$tef),
        col = col.alpha("gray", 0.65))
polygon(density(postparams$tef),
        col = col.alpha("black", 0.65))
abline(v=tef, lty = 3, lwd = 1.5)
mtext('Thermal response (tef)', cex = 0.75)

dens(postparams$k, xlim = c(-9,9), type = 'n')
polygon(density(priorparams$k),
        col = col.alpha("gray", 0.65))
polygon(density(postparams$k),
        col = col.alpha("black", 0.65))
abline(v=k, lty = 3, lwd = 1.5)
mtext('Steepness thermal response (k)', cex = 0.75)
```

```
dens(postparams$ttp_a, xlim = c(-6,6), type = 'n')
polygon(density(priorparams$ttp_a),
        col = col.alpha("gray", 0.65))
polygon(density(postparams$ttp_a),
        col = col.alpha("black", 0.65))
abline(v=ttp_a, lty = 3, lwd = 1.5)
mtext('Thermal optimum (ttp_a)', cex = 0.75)

dens(postparams$ttp_b, xlim = c(-2,2), type = 'n')
polygon(density(priorparams$ttp_b),
        col = col.alpha("gray", 0.65))
polygon(density(postparams$ttp_b),
        col = col.alpha("black", 0.65))
abline(v=ttp_b, lty = 3, lwd = 1.5)
mtext('Optimum resp. moisture (ttp_b)', cex = 0.75)

dens(postparams$b, xlim = c(-2,2), type = 'n')
polygon(density(priorparams$b),
        col = col.alpha("gray", 0.65))
polygon(density(postparams$b),
        col = col.alpha("black", 0.65))
abline(v=b, lty = 3, lwd = 1.5)
mtext('Size effect (b)', cex = 0.75)

dens(postparams$a_0, xlim = c(-0.4,0.4), type = 'n')
polygon(density(priorparams$a_0),
        col = col.alpha("gray", 0.65))
polygon(density(postparams$a_0),
        col = col.alpha("black", 0.65))
abline(v=a_0, lty = 3, lwd = 1.5)
mtext('a_0', cex = 0.75)

dens(postparams$b_a, xlim = c(-0.3,0.3), type = 'n')
polygon(density(priorparams$b_a),
        col = col.alpha("gray", 0.65))
polygon(density(postparams$b_a),
        col = col.alpha("black", 0.65))
abline(v=b_a, lty = 3, lwd = 1.5)
mtext('b_a', cex = 0.75)

dens(postparams$aC_0, xlim = c(-0.1,0.1), type = 'n')
polygon(density(priorparams$aC_0),
        col = col.alpha("gray", 0.65))
polygon(density(postparams$aC_0),
        col = col.alpha("black", 0.65))
abline(v=aC_0, lty = 3, lwd = 1.5)
mtext('aC_0', cex = 0.75)

dens(postparams$b_aC, xlim = c(-0.05,0.05), type = 'n')
polygon(density(rnorm(4e3, 0, 0.25)),
        col = col.alpha("gray", 0.65))
polygon(density(postparams$b_aC),
        col = col.alpha("black", 0.65))
```

```r
abline(v=b_aC, lty = 3, lwd = 1.5)
mtext('b_aC', cex = 0.75)
```

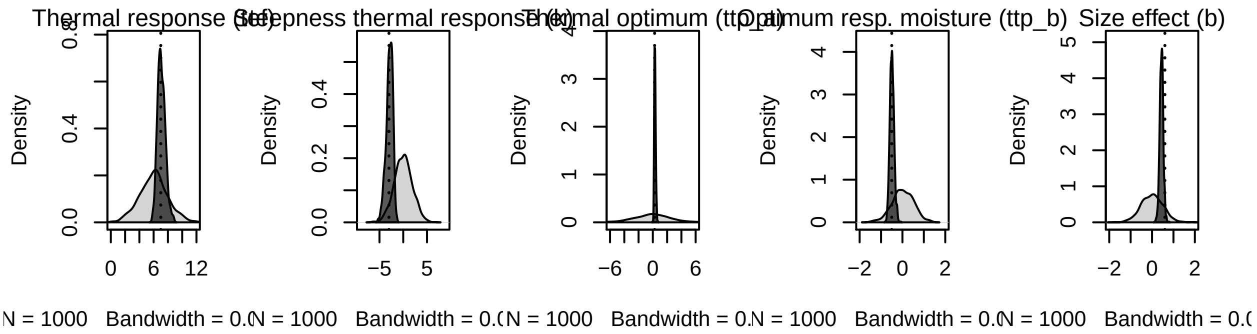

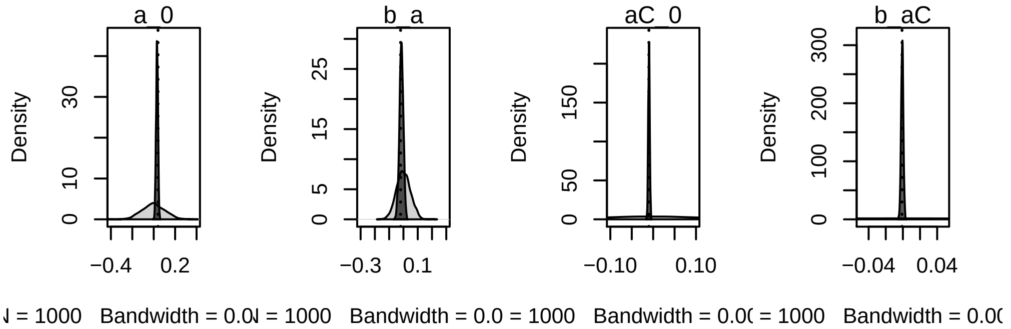

Except for a small negative bias in the estimate of the effect of ramet size (b), it seems the model does a good job recovering the true parameter values. The model still performs well when the true intercept is different slightly different from -4 (i.e., between -3 and -5, at least).

Growth

Let's now simulate the growth process. We start by setting the true parameter values. First the direct effect of temperature.

```r
## Intercept
b0 <- -0.55

## Temperature effect
tef <- 2.5 # Overall magnitude of temperature response
ttp <- 1.5 # Thermal optimum (scaled units)
tsd <- 0.5 # Thermal tolerance or niche breadth


rT <- tef*exp(-((temp - ttp)^2/2*tsd^2))

### Plot hypothetical temperature effect
curve(tef*exp(-((x - ttp)^2/2*tsd^2)) + b0, -2, 1.5,
      ylab = "Effect on growth", xlab = "Temperature")
```

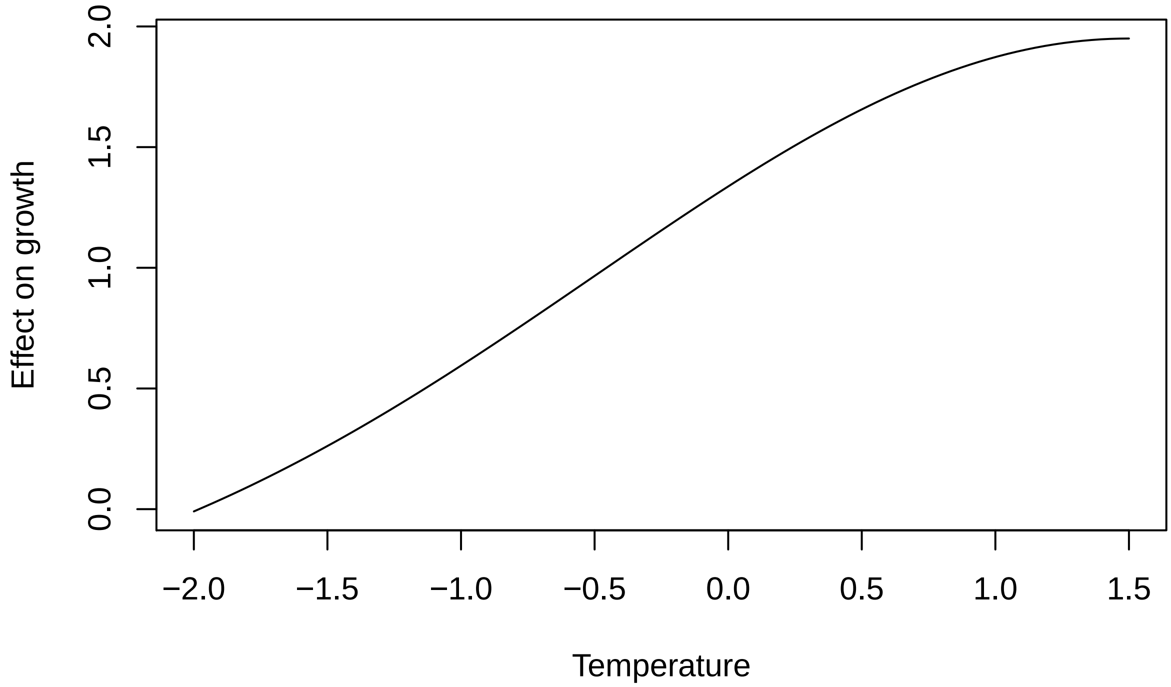

Next, the temperature-dependent neighbor effects.

```
## Response to heterospecific neighbors
aC_0 <- -5 # intercept (i.e., value at mean temperature)
b_aC <- 0.3 # response to temperature
aC <- exp(aC_0 + b_aC*temp)
summary(aC*mean(wC))
```

```
##    Min. 1st Qu.  Median    Mean 3rd Qu.    Max.
##   0.785   1.113   1.389   1.410   1.471   2.266
```

```
## Response to conspecific neighbors
a_0 <- -6 # intercept (i.e., value at mean temperature)
b_a <- -0.25 # response to temperature
a <- exp(a_0 + b_a*temp)
summary(a*mean(w))
```

```
##    Min. 1st Qu.  Median    Mean 3rd Qu.    Max.
## 0.01853 0.02657 0.02787 0.02947 0.03352 0.04484
```

And finally, the effect of ramet size and the standard deviation of the normal distribution of growth rates.

```
## Effect of ramet cover (a reasonable range is within -0.3 and -0.45)
b <- -0.25

## Growth variance
sigma <- 0.1
```

Then, before simulating growth, let's update the temperature vector with the appropriate predictor for the growth model: the average temperature of the two previous growing seasons.

```
## Temperature
temp <- dcore$tempL2S[samp_id] # Sample temperature values
temp <- (temp - mean(temp))/sd(temp) # Scale values
```

Now we can simulate growth.

```
# Simulate growth process
mu <- b0 + rT + log(u1)*b - a*w - aC*wC
g <- rnorm(N, mu, sigma)
summary(g)
```

```
##     Min.  1st Qu.   Median     Mean  3rd Qu.     Max. 
## -2.14835 -0.92539 -0.41997 -0.47164 -0.00773  1.14346
```

```
## Calculate true cover at time t+1
u2 <- u1*exp(g)

summary(u1)
```

```
##    Min. 1st Qu.  Median    Mean 3rd Qu.    Max. 
##  0.6799  2.5429  3.7272  4.4597  5.5303 25.6521
```

```
summary(u2)
```

```
##    Min. 1st Qu.  Median    Mean 3rd Qu.    Max. 
##  0.2016  1.4222  2.3855  2.9861  3.8328 22.9052
```

```
dens(u1, ylim = c(0,0.5))
dens(u2, col = "blue", add = T)
```

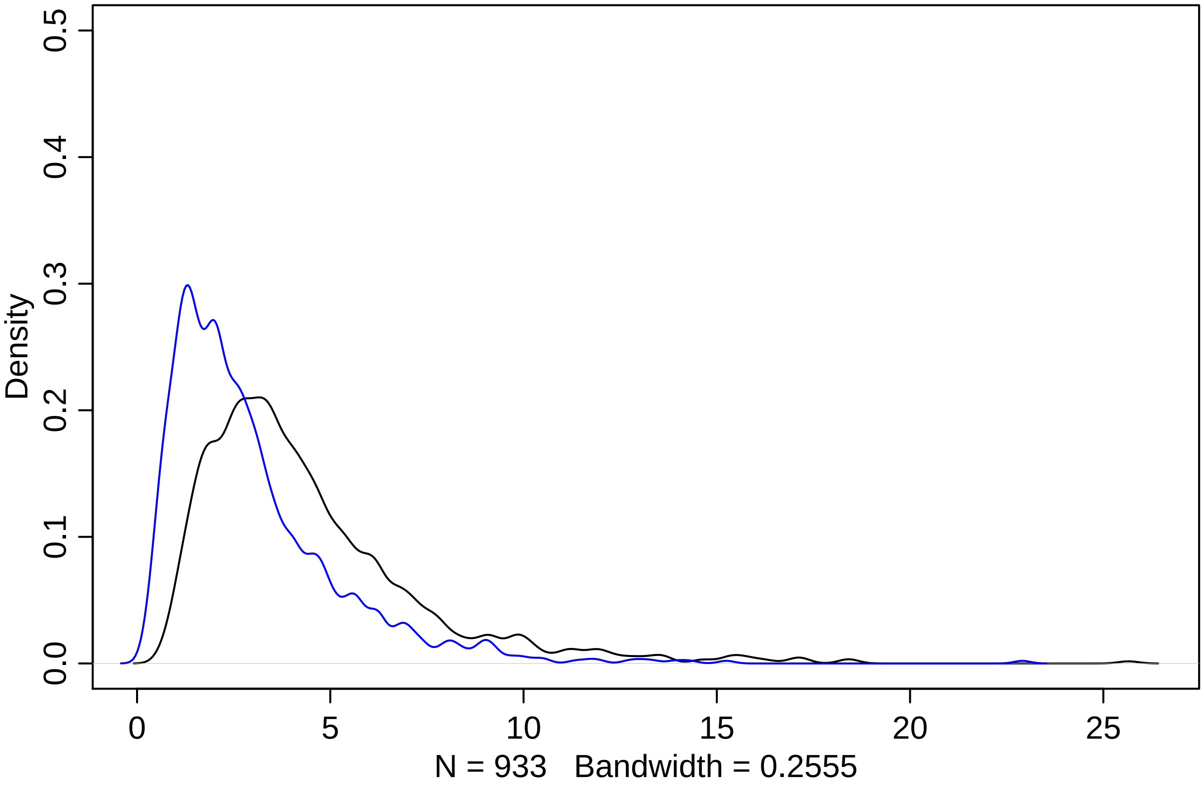

Now let's simulate the data collection process.

```
## Create vector of observed covers at time 1, assuming observation error proportional to the square ro
uobs1 <- u1 + rnorm(N, 0, sqrt(u1))

## Create vector of categorized observed covers (i.e., the available data)
ut1 <- ifelse(uobs1 < 1.56, 1.56,
```

```r
                ifelse(uobs1 < 3.125, 3.125,
                       ifelse(uobs1 < 6.25, 6.25,
                              ifelse(uobs1 < 12.5, 12.5,
                                     ifelse(uobs1 < 18.75, 18.75,
                                            ifelse(uobs1 < 25, 25,
                                                   25 + 6.25*ceiling((uobs1-25)/6.25)))))))



## Compare simulated with actually observed covers
summary(sp17$u1)
```

```
##    Min. 1st Qu.  Median    Mean 3rd Qu.    Max.
##   1.562   1.562   3.125   3.359   3.125  18.750
```

```r
summary(ut1)
```

```
##    Min. 1st Qu.  Median    Mean 3rd Qu.    Max.
##   1.560   3.125   6.250   6.355   6.250  25.000
```

```r
dens(ut1, col = "blue")
dens(sp17$u1, col = "red", add = T)
```

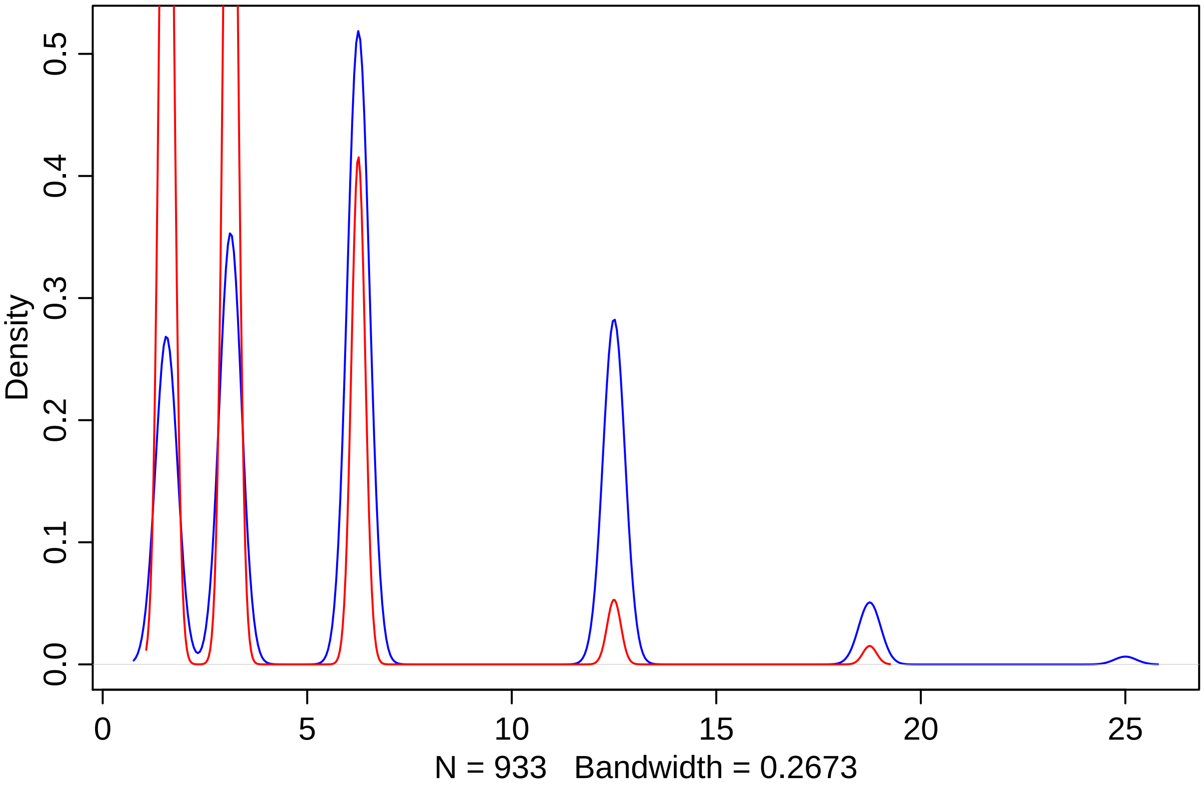


```r
## Create vector of observed covers at time 2, assuming observation error proportional to the square ro
uobs2 <- u2 + rnorm(N, 0, sqrt(u2))

## Create vector of categorized observed covers (i.e., the available data)
ut2 <- ifelse(uobs2 < 1.56, 1.56,
              ifelse(uobs2 < 3.125, 3.125,
                     ifelse(uobs2 < 6.25, 6.25,
```

```
                                        ifelse(uobs2 < 12.5, 12.5,
                                                  ifelse(uobs2 < 18.75, 18.75,
                                                            ifelse(uobs2 < 25, 25,
                                                                      25 + 6.25*ceiling((uobs2-25)/6.25)))))))
```

Finally, let's use the growth model to recover the true parameter values from the simulated data.

```
# Define lower bounds
ut2min <- ifelse(ut2 == (1.56), (0.25),
                 ifelse(ut2 == (3.125), (1.56),
                        ifelse(ut2 == (6.25), (3.125),
                               ifelse(ut2 == (12.5), (6.25),
                                      ifelse(ut2 == (18.75), (12.5),
                                             ifelse(ut2 == (25), (18.75),
                                                    (ut2 - 6.25)))))))

ut1min <- ifelse(ut1 == (1.56), (0.25),
                 ifelse(ut1 == (3.125), (1.56),
                        ifelse(ut1 == (6.25), (3.125),
                               ifelse(ut1 == (12.5), (6.25),
                                      ifelse(ut1 == (18.75), (12.5),
                                             ifelse(ut1 == (25), (18.75),
                                                    (ut1 - 6.25)))))))

# Data to train model
simdat <- list(
        u2max = ut2,
        u1max = ut1,
        u2min = ut2min,
        u1min = ut1min,
        wC = wC,
        w = w,
        temp = temp
)


# Model Stan code
gmodcode = "data{
        vector[howmany] u2max;
        vector[howmany] u1max;
        vector[howmany] u2min;
        vector[howmany] u1min;
        vector[howmany] wC;
        vector[howmany] w;
        vector[howmany] temp;
}
parameters{
        real b0;
        real<lower=0> tef;
        real<lower=0> tsd;
        real ttp;
        real b;
        real a_0;
        real b_a;
```

```
        real aC_0;
        real b_aC;
        real<lower=0> sigma;
        vector[howmany] u2;
        vector[howmany] u1;
}
model{
        vector[howmany] mu;
        vector[howmany] g;
        vector[howmany] rT;
        vector[howmany] a;
        vector[howmany] aC;

        tef ~ exponential( 1 );
        tsd ~ exponential( 0.5 );
        ttp ~ normal( 0 , 1.5 );

        for ( i in 1:howmany ) {
            rT[i] = tef*exp(-((temp[i] - ttp)^2/2*tsd^2));
        }

        b_aC ~ normal( 0 , 0.25 );
        aC_0 ~ normal( -6 , 1 );
        for ( i in 1:howmany ) {
            aC[i] = exp(aC_0 + b_aC * temp[i]);
        }

        b_a ~ normal( 0 , 0.25 );
        a_0 ~ normal( -6 , 1 );
        for ( i in 1:howmany ) {
            a[i] = exp(a_0 + b_a * temp[i]);
        }

        b0 ~ normal(-0.7, 0.2);

        b ~ normal(0, 0.05);

        sigma ~ exponential(1);

        u1 ~ normal(u1max - (u1max-u1min)/2, u1max/10);
        u2 ~ normal(u2max - (u2max-u2min)/2, u2max/10);

        for ( i in 1:howmany ) {
                g[i] = log(u2[i] / u1[i]);
                mu[i] = b0 + rT[i] + log(u1[i])*b - aC[i]*wC[i] - a[i]*w[i];
        }
        g ~ normal(mu , sigma );
}
"
gcodesp = gsub("howmany", N, gmodcode, fixed = T)


## Initial values
```

```r
initVals_gm <- list(list(u1 = rnorm(N, ut1 - (ut1-ut1min)/2, ut1/10),
                         mu = 0,
                         u2 = rnorm(N, ut2 - (ut2-ut2min)/2, ut2/10),
                         a_0 = -6,
                         b_a = 0,
                         aC_0 = -6,
                         b_aC = 0,
                         tef = 1,
                         tsd = 1,
                         ttp = 0,
                         b = -0.5,
                         b0 = -0.7),
                    list(u1 = rnorm(N, ut1 - (ut1-ut1min)/2, ut1/10),
                         mu = 0,
                         u2 = rnorm(N, ut2 - (ut2-ut2min)/2, ut2/10),
                         a_0 = -6,
                         b_a = 0,
                         aC_0 = -6,
                         b_aC = 0,
                         tef = 1,
                         tsd = 1,
                         ttp = 0,
                         b = -0.5,
                         b0 = -0.7))

gmod <- stan(model_code = gcodesp, data = simdat, chains = 2, cores = 2,
             init = initVals_gm, iter = 1500, warmup = 500,
             control = list(adapt_delta = 0.95, max_treedepth = 15))
```

Let's compare the prior and posterior distribution of the model parameters with their true values.

```r
postparams <- extract.samples(gmod)


# Figure prior, posterior, truth
par(mfrow = c(2,5))

dens(postparams$b0, xlim = c(-1.5,0.25), type = 'n')
polygon(density(rnorm(4e3, -0.7, 0.2)),
        col = col.alpha("gray", 0.65))
polygon(density(postparams$b0),
        col = col.alpha("black", 0.65))
abline(v=b0, lty = 3, lwd = 1.5)
mtext('Intercept (b0)', cex = 0.75)


dens(postparams$tef, xlim = c(-0.5,6), type = 'n')
polygon(density(rexp(4e3, 1)),
        col = col.alpha("gray", 0.65))
polygon(density(postparams$tef),
        col = col.alpha("black", 0.65))
abline(v=tef, lty = 3, lwd = 1.5)
mtext('Thermal response (tef)', cex = 0.75)
text(4.2, 1.5, "Posterior")
```

```r
text(0.55, 0.85, "Prior", col = "darkgray")
#

dens(postparams$tsd, xlim = c(0,2), type = 'n')
polygon(density(rexp(4e3, 0.5)),
        col = col.alpha("gray", 0.65))
polygon(density(postparams$tsd),
        col = col.alpha("black", 0.65))
abline(v=tsd, lty = 3, lwd = 1.5)
mtext('Thermal tolerance (tsd)', cex = 0.75)

dens(postparams$ttp, xlim = c(-3,3), type = 'n')
polygon(density(rnorm(4e3, 0, 0.5)),
        col = col.alpha("gray", 0.65))
polygon(density(postparams$ttp),
        col = col.alpha("black", 0.65))
abline(v=ttp, lty = 3, lwd = 1.5)
mtext('Thermal optimum (ttp)', cex = 0.75)

dens(postparams$b, xlim = c(-0.5,0), type = 'n')
polygon(density(rnorm(4e3, 0, 0.1)),
        col = col.alpha("gray", 0.65))
polygon(density(postparams$b),
        col = col.alpha("black", 0.65))
abline(v=b, lty = 3, lwd = 1.5)
mtext('Size effect (b)', cex = 0.75)
text(b-0.05, 7, "True parameter value", srt = 90)

dens(postparams$a_0, xlim = c(-8,-2), type = 'n')
polygon(density(rnorm(4e3, -5, 0.5)),
        col = col.alpha("gray", 0.65))
polygon(density(postparams$a_0),
        col = col.alpha("black", 0.65))
abline(v=a_0, lty = 3, lwd = 1.5)
mtext('a_0', cex = 0.75)

dens(postparams$b_a, xlim = c(-1,1), type = 'n')
polygon(density(rnorm(4e3, 0, 0.25)),
        col = col.alpha("gray", 0.65))
polygon(density(postparams$b_a),
        col = col.alpha("black", 0.65))
abline(v=b_a, lty = 3, lwd = 1.5)
mtext('b_a', cex = 0.75)

dens(postparams$aC_0, xlim = c(-8,-2), type = 'n')
polygon(density(rnorm(4e3, -5, 0.5)),
        col = col.alpha("gray", 0.65))
polygon(density(postparams$aC_0),
        col = col.alpha("black", 0.65))
abline(v=aC_0, lty = 3, lwd = 1.5)
mtext('aC_0', cex = 0.75)

dens(postparams$b_aC, xlim = c(-1,1), type = 'n')
```

```r
polygon(density(rnorm(4e3, 0, 0.25)),
        col = col.alpha("gray", 0.65))
polygon(density(postparams$b_aC),
        col = col.alpha("black", 0.65))
abline(v=b_aC, lty = 3, lwd = 1.5)
mtext('b_aC', cex = 0.75)


dens(postparams$sigma, xlim = c(-0.5,2), type = 'n')
polygon(density(rexp(4e3, 1)),
        col = col.alpha("gray", 0.65))
polygon(density(postparams$sigma),
        col = col.alpha("black", 0.65))
abline(v=sigma, lty = 3, lwd = 1.5)
mtext('sigma', cex = 0.75)
```

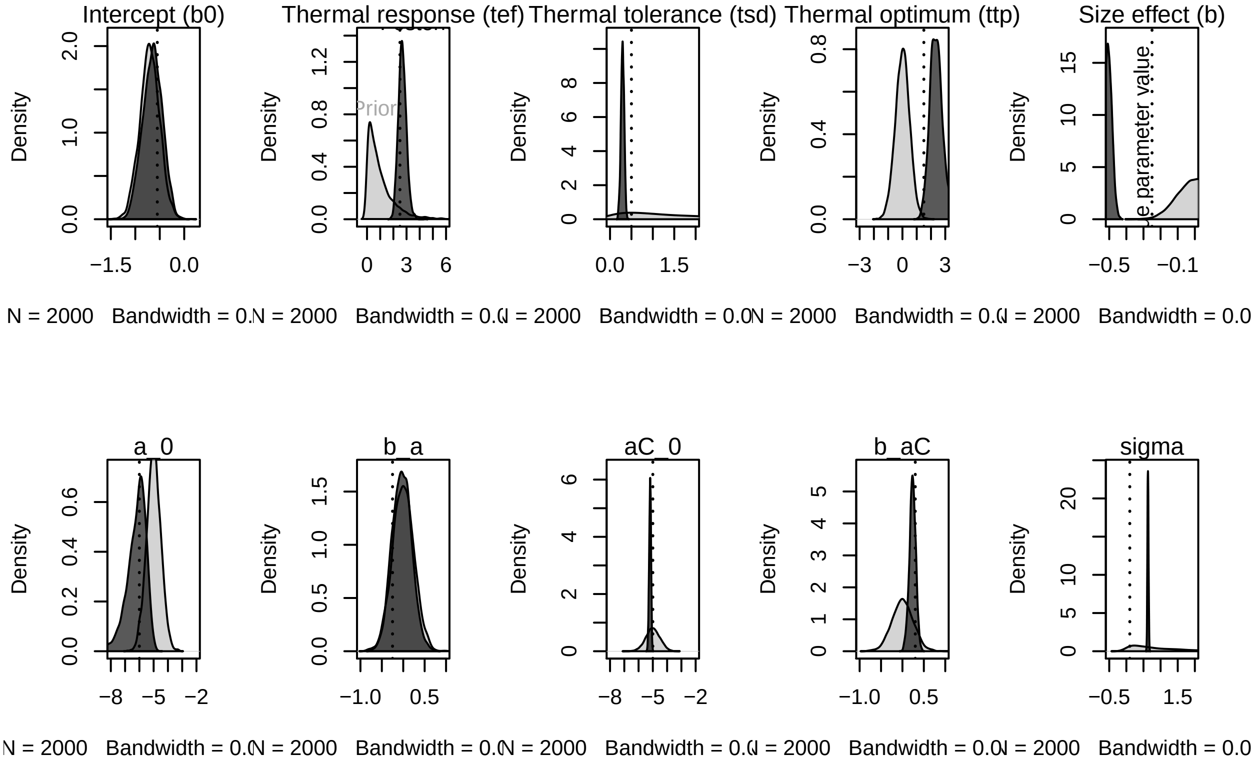

Again, despite the categorization of cover values and observation error, the model is able to make reasonable estimates of the true values of most parameters. There two particularly notable exceptions. The first is a strong negative bias in the estimates of the size effect parameter `b` (reason why the prior probability is centered around 0, despite strong reasons to expect values between -0.2 and -0.5). The second is a strong postive bias in the estimates of `sigma`. There also seems to be a negative bias in the estimates of thermal tolerance `tsd`. Moreover, the prior and posterior distributions of the intercept and of the temperature-dependence of conspecific effects `b_a` are nearly identical, which suggests that data are insufficient to properly estimate these parameters.

Recruitment

Finally, let's do the same simulation analysis with the recruitment model. First we load the recruitment table of our model species and sample predictor variables to maintain their correlations in the dataset.

```
fpath_d <- paste("processed-data/",spcode, "_RD.csv", sep = "")
d <- read.csv(fpath_d)
dcore <- subset(d, x > 20 & x < 80 & y > 20 & y < 80)

N <- nrow(dcore)
## Create vector of true covers
set.seed(123)

# Sample predictor variables from the real data to keep their correlations
samp_id <- sample(1:nrow(dcore), N, replace = T)

## Temperature
temp <- dcore$tempLS[samp_id] # Sample temperature values
temp <- (temp - mean(temp))/sd(temp) # Scale values

## Heterospecific crowding
wC <- dcore$w.cinter[samp_id]

## Conspecific crowding
w <- dcore$w[samp_id]
```

Now we set the true parameter values of the imaginary recruitment process.

```
aC_0 <- -5.5 # intercept (i.e., value at mean temperature)
b_aC <- -0.2 # response to temperature
aC <- exp(aC_0 + b_aC*temp)
summary(aC * median(wC))
```

```
##    Min. 1st Qu.  Median    Mean 3rd Qu.    Max.
##  0.5128  0.6677  0.7536  0.7553  0.8845  0.9798
```

```
summary(aC * max(wC))
```

```
##    Min. 1st Qu.  Median    Mean 3rd Qu.    Max.
##   1.793   2.335   2.636   2.642   3.094   3.427
```

```
## Response to conspecific neighbors
a_0 <- -0.5 # intercept (i.e., value at mean temperature)
b_a <- -0.1 # response to temperature
a <- exp(a_0 + b_a*temp)
summary(a * quantile(w, 0.9)) # summary(a*mean(w))
```

```
##    Min. 1st Qu.  Median    Mean 3rd Qu.    Max.
##   2.159   2.464   2.618   2.608   2.836   2.985
```

```
summary(a * max(w))
```

```
##    Min. 1st Qu.  Median    Mean 3rd Qu.    Max.
##   31.70   36.17   38.43   38.29   41.64   43.82
```

We simulate recruitment.

```
# Simulate recruitment process
logodds <- a*w  - aC*wC - 10
```

```r
p <- inv_logit(logodds)
summary(p)
```

```
##      Min.   1st Qu.    Median      Mean   3rd Qu.      Max.
## 0.0000030 0.0000180 0.0000255 0.0173377 0.0000351 1.0000000
```

```r
recruitment <- rbern(N,p)
sum(recruitment)
```

```
## [1] 216
```

And use the model to recover parameter values from simulated data.

```r
# Data to train model
d_list_rm <- list(recruitment = recruitment,
                  tempLS = temp,
                  w = w,
                  wC = wC)

initVals <- list(list(a_0 = -2,
                      b_a = 0,
                      aC_0 = -5,
                      b_aC = 0),
                 list(a_0 = -2,
                      b_a = 0,
                      aC_0 = -5,
                      b_aC = 0))

rmod <- ulam(
        alist(
                recruitment ~ dbern(p),
                logit(p) <- a*w - aC*wC - 10,
                a <- exp(a_0 + b_a * tempLS),
                a_0 ~ dnorm(-2, 1.5),
                b_a ~ dnorm(0, 0.5),
                aC <- exp(aC_0 + b_aC * tempLS),
                aC_0 ~ dnorm(-5, 1.5),
                b_aC ~ dnorm(0, 0.5)
        ),
        data = d_list_rm, chains = 2, cores = 2, iter = 3500, warmup = 500,
        log_lik = F, init = initVals)
```

Let's compare the prior and posterior distributions of the parameters to their true values.

```r
postparams <- extract.samples(rmod, n = 1e3)
priorparams <- extract.prior(rmod)
```

```
##
## SAMPLING FOR MODEL '45c1440894153f12db4bf59ddc662188' NOW (CHAIN 1).
## Chain 1:
## Chain 1: Gradient evaluation took 0.006752 seconds
## Chain 1: 1000 transitions using 10 leapfrog steps per transition would take 67.52 seconds.
## Chain 1: Adjust your expectations accordingly!
## Chain 1:
## Chain 1:
## Chain 1: Iteration:    1 / 2000 [  0%]  (Warmup)
## Chain 1: Iteration:  200 / 2000 [ 10%]  (Warmup)
```

```
## Chain 1: Iteration:  400 / 2000 [ 20%]  (Warmup)
## Chain 1: Iteration:  600 / 2000 [ 30%]  (Warmup)
## Chain 1: Iteration:  800 / 2000 [ 40%]  (Warmup)
## Chain 1: Iteration: 1000 / 2000 [ 50%]  (Warmup)
## Chain 1: Iteration: 1001 / 2000 [ 50%]  (Sampling)
## Chain 1: Iteration: 1200 / 2000 [ 60%]  (Sampling)
## Chain 1: Iteration: 1400 / 2000 [ 70%]  (Sampling)
## Chain 1: Iteration: 1600 / 2000 [ 80%]  (Sampling)
## Chain 1: Iteration: 1800 / 2000 [ 90%]  (Sampling)
## Chain 1: Iteration: 2000 / 2000 [100%]  (Sampling)
## Chain 1:
## Chain 1:  Elapsed Time: 21.0063 seconds (Warm-up)
## Chain 1:                16.8804 seconds (Sampling)
## Chain 1:                37.8866 seconds (Total)
## Chain 1:
```

```
# Figure prior, posterior, truth
par(mfrow = c(2,2))

dens(postparams$a_0, xlim = c(-8,4), type = 'n')
polygon(density(priorparams$a_0),
        col = col.alpha("gray", 0.65))
polygon(density(postparams$a_0),
        col = col.alpha("black", 0.65))
abline(v=a_0, lty = 3, lwd = 1.5)
mtext('a_0', cex = 0.75)
#text(0.4, 3.8, "Posterior")
#text(0.55, 0.7, "Prior", col = "darkgray")
#text(b0-0.1, 2.5, "True parameter value", srt = 90)

dens(postparams$b_a, xlim = c(-1.5,1.5), type = 'n')
polygon(density(priorparams$b_a),
        col = col.alpha("gray", 0.65))
polygon(density(postparams$b_a),
        col = col.alpha("black", 0.65))
abline(v=b_a, lty = 3, lwd = 1.5)
mtext('b_a', cex = 0.75)

dens(postparams$aC_0, xlim = c(-11,1), type = 'n')
polygon(density(priorparams$aC_0),
        col = col.alpha("gray", 0.65))
polygon(density(postparams$aC_0),
        col = col.alpha("black", 0.65))
abline(v=aC_0, lty = 3, lwd = 1.5)
mtext('aC_0', cex = 0.75)
#text(0.4, 3.8, "Posterior")
#text(0.55, 0.7, "Prior", col = "darkgray")
#text(b0-0.1, 2.5, "True parameter value", srt = 90)

dens(postparams$b_aC, xlim = c(-1.5,1.5), type = 'n')
polygon(density(priorparams$b_aC),
        col = col.alpha("gray", 0.65))
polygon(density(postparams$b_aC),
        col = col.alpha("black", 0.65))
```

```r
abline(v=b_aC, lty = 3, lwd = 1.5)
mtext('b_aC', cex = 0.75)
```

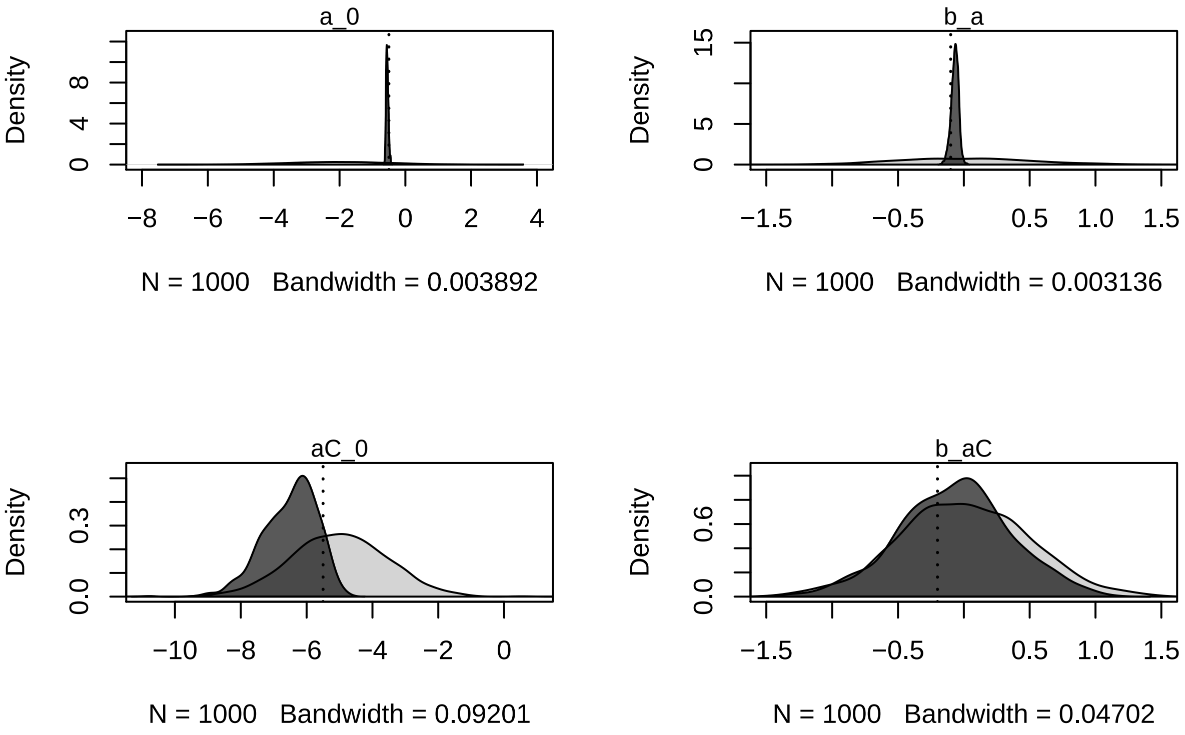

Supplement: Supplementary file 3 — Appendix S3 [file ELE-25-2156-s003.pdf]
