# Supplementary material for: Ecological lags govern the pace and outcome of plant community responses to 21st‐century climate change
Source: Ecol Lett. 2022 Aug 26;25(10):2156–66. doi: 10.1111/ele.14087 (PMC9804264; doi:10.1111/ele.14087)
Supplement: Supplementary file 4 — Appendix S4 [file ELE-25-2156-s001.pdf]

SM4: Post hoc simulation analyses to test model with sampling problems

Sampling the posterior distribution of parameters was inefficient and/or divergent transitions arose when fitting demographic models to the data of a few taxa. In the *a priori* round of simulation analyses to test the general performance of demographic models (Supporting Material 3), we noticed that divergent transitions sometimes emerged during sampling even when the models accurately estimated parameter values. Thus, when divergent transitions arose during model fitting, we did a *post hoc* simulation analysis to diagnose problems with parameter estimations, and thus decide whether we could include those taxa in downstream analyses.

We used the mean of the parameters sampled by the model to simulate new data. Then, we tested whether the model would again estimate similar parameter values when confronted with the simulated data. To demonstrate the approeach, here is the code and results of simulation analyses for one taxa in which we identified estimation problems: *Salix retusa*.

The posterior sampling for the growth model fit to *Salix retusa*'s data was inefficient and divergent transitions emerged when we ran the model for more iterations. Moreover, the model estimated an extremely large value for the parameter σ_T (`tsd`), which resulted in a small value of thermal niche breath. When simulating population dynamics, the extreme steepness and narrowness of *Salix*'s thermal niche resulted in massive population fluctuations in response to small interannual temperature variability. All of this pointed to problem with parameter estimation.

```
library(rethinking)
gmod <- readRDS("model-fits/demography/Salret_gmod.rds")
precis(gmod)
```

```
##              mean         sd       5.5%       94.5%     n_eff     Rhat4
## b0     0.07585605 0.14953938 -0.2038195  0.26411095  488.3333 1.0182211
## tef    2.03037623 0.89815262  1.0959024  3.71598434 1973.4640 1.0021762
## tsd    1.95933705 0.97759367  0.7913428  2.85011921  216.1649 1.0210684
## ttp   -0.61472883 0.14184306 -0.8107451 -0.47943863  251.0761 1.0200088
## b     -0.32828204 0.03924395 -0.3906290 -0.26467942 4969.9677 1.0000750
## a_0   -6.15174581 0.44722704 -6.8798471 -5.44026906 8459.6589 0.9994865
## b_a   -0.02532511 0.23878164 -0.4027561  0.35635117 6844.8387 0.9993972
## aC_0  -6.41406766 0.38634553 -7.0209811 -5.79743592  531.4403 1.0111461
## b_aC  -0.40213973 0.19006795 -0.6951919 -0.08325796  769.8381 1.0065475
## sigma  0.54711877 0.02469086  0.5091367  0.58795363 4677.2097 1.0000924
```

First, let's generate data representing the true sizes of a set of ramets, trying to recreate the size distribution of *Salix retusa*.

```
## Load data of species to recreate
spcode <- "Salret"
fpath_d <- paste("processed-data/", spcode, "_DMD.csv", sep = "") # file path
d <- read.csv(fpath_d) # read file
dcore <- subset(d, x > 20 & x < 80 & y > 20 & y < 80) # exclude ramets near margins
sp17 <- subset(dcore, year == 2017) # data from first transition (2017 - 2018)

## Create vector of true covers
N <- nrow(dcore)
set.seed(123)
u1 <- exp(rnorm(N, 0.6, 0.6))
dens(u1, xlab = "")
```

```
mtext(expression(paste("True cover (cm"^2* ")")), 1,line = 1.8)
```

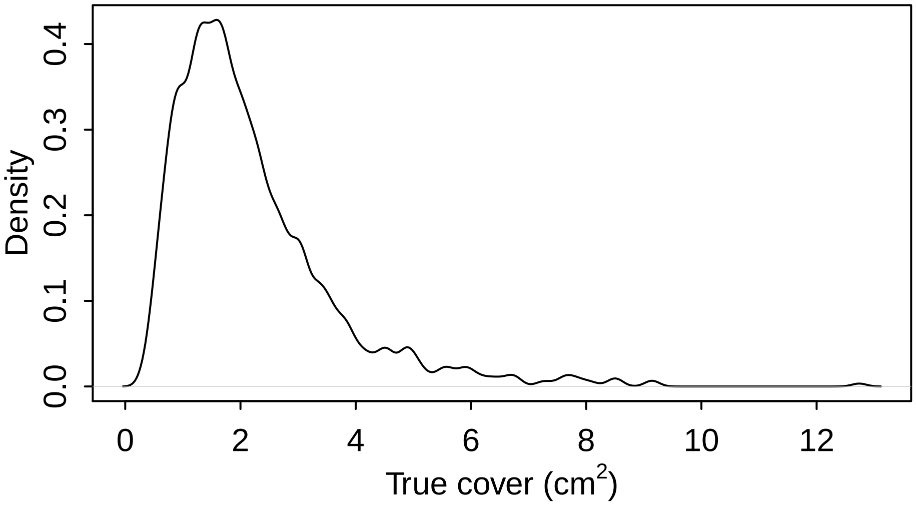

To account for the correlations between predictor variables in our dataset, we will sample predictor variables from the observed data.

```
# Sample predictor variables from the real data to keep their correlations
set.seed(321)
samp_id <- sample(1:nrow(dcore), N, replace = T)

## Temperature
temp <- dcore$tempLS[samp_id] # Sample temperature values
temp <- (temp - mean(temp))/sd(temp) # Scale values

## Moisture
moist <- dcore$moistLS[samp_id] # Sample moisterature values
moist <- (moist - mean(moist))/sd(moist) # Scale values

## Heterospecific crowding
wC <- dcore$w.cinter[samp_id]

## Conspecific crowding
w <- dcore$w[samp_id]
```

Now, let's assume the mean of the parameters estimated by the model are the true parameter values.

```
fpath_params <- paste("processed-data/params_", spcode, ".rds", sep = "")
params <- readRDS(fpath_params)

## Intercept
b0 <- mean(params$g.b0)

## Temperature effect
tef <- mean(params$g.tef) # Overall magnitude of temperature response
ttp <- mean(params$g.ttp)  # Thermal optimum (scaled units)
tsd <- mean(params$g.tsd)  # Thermal tolerance or niche breadth


rT <- tef*exp(-((temp - ttp)^2/2*tsd^2))
```

```r
### Plot hypothetical temperature effect
curve(tef*exp(-((x - ttp)^2/2*tsd^2)) + b0, -2, 1.5,
      ylab = "Effect on growth", xlab = "Temperature")
```

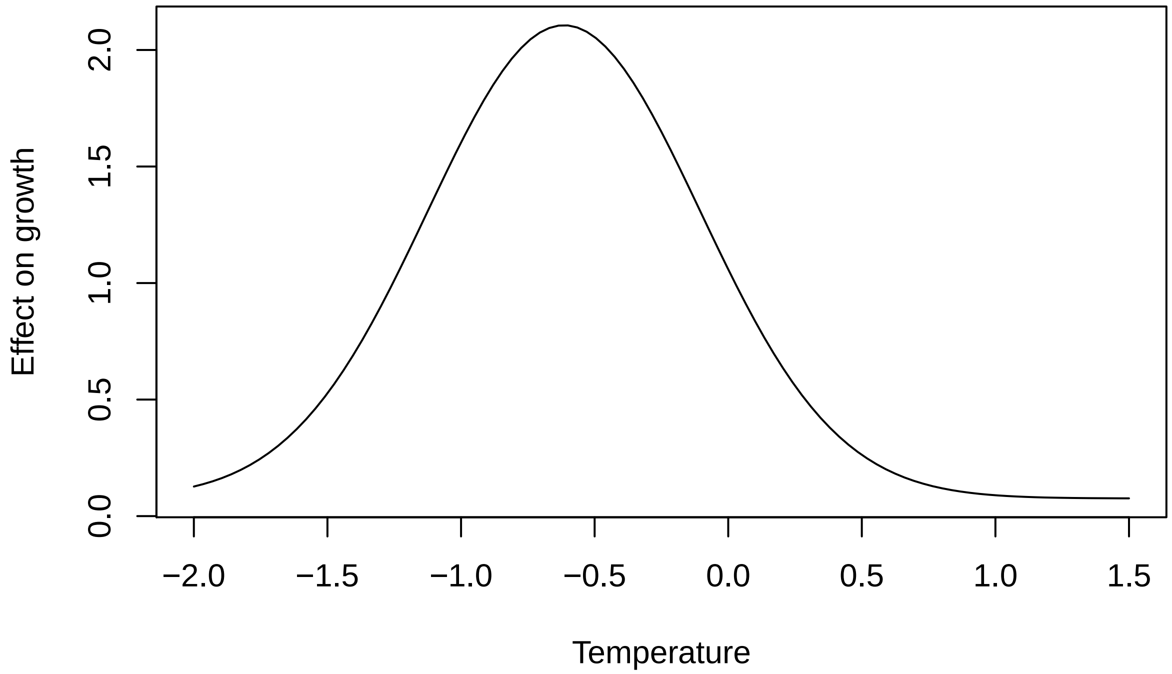


```r
## Response to heterospecific neighbors
aC_0 <- mean(params$g.aC_0)  # intercept (i.e., value at mean temperature)
b_aC <- mean(params$g.b_aC)  # response to temperature
aC <- exp(aC_0 + b_aC*temp)
summary(aC*mean(wC))
```

```
##    Min. 1st Qu.  Median    Mean 3rd Qu.    Max.
## 0.09503 0.22911 0.42701 0.37296 0.43862 0.48380
```

```r
## Response to conspecific neighbors
a_0 <- mean(params$g.a_0)  # intercept (i.e., value at mean temperature)
b_a <- mean(params$g.b_a)  # response to temperature
a <- exp(a_0 + b_a*temp)
summary(a*mean(w))
```

```
##    Min. 1st Qu.  Median    Mean 3rd Qu.    Max.
## 0.01572 0.01662 0.01728 0.01707 0.01731 0.01742
```

```r
## Effect of ramet cover (a reasonable range is within -0.3 and -0.45)
b <- mean(params$g.b)

## Growth variance
sigma <- mean(params$g.sigma)/10
```

Then, let's create a vector of scaled temperatures of the two previous summers.

```r
## Temperature
temp <- dcore$tempL2S[samp_id] # Sample temperature values
temp <- (temp - mean(temp))/sd(temp) # Scale values
```

Now we can simulate growth.

```r
# Simulate growth process
mu <- b0 + rT + log(u1)*b - a*w - aC*wC
g <- rnorm(N, mu, sigma)
summary(g)
```

```
##      Min.   1st Qu.    Median      Mean   3rd Qu.      Max.
## -0.930389  0.008897  1.137106  0.833160  1.391444  2.103539
```

```r
## Calculate true cover at time t+1
u2 <- u1*exp(g)

summary(u1)
```

```
##    Min. 1st Qu.  Median    Mean 3rd Qu.    Max.
##  0.3376  1.2608  1.8446  2.2097  2.7270 12.7384
```

```r
summary(u2)
```

```
##    Min. 1st Qu.  Median    Mean 3rd Qu.    Max.
##  0.4373  2.1490  5.2220  5.6809  8.0514 26.1702
```

```r
dens(u1, ylim = c(0,0.5))
dens(u2, col = "blue", add = T)
```

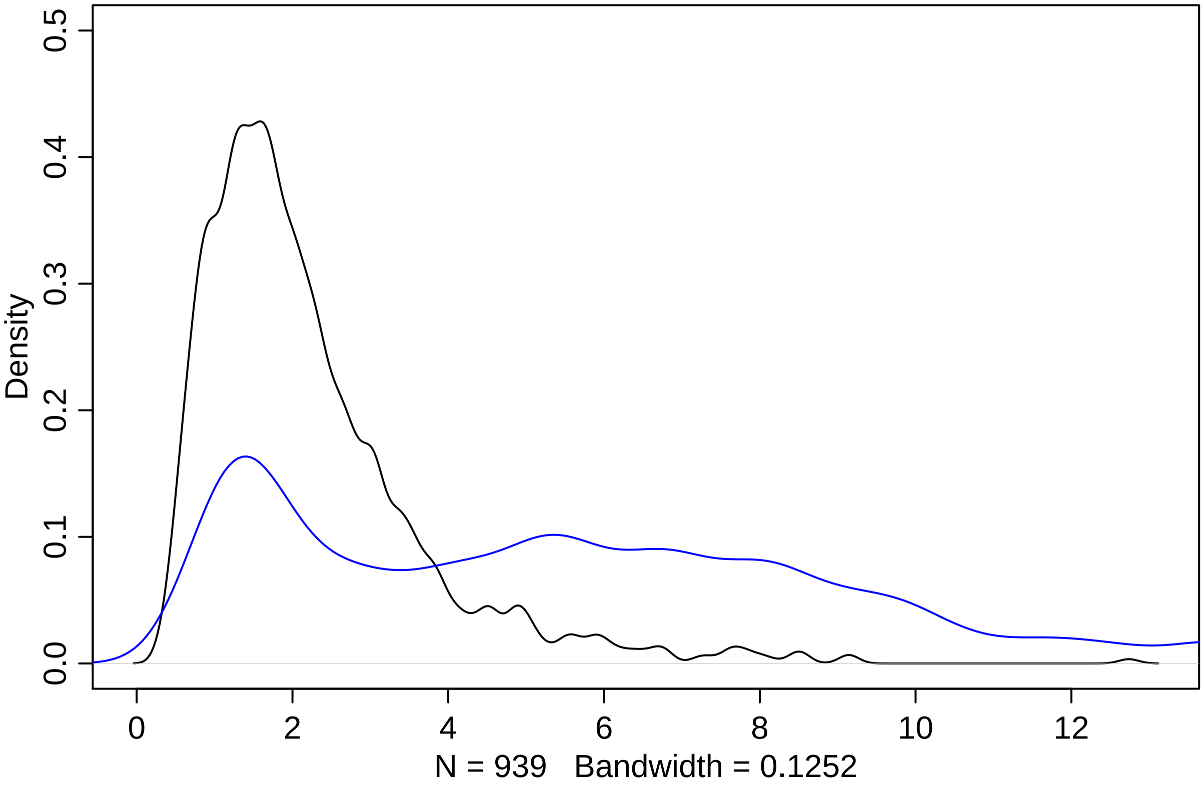

Now let's simulate the data collection process.

```r
## Create vector of observed covers at time 1,
## assuming observation error proportional to the square root of true cover
uobs1 <- u1 + rnorm(N, 0, sqrt(u1))

## Create vector of categorized observed covers (i.e., the available data)
```

```r
ut1 <- ifelse(uobs1 < 1.56, 1.56,
              ifelse(uobs1 < 3.125, 3.125,
                     ifelse(uobs1 < 6.25, 6.25,
                            ifelse(uobs1 < 12.5, 12.5,
                                   ifelse(uobs1 < 18.75, 18.75,
                                          ifelse(uobs1 < 25, 25,
                                                 25 + 6.25*ceiling((uobs1-25)/6.25)))))))



## Compare simulated with actually observed covers
summary(sp17$u1)
```

```
##    Min. 1st Qu.  Median    Mean 3rd Qu.    Max.
##   1.562   1.562   1.562   2.532   3.125  12.500
```

```r
summary(ut1)
```

```
##    Min. 1st Qu.  Median    Mean 3rd Qu.    Max.
##   1.560   1.560   3.125   3.558   6.250  18.750
```

```r
dens(ut1, col = "blue")
dens(sp17$u1, col = "red", add = T)
```

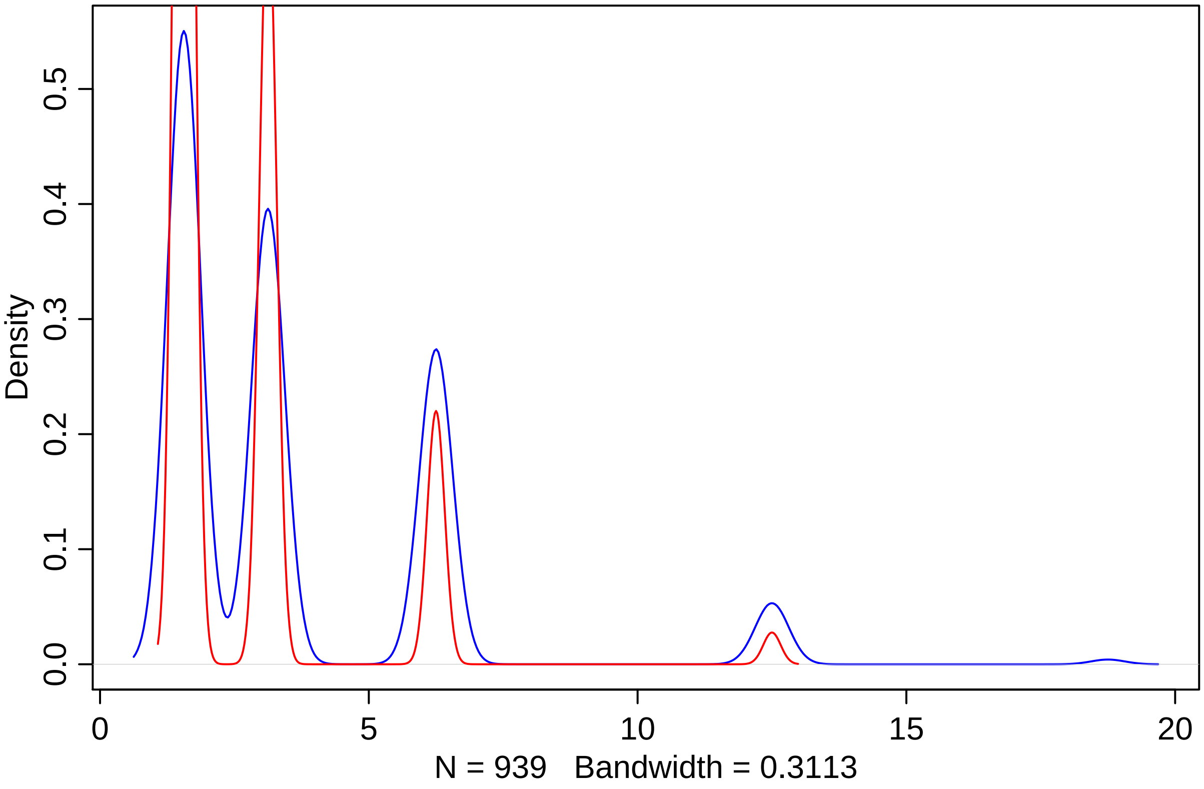


```r
## Create vector of observed covers at time 2
uobs2 <- u2 + rnorm(N, 0, sqrt(u2))

## Create vector of categorized observed covers (i.e., the available data)
ut2 <- ifelse(uobs2 < 1.56, 1.56,
              ifelse(uobs2 < 3.125, 3.125,
```

```
                          ifelse(uobs2 < 6.25, 6.25,
                                 ifelse(uobs2 < 12.5, 12.5,
                                        ifelse(uobs2 < 18.75, 18.75,
                                               ifelse(uobs2 < 25, 25,
                                                      25 + 6.25*ceiling((uobs2-25)/6.25)))))))
```

Finally, let's use the growth model to recover the true parameter values from the simulated data.

```
# Define lower bounds
ut2min <- ifelse(ut2 == (1.56), (0.25),
                 ifelse(ut2 == (3.125), (1.56),
                        ifelse(ut2 == (6.25), (3.125),
                               ifelse(ut2 == (12.5), (6.25),
                                      ifelse(ut2 == (18.75), (12.5),
                                             ifelse(ut2 == (25), (18.75),
                                                    (ut2 - 6.25)))))))

ut1min <- ifelse(ut1 == (1.56), (0.25),
                 ifelse(ut1 == (3.125), (1.56),
                        ifelse(ut1 == (6.25), (3.125),
                               ifelse(ut1 == (12.5), (6.25),
                                      ifelse(ut1 == (18.75), (12.5),
                                             ifelse(ut1 == (25), (18.75),
                                                    (ut1 - 6.25)))))))

# Data to train model
simdat <- list(
        u2max = ut2,
        u1max = ut1,
        u2min = ut2min,
        u1min = ut1min,
        wC = wC,
        w = w,
        temp = temp
)


# Model Stan code
gmodcode = "data{
        vector[howmany] u2max;
        vector[howmany] u1max;
        vector[howmany] u2min;
        vector[howmany] u1min;
        vector[howmany] wC;
        vector[howmany] w;
        vector[howmany] temp;
}
parameters{
        real b0;
        real<lower=0> tef;
        real<lower=0> tsd;
        real ttp;
        real b;
        real a_0;
```

```
        real b_a;
        real aC_0;
        real b_aC;
        real<lower=0> sigma;
        vector[howmany] u2;
        vector[howmany] u1;
}
model{
        vector[howmany] mu;
        vector[howmany] g;
        vector[howmany] rT;
        vector[howmany] a;
        vector[howmany] aC;

        tef ~ exponential( 1 );
        tsd ~ exponential( 0.5 );
        ttp ~ normal( 0 , 1.5 );

        for ( i in 1:howmany ) {
            rT[i] = tef*exp(-((temp[i] - ttp)^2/2*tsd^2));
        }

        b_aC ~ normal( 0 , 0.25 );
        aC_0 ~ normal( -6 , 1 );
        for ( i in 1:howmany ) {
            aC[i] = exp(aC_0 + b_aC * temp[i]);
        }

        b_a ~ normal( 0 , 0.25 );
        a_0 ~ normal( -6 , 1 );
        for ( i in 1:howmany ) {
            a[i] = exp(a_0 + b_a * temp[i]);
        }

        b0 ~ normal(-0.7, 0.2);

        b ~ normal(0, 0.05);

        sigma ~ exponential(1);

        u1 ~ normal(u1max - (u1max-u1min)/2, u1max/10);
        u2 ~ normal(u2max - (u2max-u2min)/2, u2max/10);

        for ( i in 1:howmany ) {
                g[i] = log(u2[i] / u1[i]);
                mu[i] = b0 + rT[i] + log(u1[i])*b - aC[i]*wC[i] - a[i]*w[i];
        }
        g ~ normal(mu , sigma );
}
"
gcodesp = gsub("howmany", N, gmodcode, fixed = T)
```

```r
## Initial values
initVals_gm <- list(list(u1 = rnorm(N, ut1 - (ut1-ut1min)/2, ut1/10),
                         mu = 0,
                         u2 = rnorm(N, ut2 - (ut2-ut2min)/2, ut2/10),
                         a_0 = -6,
                         b_a = 0,
                         aC_0 = -6,
                         b_aC = 0,
                         tef = 1,
                         tsd = 1,
                         ttp = 0,
                         b = -0.5,
                         b0 = -0.7),
                    list(u1 = rnorm(N, ut1 - (ut1-ut1min)/2, ut1/10),
                         mu = 0,
                         u2 = rnorm(N, ut2 - (ut2-ut2min)/2, ut2/10),
                         a_0 = -6,
                         b_a = 0,
                         aC_0 = -6,
                         b_aC = 0,
                         tef = 1,
                         tsd = 1,
                         ttp = 0,
                         b = -0.5,
                         b0 = -0.7))

gmod <- stan(model_code = gcodesp, data = simdat, chains = 2, cores = 2,
             init = initVals_gm, iter = 1500, warmup = 500,
             control = list(adapt_delta = 0.95, max_treedepth = 15))
```

Let's compare the prior and posterior distribution of the model parameters samples with the "true" values.

```r
postparams <- extract.samples(gmod)

# Figure prior, posterior, truth
par(mfrow = c(2,5))

dens(postparams$b0, xlim = c(-1.5,0.25), type = 'n')
polygon(density(rnorm(4e3, -0.7, 0.2)),
        col = col.alpha("gray", 0.65))
polygon(density(postparams$b0),
        col = col.alpha("black", 0.65))
abline(v=b0, lty = 3, lwd = 1.5)
mtext('Intercept (b0)', cex = 0.75)


dens(postparams$tef, xlim = c(-0.5,6), type = 'n')
polygon(density(rexp(4e3, 1)),
        col = col.alpha("gray", 0.65))
polygon(density(postparams$tef),
        col = col.alpha("black", 0.65))
abline(v=tef, lty = 3, lwd = 1.5)
mtext('Thermal response (tef)', cex = 0.75)
text(4.2, 1.5, "Posterior")
```

```r
text(0.55, 0.85, "Prior", col = "darkgray")
#

dens(postparams$tsd, xlim = c(0,4), type = 'n')
polygon(density(rexp(4e3, 0.5)),
        col = col.alpha("gray", 0.65))
polygon(density(postparams$tsd),
        col = col.alpha("black", 0.65))
abline(v=tsd, lty = 3, lwd = 1.5)
mtext('Thermal tolerance (tsd)', cex = 0.75)

dens(postparams$ttp, xlim = c(-3,3), type = 'n')
polygon(density(rnorm(4e3, 0, 0.5)),
        col = col.alpha("gray", 0.65))
polygon(density(postparams$ttp),
        col = col.alpha("black", 0.65))
abline(v=ttp, lty = 3, lwd = 1.5)
mtext('Thermal optimum (ttp)', cex = 0.75)

dens(postparams$b, xlim = c(-0.5,0), type = 'n')
polygon(density(rnorm(4e3, 0, 0.1)),
        col = col.alpha("gray", 0.65))
polygon(density(postparams$b),
        col = col.alpha("black", 0.65))
abline(v=b, lty = 3, lwd = 1.5)
mtext('Size effect (b)', cex = 0.75)
text(b-0.05, 7, "True parameter value", srt = 90)

dens(postparams$a_0, xlim = c(-10,-2), type = 'n')
polygon(density(rnorm(4e3, -5, 0.5)),
        col = col.alpha("gray", 0.65))
polygon(density(postparams$a_0),
        col = col.alpha("black", 0.65))
abline(v=a_0, lty = 3, lwd = 1.5)
mtext('a_0', cex = 0.75)

dens(postparams$b_a, xlim = c(-1,1), type = 'n')
polygon(density(rnorm(4e3, 0, 0.25)),
        col = col.alpha("gray", 0.65))
polygon(density(postparams$b_a),
        col = col.alpha("black", 0.65))
abline(v=b_a, lty = 3, lwd = 1.5)
mtext('b_a', cex = 0.75)

dens(postparams$aC_0, xlim = c(-10,-2), type = 'n')
polygon(density(rnorm(4e3, -5, 0.5)),
        col = col.alpha("gray", 0.65))
polygon(density(postparams$aC_0),
        col = col.alpha("black", 0.65))
abline(v=aC_0, lty = 3, lwd = 1.5)
mtext('aC_0', cex = 0.75)

dens(postparams$b_aC, xlim = c(-1,1), type = 'n')
```

```r
polygon(density(rnorm(4e3, 0, 0.25)),
        col = col.alpha("gray", 0.65))
polygon(density(postparams$b_aC),
        col = col.alpha("black", 0.65))
abline(v=b_aC, lty = 3, lwd = 1.5)
mtext('b_aC', cex = 0.75)


dens(postparams$sigma, xlim = c(-0.5,2), type = 'n')
polygon(density(rexp(4e3, 1)),
        col = col.alpha("gray", 0.65))
polygon(density(postparams$sigma),
        col = col.alpha("black", 0.65))
abline(v=sigma, lty = 3, lwd = 1.5)
mtext('sigma', cex = 0.75)
```

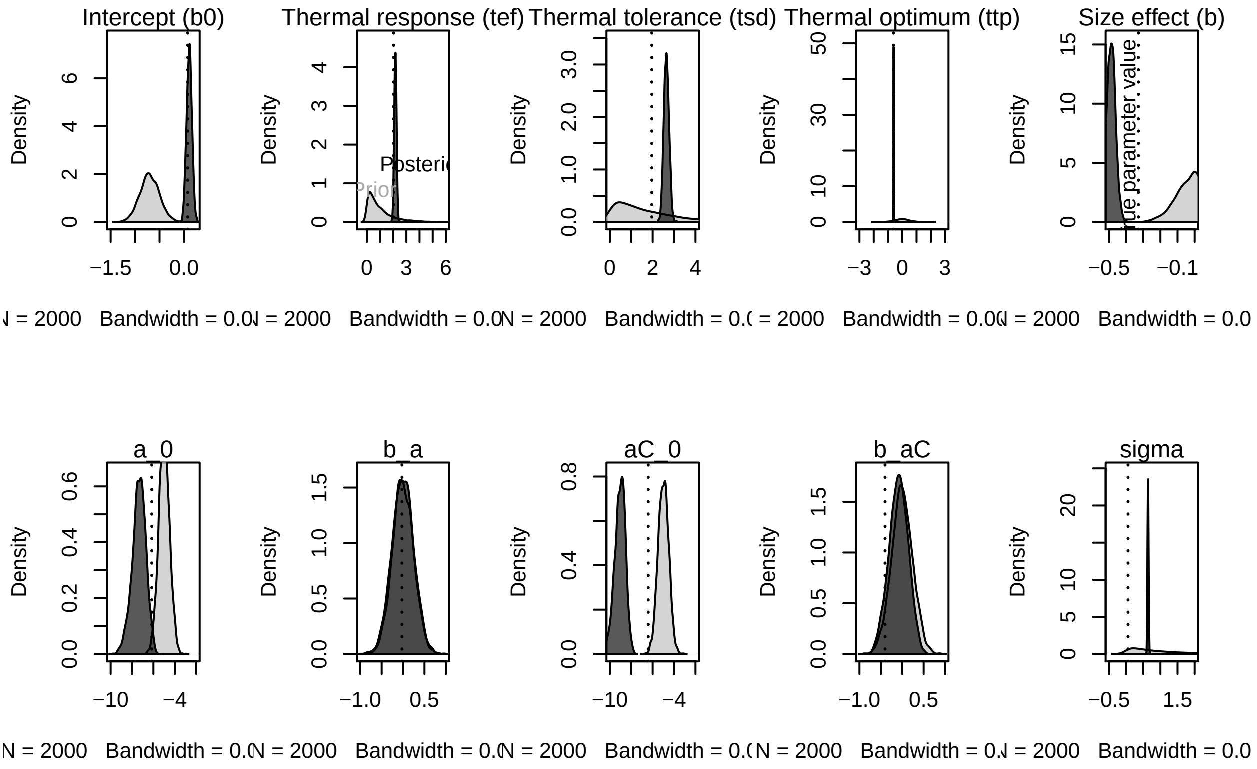

Despite a very accurate estimation of some parameters, there is a clear overestimation bias for parameter `tsd`, as well as underestimation biases for neighbor interaction parameters `a_0` and `aC_0`. We thus excluded *Salix retusa* from downstream analyses.
