# Supplementary material for: Ecological lags govern the pace and outcome of plant community responses to 21st‐century climate change
Source: Ecol Lett. 2022 Aug 26;25(10):2156–66. doi: 10.1111/ele.14087 (PMC9804264; doi:10.1111/ele.14087)
Supplement: Supplementary file 5 — Appendix S5 [file ELE-25-2156-s004.docx]

**SM5: Supplementary results**

*Taxa cover and frequency dynamics under contrasting climate change scenarios*

**
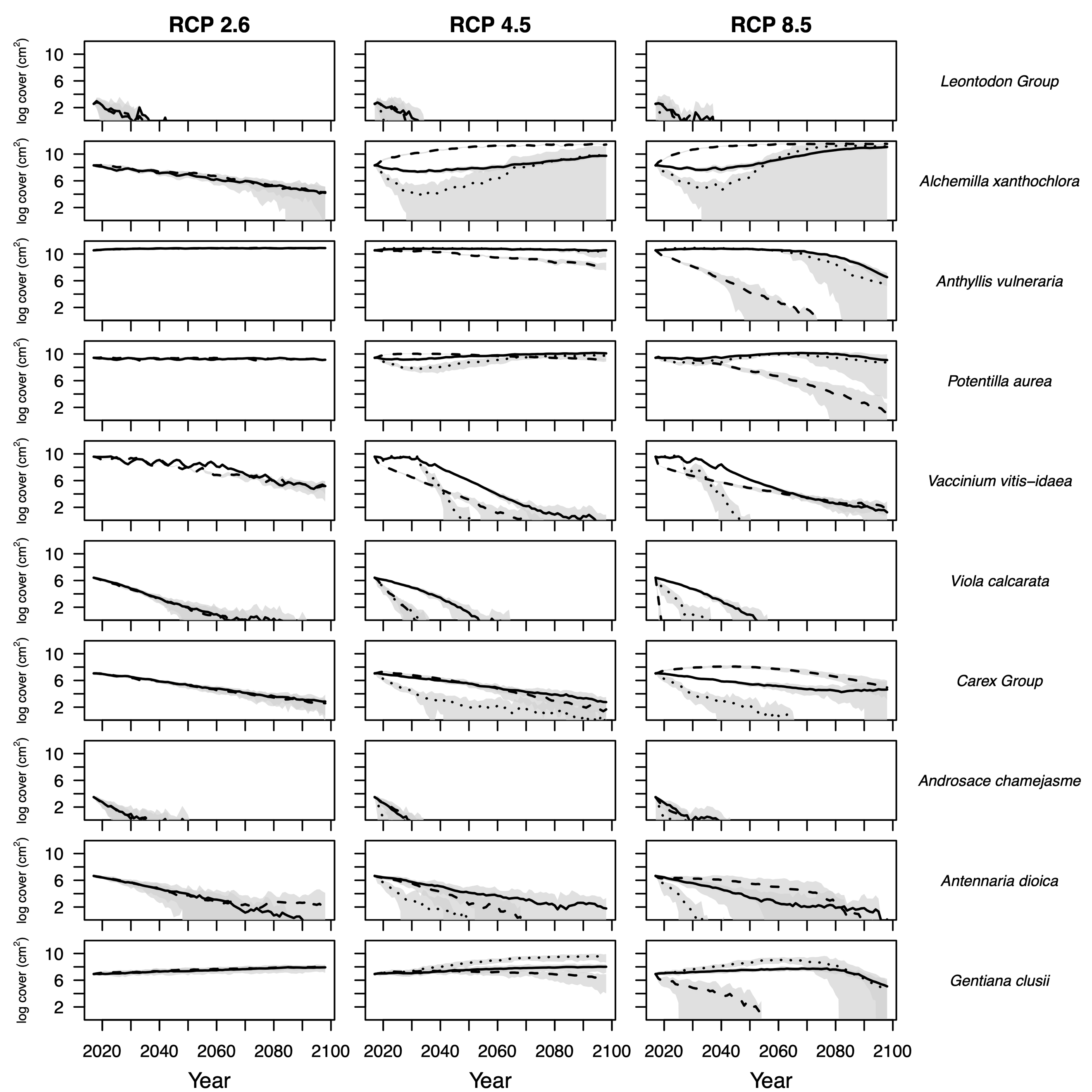
**

**Figure S5.1.** Taxa cover dynamics under different climate change scenarios with (solid lines) or without (dotted lines) the full influence of demographic and competitive lags. The dynamics following stepwise change to conditions expected by the end of the century under the respective scenarios are shown with dashed lines. Lines show the median log cover and shaded regions show the 5% and 95% quantiles across 20 simulations.

**
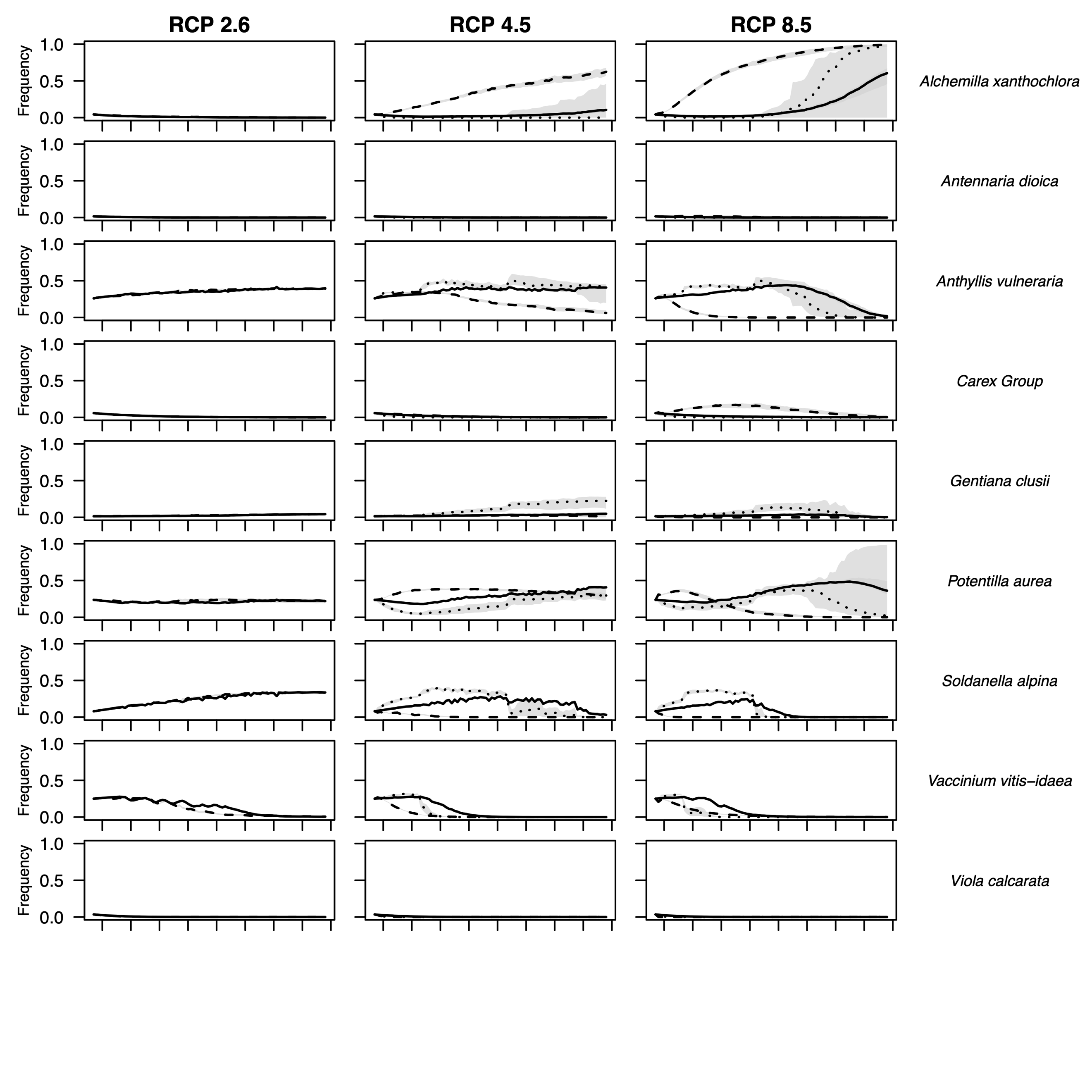
**

**Figure S5.2.** Frequency dynamics under different climate change scenarios with (solid lines) or without (dotted lines) the full influence of demographic and competitive lags. The dynamics following stepwise change to conditions expected by the end of the century under the respective scenarios are shown with dashed lines. Lines show the median log cover and shaded regions show the 5% and 95% quantiles across 20 simulations.

*Climate-dependency of taxa demography and interactions with neighbors*

The fitted relationships between each taxon’s demography, climate, and interactions with neighbors help understand how demographic and competitive lags arise and shape the trajectories of community dynamics. For example, the decline of *Anthyllis vulneraria* expected after 2050 under RCP scenario 8.5 (or immediately after stepwise change to end-of-century RCP 4.5 climate) can be partly explained by the direct negative effects of warming on vital rates, and by the effects of warming on survival mediated by its reduced tolerance to crowding by heterospecific neighbors (Fig. S5.3.1C). The summer temperatures at which *Anthyllis*’ survival became increasingly sensitive to competition (between 15 and 16 ºC) were the same at which the growth of other taxa, such as *Alchemilla*, peaked, which exacerbated *Anthyllis*’ demise. In contrast, the demise of *Potentilla* under severe warming can be explained by direct negative climatic effects on growth and recruitment, as well as by an increased sensitivity of survival to competition with heterospecifics (Fig. S5.3.1 D, I, and N).

**
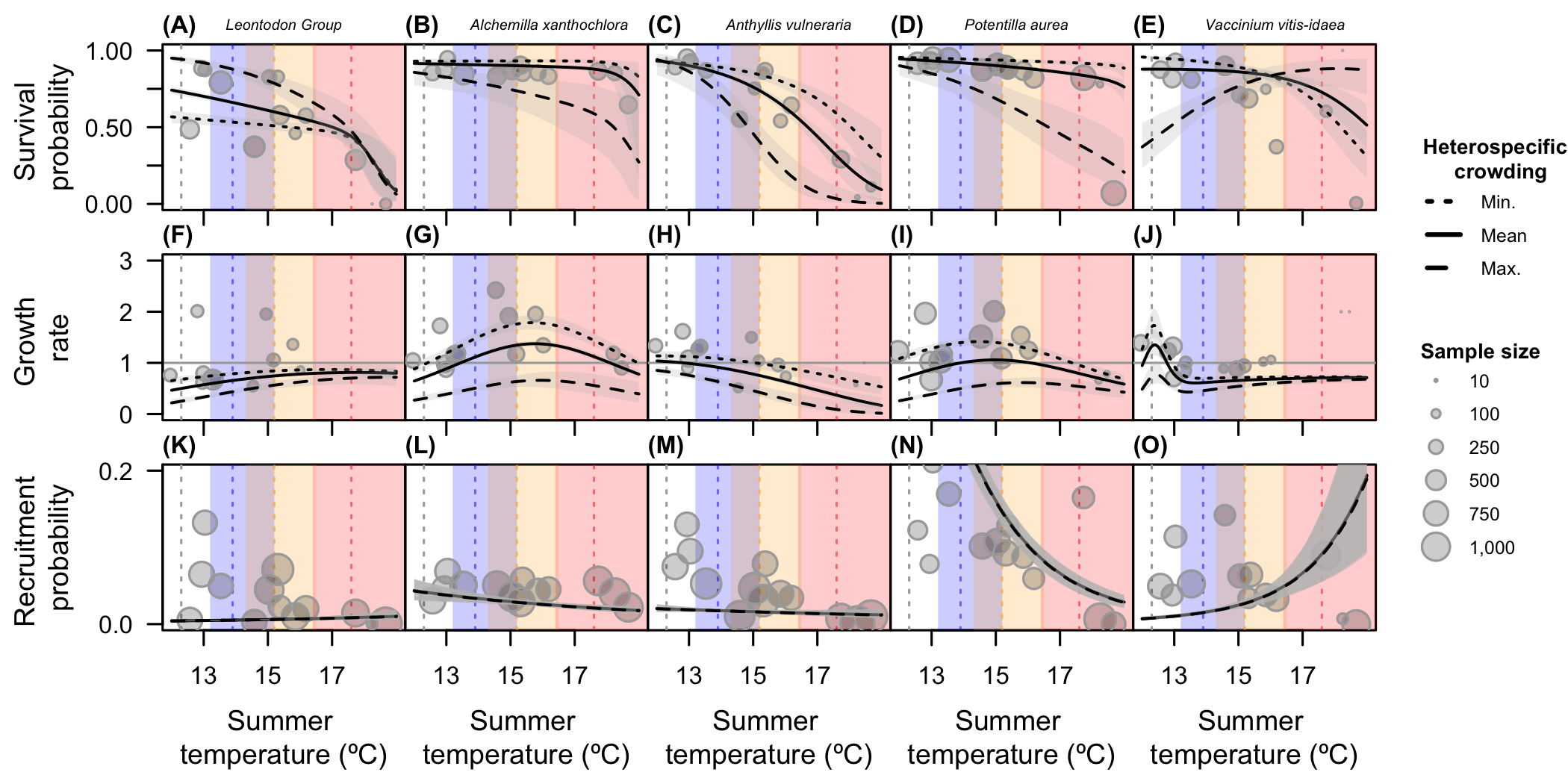
**

**Figure S5.3.1. Temperature-dependence of species’ demography and interactions.** Lines show the average model predictions of survival probability (top row), growth rate (middle row), and recruitment probability (bottom row). For all demographic rates, their temperature dependence is shown under the mean (solid lines), minimum (dotted lines), or maximum (dashed lines) observed levels of heterospecific crowding. Thus, the relative position of these lines shows how the effect of heterospecific neighbors changes with temperature (conspecific crowding was always held constant at its average value). Predictions of survival and growth rate are for a ramet of average size for that species’, and under average moisture (relevant for survival). The shaded region around each line represents 89 % confidence interval around the mean predictions. The gray points show the observed demographic rates observed at different temperatures during the survey period (i.e., values encompass variation across elevations and across years). The size of the points is proportional to the number of ramets (for survival and growth models) or empty quadrants (for recruitment models) observed at each temperature level. The vertical, gray, dashed line shows current mean summer temperature at the 2050-m focal community, while the range of likely summer temperatures expected in the region by year 2085 under RCP scenarios 2.6, 4.5, and 8.5 are depicted by the shaded blue, yellow, and red regions respectively (the corresponding vertical dashed lines show median forecasted temperatures).

**
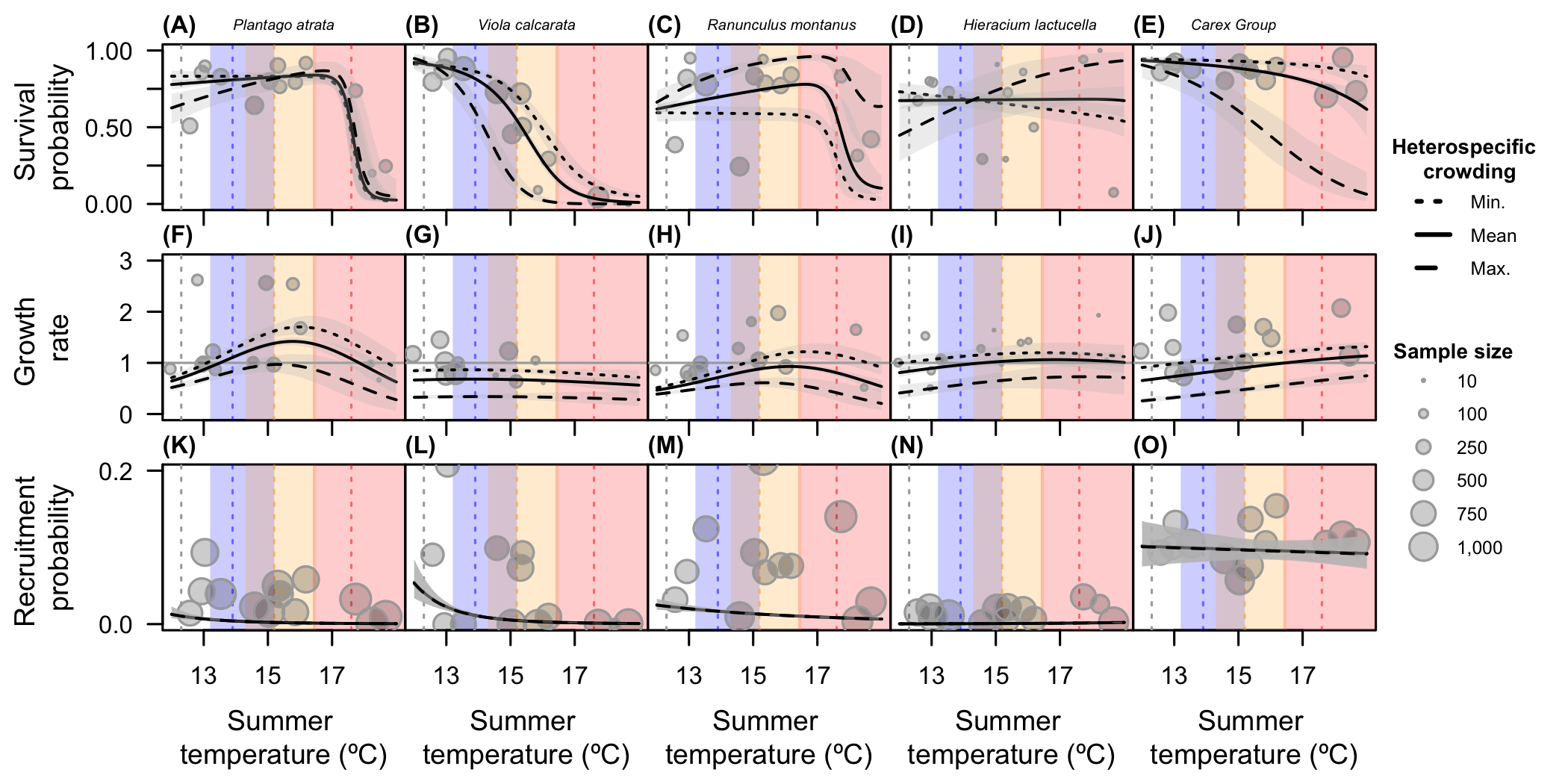
**

**Figure S5.3.2**

**
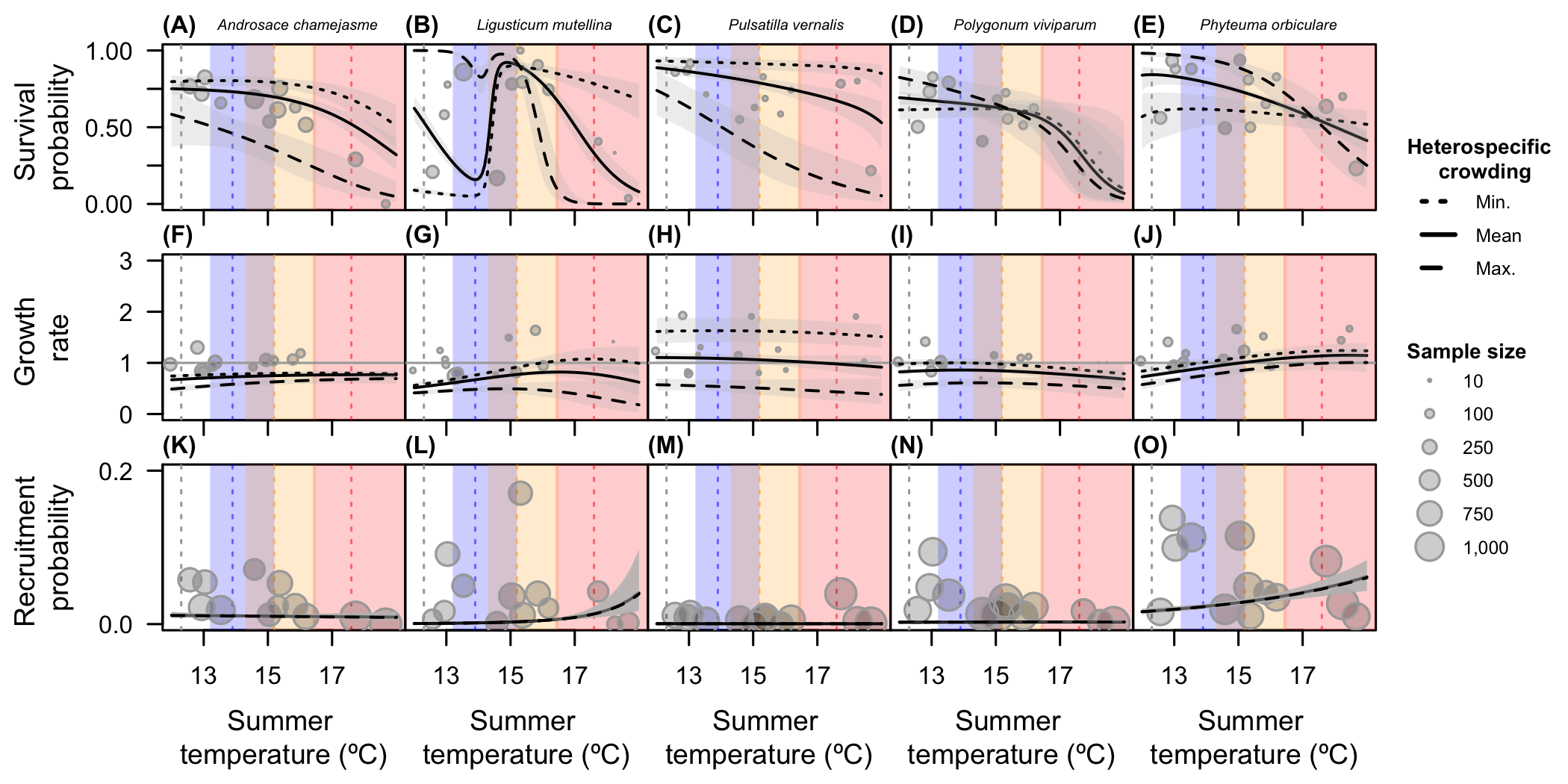
**

**Figure S5.3.3**

**
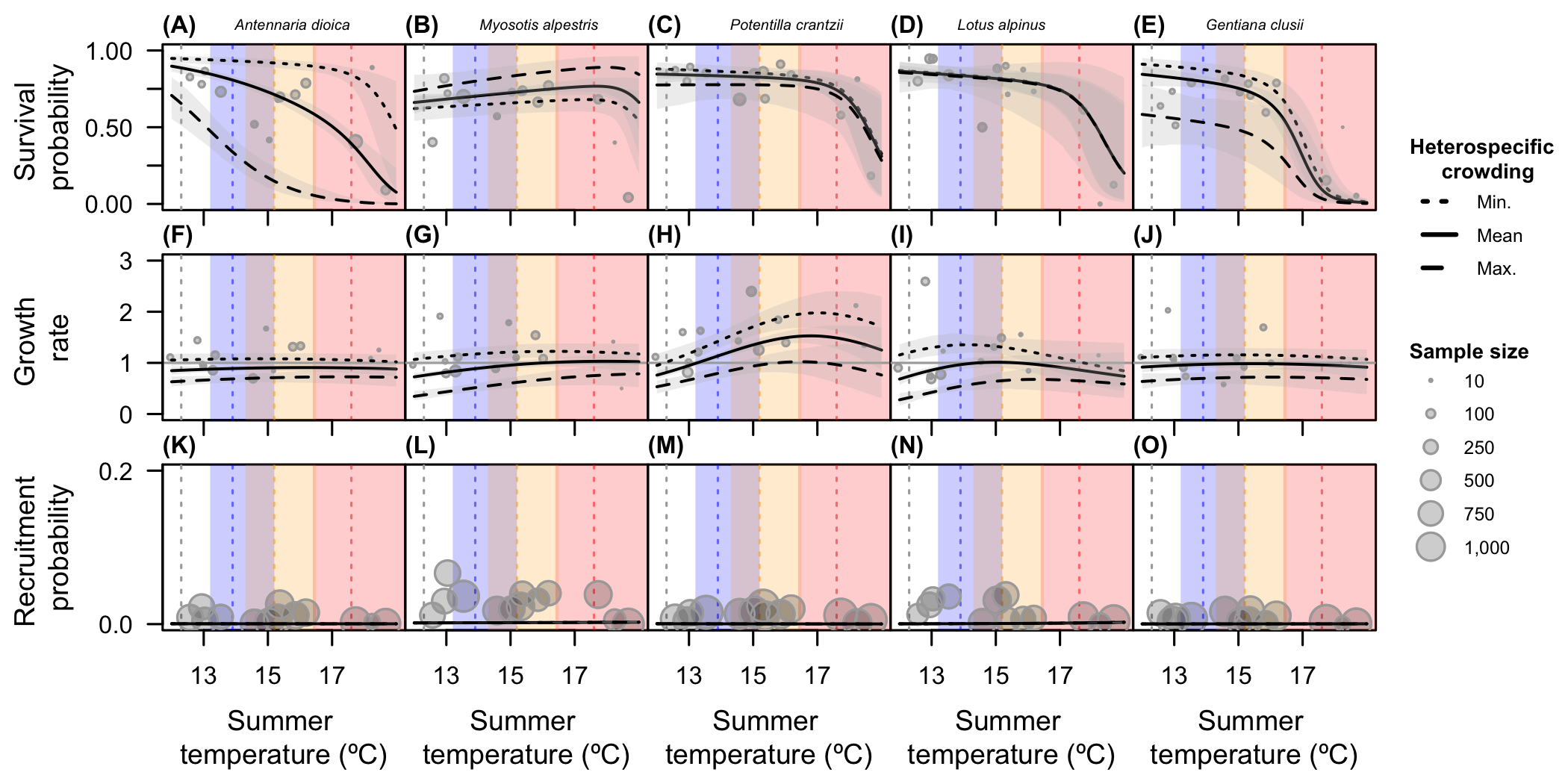
**

**Figure S5.3.4**

**
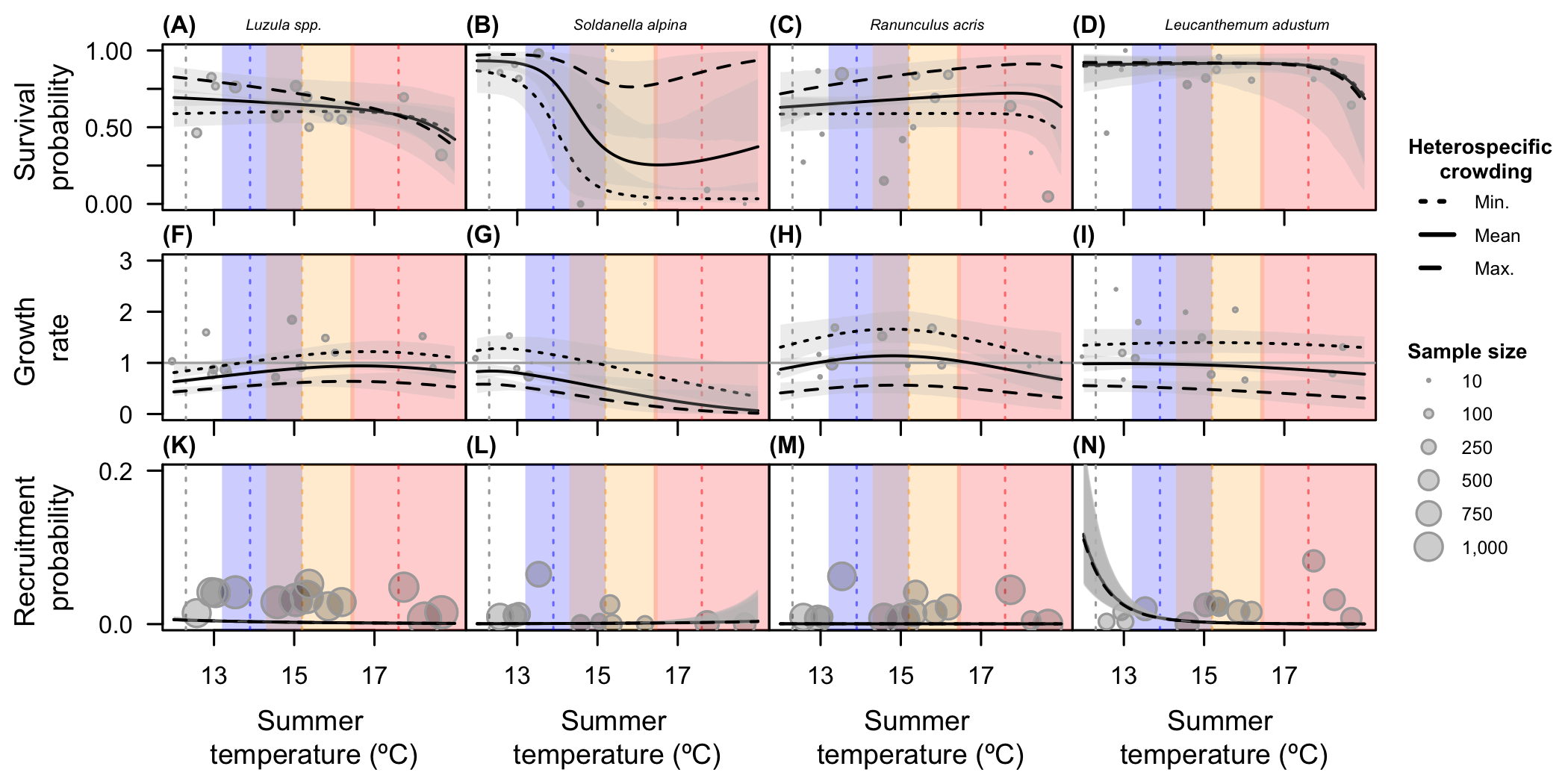
**

**Figure S5.3.5**

**
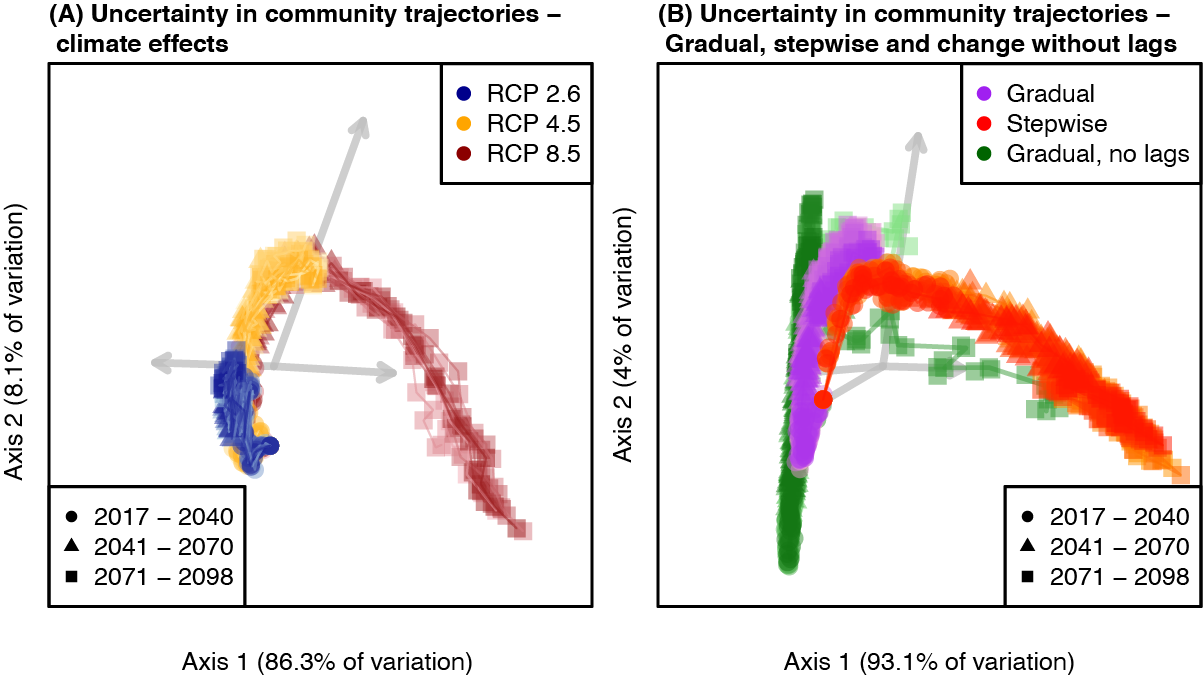
**

**Figure S5.4.** Uncertainty in community trajectories due to demographic stochasticity across the repeated simulations generating results in main text Figures 2C and 4A. Here, 10 of the 20 total simulations (chosen with equal probability) are shown on the same ordination axis as used in Figure 2C (panel A) and Figure 4A (panel B).
